# Phytosomal curcumin causes natural killer cell-dependent repolarization of glioblastoma (GBM) tumor-associated microglia/macrophages and elimination of GBM and GBM stem cells

**DOI:** 10.1186/s13046-018-0792-5

**Published:** 2018-07-25

**Authors:** Sumit Mukherjee, Angela Fried, Rahman Hussaini, Richard White, Juliet Baidoo, Sri Yalamanchi, Probal Banerjee

**Affiliations:** 1 CUNY Doctoral Program in Biochemistry, CUNY Graduate Center, 365 Fifth Avenue, New York, NY 10016 USA; 2 Department of Chemistry, City University of New York at The College of Staten Island, Building 6S, 2800 Victory Boulevard, Staten Island, NY 10314 USA; 3 Center for Developmental Neuroscience, Building 6S, City University of New York at The College of Staten Island, 2800 Victory Boulevard, Staten Island, NY 10314 USA

**Keywords:** Phytosomal curcumin, Tumor-associated microglia/macrophages (TAM), NK cells, Glioblastoma (GBM), GBM stem cells, MCP-1, Oncoimmunotherapy

## Abstract

**Background:**

Glioblastoma (GBM) is a primary brain tumor with a 5-year survival rate of ≤5%. We have shown earlier that GBM-antibody-linked curcumin (CC) and also phytosomal curcumin (CCP) rescue 50–60% of GBM-bearing mice while repolarizing the tumor-associated microglia/macrophages (TAM) from the tumor-promoting M2-type to the tumoricidal M1-type. However, systemic application of CCP yields only sub-IC50 concentrations of CC in the plasma, which is unlikely to kill GBM cells directly. This study investigates the role of CC-evoked intra-GBM recruitment of activated natural killer (NK) cells in the elimination of GBM and GBM stem cells.

**Methods:**

We have used an immune-competent syngeneic C57BL6 mouse model with the mouse-GBM GL261 cells orthotopically implanted in the brain. Using immunohistochemistry and flow cytometry, we have quantitatively analyzed the role of the intra-GBM-recruited NK cells by (i) injecting (i.p.) the NK1.1 antibody (NK1.1Ab) to temporarily eliminate the NK cells and (ii) blocking NK recruitment by injecting an IL12 antibody (IL12Ab). The treatment cohorts used randomly-chosen GL261-implanted mice and data sets were compared using two-tailed t-test or ANOVA.

**Results:**

CCP treatment caused the GBM tumor to acquire M1-type macrophages (50–60% of the TAM) and activated NK cells. The treatment also elicited (a) suppression of the M2-linked tumor-promoting proteins STAT3, ARG1, and IL10, (b) induction of the M1-linked anti-tumor proteins STAT1 and inducible nitric oxide synthase in the TAM, (c) elimination of CD133(+) GBM stem cells, and (d) activation of caspase3 in the GBM cells. Eliminating intra-GBM NK cell recruitment caused a partial reversal of each of these effects. Concomitantly, we observed a CCP-evoked dramatic induction of the chemokine monocyte chemotactic protein-1 (MCP-1) in the TAM.

**Conclusions:**

The recruited NK cells mediate a major part of the CCP-evoked elimination of GBM and GBM stem cells and stabilization of the TAM in the M1-like state. MCP-1 is known to activate peripheral M1-type macrophages to secrete IL12, an activator of NK cells. Based on such observations, we postulate that by binding to peripheral M1-type macrophages and IL12-activated NK cells, the brain-released chemokine MCP-1 causes recruitment of peripheral immune cells into the GBM, thereby causing destruction of the GBM cells and GBM stem cells.

**Electronic supplementary material:**

The online version of this article (10.1186/s13046-018-0792-5) contains supplementary material, which is available to authorized users.

## Background

Glioblastoma (GBM) is a deadly form of primary brain tumor with life expectancy of barely 12–15 months from detection, despite the available therapeutic regimens [1, 2]. After surgical excision of the tumor, most GBM patients are treated with radiation plus chemotherapy for a few weeks, which is followed by a chemotherapy regimen that involves a 5-day cycle of 150–200 mg/m^2^/day temozolomide (TMZ) every 28 days [1, 3]. This 5-day cycle is applied every 28 days as opposed to a prolonged treatment with lower doses (e.g. 75 mg/m^2^/day) because prolonged TMZ chemotherapy yields severe lymphopenia and suppression of the immune system [4, 5] and development of chemotherapy resistance by the cancer cells [6, 7]. To avoid such side effects, we investigated therapeutic strategies that would rather utilize the body’s own immune system for GBM elimination. Although the xenograft model in *nu/nu* mice has been used by many groups to study direct anticancer effects of chemotherapeutic agents after implanting human cancer cells, these mice lack T cells and therefore cannot be used to study the effect of the immune system. Therefore, instead of using xenografts in immunocompromised mice, we employed the widely used immunocompetent, syngeneic C57BL6 mouse model with orthotopically implanted mouse glioblastoma GL261 cells [8–10].

The food-derived anticancer agent curcumin (CC) has been shown to eliminate chemotherapy resistance of cancer cells via multiple mechanisms [11–13]. However, CC per se has poor bioavailability in vivo*,* which may have rendered it ineffective as an anticancer agent in clinical trials [14–16]. Nonetheless, during the last decade we have developed and tested various highly effective delivery forms of CC [10, 17–19]. In one of our recent studies, a bioavailable, phytosomal version of CC (Curcumin Phytosome Meriva (CCP)) (see further details in section “Intra-peritoneal delivery of Curcumin Phytosome Meriva (CCP) into GL261-implanted mice”) [20–23] and also an antibody-linked CC pro-drug caused complete remission in 50–60% of GL261-implanted GBM mice [8, 18].

Although CCP exhibited higher bioavailability for CC than free CC, oral gavage of CCP was reported to yield only 0.019 μΜ of CC in the plasma [21], which was far below the IC50 of CC obtained from in vitro cell-culture studies for GL261 (15 μM) [8]. Yet, CCP treatment caused GBM elimination and rescue of 60% of the orthotopically GL261-implanted mice [8]. As a clue to this surprising finding, we also observed a CCP-evoked dramatic repolarization of tumor-associated microglia/macrophages (TAM) from the tumor-promoting and immunosuppressive M2-like state to the M1 state [8, 10, 24–27]. This observation was important because innate immune cells like microglia and macrophages are the first line of defense against pathogens and tumors [28]. Additionally, it is also known that among the host of immune cells, the brain primarily harbors microglia, which in their pro-inflammatory M1 state can kill tumors directly as well as indirectly by functioning as specialized antigen-presenting cells and via activation and recruitment of other tumoricidal innate immune cells like Natural Killer (NK) cells and peripheral M1-type macrophages [8, 10, 25, 29–31]. Earlier studies have also shown that in GBM, a major portion of the tumor mass is constituted of M2-type TAM [8, 10, 32, 33]. Therefore, skewing the phenotype of TAM to M1-like state by therapeutic interventions holds immense promise in the context of GBM immunotherapy. In light of such information, we elucidate here that in addition to its direct cancer cell-selective activity [10, 34, 35], CC as CCP functions to cause repolarization of the tumor-associated M2-type microglia and intra-tumor recruitment of tumoricidal M1 macrophages and activated natural killer (NK) cells. The NK cells are highly tumoricidal and cause stabilization of M1-type TAM [29]. By treating GBM-harboring mice with CCP, with or without eliminating peripheral NK cells, we show that CCP-evoked intra-GBM recruitment of activated NK cells play a major role in augmenting the CCP-mediated repolarization of TAM from M2 to M1-like state along with elimination of GBM cells and GBM stem cells. To our knowledge, this is the first quantitative mechanistic analysis demonstrating the role of NK cells in GBM tumors. It is expected that CCP-mediated activation of these tumoricidal immune cells are primarily responsible in bringing about the immunotherapeutic remission of the GBM-harboring mice [8]. Also, the elimination of GBM stem cells is particularly important because prior studies have shown that the rarely dividing and chemo-resistant GBM stem cells promote radio-resistance [36, 37], and are stimulated to multiply following exposure to ionizing radiation [38]. Consequently, the GBM reappears even after surgical resection and overpowers the already immuno-compromised GBM patient [4, 5]. This study also throws new light on the relationship between the brain tumor microenvironment and the peripheral innate immune system. Inspired by the promises of cancer immunotherapy [39–42], our research elucidates an innovative, safe and simple approach of turning the innate immune system against GBM.

## Methods

### Animals

Adult C57BL/6 male mice (2–6 months old) were used for our experiments. Animals were bred in the College of Staten Island (CSI) Animal Care Facility and maintained on a 12-h light/dark cycle with ad libitum access to food and water. All animals were handled and used for surgery following an animal protocol approved by the Institutional Animal Care Committee (IACUC) of CSI (CUNY) (approval # 11–008).

### Cell culture

GL261 mouse glioblastoma cells were cultured according to our earlier reports [8, 10].

### Implantation of cancer cells in mice

GL261 mouse glioblastoma cells (10^5^) were implanted in mice according to our earlier report on day 1 [8, 10].

### Research design

Our earlier studies have established a condition to generate an orthotopic GBM tumor in 100% of the GL261-implanted C57BL6 mice (a syngeneic mouse model) [8, 10, 18]. Using this method, GBM-harboring mice were generated and then randomly divided into multiple cohorts for Vehicle or drug treatments as discussed below. Although our prior studies had demonstrated the generation of GBM tumor by sacrificing parallel groups of GL261-implanted mice, in the experiments included here, during drug treatment, the experimenter was completely blinded from the actual status of a GBM tumor in any GL261-implanted mouse placed in any of these cohorts. After extrication of the GBM-harboring brains from all the groups (as described below), all the brains were subjected to near-IR scanning to ensure the presence of established tumors (as detailed below) [8, 10, 18].

### Neutralization of NK cells by intra-peritoneal infusion of NK1.1 ab

On day 11 from implantation of 10^5^ GL261 cells (on day 1), each mouse in the CCP + NK1.1 group received intra-peritoneal infusion of NK cell-neutralizing anti-NK1.1 antibody (PK136) (Mouse IgG) (BD, Catalog# 553162) (100 μg). Mice in the other two groups (Vehicle and CCP) received intra-peritoneal injection of a mixture of normal mouse immunoglobulins (Invitrogen, Catalog# 31881) (100 μg). As a proof of NK cell elimination, GBM mice from a fourth group, dubbed ‘NK1.1’, received the NK cell-neutralizing anti-NK1.1 antibody (PK136) (i.p., 100 μg) on day 11, followed by Vehicle (PBS). After five injections of Vehicle or CCP, each mouse was sacrificed and the GBM brain tumor was divided into two parts for immunohistochemistry (IHC) and flow cytometry analysis.

### Neutralization of IL12-mediated signaling on NK cells by intra-peritoneal infusion of IL12 ab

On day 11 and day 14 from implantation of 10^5^ GL261 cells, each mouse in the CCP + IL12Ab group received intra-peritoneal infusion of the IL12-neutralizing Anti-IL12 (p40/p70) antibody (rat IgG) (BD, Catalog# 554475) (100 μg per mouse) [27, 29]. Mice in the other two groups (Vehicle and CCP) received a mixture of normal rat immunoglobulins (100 μg, Thermo Fisher Scientific, Catalog# 31888) and subsequent reagents from day 12 (Vehicle or CCP) similar to the earlier NK1.1Ab treatment experiment. After five injections of Vehicle or CCP, each mouse was sacrificed and the GBM brain tumors were divided into two parts for IHC and flow cytometry analysis.

### Intra-peritoneal delivery of Curcumin Phytosome Meriva (CCP) into GL261-implanted mice

Curcumin Phytosome Meriva (CCP) is a formulation with higher bioavailability of curcuminoids (CC) than free CC, prepared by allowing a defined mass of CC to bind (in an organic solvent) to an equimolar mass of phosphatidylcholine (PC) through hydrogen bonding between the hydroxy groups of CC and the polar head group of PC. Upon removal of the organic solvent and dispersion in an aqueous medium the hydrophobic side chains of PC were expected to wrap around the hydrophobic domains of CC to keep it protected and improve the stability and bioavailability of CC in vivo [20, 22, 23]. The commercially available CCP capsule (each containing 500 mg of solid) contained 96 mg of PC-bound CC along with excipients [8]. For the long-term experiments that resulted in rescue of GBM mice, intra-peritoneal (i.p.) administration of CCP was performed according our earlier report [8].

For the short-term experiments, daily intra-peritoneal (i.p.) administration of CCP was initiated from day 12 and continued until day 16. Each 30-g mouse received i.p. injections of sterile PBS (Vehicle group) or a CCP emulsion (for the cohorts “CCP”, “CCP + NK1.1Ab”, and “CCP + IL12Ab”) containing 2 mg of CCP in 200 μl of PBS every 24 h for five days. CCP was dispersed by vortexing vigorously in 200 μl of sterile PBS, the insoluble solids were allowed to settle for 2 min, and then the translucent supernatant was used for i.p. injection into each mouse.

### Preparation of Dylight800-CD68Ab and post-mortem determination of GL261-evoked GBM tumor load

Dylight800-CD68Ab was synthesized according to our earlier report [8, 10]. To monitor tumor load, each mouse was anesthetized, intranasally (IN) treated with the Dylight800-CD68Ab adduct (containing 60 μg (400 pmole) of CD68 antibody (CD68Ab)) on day 16 (post-implantation) as described earlier [8, 10], and then scanned after 24 h (following anesthesia) using the Odyssey near-IR scanner (LI-COR Biosciences, Nebraska). The Dylight800 fluorescence (tumor load, pseudocolored green) and the enhanced autofluorescence of the brain tissue (pseudocolored red) were imaged to ensure the presence of established tumors in all experimental groups [8, 18]. Thus, The combination of green and red fluorescence at the tumor site appeared as yellow. The tumor tissue along with the peripheral areas were extricated and processed for further analyses as described below [8, 10].

### Immunohistochemistry of brain tumors and scar tissue

The IHC part of the established GBM obtained from the day-17 brain was fixed in 4% paraformaldehyde (PFA), soaked in 30% sucrose, and coronal sections (30 μm) were prepared [8, 10, 18]. In both the long-term and short-term studies, randomly chosen sections were subjected to a pre-immunostaining antigen-retrieval process using formamide:2xSSC (1:1) as reported earlier [8] except for Iba1/IL12/IL10 staining experiments. Antigen-retrieval for the Iba1/IL10/IL12 sets were performed by incubating the sections in 0.1% (*w*/*v*) pepsin (Fisher, AC417071000) dissolved in 0.01 N HCl for 20 min at room temperature, followed by two PBS washes. After blocking overnight at 4 °C in the blocking solution (PBS containing 0.1% Triton X-100 and 10% rabbit serum or 3% goat serum depending on the source of the secondary antibody), the sections were treated overnight with primary antibodies against: Iba1 (goat IgG) (C20) (sc28530) (1:50), STAT3 (rabbit IgG) (sc-7179) (1:100), P-Tyr^705^-STAT3 (goat IgG) (sc-7993) (1:100), NKp46 (rabbit IgG) (sc-292,796) (1:100), IL12p40 (rabbit IgG) (sc-7926) (1:100), IL10 (goat IgG) (sc-1783) (1:100), anti-STAT1 (rabbit IgG) (sc-592) (1:100), anti-P-Tyr^701^-STAT1 (mouse IgG) (sc-8394) (1:100), anti-iNOS (rabbit IgG) (NOS2 sc-651) (1:100), anti-ARG1 (rabbit IgG) (sc-20,150) (1:100), anti-CD133 (goat IgG) (sc-19,365) (1:50), anti-Sox-2 (mouse IgG) (sc-365,823) (1:50), anti-RM0029-11H3 (macrophage marker) (rat IgG) (sc101447) (1:50), and MCP-1 (Rabbit IgG) (Fisher #PA1–22488) (1:200). All antibodies were diluted in PBS containing 2% goat serum or 2% rabbit serum in 0.1% Triton X-100 (GRT-PBS). Samples which eventually received only 2° Ab (‘2° Ab controls’, negative controls) were kept overnight at 4 °C in the blocking solution. After washing three times with PBS, the respective secondary antibodies (Alexa Fluor 488 goat anti-rabbit (green), Alexa Fluor 568 goat anti-rabbit (red), Alexa Fluor 633 rabbit anti-goat (far red, pseudocolored purple), Alexa Fluor 633 goat anti-rabbit, Alexa Fluor 633 goat anti-mouse, Alexa Fluor 568 rabbit anti-goat, Alexa Fluor 568 goat anti-mouse) (Invitrogen), and goat anti-rat-phycoerythrin (red) (sc3740) (1:1000 dilutions in GRT-PBS) were added to wells containing the respective primary antibodies as well as to the 2° Ab controls. Following overnight incubation at 4 °C and three washes with PBS, the sections were treated with HOECHST33342 (10 μg/ml) for 30 min at room temperature, washed three times with PBS, and mounted on microscope slides with Prolong Gold anti-fade mounting fluid (Invitrogen, Catalog# P36930). Confocal Imaging was conducted using a Leica SP2 microscope from multiple randomly chosen fields encompassing regions in and around the tumor (for all the samples of all the groups from the short-term study and the ‘Vehicle’ samples of the long-term rescue study), and scar-tissue region (for the ‘CCP treated and rescued’ samples of the long-term rescue study) [8]. ImageJ was used to quantify the fluorescence intensities and the intensity for each marker was normalized to HOECHST33342 intensity (blue). Since STAT3 and STAT1 displayed both induction as well as phosphorylation-mediated activation, the HOECHST-normalized staining intensities were expressed both as P-STAT3/STAT3 and P-STAT1/STAT1 as well as P-STAT3/HOECHST and P-STAT1/HOECHST. Wherever necessary, co-stained cells (single, double or triple-stained) were counted and analyzed using ImageJ and the data was expressed as percentage of total count for the Vehicle group.

### Flow cytometry of immunostained brain tumor cells

For the 5-day study, the brains of the GL261 implanted, GBM mice from the four groups (Vehicle, CCP, CCP + NK1.1, and CCP + IL12Ab) were extricated (after anesthesia) on day 17 (from implantation) and without fixing and one-half of each GBM brain was rinsed twice with PBS. Next, only the cells from the tumor area (and surrounding tissue) from these unfixed brains were dissociated by mild trypsinization and single-cell suspensions were generated and fixed (in PFA) according to our earlier reports [8, 10]. Around 2 million fixed cells from each animal were used for immunostaining as described earlier for flow cytometry [8, 10, 27]. After each antibody treatment, the cells were pelleted and resuspended in washing buffers. Antibodies against Iba1 (C20) (1:50), iNOS (rabbit IgG) (NOS2 sc-651) (1:100), ARG1 (rabbit IgG) (sc-20,150) (1:100), NKp46 (1:100), IL12p40 (1:50), IL10 (1:50), CD68 (H-255) (Rabbit IgG) (sc-9139) (1:50), Active-Caspase3 (Asp175) (Rabbit IgG) (CST #9661) (1:100), and CD133 (1:50) were used for staining. Cells treated with the secondary antibody alone were used to set the threshold.

Flow cytometry was performed as reported earlier using cells from the high forward- versus side-scatter region (Additional file 1: Figure S1C) [8, 10, 27]. The double-stained fluorescent events from CD68(+)/Iba1(+), ARG1+/Iba1(+), iNOS+/Iba1(+), IL10+/Iba1(+), IL12+/Iba1(+), and CD68(+)/Active-Caspase3(+) cells appeared as sub-populations in the upper right (UR) quadrant within the coordinates 520 nm (green for CD68, ARG1, iNOS, IL10 and IL12) (FL1-A) and 580 nm (red for Iba1 and Active-Caspase3) (FL2-A). Single-stained fluorescent events from the scatter plots and from Nkp46(+) and CD133(+) cells appeared as sub-populations in the upper left (UL) quadrant within the coordinate 580 nm (red) and lower right (LR) quadrant within the coordinate 520 nm (green). Integrated fluorescence intensity was measured (for comparison between groups) by multiplying the number of positive events (single stained or double stained cells) by the mean fluorescence intensity.

*Integrated fluorescence:* The fluorescence profiles presented in the flow cytometry data are a combination of altered fluorescence intensity per cell and a change in the number of cells expressing a specific fluorophore [10, 27]. For example, the profiles in Fig. 4a(iv) and b(iv) indicated that a specific population of Iba1(+) TAM, which was also ARG1+, dramatically lost ARG1 fluorescence in the CCP-treated mice (a decrease in ARG1 intensity shown by a lateral shift in the ARG1 peak in Fig. 4a(iv)), while another population of possibly M_0_ cells was activated into an Iba1(+), iNOS^high^ state (an increase in iNOS fluorescence intensity per cell in Fig. 4b(iv)). In contrast, the fluorescence profile for IL10 in Iba1(+) TAM in Fig. 4c(iv) showed a less dramatic CCP-evoked lateral shift in IL10 fluorescence intensity per cell, but it was accompanied by a dramatic decrease in the IL10 peak area, which represents the number of events or cells. Therefore, for uniformity, we expressed our data as “integrated fluorescence” (fluorescence per cell x the total number of events (cells)) [10, 27].

### Statistical analysis

Two-tailed t-tests with unequal variance were used while comparing two groups and one-way ANOVA was used to compare among three groups. *p* ≤ 0.05 was considered as significant.

## Results

We have recently reported that the GBM tumors that kill the Vehicle-treated mice harbor tumor-promoting arginase1 (ARG1)^high^_,_ inducible nitric oxide synthase (iNOS)^low^ M2-like microglia/macrophages (TAM). In sharp contrast, the mice rescued from GBM by both antibody-linked CC and CCP contained ARG1^low^, iNOS^high^ M1-like TAM in the scar tissue [8]. In this report, we also demonstrated that CCP induces the transcription factor STAT1, which is known to trigger iNOS and IL12 synthesis in the TAM [27, 43]. Here, we investigate the possibility of NK cell recruitment and its role in this CCP-evoked M2➔M1 repolarization of the TAM and the cytokine balance occurring in the GBM tumor microenvironment [27, 44, 45].

### Sharp inhibition of TAM-associated STAT3 and IL10 and induction of IL12 observed in the scar tissue sections isolated from the CCP-treated and rescued mice

The transcription factor STAT3 is known to cause IL10-mediated suppression of both STAT1 as well as STAT1-mediated induction of the anti-tumor cytokine IL12 [8, 27, 46]. Our IHC analysis demonstrated that the GBM tumor in the Vehicle-treated mice at death harbored high levels of activated STAT3 (P-Y^705^-STAT3 or P-STAT3) (Additional file 1: Figure S1A and B), but the scar tissue sections from the CCP-treated and rescued mice displayed a 98% suppression of P-STAT3 (Additional file 1: Figure S1A and B) in the Iba1(+) (activated) TAM. The overall suppression of P-STAT3 was due to a combination of suppressed STAT3 expression (Additional file 1: Figure S1C) and inhibited STAT3 phosphorylation (Additional file 1: Figure S1D).

Based on earlier studies [27, 46], we expected that CCP-evoked inhibition of STAT3 in the TAM would prompt suppression of IL10 and induction of IL12, and IHC analysis yielded the expected results (Additional file 2: Figure S2A-C) [8].

### CCP treatment-associated recruitment of activated NK cells into the GBM scar tissue

**Fig. 1:**
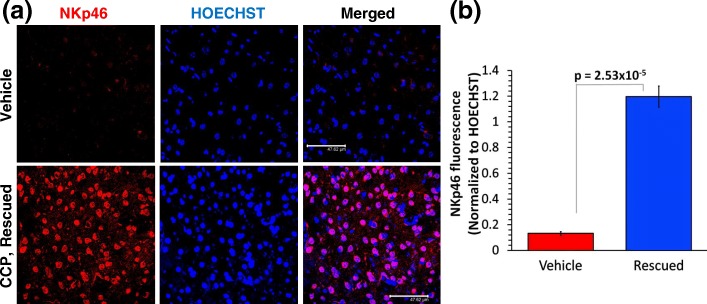
The scar tissue in the CCP-treated and rescued mice harbors activated natural killer (NK) cells: possible CCP-induced recruitment of activated NK cells. Brain sections harboring the tumor (Vehicle-treated) and Scar tissue (CCP-treated and rescued) were analyzed by IHC using the activated NK cell-specific NKp46 antibody. **a** and **b** The Vehicle-treated tumor tissue display very little NKp46 stain, the scar tissue sections derived from the CCP-treated and rescued mice show an 800% increase in NKp46-staining (mean ± S.D.). Three sections per mouse were used for IHC and the data (mean ± S.D.) obtained from Vehicle-treated (*n* = 4) and CCP-treated and rescued (*n* = 4) (Scale bar: 47.62 μm)

On measuring the expression of NKp46 that marks activated NK cells, we observed that the GBM tumor in the Vehicle-treated mice harbored very few activated NK cells, but the scar tissue region of CCP-treated and rescued mice displayed a large number of NK cells and an 800% increase in NKp46 fluorescence compared to the Vehicle-treated group (Fig. 1a and b) [10, 27].

We next studied the role of the NK cells in the observed CC-evoked M2➔M1 repolarization of TAM. To achieve this, we used a short-term regimen of CCP treatment, which was insufficient to cause complete elimination of the GBM. Earlier studies have shown that the NK cell surface antigen NK1.1 (PK136) is a highly selective NK-cell marker in C57BL/6 mice (the mouse strain used in this study) [47–49]. So, one set of randomly chosen GBM mice scheduled for CCP treatment was first injected with the NK1.1 (PK136) antibody (NK1.1Ab) to eliminate the peripheral NK cells as reported earlier [29, 50].

**Fig. 2:**
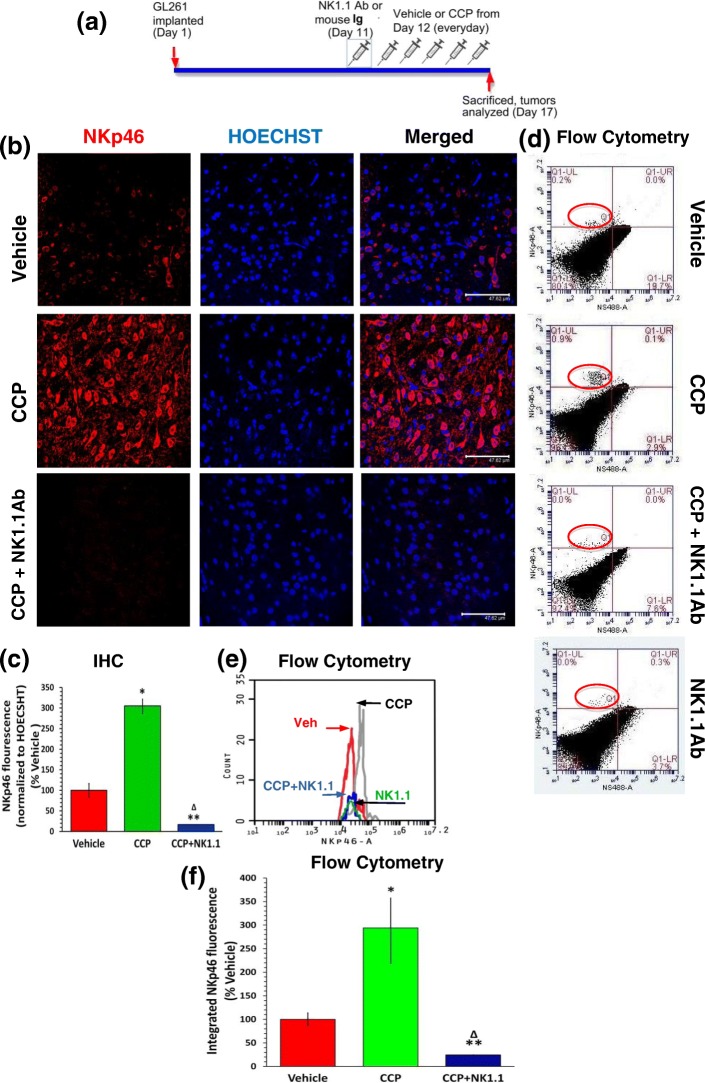
CCP treatment is associated with intra-tumor recruitment of activated NK cells, which is eliminated in mice peripherally injected with the NK cell-neutralizing NK1.1 antibody (NK1.1Ab). **a** After intracranial implantation of 10^5^ GL261 cells on day 1, the GBM-mice (*n* = 17) were randomly divided into four groups: ‘Vehicle’ (*n* = 5), ‘CCP’ (*n* = 5), ‘CCP + NK1.1’ (*n* = 4), and ‘NK1.1’ (*n* = 3). On day 11, the CCP + NK1.1 and NK1.1 groups received the NK1.1Ab (100 μg/mouse), whereas the other two groups received mouse IgG (as a negative control) (100 μg/mouse) (i.p.).On day 12, each mouse received either CCP (2 mg/ mouse/day in 200 μl PBS) for the ‘CCP’ and ‘CCP + NK1.1’ groups or PBS for the ‘Vehicle’ and ‘NK1.1’ groups for five days. On day 17, all mice were sacrificed and the GBM tumor in each mouse was cut into two parts and processed for IHC (fixed and sectioned) and flow cytometry (dispersed by trypsinization and fixed), respectively. **b** and **c** IHC: Four randomly chosen GBM-containing brain sections per mouse from each group were stained with the NKp46 antibody. The Vehicle-treated mice displayed very weak and sparse NKp46 staining (**b** top row and **c**), but the CCP-treated showed a 200% increase in NKp46 fluorescence (**b** middle row and **c**). This CCP-evoked NKp46 staining was virtually eliminated in the NK1.1Ab-treated mice (**b** bottom row and **c**) (*p = 1.2 × 10^− 8^ CCP versus Vehicle-treated; ∆ *p* = 7.3 × 10^− 9^ CCP versus CCP + NK1.1; **p = 1.0 × 10^− 3^ CCP + NK1.1 versus Vehicle-treated) (Scale bar: 47.62 μm). **d**-**f** Flow cytometry: CCP-treated samples displayed a 190% increase in integrated NKp46 fluorescence (IF) (*p = 0.02, CCP versus Vehicle). The NKp46 IF was virtually eliminated in the CCP + NK1.1 samples (**f**, ∆ *p* = 0.04, CCP + NK1.1 versus CCP; **p = 5.0 × 10^− 3^, CCP + NK1.1 versus Vehicle-treated). Data (mean ± S.D.) obtained from Vehicle (*n* = 4), CCP (*n* = 4), and CCP + NK1.1 (*n* = 4). Integrated fluorescence (IF) = mean fluorescence per cell X total number of cells (events) in a segregated population. **d**, **e** The NKp46(+) events (NK cells) were virtually eliminated in the NK1.1 samples

The CCP treatment was conducted from the following day on one of two mouse immunoglobulin (MIg)-treated sets (CCP group) and the NK1.1Ab-treated set (CCP + NK1.1Ab group) for five days. The second MIg-treated set received PBS (Vehicle group) for five days. This was followed by sacrifice and analysis of the GBM tissue (Additional file 3: Figure S3B and Fig. 2a).

### GL261 implantation on day 1 results in a large brain tumor on day 17, which harbors CD68^high^, Iba1(−) tumor cells and CD68^low^, Iba1(+) TAM

Earlier clinical studies of patient-derived GBM samples showed that high CD68 expression was a prognostic marker for GBM [51]. Our previous studies have also established that GBM cells express high levels of CD68 both in vitro and in vivo but are Iba1(−) [8, 10, 18], whereas the microglia express much lower levels of CD68 [18]. In these studies, murine and human GBM cell lines and xenograft explants showed high CD68 expression in vitro [8, 10, 18]. Additionally, the entire tumor core area of the Vehicle-treated GL261-implanted GBM mice showed pronounced expression of CD68 (CD68^high^/Iba1(−) GBM tumor cells), whereas the scar tissue area of the CCP-treated and rescued mice was devoid of the CD68^high^ (GBM tumor cells) population. Moreover, staining of the GBM brains with the dylight800-CD68Ab adduct clearly labeled the tumors [8, 10, 18]. As mentioned earlier, intra-GBM TAM express relatively low levels of CD68, while displaying high levels of Iba1 (CD68^low^/Iba1(+) TAM) [8, 10]. This differentially-expressed CD68 molecule has been exploited by our group as a targeting marker for GBM therapy to cause rescue of a significant number of mice with established tumors [8, 18].

Implantation of 10^5^ GL261 cells on day 1 resulted in tumors in all the mice of all the groups on day 17, esp. large tumors in the Vehicle-treated mice (Additional file 3: Figure S3A) [18]. Following dissociation of these tumor cells by mild trypsinization and staining for CD68 and Iba1, the cells were analyzed by flow cytometry in the high forward- and side-scatter range (Additional file 3: Figure S3C) [8, 10, 27]. Two clearly divided populations of CD68(+) cells were identified (Additional file 3: Figure S3D). The larger population of presumably GBM cells was Iba1(−) and CD68^high^, whereas the Iba1(+) but CD68^low^ cells were expected to be the TAM (Additional file 3: Figure S3D-F) [18]. The integrated CD68 fluorescence (IF) (mean fluorescence per cell x total number of cells in the population) in the CD68^high^ population was 780% of that in the CD68^low^ population (a 680% increase) (Additional file 3: Figure S3E and F).

### CCP treatment triggers NK cell recruitment into the GBM tumor and NK1.1Ab pre-treatment abrogates it

Five days of treatment of the GBM mice with Vehicle (PBS with MIg pretreatment), or CCP with either MIg pretreatment (CCP group) or NK1.1Ab pre-treatment (CCP + NK1.1 group) (Fig. 2a) caused a 200% increase in activated (NKp46(+)) NK cells in the GBM tumor of the CCP- but not CCP + NK1.1Ab-treated mice (Fig. 2b and c). Virtually no NKp46(+) cells were detected in the NK1.1Ab-pretreated mice (Fig. 2b, third row and c). Parallel flow cytometry analysis confirmed that CCP-treatment elicits a dramatic (190%) increase in NKp46 IF, demonstrating infiltration of activated NK cells into the GBM tumor. This CCP-evoked NK infiltration was virtually eliminated the NK1.1Ab-treated mice (Fig. 2d-f). Due to the lack of NKp46(+) NK cells in the ‘Vehicle’ group and since it is known that the blood-brain barrier (BBB) impedes the intra-brain entry of large molecules like antibodies, it is most likely that the peripheral infusion of the NK1.1Ab depleted only peripheral NK cells in the ‘CCP + NK1.1’ group to abrogate CCP-induced intra-GBM recruitment of activated NK cells [52].

As a negative control for CCP and proof of NK1.1-mediated NK cell neutralization, the flow cytometry profile for NKp46 staining of the NK1.1 group showed negligible quantities of intra-GBM NK cells (green line), which was almost identical to the NKp46 profile of the CCP + NK1.1 mice (blue line) (Fig. 2e). This NK cell depletion further demonstrated the high specificity of the peripherally administered NK1.1Ab toward the NKp46(+) NK cells.

### Intra-GBM recruitment of NK cells is eliminated upon neutralization of peripheral IL12 signaling using an IL12 antibody

Similar to the complete blockage of intra-GBM recruitment of NK cells in mice peripherally injected with the NK1.1 antibody (Fig. 2), blocking peripheral IL12 signaling by injecting GBM mice with an IL12 antibody virtually eliminated NK cell recruitment into the GBM tumor (Additional file 4: Figure S4A-C) [27, 29]. This confirmed that peripheral IL12-mediated signaling played a major role in the recruitment of peripheral NK cells into the GBM tumor.

### CCP treatment causes NK cell-independent recruitment of activated macrophages into GBM

**Fig. 3:**
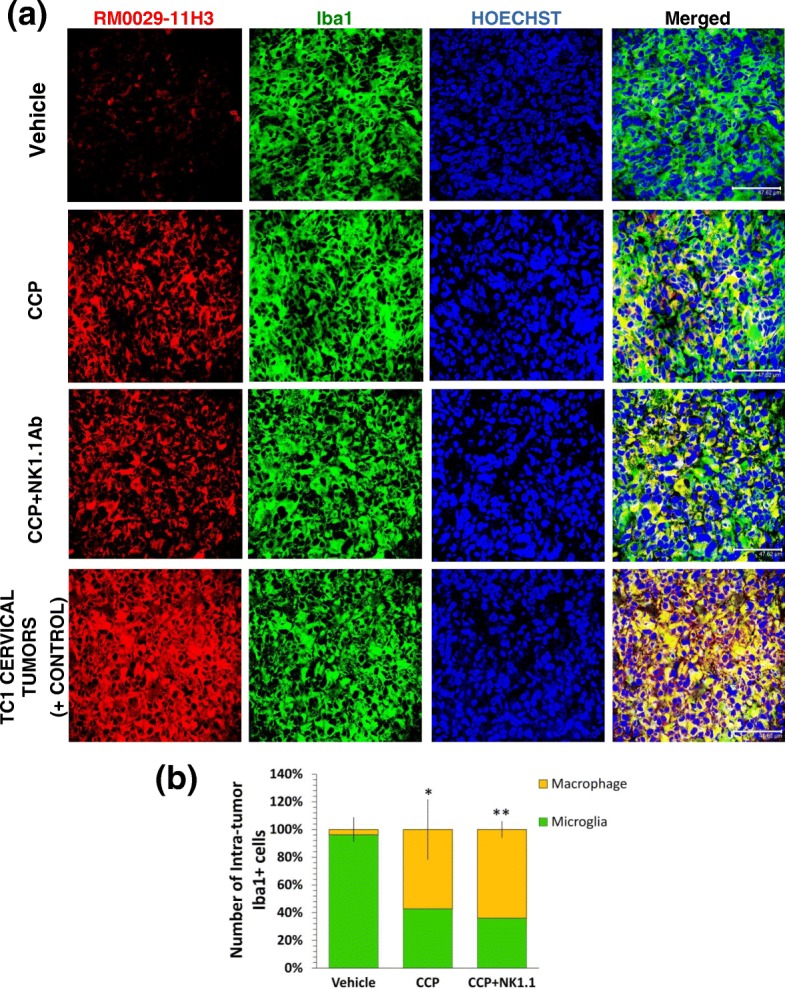
CCP treatment triggers intra-tumor recruitment of peripheral macrophages equally in both CCP and CCP + NK1.1 groups. GBM Brain sections parallel to those used for IHC in Fig. 2 were used to assess the possibility of intra-tumor recruitment of peripheral macrophages (macrophage specific marker RM0029-11H3(+)). **a** The GBM sections from the Vehicle-treated mice harbored mostly tumor-associated microglia (Iba1(+), RM0029-11H3(−), green) and a few macrophages (Iba1(+), RM0029-11H3(+), yellow) (first row), whereas the CCP (second row) and CCP + NK1.1-treated (third row) mice showed abundant tumor-associated macrophages (Iba1(+), RM0029-11H3(+)). Double-staining (Iba1(+), RM0029-11H3(+)) of peripheral tumors (HPV(+) TC1 tumors) (fourth row) confirmed that the tumor-associated cells observed in the GBM sections were macrophages from the periphery. **b** CCP-treatment triggered a 53% increase in the number of recruited intra-tumor macrophages (yellow)(*p = 7.8 × 10^− 4^ Vehicle versus CCP). The CCP + NK1.1 group also showed a 60% increase in the number of recruited intra-GBM macrophages (**p = 3 × 10^− 3^ Vehicle versus CCP + NK1.1). Three sections per mouse were used for IHC and the data (mean ± S.D.) obtained from Vehicle-treated (*n* = 4), CCP-treated (*n* = 4), and CCP + NK1.1-treated mice (*n* = 4). (Scale bar: 47.62 μm)

In addition to NK cell influx, we observed recruitment of activated macrophages into the GBM tumor equally in both CCP-treated and CCP + NK1.1Ab-treated mice, which ruled out any role of activated NK cells in the intra-GBM recruitment of macrophages (Fig. 3). The activated macrophages were identified by selective staining with the RM0028-11H3 antibody [18, 53]. Iba1 staining, however, marked both activated microglia and macrophages [8, 18, 27].

### CCP treatment causes a dramatic suppression of STAT3 in TAM, which is partially reversed in the NK1.1Ab pre-treated mice

We next tested the involvement of the NK cells in CCP-evoked inhibition of STAT3 expression and activation in the TAM. Whereas mice receiving five days of Vehicle treatment showed high levels of P-STAT3 as well as STAT3 in the Iba1(+) TAM, CCP treatment elicited an overall 88.5% decrease in P-STAT3, and this suppression was only by 61% in mice pretreated with the NK1.1Ab (Additional file 5: Figure S5A and B). Thus it is likely that the difference (88.5–61% = 27.5%) in P-STAT3 inhibition was due to the recruited NK cells. The CCP-evoked 88.5% suppression of P-STAT3 was the combined result of an inhibition of STAT3 (expression) (79%) and suppression of P-STAT3 (activation) (69%) in the Iba1(+) TAM (Additional file 5: Figure S5A-D). Both STAT3 as well P-STAT3 levels were partially restored in the Iba1(+) TAM in mice receiving CCP + NK1.1 (Additional file 5: Figure S5A-D).

### CCP treatment prompts a dramatic increase in P-STAT1, which is partially reversed in the NK1.1Ab pre-treated mice

The Vehicle-treated mice showed low levels of STAT1 and very little P-STAT1 whereas the CCP-treated mice displayed a 1286% increase in P-STAT1 in the Iba1(+) TAM. In contrast, the NK1.1Ab + CCP-treated mice showed only a 300% increase in P-STAT1 (Additional file 6: Figure S6A and B). Thus, the difference in P-STAT1 induction (1286–300% = 986%) was likely due to the recruited NK cells. The overall CCP-evoked increase in P-STAT1 was due to a combination of STAT1 induction (Additional file 6: Figure S6C) and STAT1 activation (P-STAT1 normalized to STAT1) (Additional file 6: Figure S6D) and both of these effects were partially reverted in the CCP + NK1.1Ab group.

### Pretreatment with the NK1.1Ab partially reverses CCP-evoked M2➔M1 repolarization, IL10 suppression, and IL12 induction

**Fig. 4:**
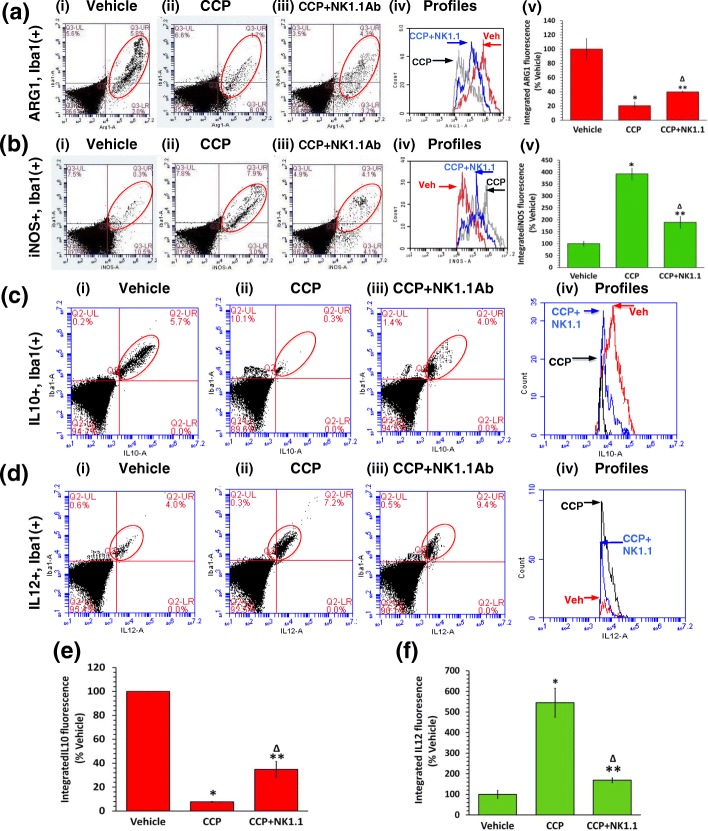
Peripheral injection of the NK cell-neutralizing NK1.1 antibody partially reverses the CCP-mediated M2➔M1 repolarization, IL10 suppression, and IL12 induction in TAM. **a** The dispersed Iba1(+) cells in the CCP-treated GBM displayed an 80% decrease in integrated ARG1 fluorescence with respect to the Vehicle-treated (*p = 0.01, Vehicle versus CCP) and this decrease was only 60% in the CCP + NK1.1 samples (Δ *p* = 0.03, CCP versus CCP + NK1.1; **p = 0.02, Vehicle versus CCP + NK1.1) (**a i**-**v**). Compared to the iNOS IF values in the Vehicle-treated, the Iba1(+) cells in the other two groups displayed a 293% increase in the CCP-treated (*p = 0.004, CCP versus Vehicle) and an 89% increase in the CCP + NK1.1-treated (Δ *p* = 0.014 CCP + NK1.1 versus CCP; **p = 0.04, Vehicle versus CCP + NK1.1) (**b i-v**). The graphs represent mean ± S.D. for iNOS/ARG1 obtained from Vehicle (*n* = 4), CCP (n = 4), and CCP + NK1.1 (*n* = 3) mice. **c**-**f** The Iba1(+) cells displayed a 92% decrease IL10 IF in the CCP-treated (*p = 8.7 × 10^− 6^, CCP versus Vehicle) and only a 65% decrease in the CCP + NK1.1-treated samples (Δ p = 0.03, CCP + NK1.1 versus CCP; **p = 5.0 × 10^− 3^, Vehicle versus CCP + NK1.1) (**c i** - **iv** and **e**). The Iba1(+) cells displayed a 445% increase in IL12 IF in the CCP-treated mice (*p = 0.01, CCP versus Vehicle) and a 69% increase in the CCP + NK1.1 mice (Δ *p* = 0.01, CCP + NK1.1 versus CCP; **p = 0.04, Vehicle versus CCP + NK1.1) (**d** **i**-**iv** and **f**). The graphs represent mean ± S.D. obtained from Vehicle (*n* = 4), CCP (*n* = 4), and CCP + NK1.1 (*n* = 3) mice

The CCP-evoked inhibition of STAT3 and induction of STAT1 in the Iba1(+) TAM (Additional file 5: Figure S5 and Additional file 6: Figure S6) would be expected to have their respective downstream effects on the synthesis of the cytokines IL10 and IL12. As discussed earlier, the transcription factor STAT3 is known to induce both ARG1 and IL10, whereas STAT1 has been reported to boost iNOS and IL12 [29, 46]. Interestingly, the GL261 tumor model is characterized by differential levels of hypoxia, and high hypoxia in turn supports the M2-like TAM in the GBM mass [54–57]. Thus, it was imperative for us to ascertain and compare the overall magnitude of TAM repolarization to the M1 state following different treatments by using dispersed cells from the whole tumor mass as well as the tumor periphery (see methods for further details) [8, 10]. Thus, as shown by flow cytometry, the CCP-evoked suppression of activated STAT3 (P-STAT3) was associated with an 80% inhibition of ARG1 staining in the Iba1(+) TAM (Fig. 4a i, ii, v). Only a 60% decrease in ARG1 was observed in the CCP + NK1.1Ab group (Fig. 4a iii). Thus, the difference (80–60% = 20%) was likely due to the recruited NK cells. A concomitant 293% increase in iNOS was also observed in the Iba1(+) TAM (Fig. 4b i, ii, v). In contrast, the NK1.1Ab + CCP-treated mice showed only an 89% increase in iNOS (Fig. 4b iii and v). Thus, the difference (293–89% = 204%) was likely due to the recruited NK cells.

In addition to this repolarization, the integrated fluorescence measurements in flow cytometry showed high IL10 expression by the Iba1(+) TAM in the Vehicle-treated mice, which was suppressed by 92% in the CCP-treated mice (Fig. 4c i, ii, iv and e). In contrast the NK1.1Ab + CCP-treated mice showed only a 65% decrease in IL10 (Fig. 4c iii and e). Thus the difference (92–65% = 27%) was likely due to the recruited NK cells.

The high IL10 expression in the Iba1(+) TAM was accompanied by a very low level of IL12 expression in the Vehicle-treated group. However, the IL12 level was boosted by 445% in the CCP-treated mice (Fig. 4d i, ii, iv, and f). In sharp contrast, the NK1.1Ab + CCP-treated mice showed only a 69% increase in IL12 (Fig. 4d iii and f). Thus, the difference in IL12 induction (445–69% = 376%) was likely due the recruited NK cells.

To confirm the overall functional phenotype of the TAM and validate the flow cytometry data, we also measured the expression levels of ARG1 and iNOS in the Iba1(+) TAM in the three groups by performing IHC on GBM sections (parallel to the dispersed cells used in flow cytometry analysis, see methods) (Additional file 7: Figure S7). The IHC experiments reconfirmed the flow cytometry results by establishing the pivotal role of the intra-GBM recruited NK cells in the CCP-evoked repolarization of TAM.

Similarly, to determine the overall functional polarity of the TAM and to corroborate the flow cytometry data, we measured the expression levels of IL10 and IL12 in the Iba1(+) TAM in the three groups by performing IHC on GBM sections (parallel to the dispersed cells used in flow cytometry analysis, see methods). Results obtained reconfirmed the crucial function of the intra-GBM recruited NK cells in CCP-mediated suppression of IL10 and induction of IL12 in TAM (Additional file 8: Figure S8).

We employed IHC and flow cytometry because the M2 and M1 markers are also ubiquitous enzymes (iNOS and ARG1) and cytokines (IL10 and IL12) which can be expressed by other Iba1(−) cell types (non-TAM, including other immune cells) in the GBM tumor microenvironment. Differentiation of such cells from the TAM was possible through IHC and flow cytometry analyses and not by ELISA-based cytokine secretion assays or Western blotting [8, 10].

### NK1.1Ab pre-treatment partially reverses CCP-evoked elimination of CD133(+) and SOX2(+) GBM stem cells and caspase-3 activation in CD68^high^ GBM cells

**Fig. 5:**
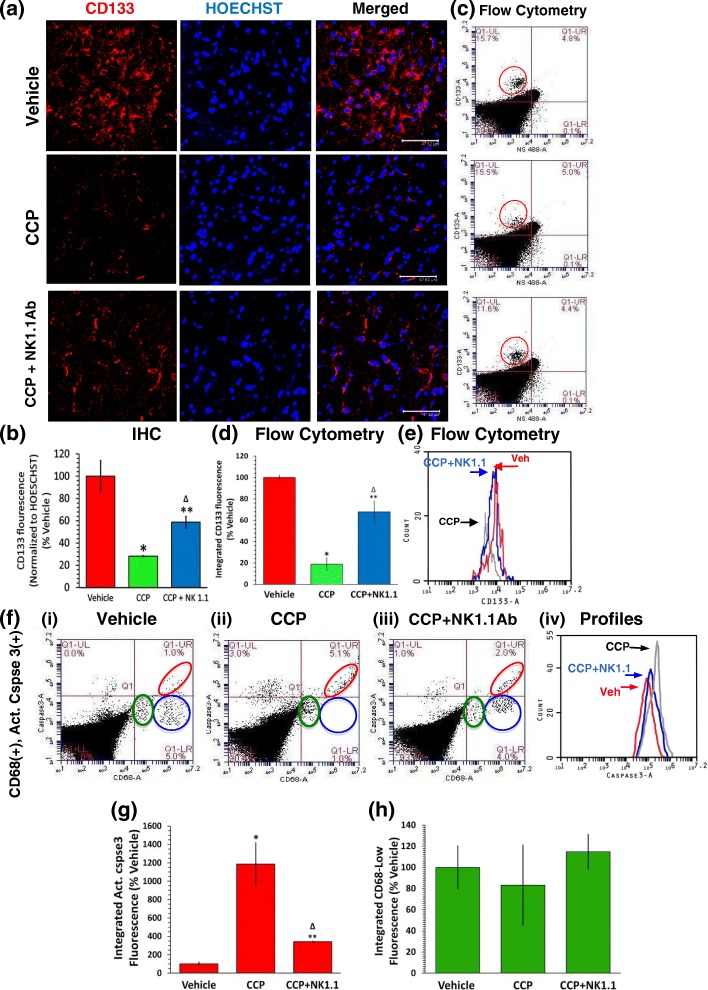
NK1.1Ab treatment partially reverses the CCP-evoked elimination of CD133(+) GBM stem cells and CD68^high^ GBM cells. The GBM tumor in each mouse was cut into two parts and processed for IHC and Flow Cytometry, respectively. **a**-**b** IHC: the Vehicle-treated mouse GBM sections harbored a significant number of CD133(+) GBM stem cells (red) (**a** first row). Compared to the Vehicle-treated, the CD133(+) fluorescence showed a 72% decrease in the CCP-treated (*p = 6.5 × 10^− 3^ CCP versus Vehicle) (**a**, second row and **b**) and a 41% decrease in the CCP + NK1.1-treated mice (**a** third row and **b**) (Δ *p* < 0.02, CCP + NK1.1 versus CCP; **p = 0.03, CCP + NK1.1 versus Vehicle). (Scale bar: 47.62 μm). **c**-**f** Flow Cytometry: compared to the Vehicle-treated, the CD133(+) cells (**c**, top, red circle) were suppressed by 81% in the CCP-treated (**c** middle and CD133 IF shown in **d**) (*p < 0.05, CCP versus Vehicle) and by 32% in the CCP + NK1.1 mice (**c**, bottom, and **d**) (Δ *p* < 0.03, CCP + NK1.1 versus CCP; **p = 3.0 × 10^− 3^, CCP + NK1.1 versus Vehicle). **e** The fluorescence profiles of the CD133(+) cells in the Vehicle, CCP, and CCP + NK1.1 samples. **f**-**h** The GBM cells were also probed for CD68, and active-caspase-3 (Act. Cspse 3). **f i** In the Vehicle-treated, two segregated populations of CD68(+) but Cspse3(−) cells were identified (Lower Right, LR). The larger population (shown within the blue circle) was CD68^high^ (presumably GBM cells) whereas a smaller population (shown within the green circle) was CD68^low^ (expected to be the TAM) [8, 10, 18]. A very small population of CD68(+), Act. Cspse3(+) (double positive) cells was identified in the upper right (UR) quadrant (within the red ellipse). **f ii** In the CCP-treated, CD68^high^ cells showed an 1187% increase in Act. Cspse 3 IF (red ellipse in the UR quadrant) with respect to Vehicle, along with the virtual disappearance of the CD68^high^ but Cspse3(−) cells in the blue circle in the LR quadrant (*p = 0.036, CCP versus Vehicle) (**f ii** and **g**). The Act. Cspse 3 IF increased by 343% in the CCP + NK1.1 samples, along with the reappearance of some CD68^hgh^ but Act. Cspse 3(−) cells in the blue circle in the LR quadrant (**f iii** and **g**) (Δ *p* < 0.04, CCP versus CCP + NK1.1; **p = 3.1 × 10^− 3^, CCP + NK1.1 versus Vehicle). (**f iv**) Fluorescence profiles for Act.Cspse 3. The CD68 fluorescence in CD68^low^ cells (TAM) was not significantly different in the three groups (**h**). The graphs represent mean ± S.D. obtained from mice treated with Vehicle (*n* = 4), CCP (*n* = 4), and CCP + NK1.1 (*n* = 3)

Since NK cells are known to eliminate both GBM cells as well as GBM stem cells [58, 59], we studied the contribution of the CCP-evoked intra-GBM recruited NK cells in elimination of GBM. IHC analysis of the GBM stem cell marker CD133 [10, 60] revealed a 72% decrease in CD133(+) cells in IHC in the CCP-treated mice. In contrast, NK1.1Ab + CCP-treated mice showed only a 41% suppression of CD133 staining (Fig. 5a, b). Corroboratively, flow cytometry demonstrated an 81% suppression of CD133(+) GBM stem cells in the CCP-treated, but only a 32% decrease in the NK1.1Ab + CCP-treated mice (Fig. 5c and d). Thus, the difference between the CCP and CCP + NK1.1 samples (81–32% = 49%) was likely due to the recruited NK cells.

The CD133(+) GBM stem cells are also known to be SOX2 positive [10, 37], and IHC analysis of parallel sections for SOX2 (Additional file 9: Figure S9) corroborated data presented in Fig. 5.

Even though the 5-day CCP treatment caused a decrease in tumor load in the GBM mice and this decrease was partially reversed in the NK-cell neutralized groups (CCP + NK1.1Ab and CCP + IL12Ab groups), these changes in tumor load were not statistically significant. It is known that GBM tumor harbors necrotic tissues and edema, which can still remain following therapy-triggered cell apoptosis, contributing to the apparent tumor size [61]. The complete clearance of these dead cells are likely to require much more than five days, as indicated by our CCP-mediated GBM mouse rescue studies where we observed the presence of a scar tissue area at the site of tumor implantation approximately 4 months after discontinuation of CCP treatment [8]. Thus, to verify the role of NK cells in 5-day CCP treatment-evoked apoptosis of GBM cells, we detected a population of CD68^high^ but activated caspase-3(−) (Act. Cspse3(−)) cells (in the blue circle) in the Vehicle-treated mouse tumors (Fig. 5fi and g). In sharp contrast, the tumors in the CCP-treated mice harbored mainly CD68^high^, Act. Cspse3(+) cells (in the red ellipse) with a very small number of CD68^high^ but Act. Cspse3(−) cells (in the blue circle) (Fig. 5fii and g) [10]. This CCP-evoked 1187% increase in Act. Cspse3(+), CD68^high^ cells was reduced to 343% in the NK1.1Ab pre-treated mice (Fig. 5fiii, iv, and g). By contrast, the CD68^low^, Act. Cspse3(−) cells (presumably the TAM) remained unaltered in all three groups, signifying that CCP-treatment doesn’t affect the viability of these cells (Fig. 5h). Thus, the difference in the increase in Act. Cspse3(+) cells between CCP and CCP + NK1.1Ab (1187–343% = 844%) was likely due to the recruited NK cells.

### In the CCP-treated mice, the magnitude of M1-type polarization phenotype of both macrophages and microglia in the GBM tumor is partially eliminated upon peripheral injection of the IL12 antibody

Elimination of NK cell recruitment through peripheral injection of NK1.1 antibody causes partial reversal of M2➔M1 repolarization (Fig. 4). Since data presented in Additional file 4: Figure S4 show that this NK cell recruitment into the GBM tumor is dependent on peripheral IL12 signaling, we expected that IL12Ab treatment would partially abrogate the magnitude of the M1-type polarization of both macrophages and microglia harbored within the GBM tumor. Corroboratively, the CCP-evoked induction in iNOS in the microglia (RM0029-H3(−), Iba1(+)) (474%) and the (RM0029-H3(+), Iba1(+)) macrophages (498%) was partly eliminated in the IL12Ab-treated mouse tumors (Additional file 8: Figure S8A, B). With respect to the Vehicle-treated, the CCP + IL12Ab-treated mice displayed a 242% increase in iNOS in the microglia and a 250% increase in iNOS in the macrophages (Additional file 10: Figure S10A, B). Therefore, the differences (474–242 = 232%) and (498–250% = 248%), respectively for microglia and macrophages were due to the stabilization of M1-polarization of the TAM by the recruited NK cells.

### CCP treatment causes a dramatic induction of monocyte chemotactic protein-1 (MCP-1) expression in the Iba1(+) TAM, which is not altered upon peripheral injection of IL12Ab

Earlier studies have established the chemokine MCP-1 as a marker of M1 macrophages and microglia [62, 63]. MCP-1 is also known to possess anti-tumor attributes and can compromise the BBB to translocate from the brain into the peripheral system to cause recruitment of immune cells such as M1-like macrophages and activated NK cells [31, 45, 64, 65]. CCP treatment in both absence as well as presence of peripheral IL12Ab injection caused a dramatic induction of TAM-associated MCP-1 in the GBM brain (Additional file 11: Figure S11). Therefore, this CCP-induced MCP-1 synthesis in the TAM was not dependent on the infiltration of activated NK cells into the GBM brain.

Based on this information, we postulate a likely mechanism of intra-tumor recruitment of M1-like macrophages and IL12-activated NK cells (Additional file 12: Figure S12) in which CCP-induced MCP-1 release from TAM initiates the process of macrophage activation, intra-tumor recruitment, NK cell activation by macrophage-released IL12, and MCP-1-evoked recruitment of activated NK cells into GBM.

## Discussion

Our earlier report [8] and this study demonstrate that CCP treatment affords relatively permanent repolarization of TAM from a tumor-promoting M2-like population to a tumoricidal M1-like milieu in the GBM. Since it is known that activated NK cells are recruited into peripheral tumors and that they also enter the brain under various conditions [29, 59, 66], we asked if CCP treatment triggers intra-GBM recruitment of NK cells and also if the NK cells influence CCP-evoked repolarization of TAM and elimination of GBM and GBM stem cells.

We noticed that the scar tissue from the CCP-treated and rescued mice but not the GBM tumor in the Vehicle-treated mice harbor a large number of activated NK cells (Fig. 2). These recruited NK cells were partially responsible for the CCP-evoked suppression of the M2-type markers, such as P-STAT3 (by 27.5%), IL10 (by 27%), and ARG1 (by 20%) in the TAM. Simultaneously, the NK cells were partially responsible for the CCP-evoked dramatic increase in the tumoricidal M1-type markers, such as P-STAT1 (by 986%) and IL12 (by 376%) in the TAM. The consequent IL12-evoked NK activation may amplify and prolong the M1 polarization of TAM in the GBM via IFNγ released by activated NK cells [27, 67], thereby causing a 204% increase in iNOS, which would catalyze the formation of cytotoxic NO inside the GBM (Additional file 2: Figure S2; Additional file 3: Figure S3, Additional file 4: Figure S4; Additional file 10: Figure S10 and Additional file 12: Figure S12) [66]. Finally, the recruited NK cells also account for 49% of the CCP-evoked suppression of CD133(+) GBM stem cells and 844% of the 1187% increase in active caspase-3 in the CD68^high^ GBM cells (Fig. 5).

In view of the earlier observation that the GBM stem cells trigger STAT3-mediated activation and proliferation of immunosuppressive M2 microglia/macrophages [68], anti-GBM-stem-cell and anti-STAT3 activities of CCP (Fig.5, Additional file 9: Figure S9) are likely to be pivotal in the observed CCP-evoked (i) correction of immunosuppression in the GBM microenvironment, (ii) M2**➔**M1 polarization of the tumor-associated microglia, and (iii) intra-tumor recruitment of M1-like macrophages and activated NK cells [10, 69]. Additionally, anti-GBM-stem-cell activities of TAM and NK cells are also likely to be central to the observed GBM elimination (Fig. 5) [10, 26, 59]. GBM stem cells are known cause tumor recurrence after conventional GBM therapies, so the permanent elimination of these cells is most likely to be crucial for the complete tumor remission of the CCP-treated GBM mice [8].

We observed NK-cell-independent CCP-triggered recruitment of M1-like macrophages into the GBM, such that the TAM were eventually composed of 50–60% of M1-macrophages (Fig. 3). This may partially account for the dramatic M2➔M1 switch in the TAM milieu. But what was the mechanism of intra-GBM recruitment of M1-macrophages and activated NK cells? It is known that MCP-1 (a.k.a. CCL2) is a chemokine marker of M1 microglia/macrophages [70, 71]. IHC analysis of brain sections from the 5-day CCP treatment revealed a peripheral-NK cell-independent, CCP-evoked induction of MCP-1 within the GBM TAM (Additional file 11: Figure S11). This anti-tumor chemokine MCP-1 is known to compromise the blood-brain barrier and exit into the blood stream. It is also known to bind to its receptor (CCR2) on M1-type macrophages and activated NK cells to recruit them to the source of its origin like the brain and tumor mass [31, 45, 64, 72, 73]. Based on such information, we hypothesize that the CCP-induced M1 microglia-derived chemokine MCP-1 exits the brain, triggers a cascade of events to first activate M1-type macrophages, which signal through IL12, activating the NK cells to express CCR2 and bind to MCP-1. M1 microglia-derived MCP-1 thus recruits both cell types (M1 macrophages and activated NK cells) into the GBM tumor (Additional file 12: Figure S12).

## Conclusion

Immunotherapy approaches using chimeric antigen receptor (CAR) T cell treatment and regulators of T-cell activation (checkpoint inhibitors) have recently come into prominence but such strategies are riddled with limitations and side effects [74, 75]. Although these cancer immunotherapy approaches which focus exclusively on T cells have yielded technically successful treatment protocols for most cancers, only a small percentage of cancer patients have been observed to respond to these treatments [76, 77]. Currently, these conventional cancer immunotherapeutic strategies face unique difficulties and challenges in the context of GBM therapy. Just to name a few, (i) GBM cells and GBM stem cells cause immunosuppression, (ii) the BBB prevents delivery of therapeutic agents into GBM, (iii) the brain harbors very few anti-tumor T cells, and (iv) the absence of a lymphatic system in the brain limits entry of immune cells into the CNS [78, 79]. Perhaps due to such reasons, recent times have seen a heightened interest among researchers to study the relatively untapped innate immune cells like macrophages and NK cells as potential targets for effective cancer immunotherapy [80]. Our findings take us a step further by exploring an alternative, safe strategy to recruit such cells into the brain to eliminate GBM. Thus, we provide here a highly effective immunotherapeutic approach to eliminate GBM tumors. Due to the fact that the peripheral cancers also involve recruitment of macrophages and NK cells, our strategy can eventually become an effective general approach for new-age cancer immunotherapy [10, 27]. CCP is therefore a safe yet promising agent that may combat GBM tumors in patients by recruiting the cells of the innate immune system.

## Additional files

Additional file 1: **Figure S1.** The activated (Iba1(+)) microglia/macrophages (TAM) in the CCP-treated and rescued mice display a dramatic inhibition of STAT3. Brain sections parallel to those used in our previous report [8] were used to assess the levels of STAT3 and P-Tyr^705^-STAT3 (activated) in the Iba1(+) TAM. (A upper and lower rows) and **(B)** The Vehicle-treated mice displayed a high level of activated (P-Y^705^-STAT3) TAM, which was suppressed by 98% in the scar tissue sections from CCP-treated and rescued mice. This overall suppression of P-STAT3 was a result of suppression of STAT3 expression (STAT3 normalized to HOECHST) **(C)** and STAT3 activation (P-STAT3 normalized to STAT3) **(D)**. Four sections per mouse were used for imaging and data (mean ± S.D.) were graphically presented as obtained from Vehicle-treated mice (*n* = 3), and CCP-treated and rescued mice (*n* = 3). HOECHST = HOECHST33342. (Scale bar: 47.62 μm). (DOC 7669 kb)

Additional file 2: **Figure S2.** GBM-associated, activated (Iba1(+)) TAM are IL10^high^ and IL12^low^, whereas the TAM in the scar tissue of CCP-treated and rescued mice are IL10^low^ and IL12^high^. Brain sections harboring the tumor (Vehicle-treated) and scar tissue (CCP-treated and rescued) were triple-stained with Iba1, IL10 and IL12 antibodies. The TAM in the GBM tissue in Vehicle-treated mice display high levels of IL10 and low levels of IL12 (**A**, upper row), whereas the scar tissue from the CCP-treated and rescued mice displayed IL10^low^ and IL12^high^ TAM (**A**, lower row, **B** and **C**). The graphs show a 91% decrease in IL10 and a 300% increase in IL12 (CCP IL12: 400% of Vehicle-treated) (mean ± S.D.) following CCP-treatment (n = 3 per group, with four randomly chosen brain sections from each mouse). (Scale bar: 47.62 μm). HOECHST = HOECHST33342. (DOC 1761 kb)

Additional file 3: **Figure S3.** GBM tumor generation by GL261 implantation on day 1, peripheral treatment with mouse immunoglobulin (mouse Ig or MIg) or the NK-cell-neutralizing NK1.1 antibody on day 11, and a regimen of Vehicle (PBS) or CCP treatment for five days yields tumors in all the groups on day 17. **(A)** As shown by near-IR scanning, implantation of 10^5^ GL261 cells on day 1 resulted in large tumors on day 17 in the mice from all the groups (see below). A representative Vehicle-treated tumor (yellow) is shown here. **(B)** On day 11, GBM-mice (*n* = 14) were randomly divided into three groups: ‘Vehicle’ (*n* = 5), ‘CCP’ (*n* = 5), and ‘CCP + NK1.1’ (*n* = 4). The mice in the CCP + NK1.1 group received intra-peritoneal (i.p.) infusion of the NK cell-neutralizing anti-NK1.1 antibody (100 μg/mouse). On day 12, each mouse received i.p. injections of sterile PBS (Vehicle group) or CCP (2 mg/ mouse/day) for the CCP and CCP + NK1.1 groups for five days. On day 17, all mice were sacrificed. **(C)** Forward- versus side-scatter plot showing the population of cells chosen for the analysis of fluorescence. **(D)** The day-17 GBM tumors from mice of all the groups were extricated, dissociated, cells fixed and then subjected to immunostaining for CD68 and Iba1 followed by flow cytometry analysis to confirm the presence of established GBM tumors [8, 10]. Two segregated populations of CD68(+) cells were identified [10]. The larger population (presumably GBM cells) were Iba1(−) and CD68^high^, whereas the Iba1(+) but CD68^low^ cells were expected to be the TAM [8, 10, 18]. **(E and F)** The Iba1(−)/CD68^high^ (GBM cells) in the GBM samples showed 680% higher integrated CD68 fluorescence (fluorescence per cell in arbitrary units X number of events) × 10^8^ relative to the Iba1(+)/CD68^low^ (TAM cells) (*p* = 1.7 × 10^− 4^). The graph represents mean ± S.D. obtained from the analysis of GBM mice (*n* = 6). (DOC 390 kb)

Additional file 4: **Figure S4.** CCP treatment causes intra-tumor recruitment of activated NK cells, which is eliminated in mice peripherally administered with an IL12 antibody. After intracranial implantation of 10^5^ GL261 cells on day 1, the GBM-mice (*n* = 9) were randomly divided into three groups: ‘Vehicle’ (*n* = 3), ‘CCP’ (*n* = 3), and ‘CCP + IL12Ab’ (*n* = 3). On day 11 and day 14, the CCP + IL12Ab group received NK cell-neutralizing anti-IL12p40 Ab (100 μg/mouse/day), whereas the other two groups received rat Ig (100 μg/mouse) (i.p.). From day 12, each mouse received CCP (2 mg/ mouse/day in 200 μl PBS) for the CCP and CCP + IL12Ab groups and sterile PBS for the “Vehicle” group for five days. On day 17, all mice were sacrificed and the dispersed cells were immunostained for flow cytometry analysis. CCP-treated samples display a 270% increase in integrated NKp46 fluorescence (370% of Vehicle-treated) (**A**, **B** and **C;** *p = 0.01, CCP versus Vehicle). The NKp46 fluorescence was virtually eliminated in the GBM samples from the CCP + IL12Ab mice (**A**, **B** and **C; Δ** *p* = 4.4 × 10^− 4^, CCP + IL12Ab versus CCP; **p = 2.7 × 10^− 3^, CCP + IL12Ab versus Vehicle-treated). The graphs represent data (mean ± S.D.) obtained from Vehicle (*n* = 3), CCP (*n* = 3), and CCP + IL12Ab (*n* = 3). Integrated fluorescence = average fluorescence per cell X total number of cells (events) in a segregated population. (DOC 1976 kb)

Additional file 5: **Figure S5.** Peripheral neutralization of NK cells by pre-injecting with the NK1.1 Ab partially reverses the CCP-mediated suppression of STAT3 in the TAM. GBM Brain sections parallel to those used in Fig. 3 from the three groups (Vehicle, CCP and CCP + NK1.1Ab) were used to assess the levels of STAT3 and activated STAT3 (P-Y^705^-STAT3) (P-STAT3) in the Iba1(+) TAM. **(A)** The GBM sections from the Vehicle-treated mice displayed high levels of STAT3 and P-STAT3 (top row), whereas the CCP-treated mice showed an 88.5% decrease in P-STAT3 (normalized to HOECHST) (*p = 5.1 × 10^− 5^, CCP-treated versus Vehicle) and this CCP-evoked suppression was only by 61% in samples obtained from the CCP + NK1.1 mice (Δ *p* = 5.9 × 10^− 3^, CCP + NK1.1 versus CCP-treated) **(B)**. The CCP-evoked 88.5% suppression of P-STAT3 in the TAM was the result of a 79% decrease in STAT3 (normalized to HOECHST) (only 68% in the CCP + NK1.1 mice) **(C)**, and a 68% decrease P-STAT3 (normalized to STAT3) (only 48% in CCP + NK1.1) (P-STAT3 normalized to STAT3) (**p = 1.2 × 10^− 4^ Vehicle versus CCP + NK1.1) **(D)**. Three sections per mouse were used for imaging and the graphs represent mean ± S.D. obtained from Vehicle (*n* = 4), CCP (*n* = 4), and CCP + NK1.1 (*n* = 4). (Scale bar: 47.62 μm). (DOC 8919 kb)

Additional file 6: **Figure S6.** Peripheral neutralization of NK cells partially reverses the CCP-mediated induction and activation of STAT1 in the TAM. GBM Brain sections parallel to those used in Fig. S5 from the three groups (Vehicle, CCP and CCP + NK1.1Ab) were used to evaluate the levels of STAT1 and activated STAT1 (Tyr^701^-STAT1) (P-STAT1) in the Iba1(+) TAM. **(A)** The Vehicle-treated mice showed low levels of STAT1 (red) and P-STAT1 (purple) in the Iba1(+) (green) cells (First row and **B**), but a 1286% overall increase in P-STAT1 was observed in the CCP-treated GBM sections (Second row and **B**) (*p = 2.3 × 10^− 4^, Vehicle versus CCP). This CCP-evoked increase in P-STAT1 was only 300% in the CCP + NK1.1 mouse samples (Third row and **B**) (Δ *p* = 0.04, CCP versus CCP + NK1.1). The CCP-evoked increase in P-STAT1 was the result of a 300% induction in STAT1 (only 194% increase in the CCP + NK1.1 sections) **(A, C),** and a 423% augmentation of P-STAT1 with respect to STAT1 (activation) (only 206% activation in the CCP + NK1.1 sections) (_**_p = 2.9 × 10^− 4^, Vehicle versus CCP + NK1.1) **(A, D).** Three sections per mouse were used for imaging and the graphs represents mean ± S.D. obtained from mice treated with Vehicle (*n* = 4), CCP (*n* = 4), and CCP + NK1.1 (*n* = 3). (Scale bar: 47.62 μm). (DOC 113 kb)

Additional file 7: **Figure S7.** Peripheral neutralization of NK cells partially reverses the CCP-induced M2➔M1 repolarization of TAM within the GBM mass. To verify the data presented in Fig. 4, brain sections parallel to those used in Fig. S6, harboring the GBM tumor from the three groups of mice (Vehicle, CCP and CCP + NK1.1Ab) were triple-stained with Iba1 (green), iNOS (red) and ARG1 (purple) antibodies. **(A)** The Vehicle-treated GBM sections displayed weak iNOS staining but strong ARG1 staining in the Iba1(+) TAM (**A**, top row). In contrast, the CCP-treated mice presented a 58% decrease in ARG1 (*p = 2.5 × 10^− 6^ CCP versus Vehicle). This CCP-evoked suppression in ARG1 was only 35% in the CCP + NK1.1 sections (Δ *p* = 5.6 × 10^− 7^, CCP + NK1.1 versus CCP; **p = 3 × 10^− 4^, CCP + NK1.1 versus Vehicle) (**A**, middle row, and **B**). In contrast, the Iba1(+) TAM in the CCP-treated mice showed a 212% increase in iNOS (*p = 6.2 × 10^− 7^, CCP versus Vehicle) and this CCP-evoked increase in iNOS was only 147% in the CCP + NK1.1 group (Δ *p* = 1.1 × 10^− 4^, CCP + NK1.1 versus CCP; **p = 7.6 × 10^− 5^, CCP + NK1.1 versus Vehicle) (**A**, lowest row, and **C**). Four sections per mouse were used for imaging and the graphs represent mean ± S.D. obtained from Vehicle (*n* = 4), CCP (*n* = 4), and CCP + NK1.1 (*n* = 3). (Scale bar: 47.62 μm). (DOC 1300 kb)

Additional file 8: **Figure S8.** Abrogation of NK cells by peripheral infusion of NK1.1 antibody partially reverts the CCP-mediated suppression of IL10 and induction of IL12 in the TAM within the GBM mass. As a corroboration of the flow cytometry data presented in Fig. 4, GBM brain sections parallel to those used in Fig. S7, harboring the tumor from the three groups (Vehicle, CCP and CCP + NK1.1Ab) were triple-stained with antibodies against Iba1 (green), IL12 (red), and IL10 (purple). Sections from Vehicle-treated mice showed strong IL10 expression in the Iba1(+) TAM (**A** first row), which was suppressed by 83% in the CCP-treated mice (*p = 9.1 × 10^− 8^, CCP versus Vehicle) (**A**, second row and **B**), but this CCP-evoked suppression of IL10 was only 45% in the CCP + NK1.1 sections (Δ *p* = 8.3 × 10^− 8^, CCP + NK1.1 versus CCP; **p = 2.7 × 10^− 5^, CCP + NK1.1 versus Vehicle) (**A** third row and **B**). In contrast, IL12 expression in the Iba1(+) cells was very low in the sections from the Vehicle-treated mice (**A** first row), but it increased by 439% in the CCP-treated mice (*p = 1.3 × 10^− 10^, CCP versus Vehicle) (**A** second row and **C**), and this increase was only 277% in the CCP + NK1.1 mice (Δ *p* = 9.3 × 10^− 6^, CCP + NK1.1 versus CCP; **p = 4.6 × 10^− 7^, CCP + NK1.1 versus Vehicle) (**A** third row and **C**). Four sections per mouse from Vehicle (n = 4), CCP (n = 4), and CCP + NK1.1 (*n* = 3) mice were used for imaging and each graph represents mean ± S.D. (Scale bar: 47.62 μm). (DOC 4096 kb)

Additional file 9: **Figure S9.** Peripheral neutralization of NK cells partially reverses the CCP-mediated elimination of SOX2(+) GBM stem cells. **(A)** Multiple, randomly chosen GBM brain sections parallel to those used in Fig. S8, from the three groups (Vehicle, CCP and CCP + NK1.1Ab) were single-stained with an antibody against SOX2. The sections from the Vehicle-treated mice displayed significant number of SOX2(+) GBM stem cells (**A** first row). In contrast, the sections from CCP-treated mice showed a 79% suppression of SOX2(+) cells (*p = 8.6 × 10^− 3^, CCP versus Vehicle) (**A**, second row, and **B**). In contrast, the CCP-evoked suppression of SOX2(+) cells was only 54% in the sections from CCP + NK1.1 treated mice (Δ *p* = 0.015, CCP + NK1.1 versus CCP;**p = 0.025, CCP + NK1.1 versus Vehicle) (**A**, third row, and **B**). Three sections per mouse were used for imaging and the graphs represent mean ± S.D. obtained from Vehicle (*n *= 4), CCP (*n* = 4), and CCP + NK1.1 (*n* = 3). (Scale bar: 47.62 μm). (DOC 6174 kb)

Additional file 10: **Figure S10.** Peripheral pre-treatment with IL12 antibody partially eliminates the CCP-mediated M1-like phenotype of the TAM in GBM tumor. GBM Brain sections parallel to the dispersed cells used in Fig. S4 from the Vehicle, CCP and CCP + IL12Ab groups were used to assess and quantify the expression of iNOS on tumor associated microglia (Iba1(+)/RM0029-11H3(−)) and macrophages (Iba1(+)/RM0029-11H3(+)) upon CCP and CCP + IL12Ab treatment. **(A)** The GBM sections from the Vehicle-treated mice harbored mostly tumor-associated microglia and few macrophages (first row) which showed sparse iNOS staining. The CCP (second row) treatment showed copious presence of both iNOS+ intra-GBM recruited tumor-associated macrophages and resident tumor-associated microglia. The CCP + IL12Ab-treated (third row) mice showed intermediate levels of iNOS in the macrophages and microglia. **(B) (Left)** CCP-treatment caused a 474% increase in the intensity of microglia-associated iNOS (fluorescence intensity normalized to the number of cells) (*p = 8.8 × 10^− 6^ Vehicle versus CCP), while CCP + IL12Ab treatment reduced this augmentation to 242% with respect to the Vehicle (**p = 1.2 × 10^− 3^ Vehicle versus CCP + IL12Ab; ∆ *p* = 4.1 × 10^− 3^ CCP versus CCP + IL12Ab). **(Right)** CCP-treatment induced a 498% increase in macrophage-associated iNOS intensity (*p = 5.4 × 10^− 5^ Vehicle versus CCP), whereas the CCP + IL12Ab group showed a partial reversal of this increase to 250% with respect to the Vehicle (**p = 5.4 × 10^− 5^ Vehicle versus CCP + IL12Ab; ∆ *p* = 3.5 × 10^− 4^ CCP versus CCP + IL12Ab). Four sections per mouse were used for imaging and counting and the graphs represent mean ± S.D. obtained from Vehicle (*n* = 3), CCP (*n* = 3), and CCP + IL12Ab (*n* = 3). (Scale bar: 47.62 μm). (DOC 4392 kb)

Additional file 11: **Figure S11.** CCP treatment causes a dramatic induction of MCP-1 in the Iba1(+) TAM. Sections made from GBM brain tissues parallel to the dispersed cells used in Fig. S10, from the three groups (Vehicle, CCP and CCP + IL12Ab) were immunostained to assess the expression of MCP-1 on the Iba1(+) TAM. **(A)** The GBM sections from the Vehicle-treated mice harbored mostly tumor-associated microglia and few macrophages (first row) which expressed very little MCP-1, whereas the tumors from both the CCP (second row) and CCP + IL12Ab-treated (third row) mice showed both recruited tumor-associated macrophages and resident tumor-associated microglia, both strongly expressing MCP-1. **(B)** CCP-treatment triggered a 374% increase in the MCP-1 fluorescence in the microglia (*p = 2.6 × 10^− 5^ Vehicle versus CCP), whereas CCP + IL12Ab-treatment showed 323% increase in MCP-1 intensity (**p = 6.5 × 10^− 6^ Vehicle versus CCP + IL12Ab). No significant difference was observed between the CCP and CCP + IL12Ab groups. Four sections per mouse were used for imaging and counting and the graph represent mean ± S.D. (fluorescence intensity normalized to the number of cells) obtained from Vehicle (*n* = 3), CCP (*n* = 3), and CCP + IL12Ab (*n* = 3). (Scale bar: 47.62 μm). We have shown earlier that CCP-treatment of mice causes an induction of activated, p65 NF-kB in GBM TAM [8, 27]. Additionally, p65 NF-kB has been shown to induce MCP-1 expression [81], which is most likely the mechanism of CCP-mediated induction of MCP-1 in the GBM TAM in these mice. (DOC 8284 kb)

Additional file 12: **Figure S12.** Possible signaling pathways involved in intra-GBM recruitment of M1-type macrophages and activated NK cells upon CCP treatment. CCP initiates a complete cycle by causing inhibition of STAT3 in the tumor-associated microglia [82]. This releases STAT1 from the inhibitory effects of STAT3 [83]. Induced P-STAT1 triggers the synthesis of iNOS, IL12, thereby increasing M1-type microglia [62, 63]. MCP-1 released by M1-type microglia compromises the blood-brain barrier, exits into the blood, binds to its receptor (CCR2) on macrophages, polarize them to the M1-type state, and recruits them into GBM in the brain [45, 64, 65, 73, 84, 85]. Meanwhile, M1-type macrophages in blood elicit STAT1-mediated IL12 synthesis and release [43]. The released IL12 binds to IL12 receptor (IL12R) on the NK cells, thereby activating these cells and causing interferon-gamma (IFNγ) release [67]. The released IFNγ causes receptor-mediated inhibition of STAT3 in the macrophages [86–88], which in turn amplifies activated STAT1 and IL12 release [83]. Additionally, IFNγ also causes receptor-mediated activation of STAT1 [89, 90]. This stabilizes the M1 phenotype and the activation of NK cells. Concomitantly, GBM-associated M1-type microglia-released MCP-1 binds to CCR2 on the IL12-activated NK cells [70] and causes recruitment of these cells into the GBM [45]. Once in the GBM, the activated NK cells engage in receptor-mediated interactions with the GBM and GBM stem cells [58, 59], thereby killing GBM and GBM stem cells. Additionally, the activated NK cells also kill resting microglia, thus enriching the M1-type microglia in the TAM [66]. Simultaneously, the M1-type macrophages and microglia within the GBM elicit iNOS-mediated release of nitric oxide (NO) [29, 84], which eliminates GBM and GBM stem cells. (DOC 1280 kb)

## Abbreviations

2×SSC: 2× saline-sodium citrate buffer
anti-RM0029-11H3: Anti-Macrophage antibody
ARG1: Arginase1
BBB: Blood-brain barrier
CC: Curcumin
CCP + IL12Ab: IL12-Neutralizing Antibody Infusion Followed By Curcumin Phytosome Treatment
CCP + NK1.1Ab/ CCP + NK1.1: Natural Killer Cell-Neutralizing Antibody Infusion Followed By Curcumin Phytosome Treatment
CCP: Curcumin Phytosome
CCR2: Chemokine receptor type 2
CD: Cluster of Differentiation
CSI: College of Staten Island
CUNY: City University of New York
GBM: Glioblastoma
HCl: Hydrogen chloride
IACUC: Institutional Animal Care Committee
Iba1: Ionized Calcium-Binding Adapter Molecule 1
IF: Integrated Fluorescence
IFN-γ: Interferon-gamma
IgG: Immunoglobulin G
IHC: immunohistochemistry
IL10: Interleukin 10
IL12: Interleukin 12
IL12Ab: IL12-neutralizing antibody treatment
IN: Intranasal
iNOS: Inducible Nitric Oxide Synthase
LR: Lower right
MCP-1: Monocyte Chemotactic Protein-1 (also known as CCL2)
MIg: Mouse Immunoglobin G (mouse Ig)
NK cells: Natural Killer cells
NK1.1 Ab: Natural Killer cell-neutralizing antibody treatment
NO: Nitric oxide
PBS: Phosphate-buffered saline
PC: Phosphotidylcholine
PFA: Paraformaldehyde
P-Tyr701-STAT1: Phospho-STAT1 antbody
P-Tyr705-STAT3: Phospho-STAT3 antbody
S.D.: Standard deviation
STAT1/3: Signal Transducer and Activator of Transcription 1/3
TAM: Tumor-associated microglia/macrophages
TMZ: Temozolomide
UL: Upper left
