# Supplementary material for: Phytosomal curcumin causes natural killer cell-dependent repolarization of glioblastoma (GBM) tumor-associated microglia/macrophages and elimination of GBM and GBM stem cells
Source: J Exp Clin Cancer Res. 2018 Jul 25;37:168. doi: 10.1186/s13046-018-0792-5 (PMC6058381; doi:10.1186/s13046-018-0792-5)
Supplement: Supplementary file 1 — Figure S1. The activated (Iba1(+)) microglia/macrophages (TAM) in the CCP-treated and rescued mice display a dramatic inhibition of STAT3. Brain sections parallel to those used in our previous report [8] were used to assess the levels of STAT3 and P-Tyr705-STAT3 (activated) in the Iba1(+) TAM. (A upper and lower rows) and (B) The Vehicle-treated mice displayed a high level of activated (P-Y705-STAT3) TAM, which was suppressed by 98% in the scar tissue sections from CCP-treated and rescued mice. This overall suppression of P-STAT3 was a result of suppression of STAT3 expression (STAT3 normalized to HOECHST) (C) and STAT3 activation (P-STAT3 normalized to STAT3) (D). Four sections per mouse were used for imaging and data (mean ± S.D.) were graphically presented as obtained from Vehicle-treated mice (n = 3), and CCP-treated and rescued mice (n = 3). HOECHST = HOECHST33342. (Scale bar: 47.62 μm). (DOC 7669 kb) [file 13046_2018_792_MOESM1_ESM.doc]

| **(A)** | **P-STAT3** | **STAT3** | | **Iba1** | **HOECHST** | | **Merged** |
| --- | --- | --- | --- | --- | --- | --- | --- |
| **Vehicle** | **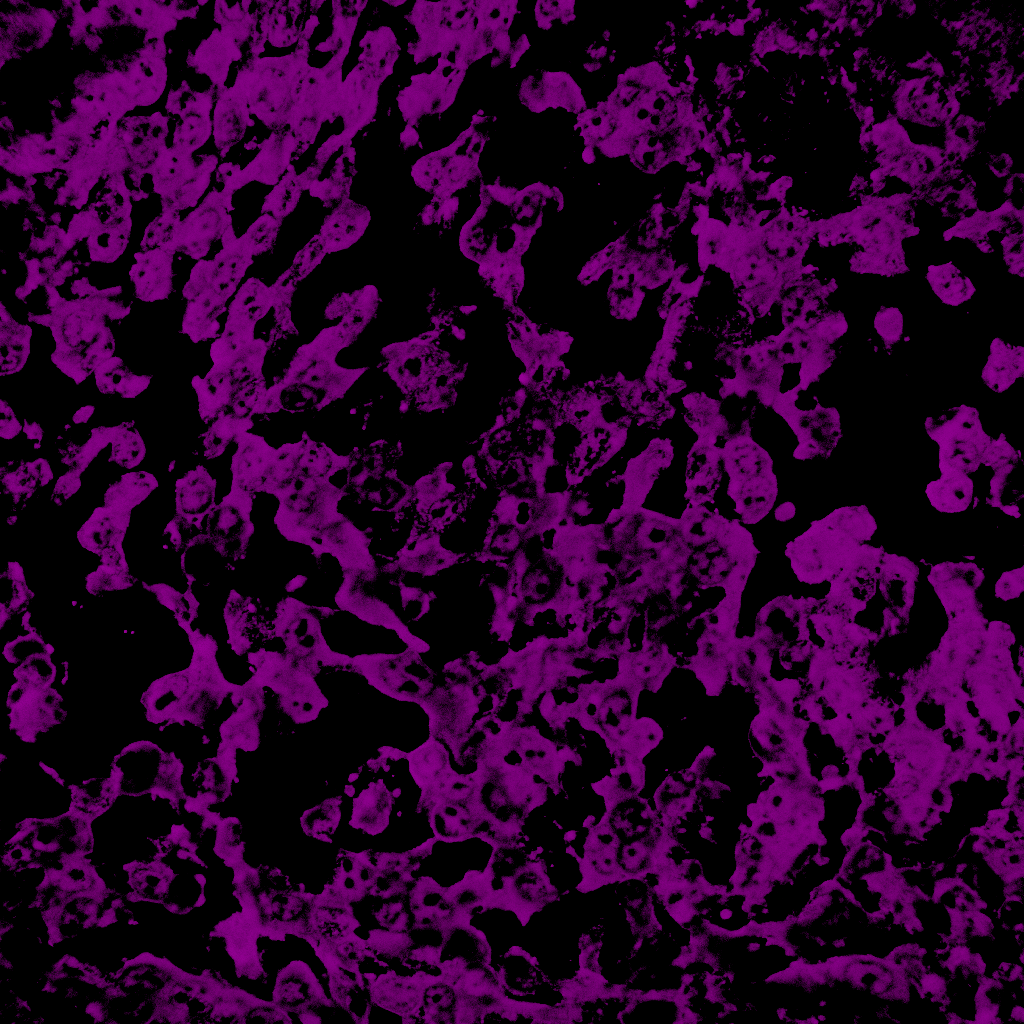** | **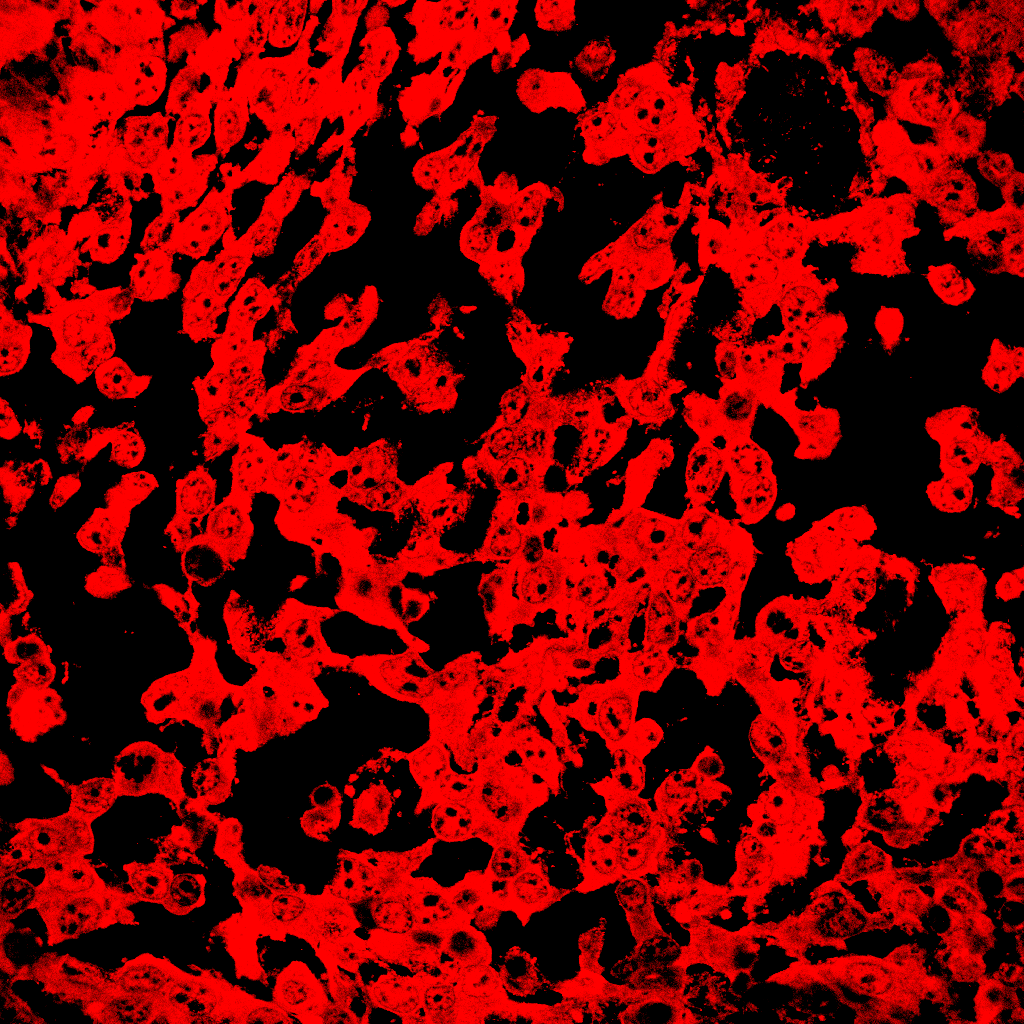** | | **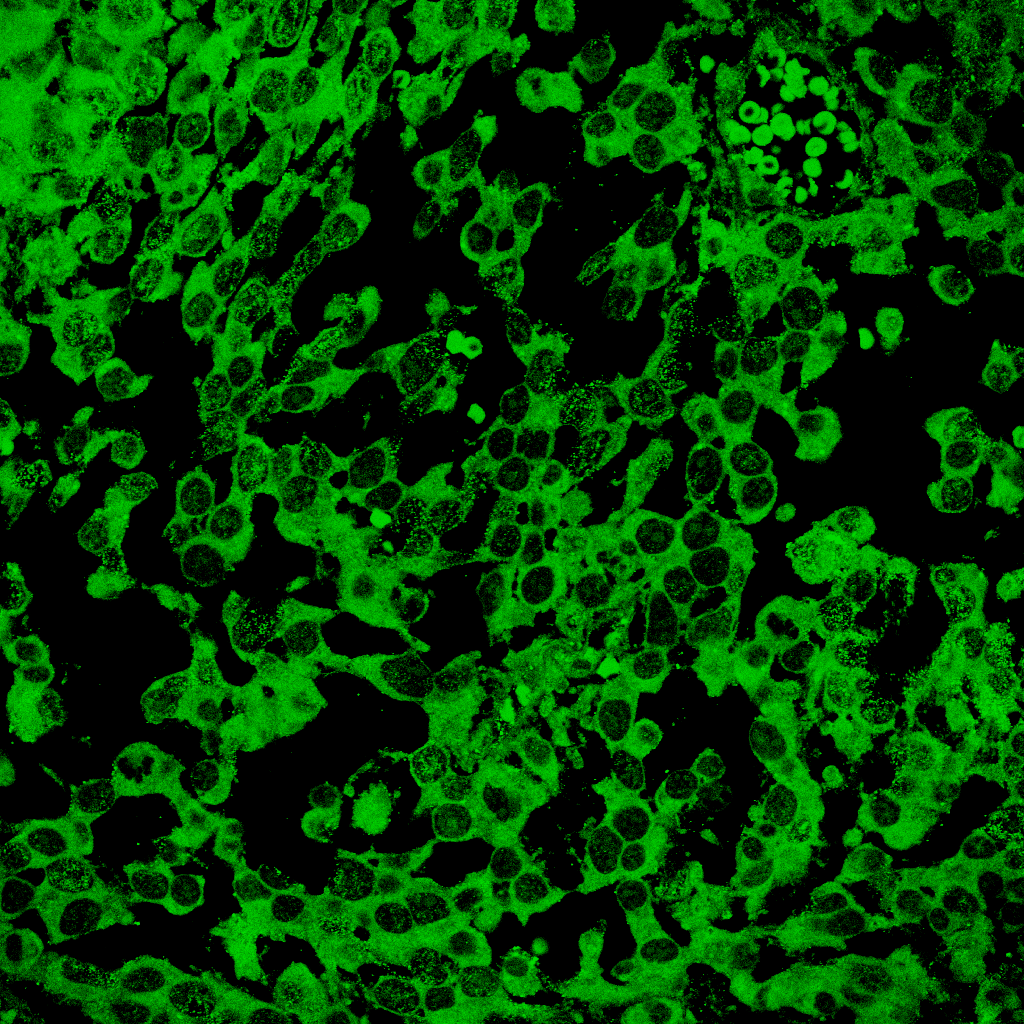** | **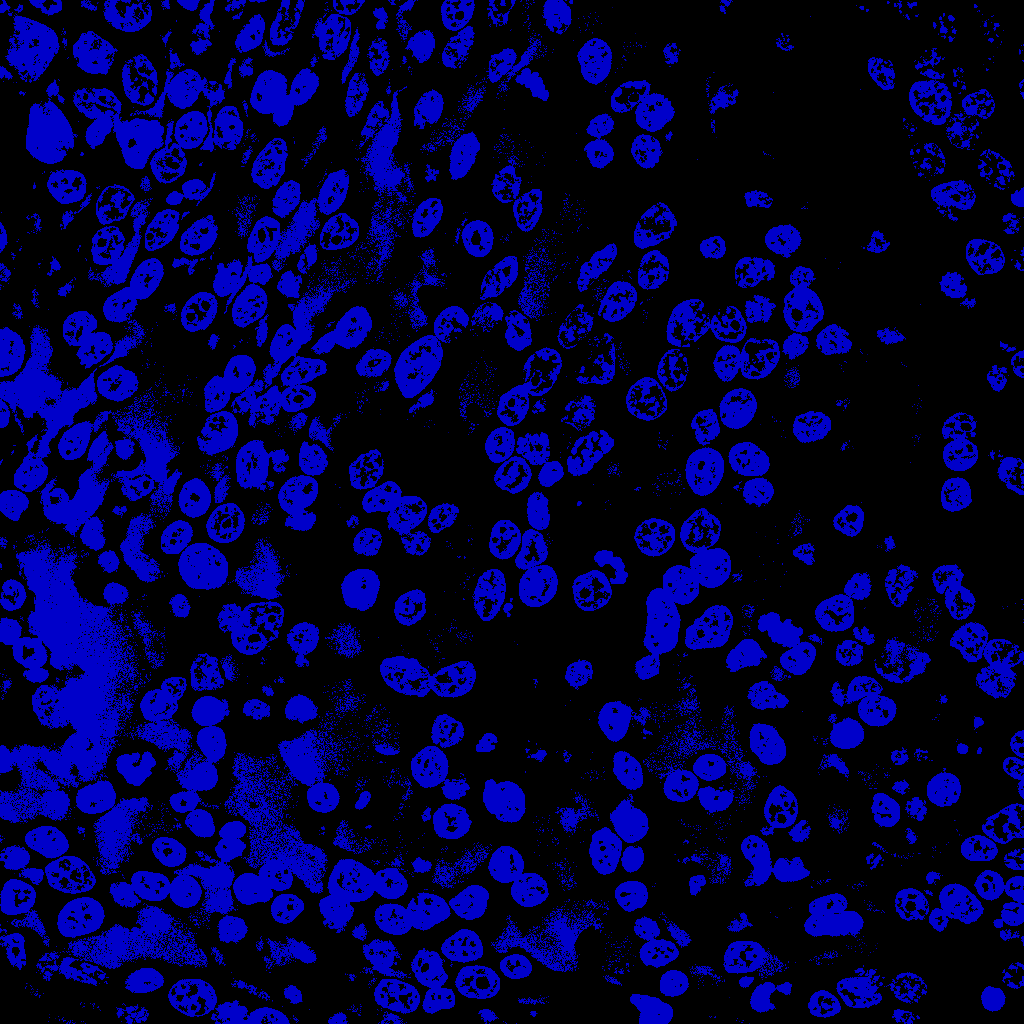** | | **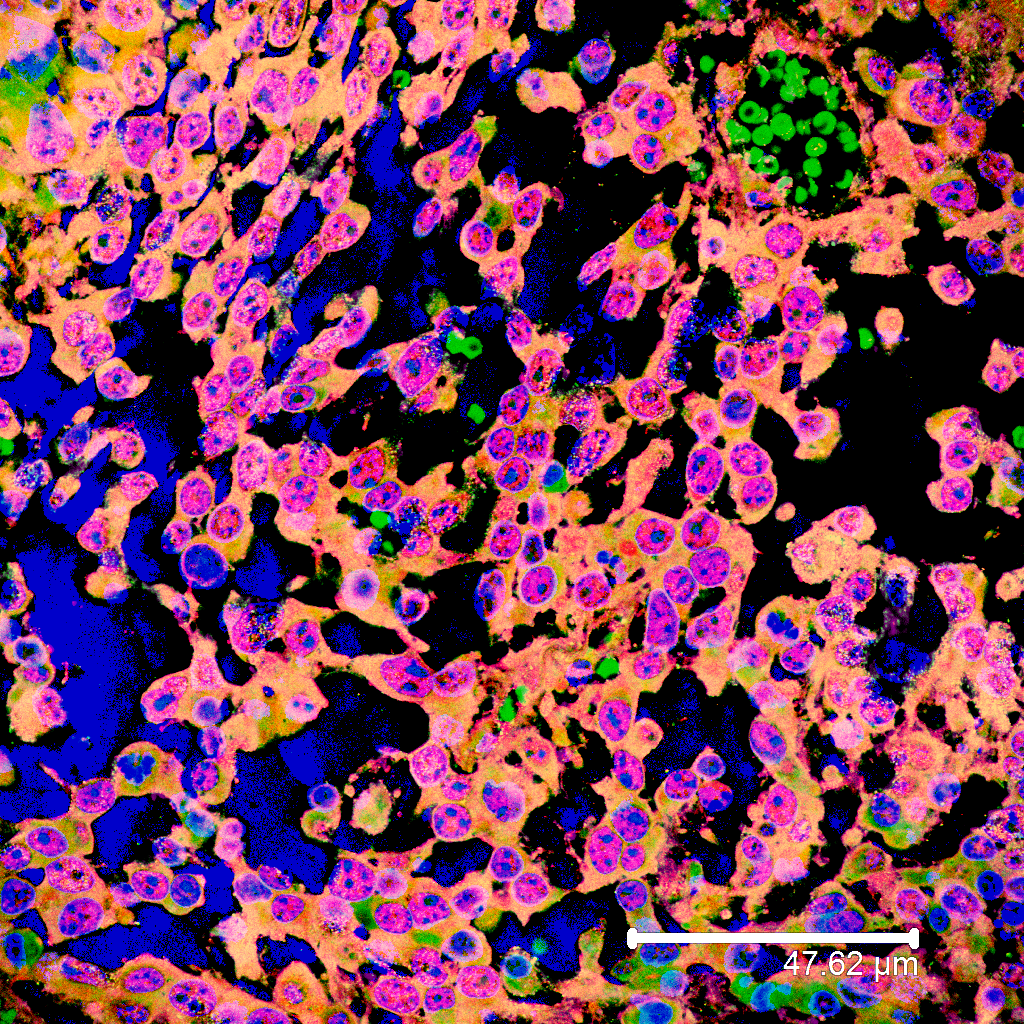** |
| **CCP, Rescued** | **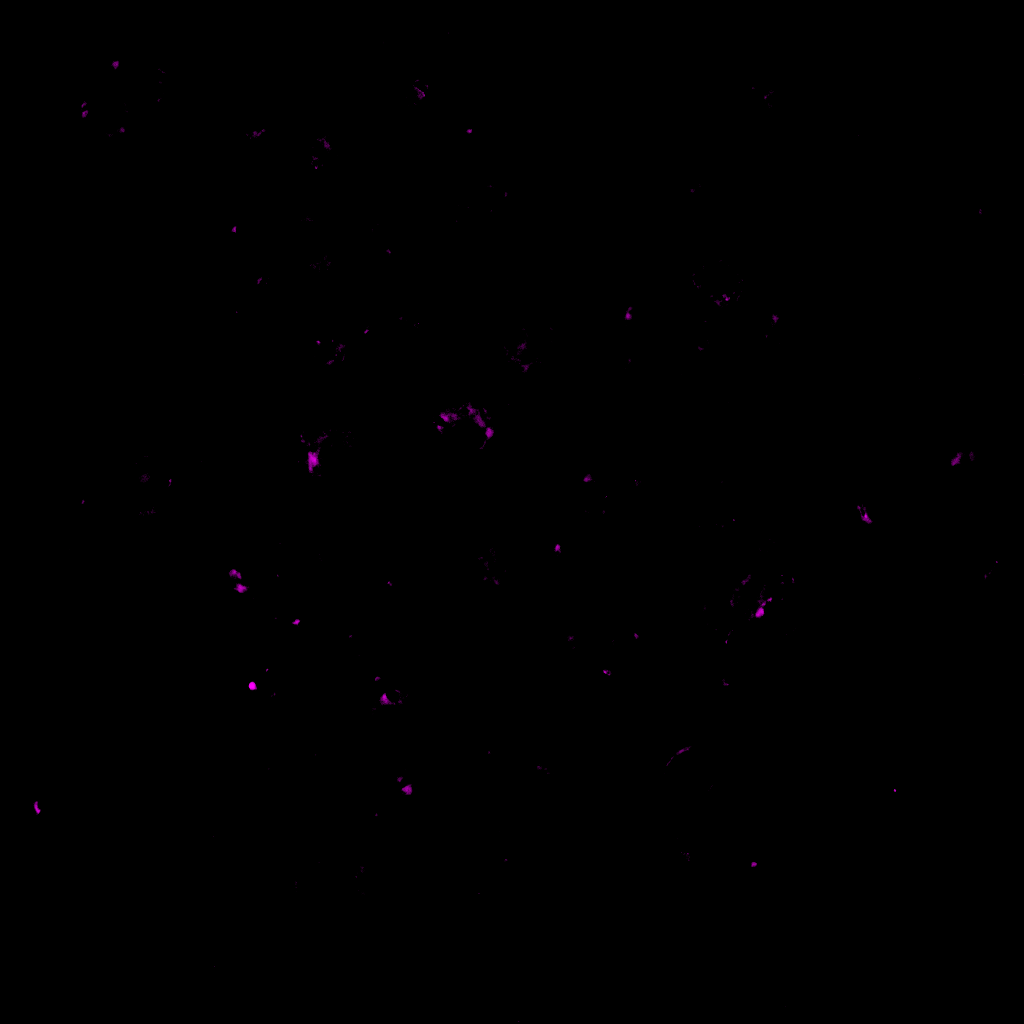** | **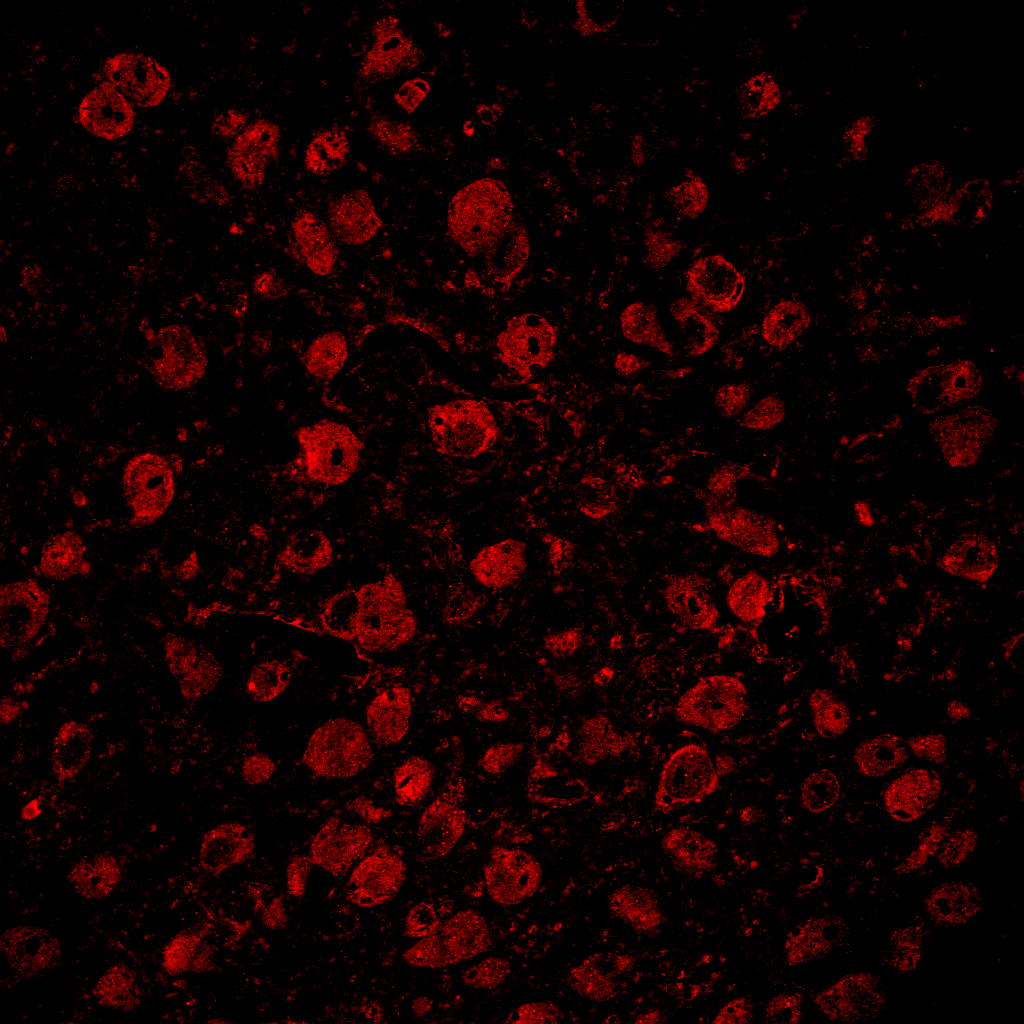** | | **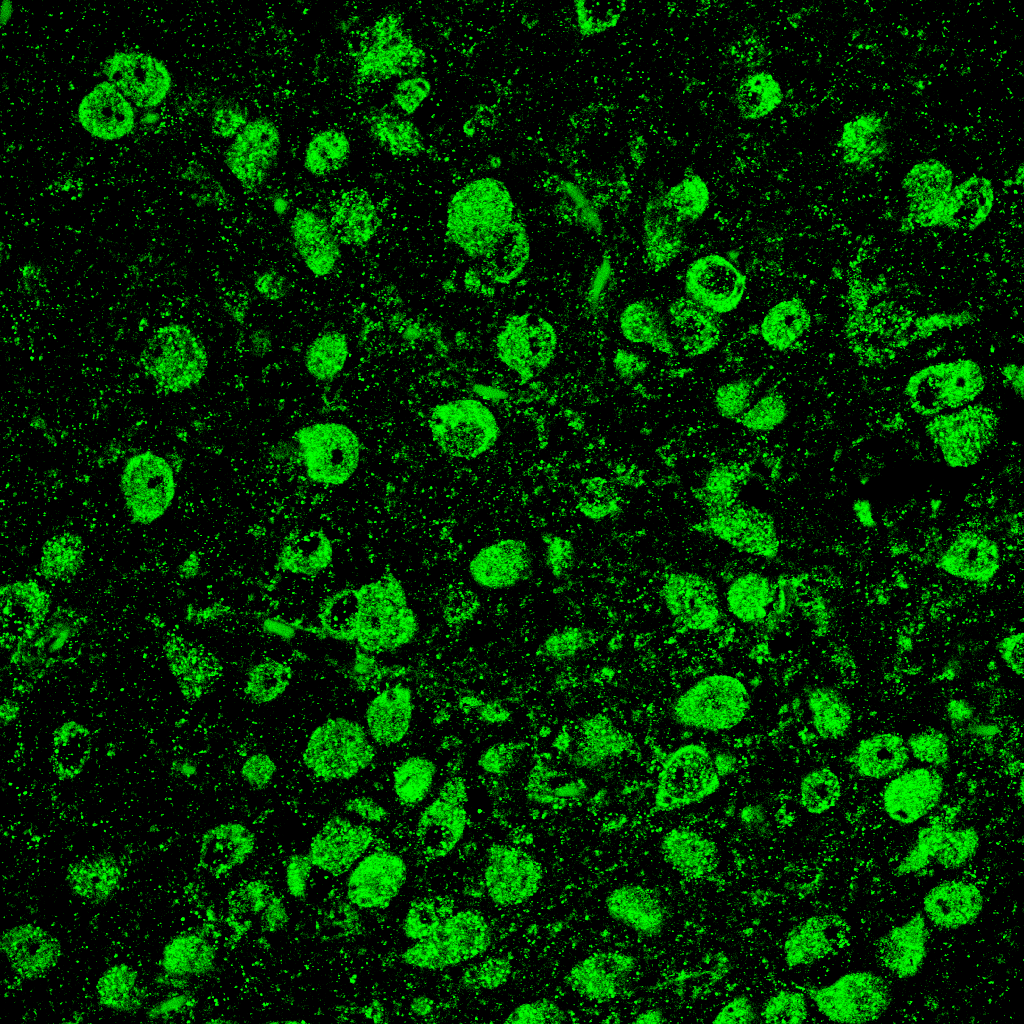** | **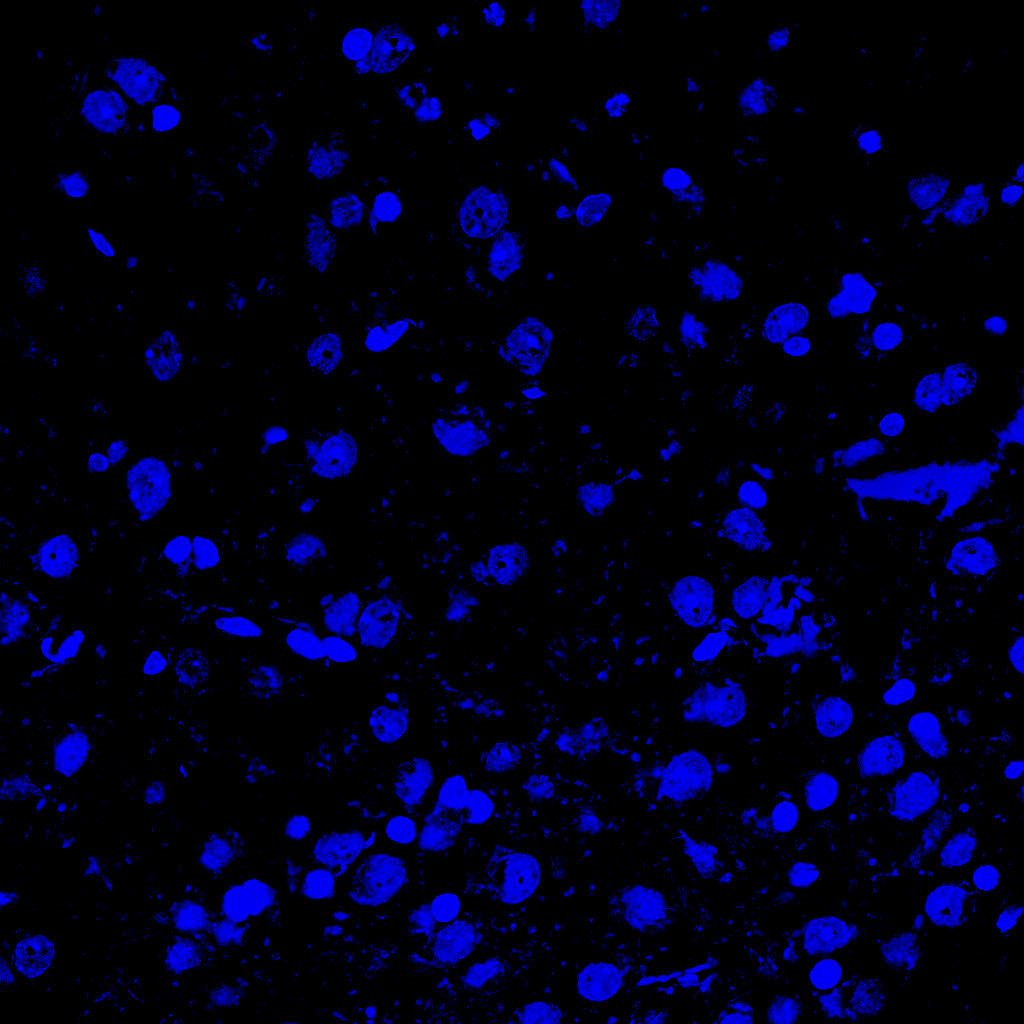** | | **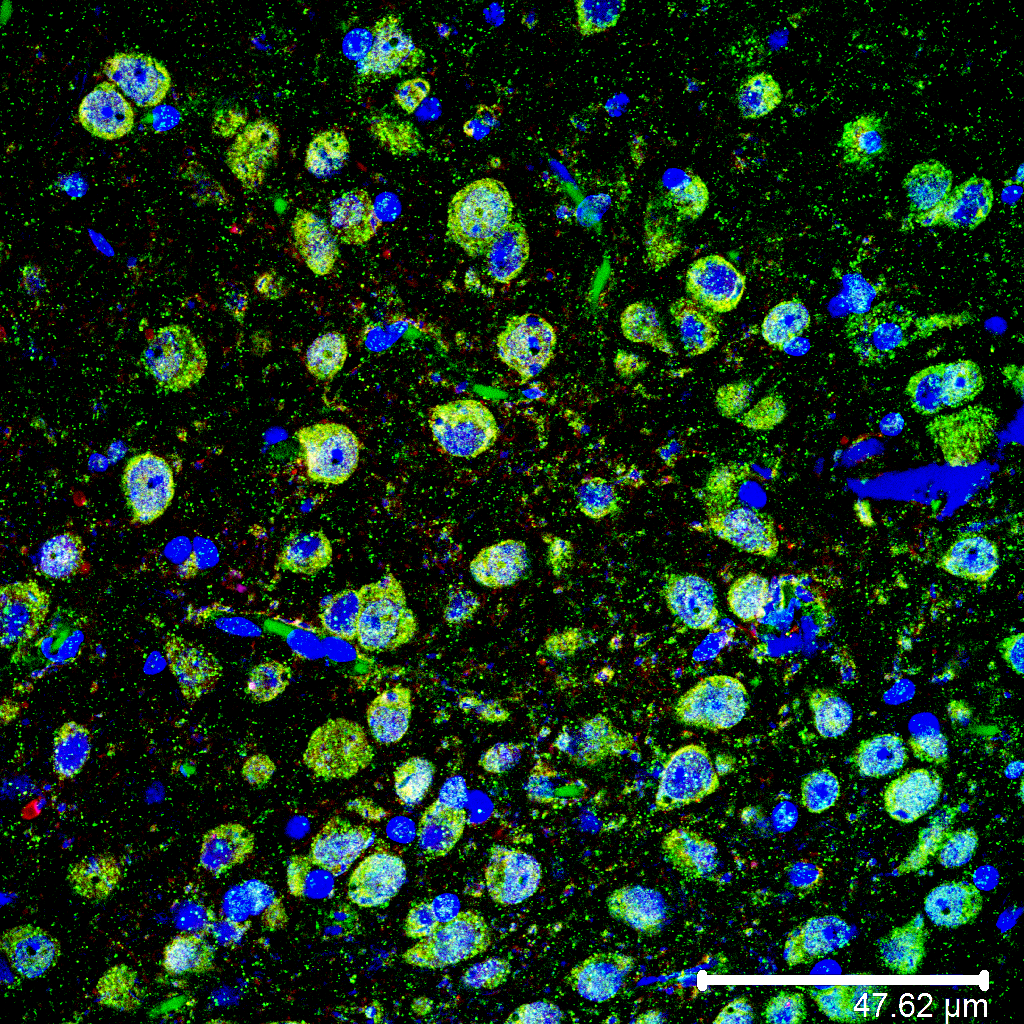** |
|  | **(B)**  **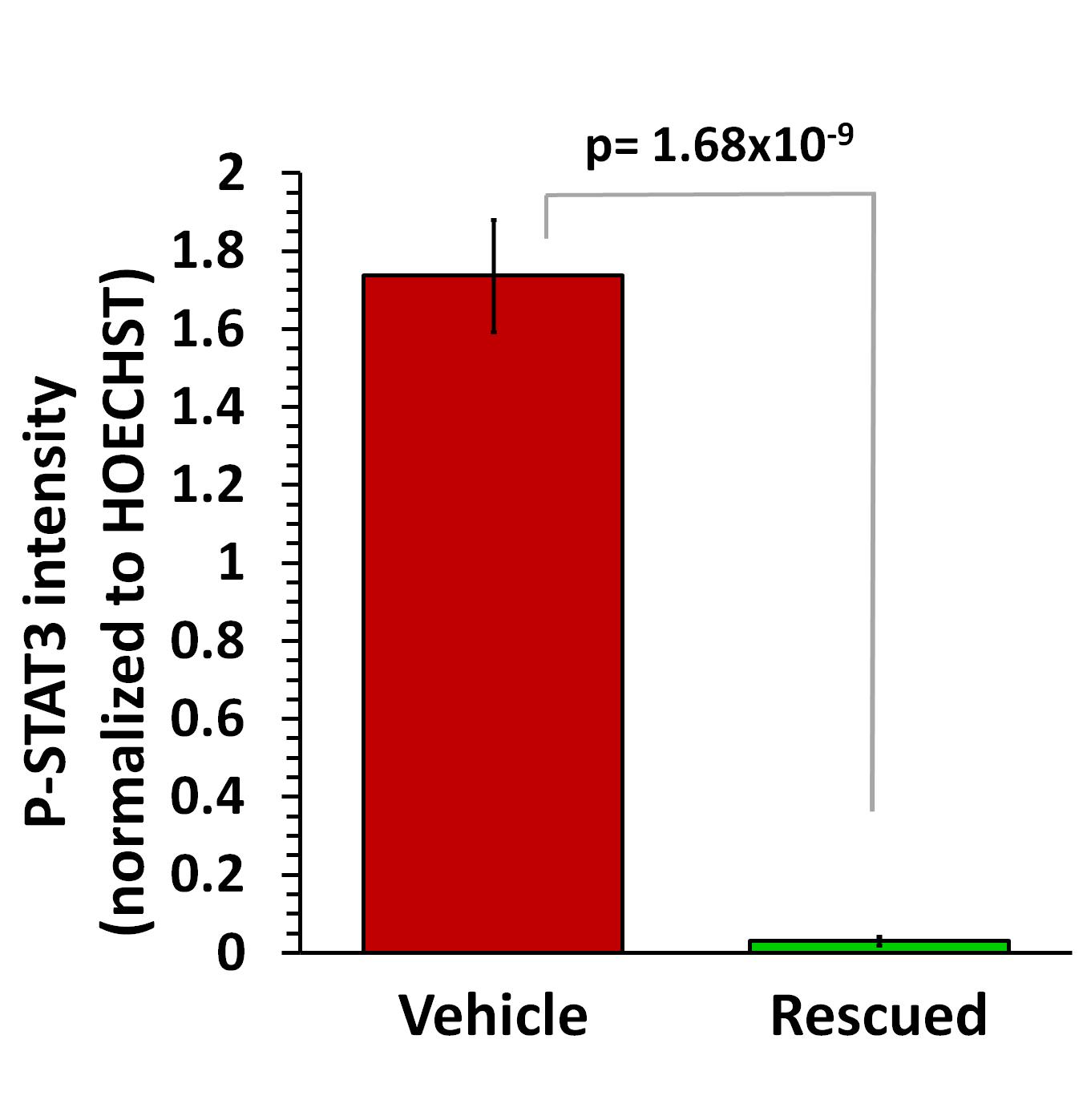** | | **(C)**  **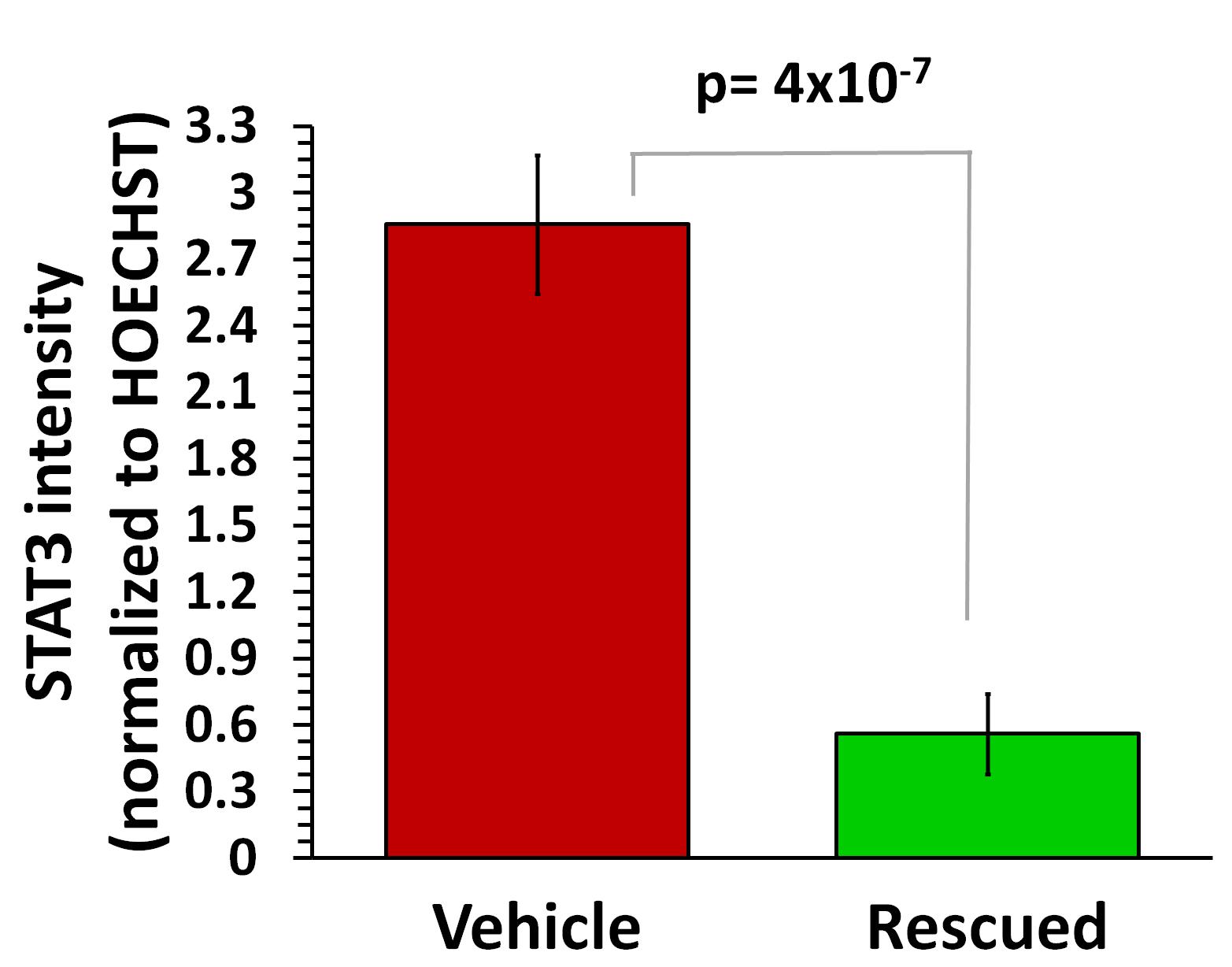** | | | **(D)**  **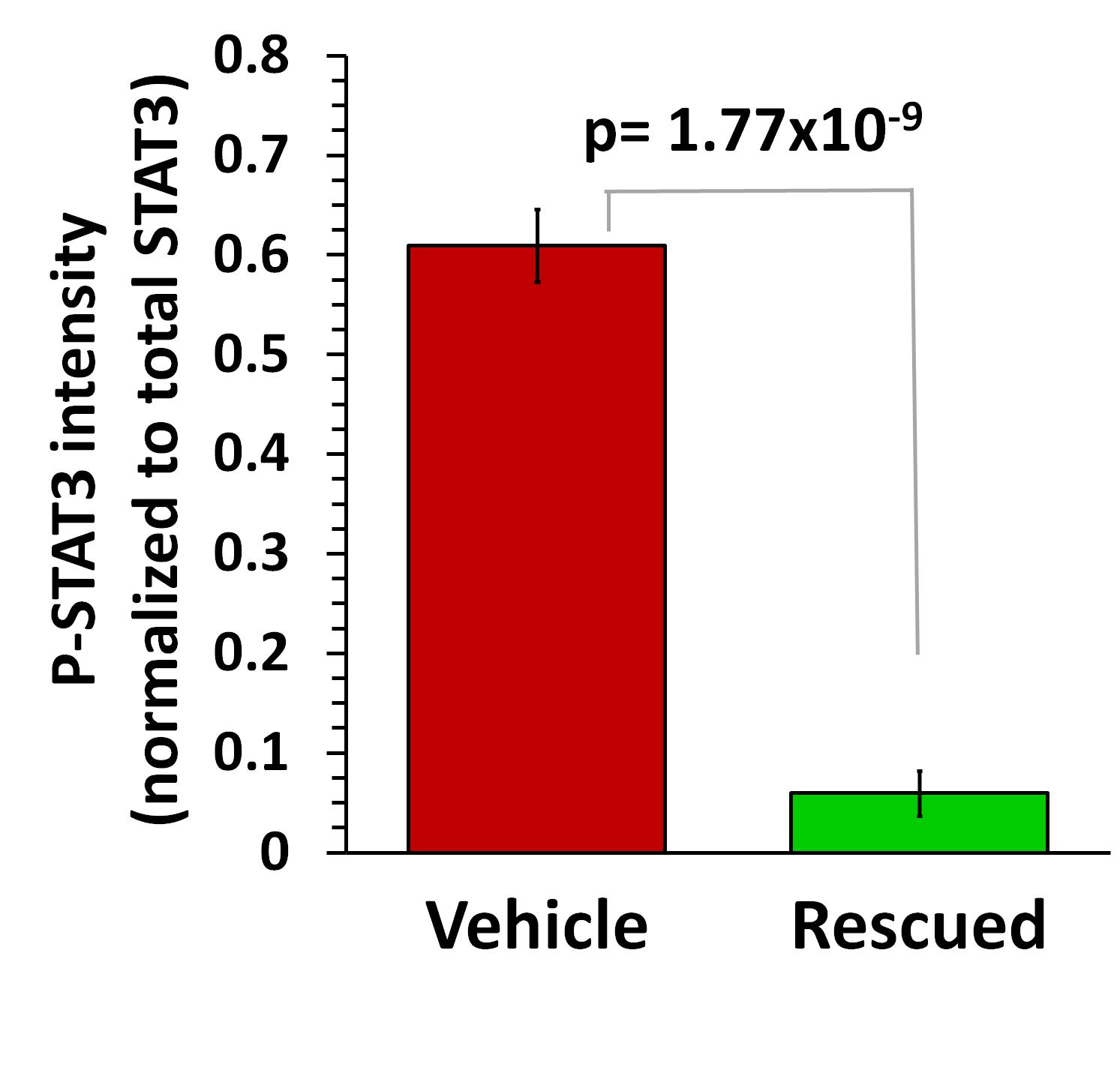** | |

**Additional file 1: Figure S1. The activated (Iba1(+)) microglia/macrophages (TAM) in the CCP-treated and rescued mice display a dramatic inhibition of STAT3.** Brain sections parallel to those used in our previous report [8] were used to assess the levels of STAT3 and P-Tyr^705^-STAT3 (activated) in the Iba1(+) TAM. **(A upper and lower rows)** and **(B)** The Vehicle-treated mice displayed a high level of activated (P-Y^705^-STAT3) TAM, which was suppressed by 98% in the scar tissue sections from CCP-treated and rescued mice. This overall suppression of P-STAT3 was a result of suppression of STAT3 expression (STAT3 normalized to HOECHST) **(C)** and STAT3 activation (P-STAT3 normalized to STAT3) **(D)**. Four sections per mouse were used for imaging and data (mean ± S.D.) were graphically presented as obtained from Vehicle-treated mice (n=3), and CCP-treated and rescued mice (n=3). HOECHST = HOECHST33342. (Scale bar: 47.62 µm).
