# Supplementary material for: Phytosomal curcumin causes natural killer cell-dependent repolarization of glioblastoma (GBM) tumor-associated microglia/macrophages and elimination of GBM and GBM stem cells
Source: J Exp Clin Cancer Res. 2018 Jul 25;37:168. doi: 10.1186/s13046-018-0792-5 (PMC6058381; doi:10.1186/s13046-018-0792-5)
Supplement: Supplementary file 2 — Figure S2. GBM-associated, activated (Iba1(+)) TAM are IL10high and IL12low, whereas the TAM in the scar tissue of CCP-treated and rescued mice are IL10low and IL12high. Brain sections harboring the tumor (Vehicle-treated) and scar tissue (CCP-treated and rescued) were triple-stained with Iba1, IL10 and IL12 antibodies. The TAM in the GBM tissue in Vehicle-treated mice display high levels of IL10 and low levels of IL12 (A, upper row), whereas the scar tissue from the CCP-treated and rescued mice displayed IL10low and IL12high TAM (A, lower row, B and C). The graphs show a 91% decrease in IL10 and a 300% increase in IL12 (CCP IL12: 400% of Vehicle-treated) (mean ± S.D.) following CCP-treatment (n = 3 per group, with four randomly chosen brain sections from each mouse). (Scale bar: 47.62 μm). HOECHST = HOECHST33342. (DOC 1761 kb) [file 13046_2018_792_MOESM2_ESM.doc]

| **(A)** | **IL10** | **IL12 Iba1 HOECHST** | | | | **Merged** |
| --- | --- | --- | --- | --- | --- | --- |
| **Vehicle** | 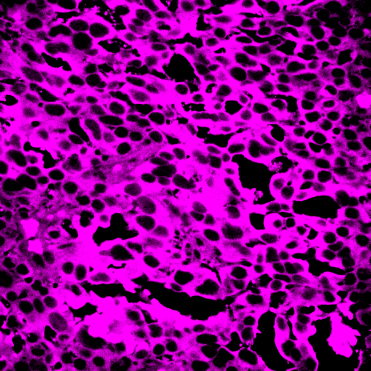 | 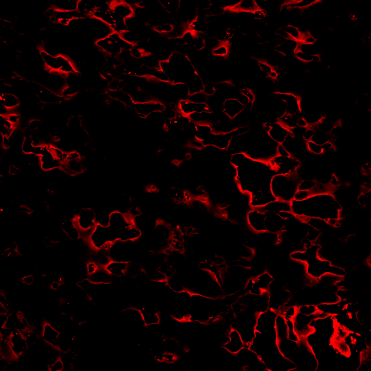 | 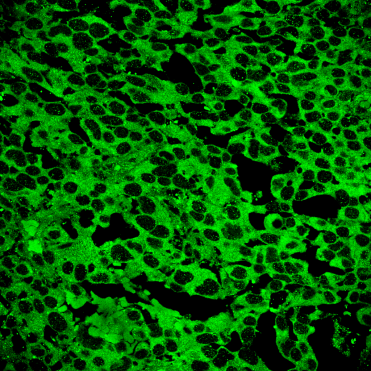 | | 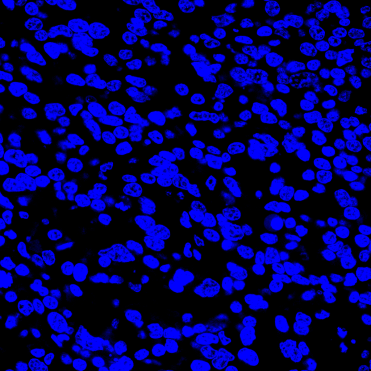 | 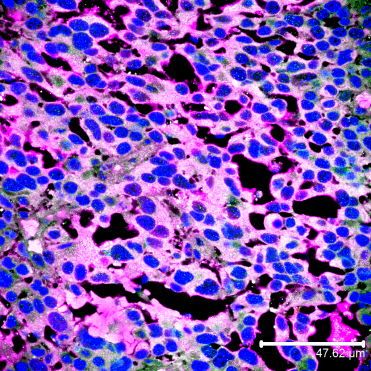 |
| **CCP, Rescued** | 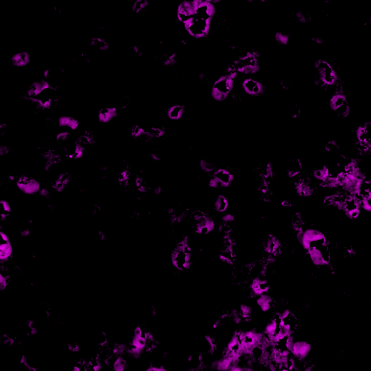 | 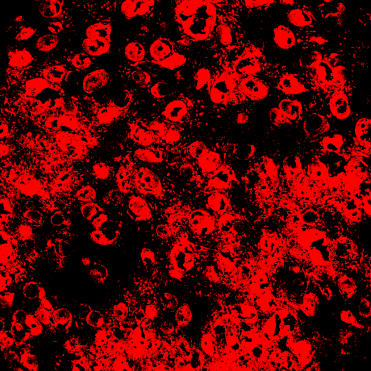 | 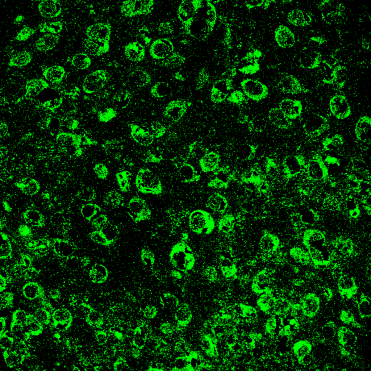 | | 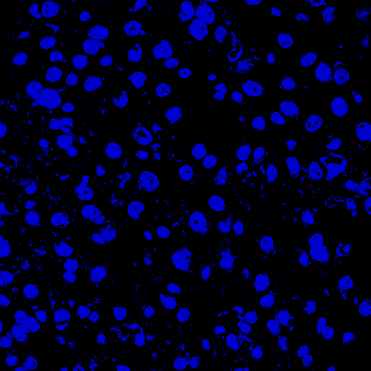 | 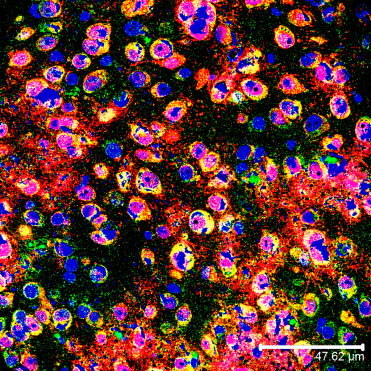 |
|  | **(B) (D)** | | | **(C)** | | |
|  | 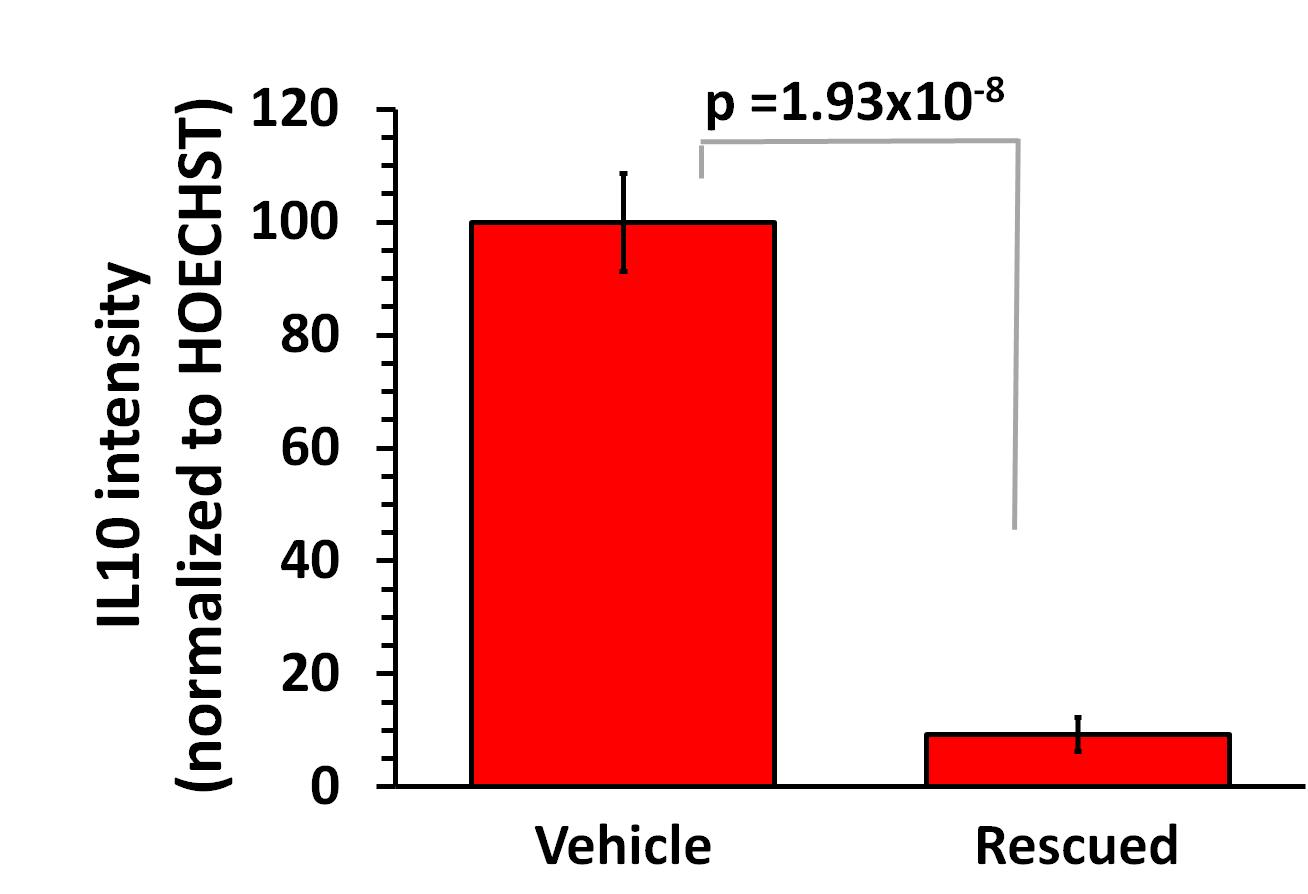 | | | **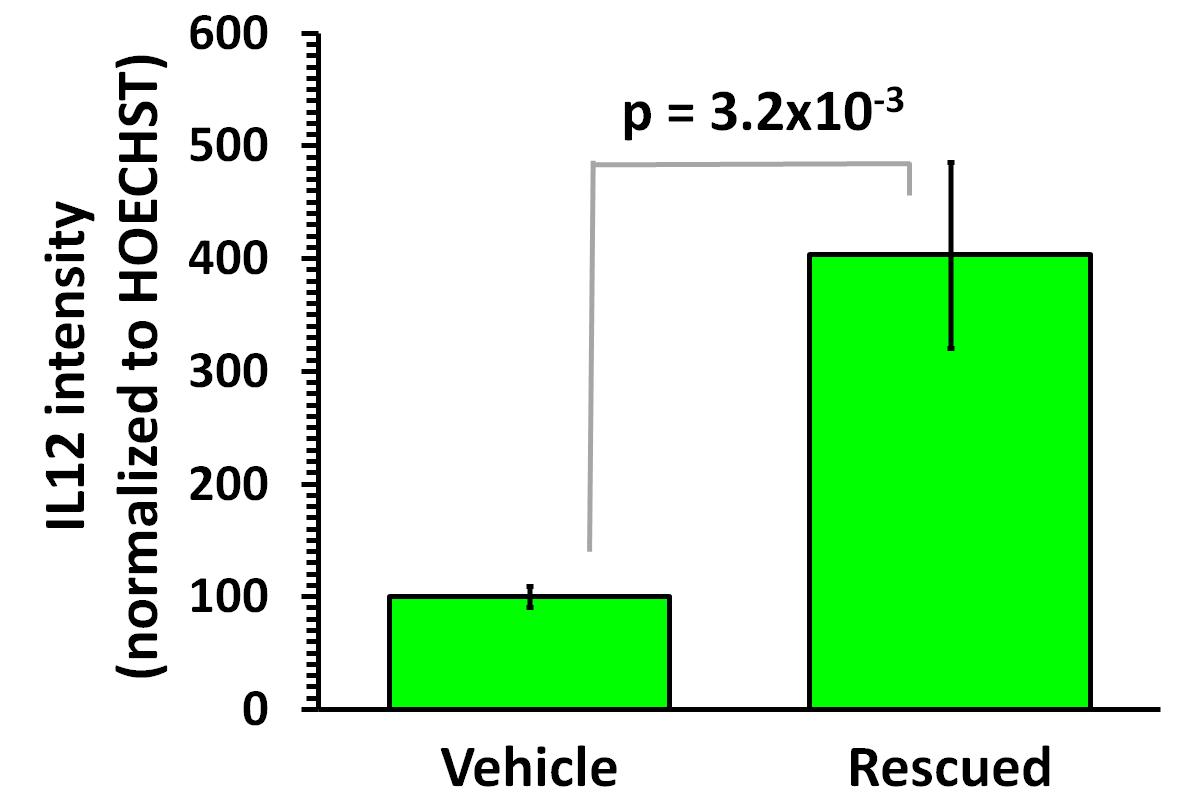** | | |

**Additional file 2: Figure S2. GBM-associated, activated (Iba1(+)) TAM are IL10^high^ and IL12^low^, whereas the TAM in the scar tissue of CCP-treated and rescued mice are IL10^low^ and IL12^high^.** Brain sections harboring the tumor (Vehicle-treated) and scar tissue (CCP-treated and rescued) were triple-stained with Iba1, IL10 and IL12 antibodies. The TAM in the GBM tissue in Vehicle-treated mice display high levels of IL10 and low levels of IL12 **(A, upper row)**, whereas the scar tissue from the CCP-treated and rescued mice displayed IL10^low^ and IL12^high^ TAM **(A, lower row, B and C).** The graphs show a 91% decrease in IL10 and a 300% increase in IL12 (CCP IL12: 400% of Vehicle-treated) (mean ± S.D.) following CCP-treatment (n=3 per group, with four randomly chosen brain sections from each mouse). (Scale bar: 47.62 µm). HOECHST = HOECHST33342.
