# Supplementary material for: Phytosomal curcumin causes natural killer cell-dependent repolarization of glioblastoma (GBM) tumor-associated microglia/macrophages and elimination of GBM and GBM stem cells
Source: J Exp Clin Cancer Res. 2018 Jul 25;37:168. doi: 10.1186/s13046-018-0792-5 (PMC6058381; doi:10.1186/s13046-018-0792-5)
Supplement: Supplementary file 3 — Figure S3. GBM tumor generation by GL261 implantation on day 1, peripheral treatment with mouse immunoglobulin (mouse Ig or MIg) or the NK-cell-neutralizing NK1.1 antibody on day 11, and a regimen of Vehicle (PBS) or CCP treatment for five days yields tumors in all the groups on day 17. (A) As shown by near-IR scanning, implantation of 105 GL261 cells on day 1 resulted in large tumors on day 17 in the mice from all the groups (see below). A representative Vehicle-treated tumor (yellow) is shown here. (B) On day 11, GBM-mice (n = 14) were randomly divided into three groups: ‘Vehicle’ (n = 5), ‘CCP’ (n = 5), and ‘CCP + NK1.1’ (n = 4). The mice in the CCP + NK1.1 group received intra-peritoneal (i.p.) infusion of the NK cell-neutralizing anti-NK1.1 antibody (100 μg/mouse). On day 12, each mouse received i.p. injections of sterile PBS (Vehicle group) or CCP (2 mg/ mouse/day) for the CCP and CCP + NK1.1 groups for five days. On day 17, all mice were sacrificed. (C) Forward- versus side-scatter plot showing the population of cells chosen for the analysis of fluorescence. (D) The day-17 GBM tumors from mice of all the groups were extricated, dissociated, cells fixed and then subjected to immunostaining for CD68 and Iba1 followed by flow cytometry analysis to confirm the presence of established GBM tumors [8, 10]. Two segregated populations of CD68(+) cells were identified [10]. The larger population (presumably GBM cells) were Iba1(−) and CD68high, whereas the Iba1(+) but CD68low cells were expected to be the TAM [8, 10, 18]. (E and F) The Iba1(−)/CD68high (GBM cells) in the GBM samples showed 680% higher integrated CD68 fluorescence (fluorescence per cell in arbitrary units X number of events) × 108 relative to the Iba1(+)/CD68low (TAM cells) (p = 1.7 × 10− 4). The graph represents mean ± S.D. obtained from the analysis of GBM mice (n = 6). (DOC 390 kb) [file 13046_2018_792_MOESM3_ESM.doc]

| **(A) (B)**  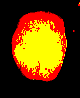 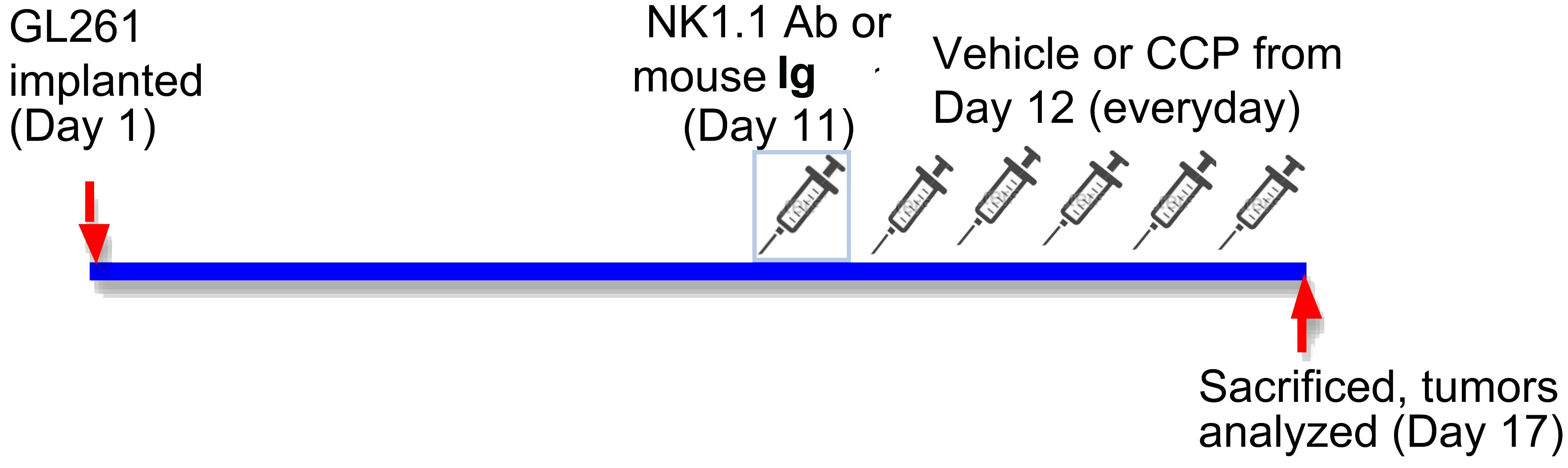  **CD68^low^ CD68^high^**  **Day 17 Vehicle** | | |
| --- | --- | --- |
| **(C)**  **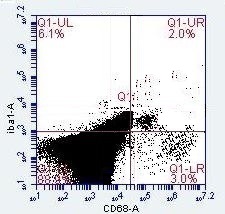**  **CD68^low^ CD68^high^**  **Iba1(+) Iba1(-)** | **(D)**  **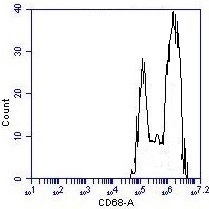** | **(E)**  **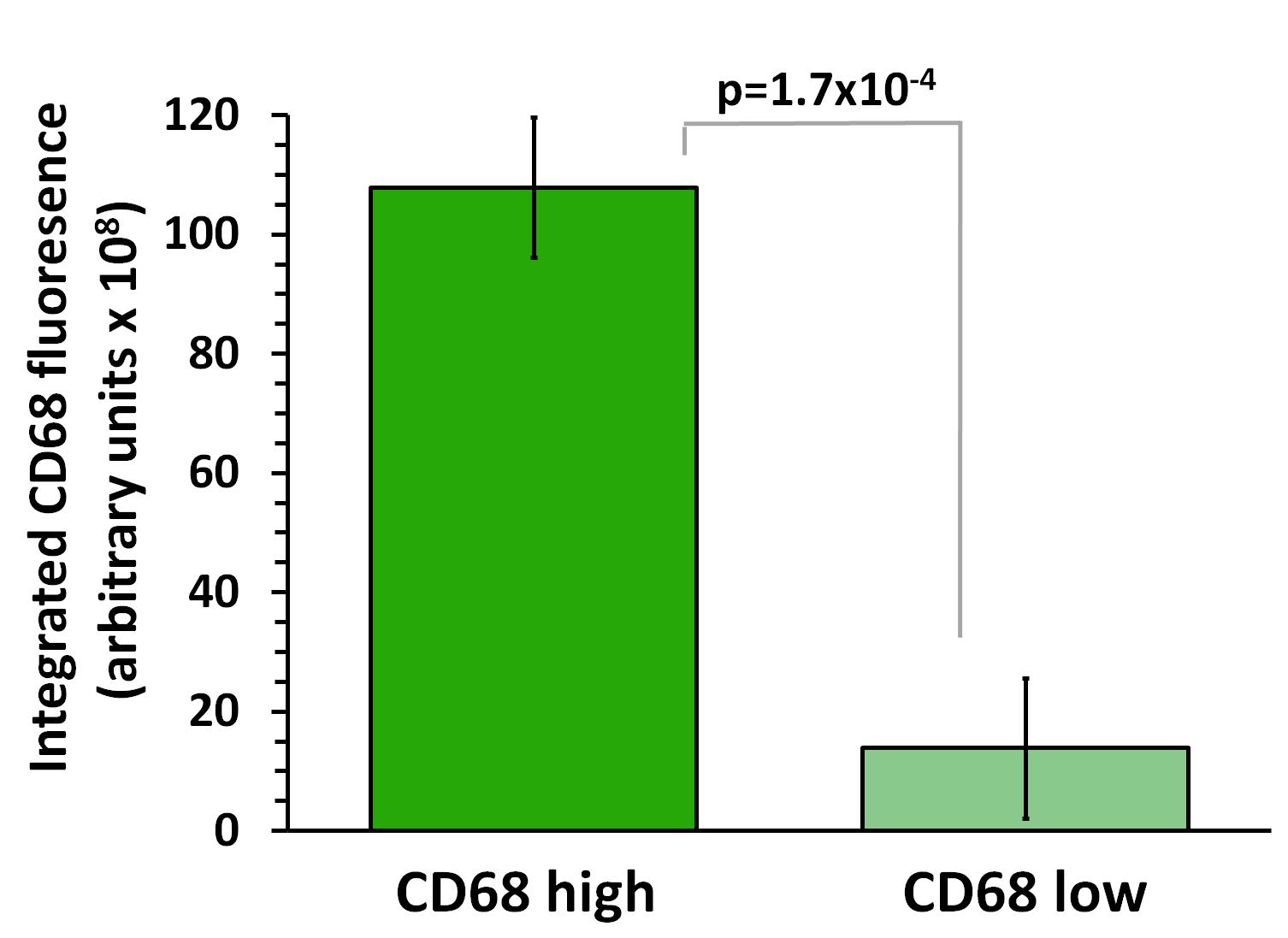** |

**Additional file 3: Figure S3. GBM tumor generation by GL261 implantation on day 1, peripheral treatment with mouse immunoglobulin (mouse Ig or MIg) or the NK-cell-neutralizing NK1.1 antibody on day 11, and a regimen of Vehicle (PBS) or CCP treatment for five days yields tumors in all the groups on day 17.** **(A)** As shown by near-IR scanning**,** implantation of 10^5^ GL261 cells on day 1 resulted in large tumors on day 17 in the mice from all the groups (see below). A representative Vehicle-treated tumor (yellow) is shown here. **(B)** On day 11, GBM-mice (n=14) were randomly divided into three groups: 'Vehicle' (n=5), 'CCP' (n=5), and 'CCP+NK1.1' (n=4). The mice in the CCP+NK1.1 group received intra-peritoneal (i.p.) infusion of the NK cell-neutralizing anti-NK1.1 antibody (100 µg/mouse). On day 12, each mouse received i.p. injections of sterile PBS (Vehicle group) or CCP (2 mg/ mouse/day) for the CCP and CCP+NK1.1 groups for five days. On day 17, all mice were sacrificed. **(C)** Forward- versus side-scatter plot showing the population of cells chosen for the analysis of fluorescence. **(D)** The day-17 GBM tumors from mice of all the groups were extricated, dissociated, cells fixed and then subjected to immunostaining for CD68 and Iba1 followed by flow cytometry analysis to confirm the presence of established GBM tumors [8, 10]. Two segregated populations of CD68(+) cells were identified [10]. The larger population (presumably GBM cells) were Iba1(-) and CD68^high^, whereas the Iba1(+) but CD68^low^ cells were expected to be the TAM [8, 10, 18]. **(E and F)** The Iba1(-)/CD68^high^ (GBM cells) in the GBM samples showed 680% higher integrated CD68 fluorescence (fluorescence per cell in arbitrary units X number of events) x10^8^ relative to the Iba1(+)/CD68^low^ (TAM cells) (p=1.7x10^-4^). The graph represents mean ± S.D. obtained from the analysis of GBM mice (n = 6).
