# Supplementary material for: Phytosomal curcumin causes natural killer cell-dependent repolarization of glioblastoma (GBM) tumor-associated microglia/macrophages and elimination of GBM and GBM stem cells
Source: J Exp Clin Cancer Res. 2018 Jul 25;37:168. doi: 10.1186/s13046-018-0792-5 (PMC6058381; doi:10.1186/s13046-018-0792-5)
Supplement: Supplementary file 4 — Figure S4. CCP treatment causes intra-tumor recruitment of activated NK cells, which is eliminated in mice peripherally administered with an IL12 antibody. After intracranial implantation of 105 GL261 cells on day 1, the GBM-mice (n = 9) were randomly divided into three groups: ‘Vehicle’ (n = 3), ‘CCP’ (n = 3), and ‘CCP + IL12Ab’ (n = 3). On day 11 and day 14, the CCP + IL12Ab group received NK cell-neutralizing anti-IL12p40 Ab (100 μg/mouse/day), whereas the other two groups received rat Ig (100 μg/mouse) (i.p.). From day 12, each mouse received CCP (2 mg/ mouse/day in 200 μl PBS) for the CCP and CCP + IL12Ab groups and sterile PBS for the “Vehicle” group for five days. On day 17, all mice were sacrificed and the dispersed cells were immunostained for flow cytometry analysis. CCP-treated samples display a 270% increase in integrated NKp46 fluorescence (370% of Vehicle-treated) (A, B and C; *p = 0.01, CCP versus Vehicle). The NKp46 fluorescence was virtually eliminated in the GBM samples from the CCP + IL12Ab mice (A, B and C; Δ p = 4.4 × 10− 4, CCP + IL12Ab versus CCP; **p = 2.7 × 10− 3, CCP + IL12Ab versus Vehicle-treated). The graphs represent data (mean ± S.D.) obtained from Vehicle (n = 3), CCP (n = 3), and CCP + IL12Ab (n = 3). Integrated fluorescence = average fluorescence per cell X total number of cells (events) in a segregated population. (DOC 1976 kb) [file 13046_2018_792_MOESM4_ESM.doc]

| **(A)** | **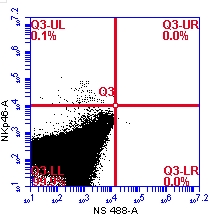** | **(B) Flow Cytometry (Profile)** |
| --- | --- | --- |
| **Vehicle** |  | **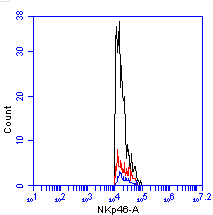** |
| **CCP** | **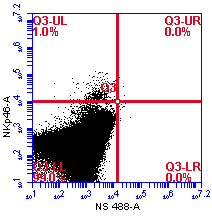** | **(C)**  **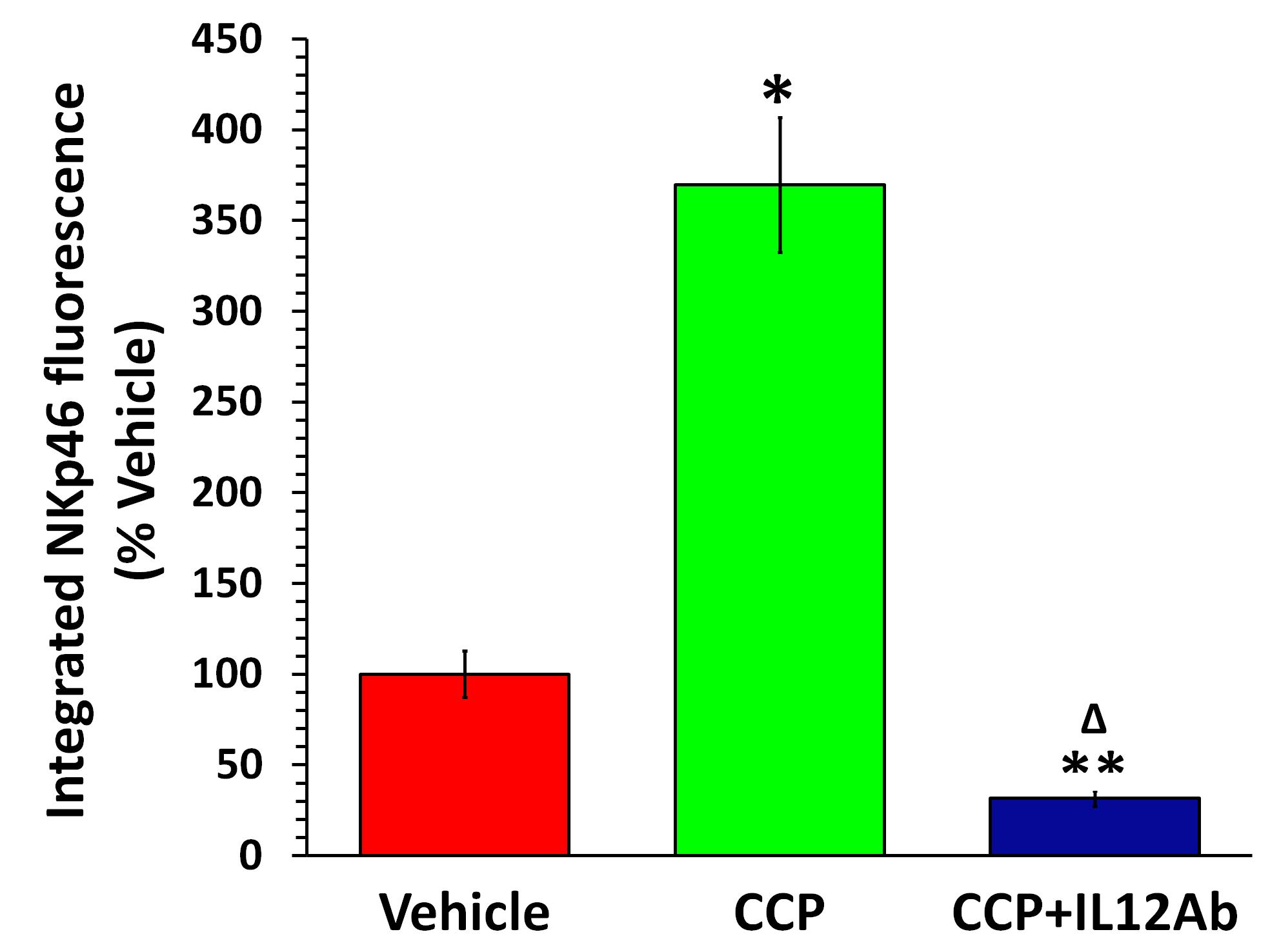** |
| **CCP + IL12Ab** | **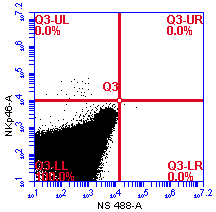** |  |

**CCP**

**CCP+IL12Ab**

**Veh**

**Additional file 4: Figure S4. CCP treatment causes intra-tumor recruitment of activated NK cells, which is eliminated in mice peripherally administered with an IL12 antibody.** After intracranial implantation of 10^5^ GL261 cells on day 1, the GBM-mice (n=9) were randomly divided into three groups: 'Vehicle' (n=3), 'CCP' (n=3), and 'CCP+IL12Ab' (n=3). On day 11 and day 14, the CCP+IL12Ab group received NK cell-neutralizing anti-IL12p40 Ab (100 µg/mouse/day), whereas the other two groups received rat Ig (100 µg/mouse) (i.p.). From day 12, each mouse received CCP (2 mg/ mouse/day in 200 µl PBS) for the CCP and CCP+IL12Ab groups and sterile PBS for the “Vehicle” group for five days. On day 17, all mice were sacrificed and the dispersed cells were immunostained for flow cytometry analysis. CCP-treated samples display a 270% increase in integrated NKp46 fluorescence (370% of Vehicle-treated) (**A**, **B and C;** *p = 0.01, CCP versus Vehicle). The NKp46 fluorescence was virtually eliminated in the GBM samples from the CCP+IL12Ab mice (**A**, **B and C;** **∆** p = 4.4 x10^-4^, CCP+IL12Ab versus CCP; **p = 2.7x10^-3^, CCP+IL12Ab versus Vehicle-treated). The graphs represent data (mean ± S.D.) obtained from Vehicle (n=3), CCP (n=3), and CCP+IL12Ab (n=3). Integrated fluorescence = average fluorescence per cell X total number of cells (events) in a segregated population.
