# Supplementary material for: Phytosomal curcumin causes natural killer cell-dependent repolarization of glioblastoma (GBM) tumor-associated microglia/macrophages and elimination of GBM and GBM stem cells
Source: J Exp Clin Cancer Res. 2018 Jul 25;37:168. doi: 10.1186/s13046-018-0792-5 (PMC6058381; doi:10.1186/s13046-018-0792-5)
Supplement: Supplementary file 5 — Figure S5. Peripheral neutralization of NK cells by pre-injecting with the NK1.1 Ab partially reverses the CCP-mediated suppression of STAT3 in the TAM. GBM Brain sections parallel to those used in Fig. 3 from the three groups (Vehicle, CCP and CCP + NK1.1Ab) were used to assess the levels of STAT3 and activated STAT3 (P-Y705-STAT3) (P-STAT3) in the Iba1(+) TAM. (A) The GBM sections from the Vehicle-treated mice displayed high levels of STAT3 and P-STAT3 (top row), whereas the CCP-treated mice showed an 88.5% decrease in P-STAT3 (normalized to HOECHST) (*p = 5.1 × 10− 5, CCP-treated versus Vehicle) and this CCP-evoked suppression was only by 61% in samples obtained from the CCP + NK1.1 mice (Δ p = 5.9 × 10− 3, CCP + NK1.1 versus CCP-treated) (B). The CCP-evoked 88.5% suppression of P-STAT3 in the TAM was the result of a 79% decrease in STAT3 (normalized to HOECHST) (only 68% in the CCP + NK1.1 mice) (C), and a 68% decrease P-STAT3 (normalized to STAT3) (only 48% in CCP + NK1.1) (P-STAT3 normalized to STAT3) (**p = 1.2 × 10− 4 Vehicle versus CCP + NK1.1) (D). Three sections per mouse were used for imaging and the graphs represent mean ± S.D. obtained from Vehicle (n = 4), CCP (n = 4), and CCP + NK1.1 (n = 4). (Scale bar: 47.62 μm). (DOC 8919 kb) [file 13046_2018_792_MOESM5_ESM.doc]

| **(A)** | **P-STAT3** | | **STAT3** | | **Iba1** | **HOECHST** | | **Merged** |
| --- | --- | --- | --- | --- | --- | --- | --- | --- |
| **Vehicle** | **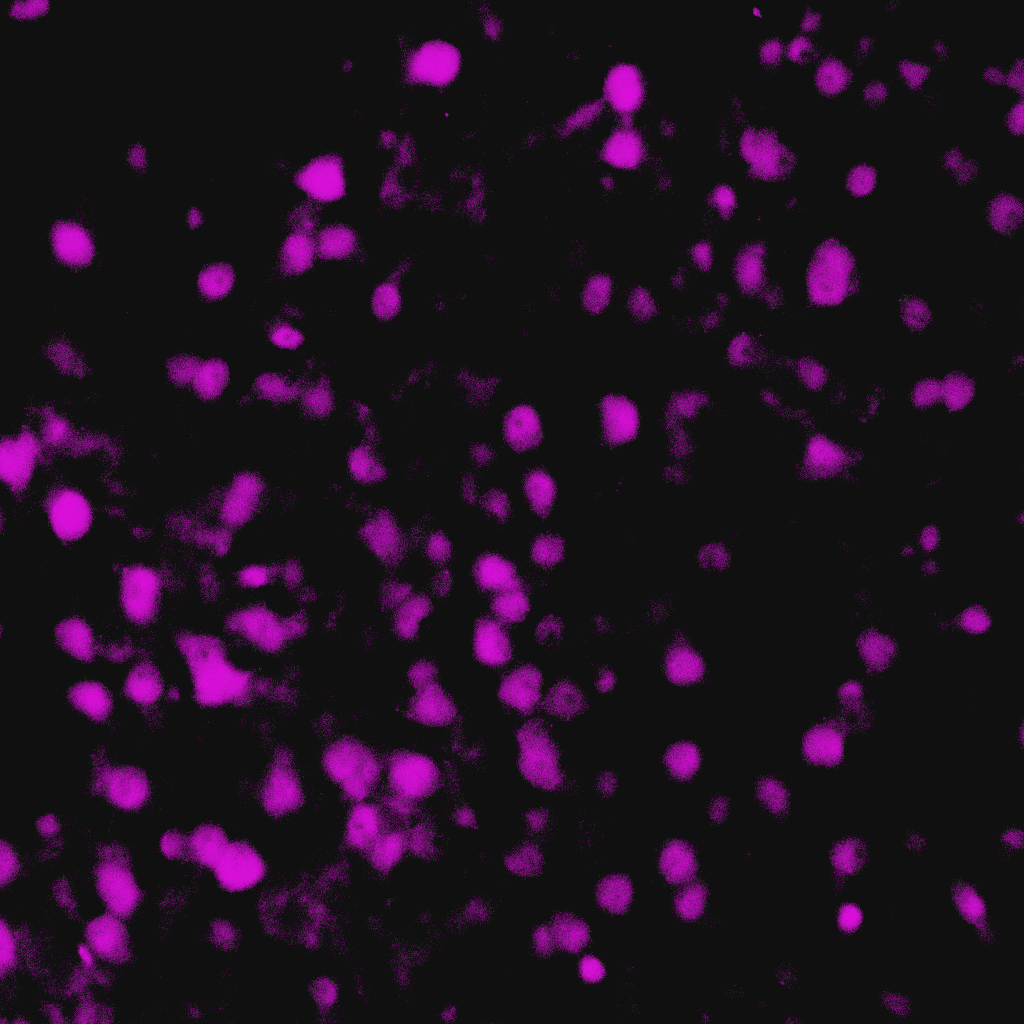** | **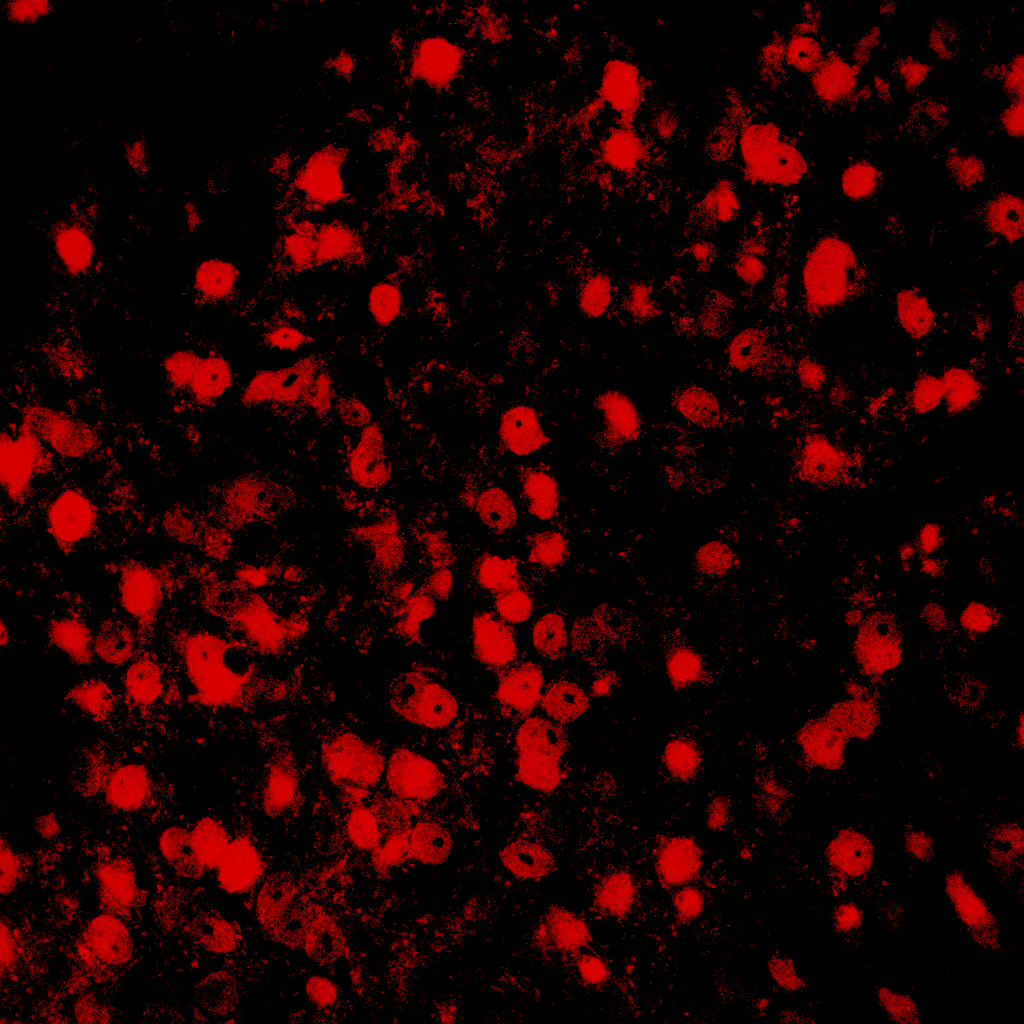** | | | **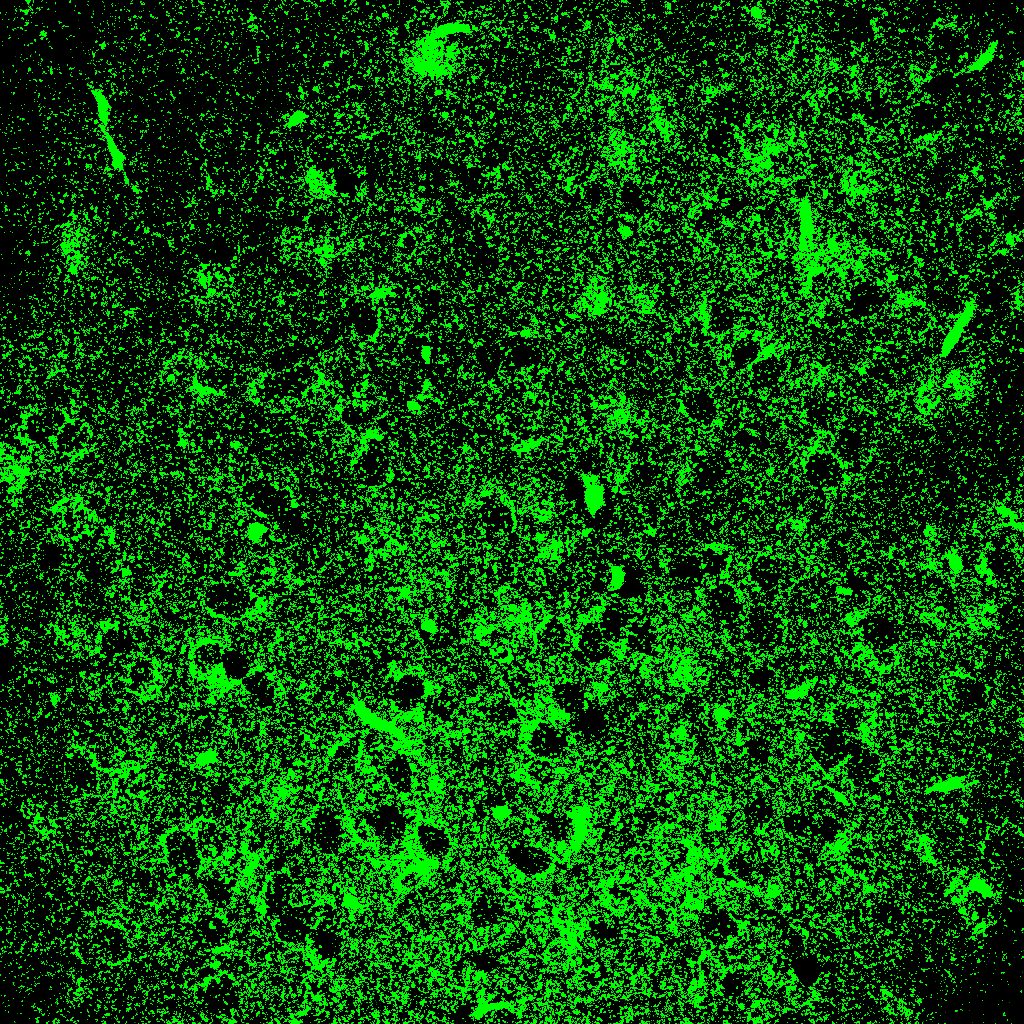** | **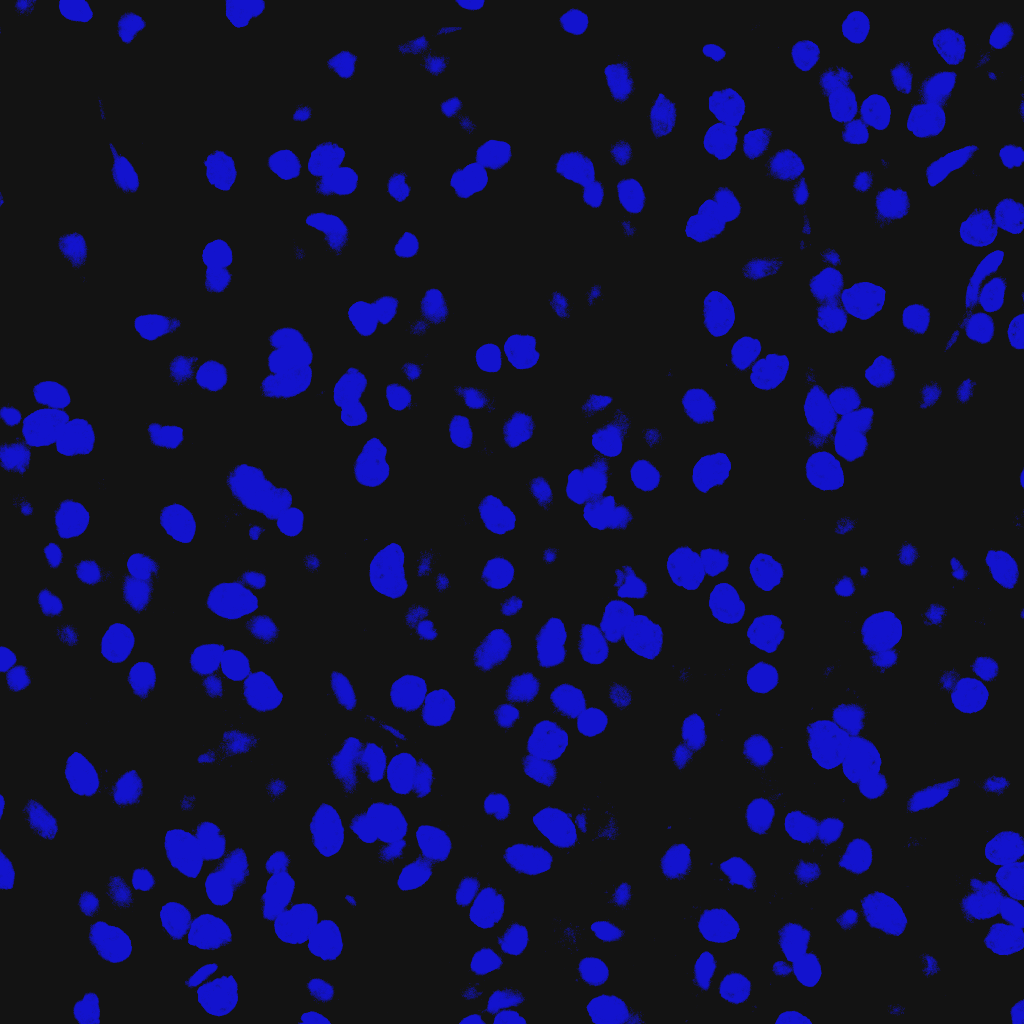** | | **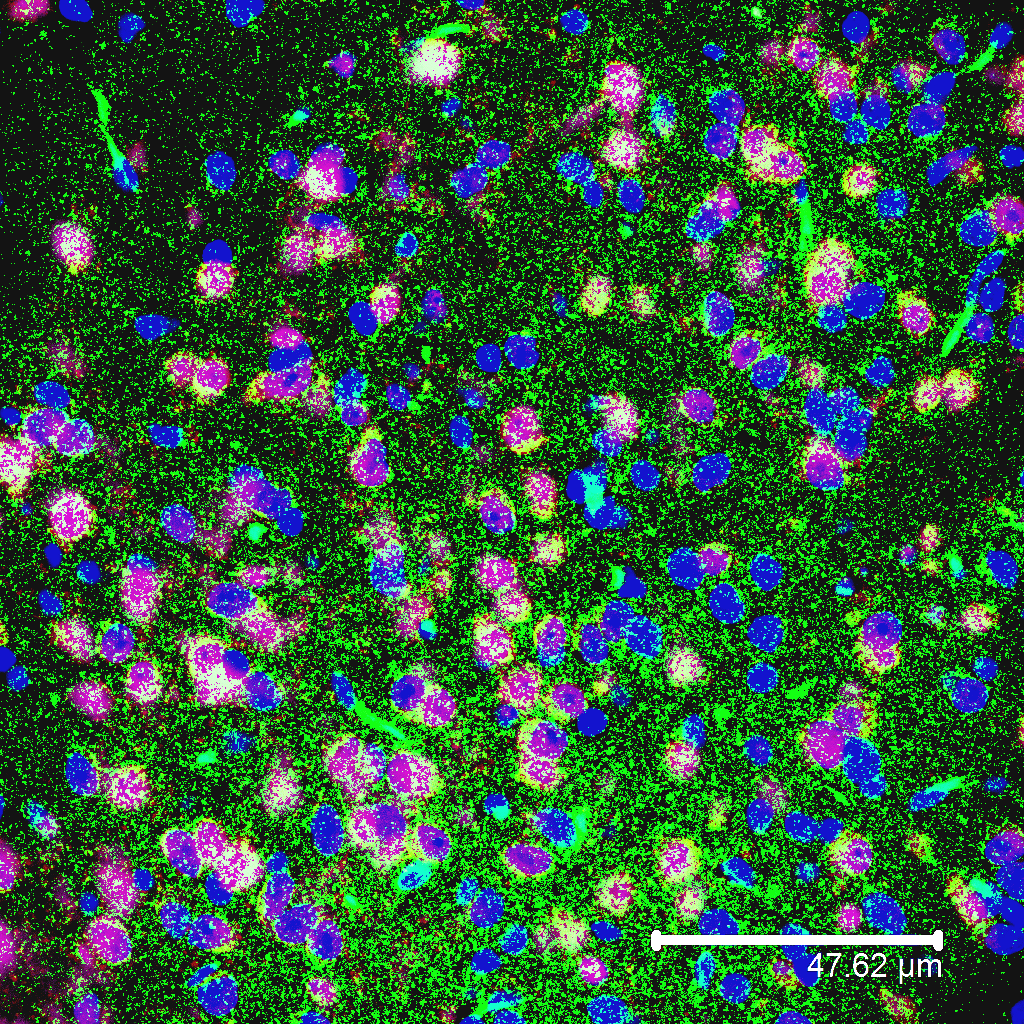** |
| **CCP** | **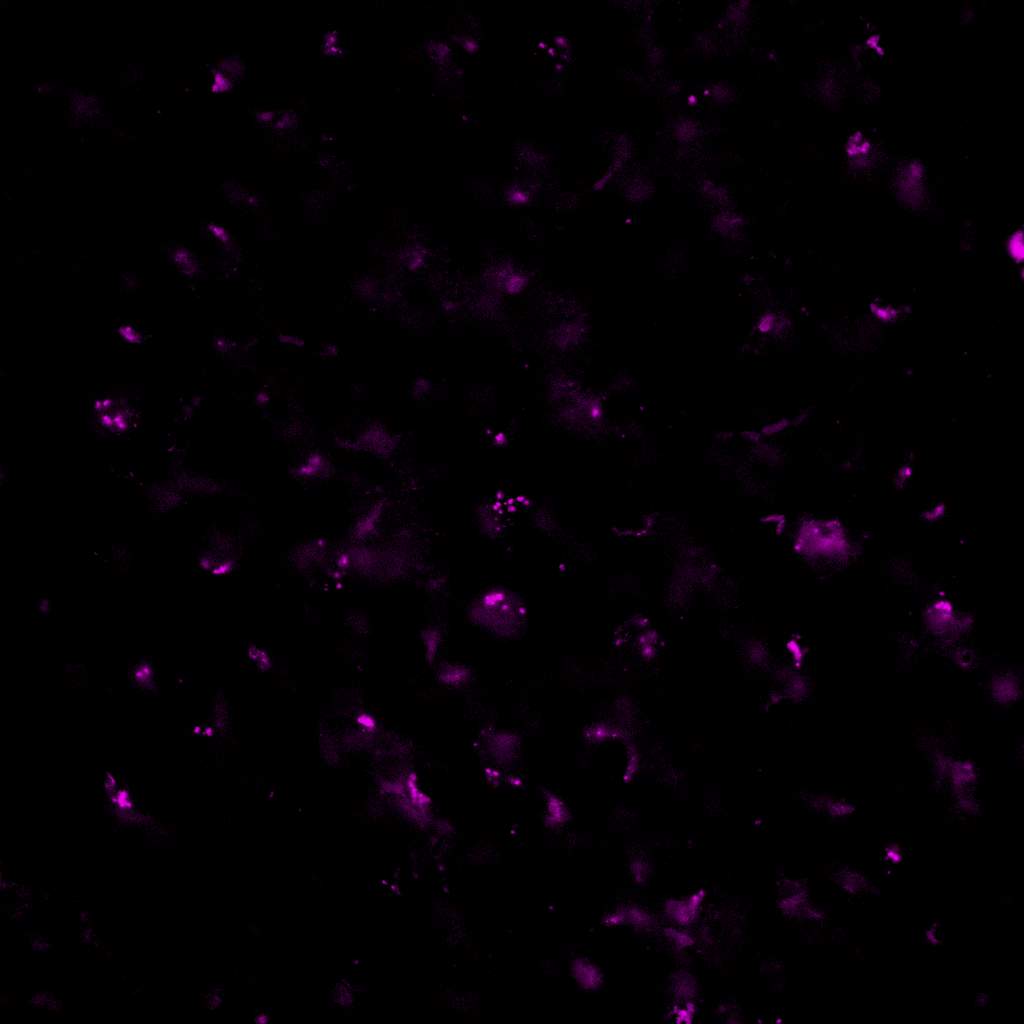** | **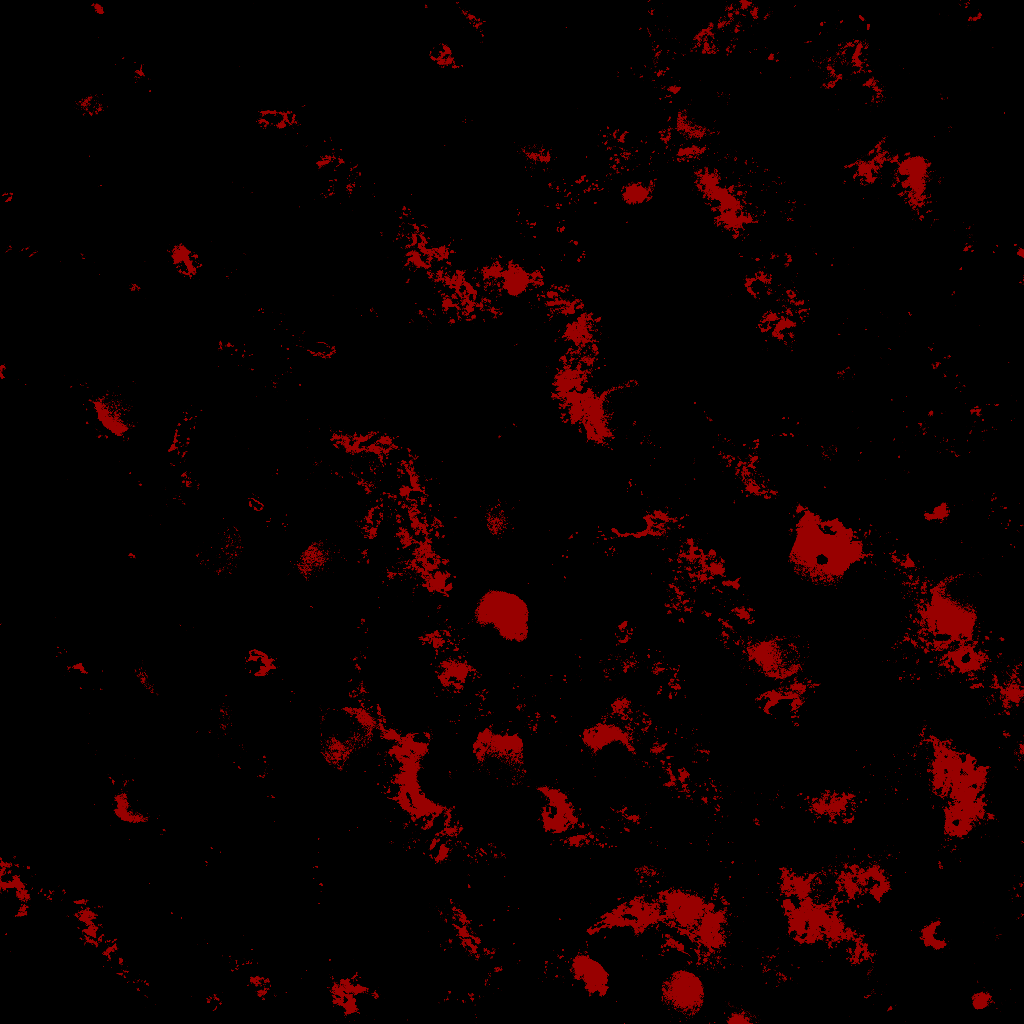** | | | **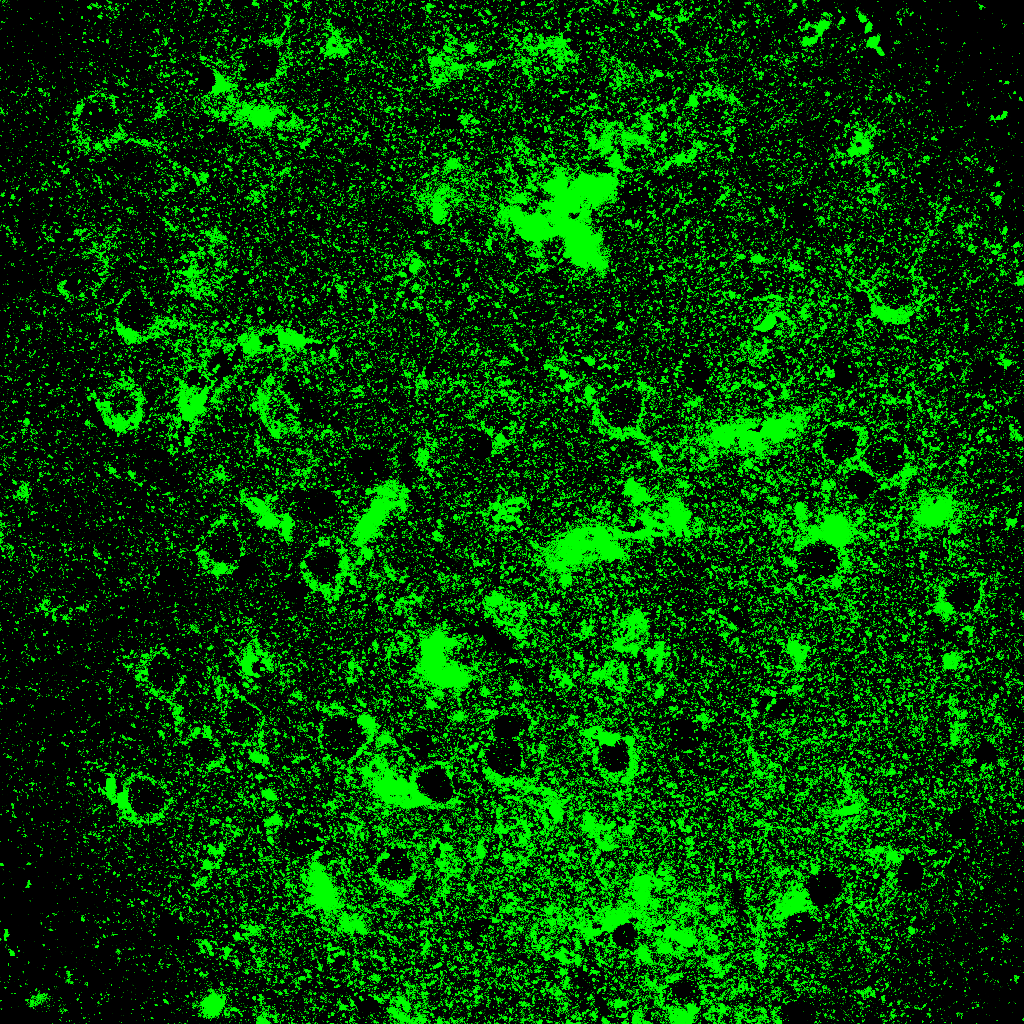** | **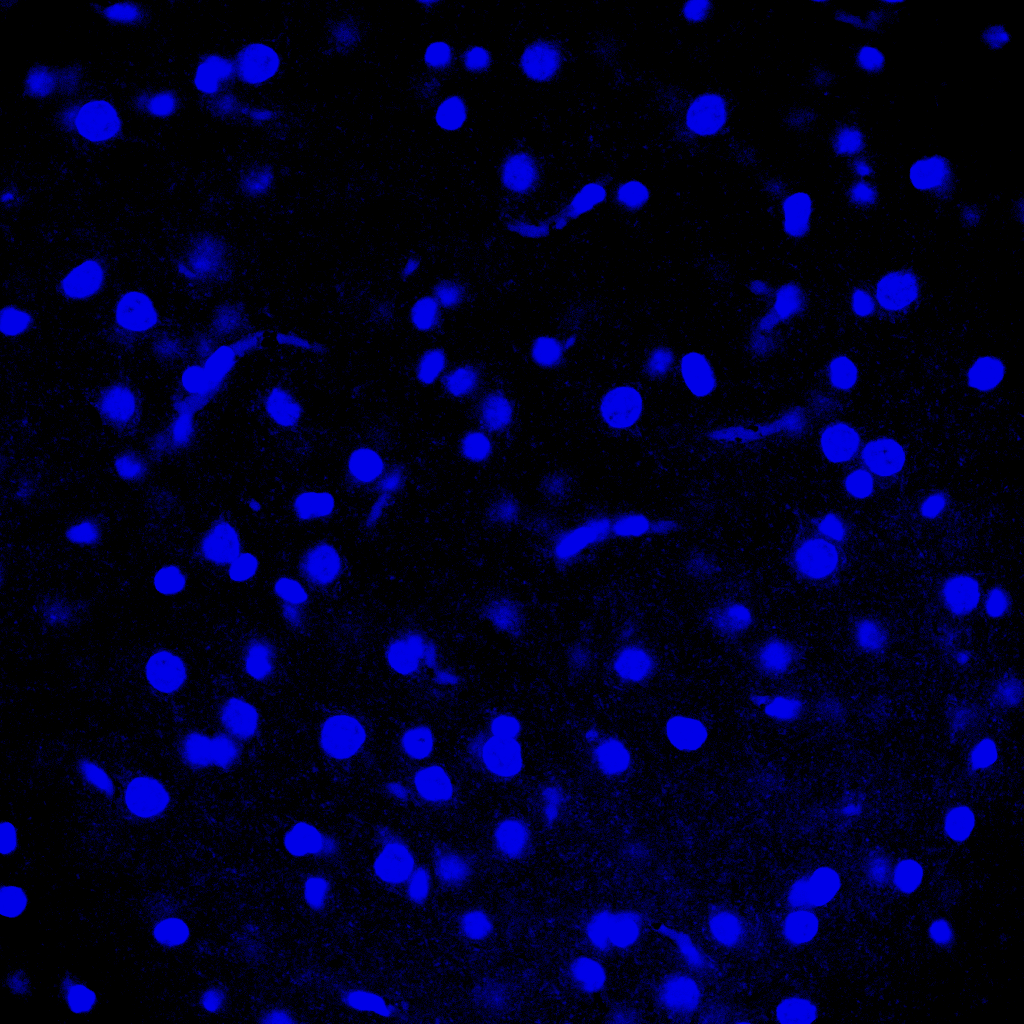** | | **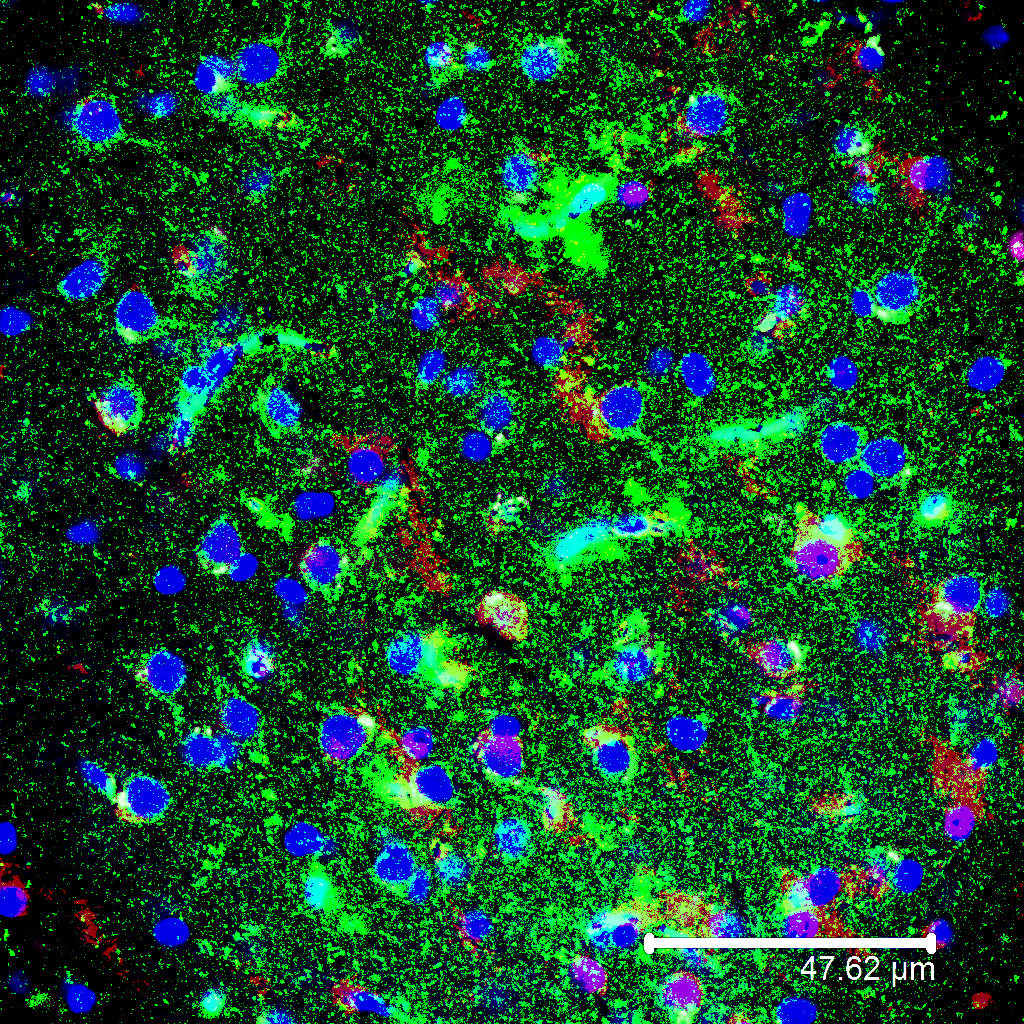** |
| **CCP+NK1.1Ab** | **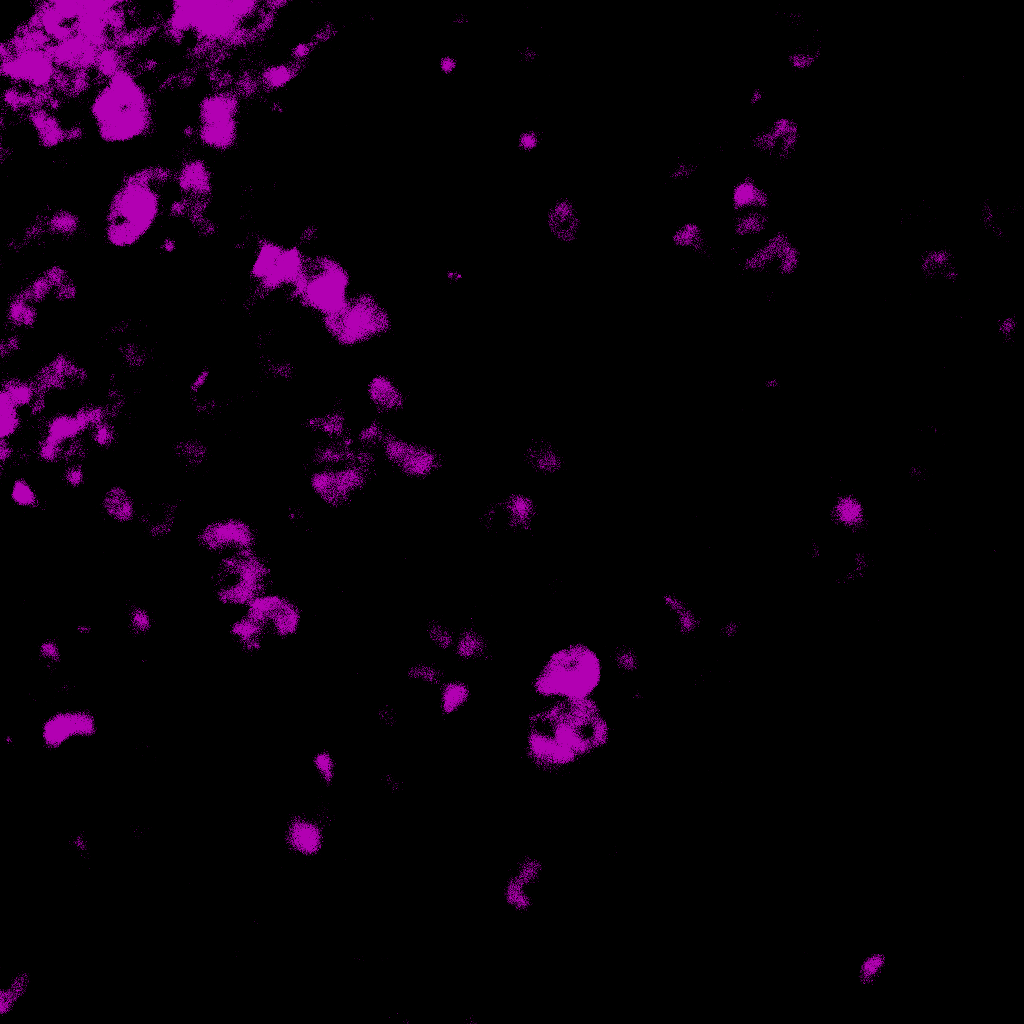** | **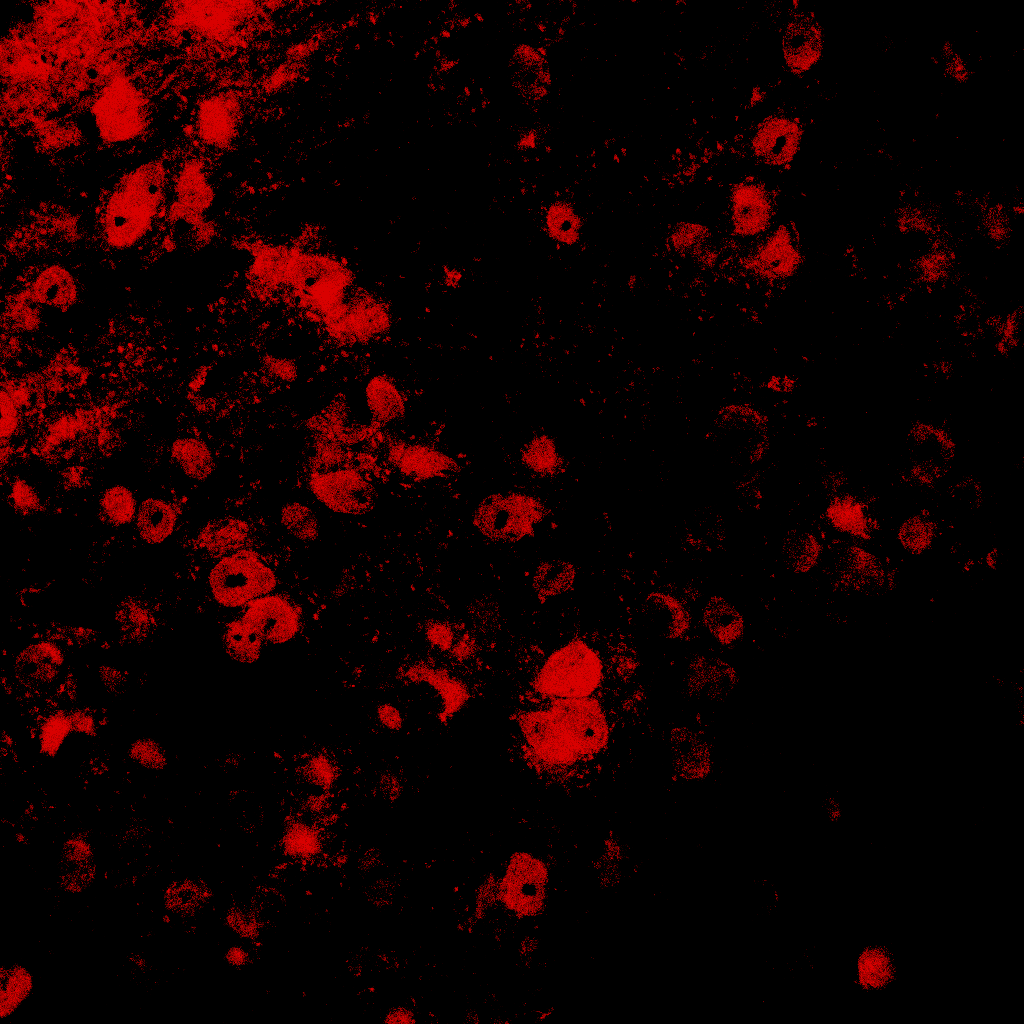** | | | **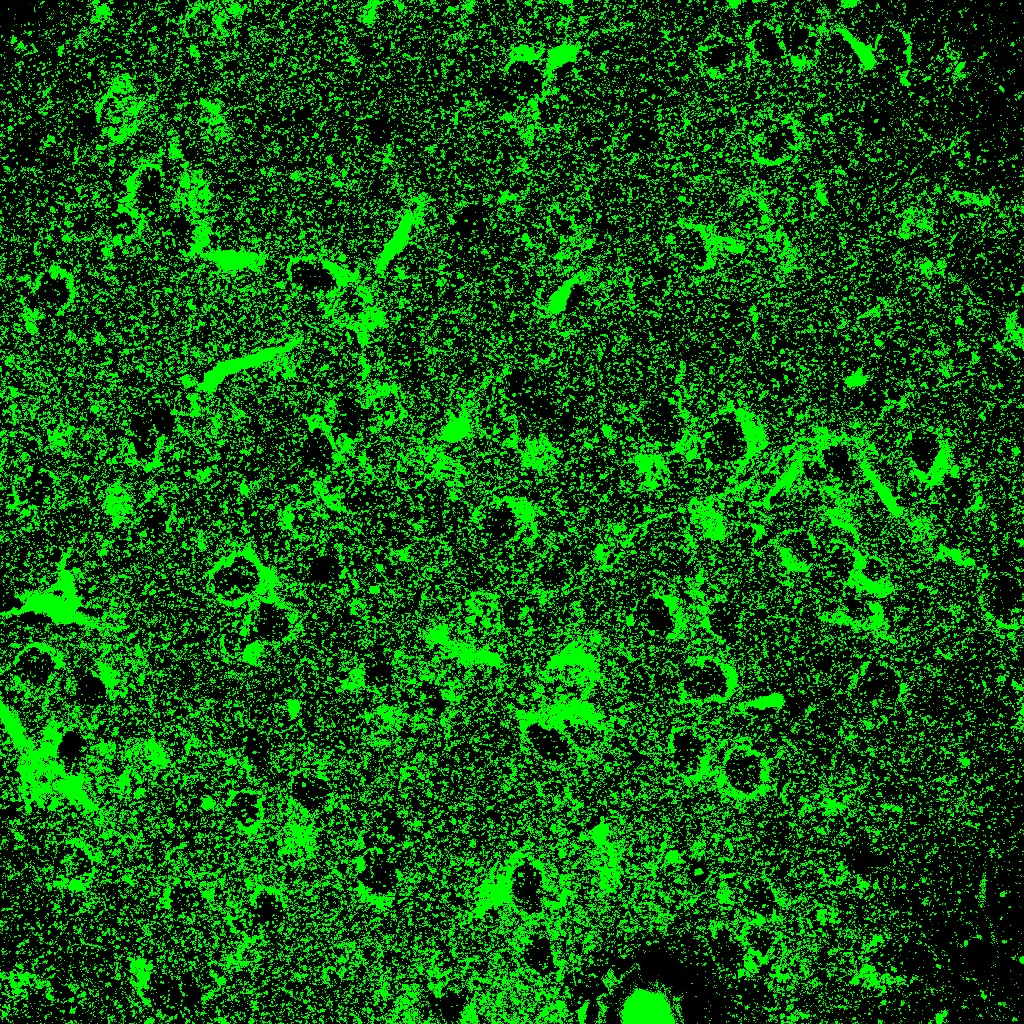** | **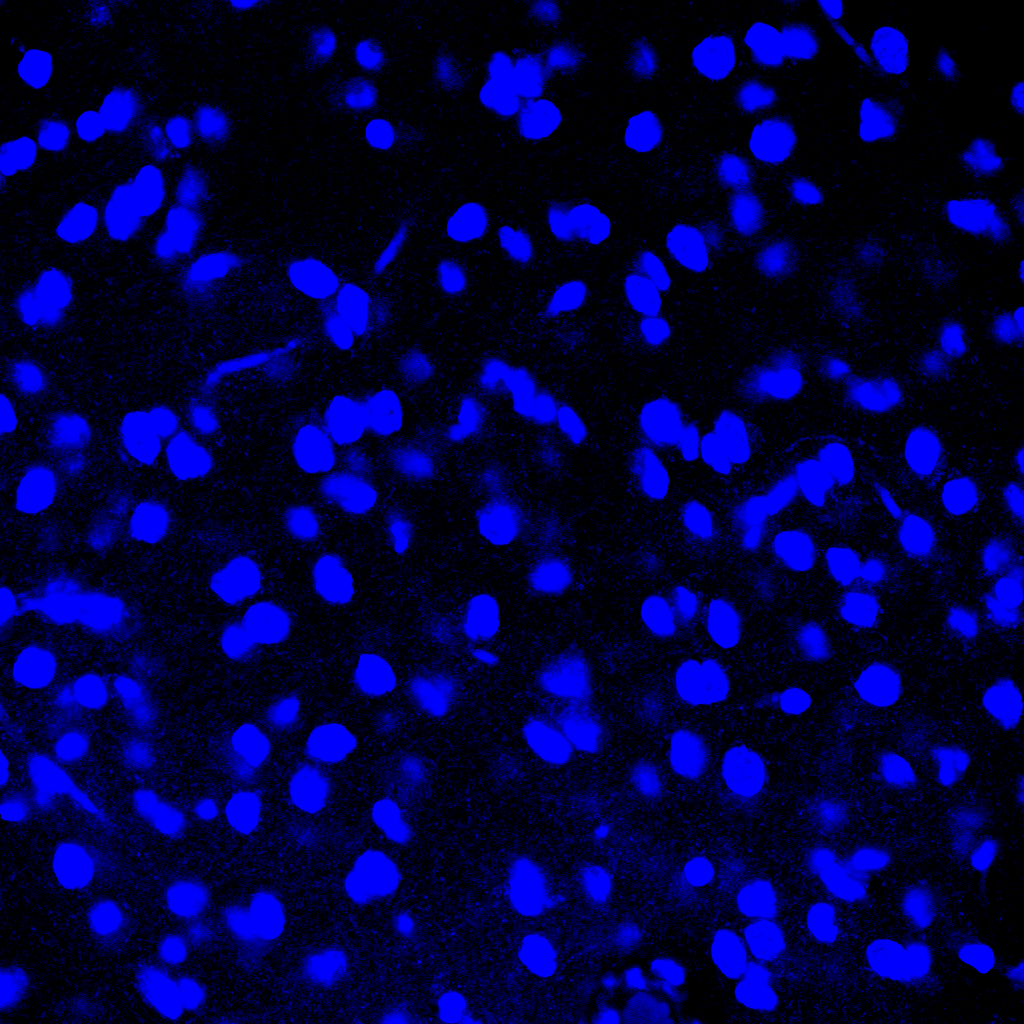** | | **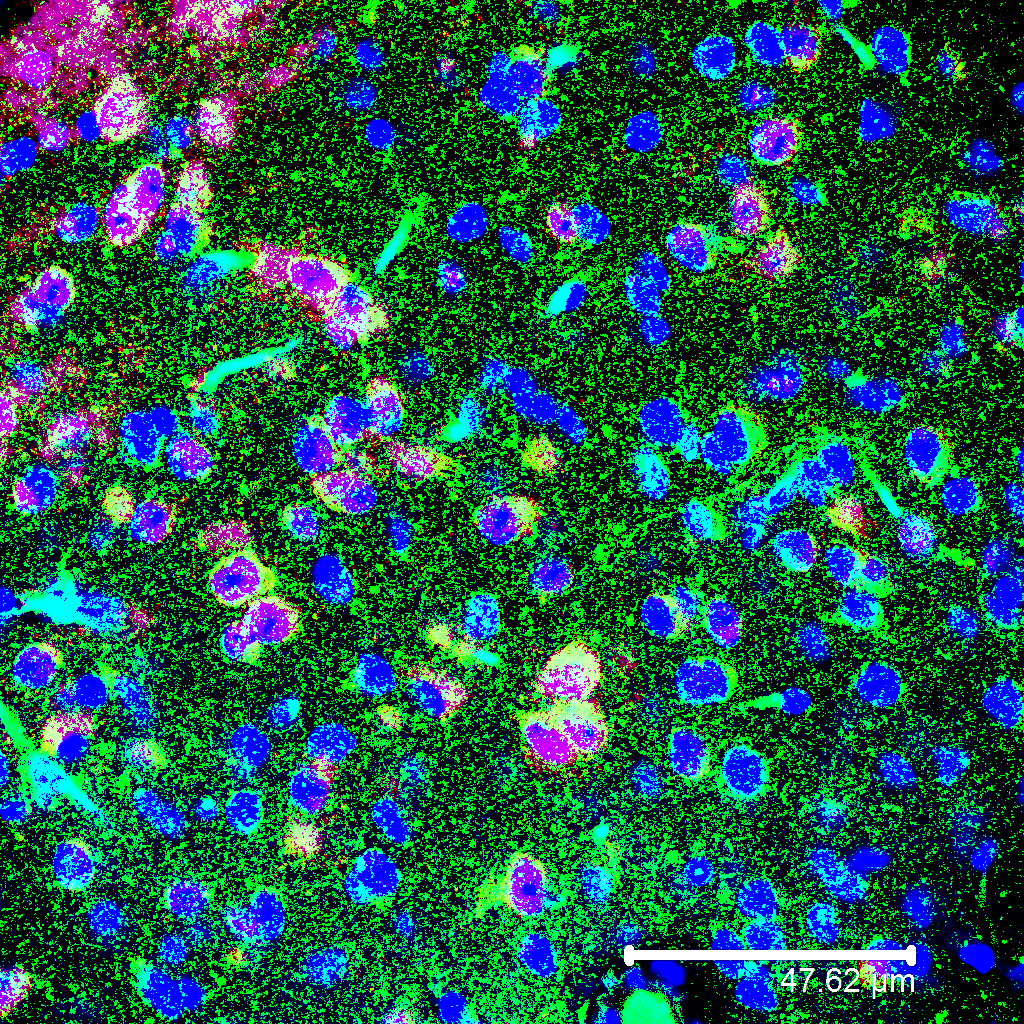** |
|  | **(B)**  **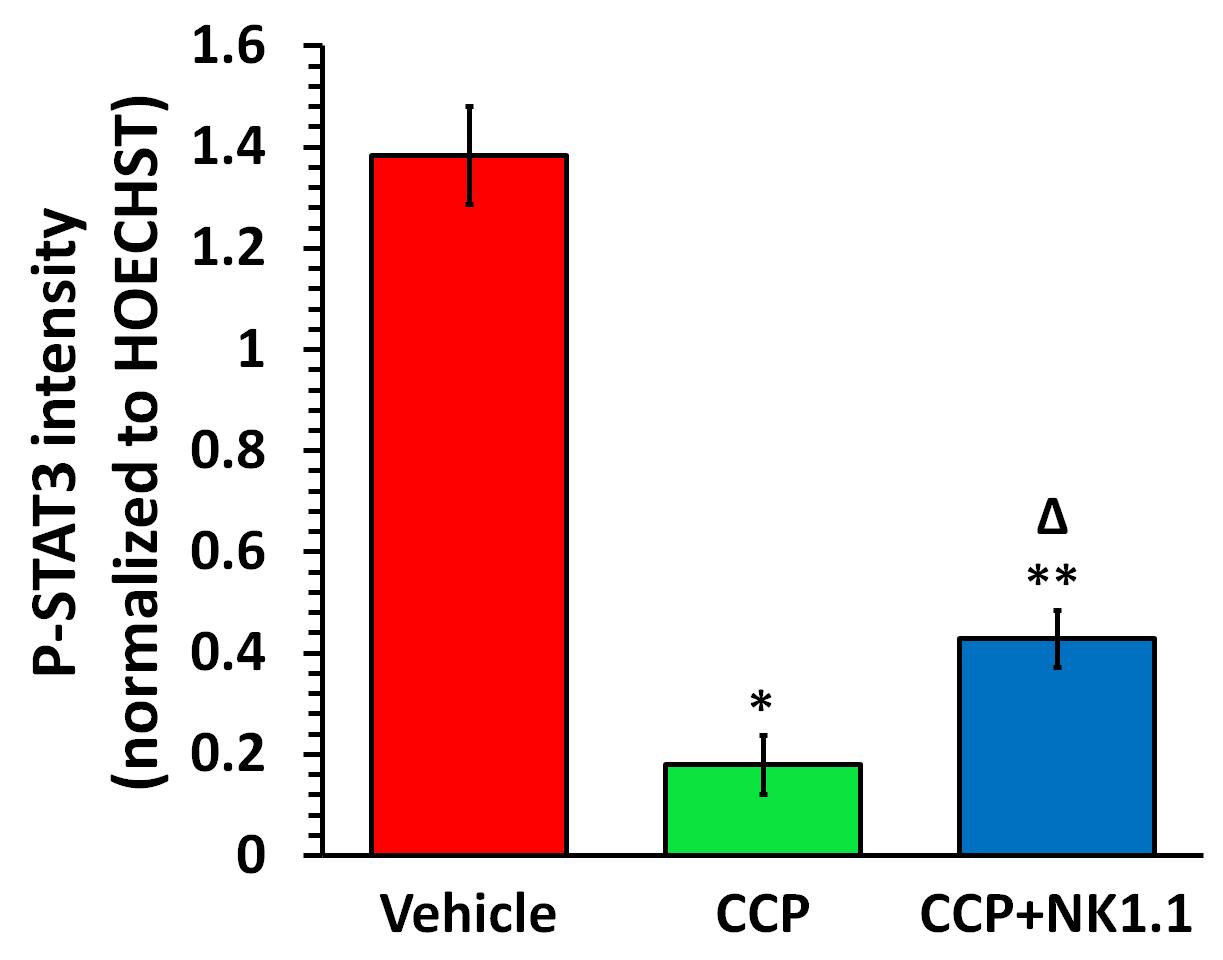** | | | **(C)**  **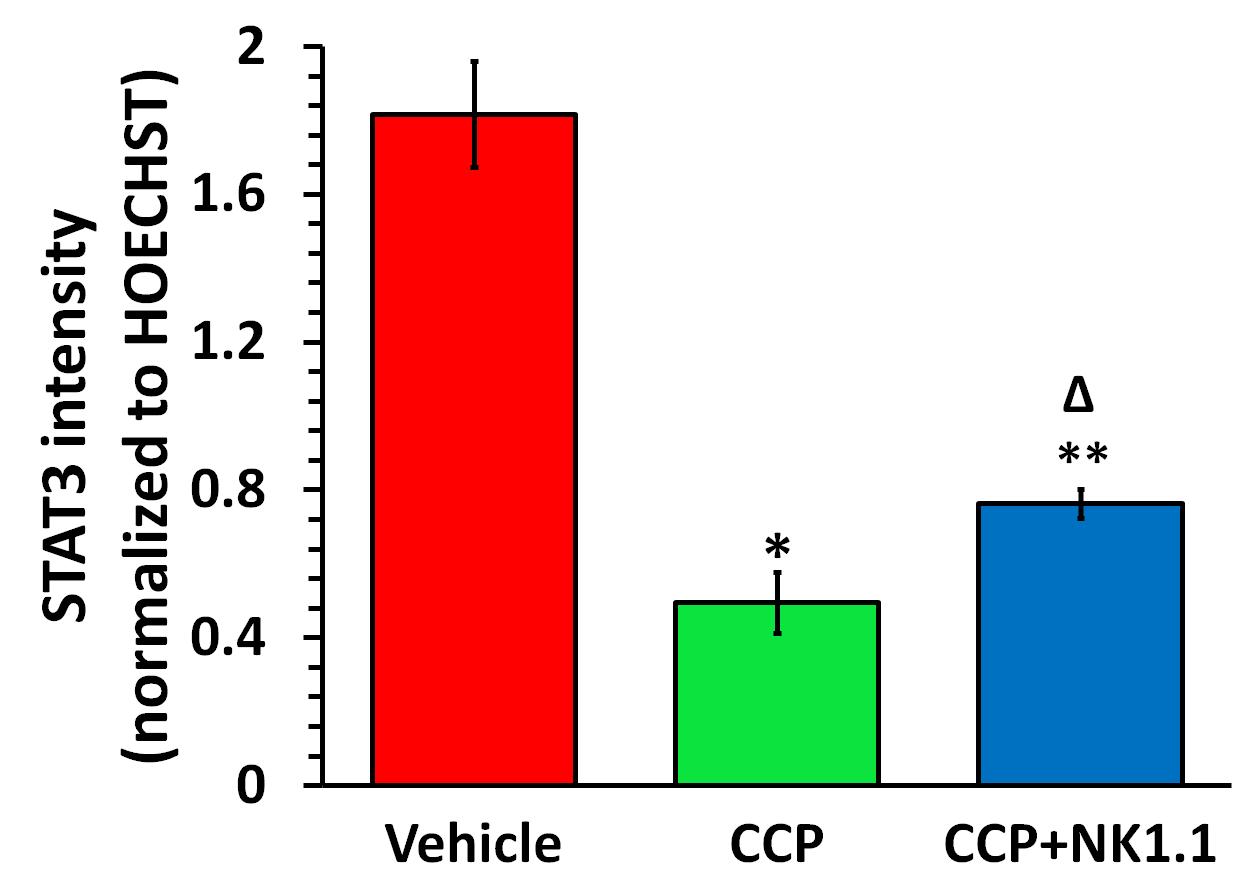** | | | **(D)**  **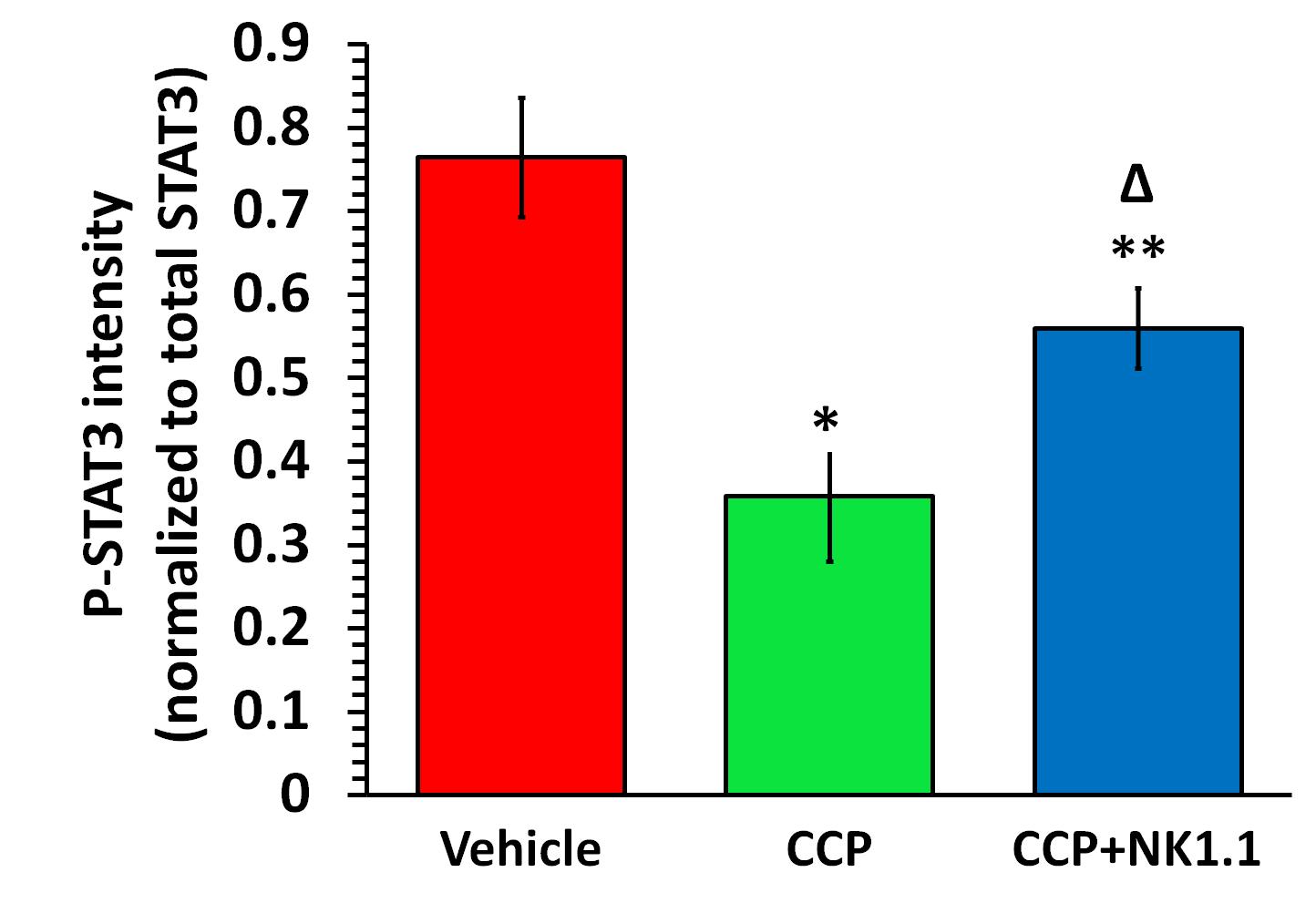** | |

**Additional file 5: Figure S5. Peripheral neutralization of NK cells by pre-injecting with the NK1.1 Ab partially reverses the CCP-mediated suppression of STAT3 in the TAM.** GBM Brain sections parallel to those used in Figure 3 from the three groups (Vehicle, CCP and CCP+NK1.1Ab) were used to assess the levels of STAT3 and activated STAT3 (P-Y^705^-STAT3) (P-STAT3) in the Iba1(+) TAM. **(A)** The GBM sections from the Vehicle-treated mice displayed high levels of STAT3 and P-STAT3 (top row), whereas the CCP-treated mice showed an 88.5% decrease in P-STAT3 (normalized to HOECHST) (*p = 5.1 x 10^-5^, CCP-treated versus Vehicle) and this CCP-evoked suppression was only by 61% in samples obtained from the CCP+NK1.1 mice (Δ p = 5.9 x 10^-3^, CCP+NK1.1 versus CCP-treated) **(B)**. The CCP-evoked 88.5% suppression of P-STAT3 in the TAM was the result of a 79% decrease in STAT3 (normalized to HOECHST) (only 68% in the CCP+NK1.1 mice) **(C)**, and a 68% decrease P-STAT3 (normalized to STAT3) (only 48% in CCP+NK1.1) (P-STAT3 normalized to STAT3) (**p = 1.2x10^-4^ Vehicle versus CCP+NK1.1) **(D)**. Three sections per mouse were used for imaging and the graphs represent mean ± S.D. obtained from Vehicle (n=4), CCP (n=4), and CCP+NK1.1 (n=4). (Scale bar: 47.62 µm.).
