# Supplementary material for: Phytosomal curcumin causes natural killer cell-dependent repolarization of glioblastoma (GBM) tumor-associated microglia/macrophages and elimination of GBM and GBM stem cells
Source: J Exp Clin Cancer Res. 2018 Jul 25;37:168. doi: 10.1186/s13046-018-0792-5 (PMC6058381; doi:10.1186/s13046-018-0792-5)
Supplement: Supplementary file 6 — Figure S6. Peripheral neutralization of NK cells partially reverses the CCP-mediated induction and activation of STAT1 in the TAM. GBM Brain sections parallel to those used in Fig. S5 from the three groups (Vehicle, CCP and CCP + NK1.1Ab) were used to evaluate the levels of STAT1 and activated STAT1 (Tyr701-STAT1) (P-STAT1) in the Iba1(+) TAM. (A) The Vehicle-treated mice showed low levels of STAT1 (red) and P-STAT1 (purple) in the Iba1(+) (green) cells (First row and B), but a 1286% overall increase in P-STAT1 was observed in the CCP-treated GBM sections (Second row and B) (*p = 2.3 × 10− 4, Vehicle versus CCP). This CCP-evoked increase in P-STAT1 was only 300% in the CCP + NK1.1 mouse samples (Third row and B) (Δ p = 0.04, CCP versus CCP + NK1.1). The CCP-evoked increase in P-STAT1 was the result of a 300% induction in STAT1 (only 194% increase in the CCP + NK1.1 sections) (A, C), and a 423% augmentation of P-STAT1 with respect to STAT1 (activation) (only 206% activation in the CCP + NK1.1 sections) (**p = 2.9 × 10− 4, Vehicle versus CCP + NK1.1) (A, D). Three sections per mouse were used for imaging and the graphs represents mean ± S.D. obtained from mice treated with Vehicle (n = 4), CCP (n = 4), and CCP + NK1.1 (n = 3). (Scale bar: 47.62 μm). (DOC 113 kb) [file 13046_2018_792_MOESM6_ESM.doc]

| **(A)** | **P-STAT1** | **STAT1** | | **Iba1** | | | **HOECHST** | | | **Merged** | |
| --- | --- | --- | --- | --- | --- | --- | --- | --- | --- | --- | --- |
| **Vehicle** | **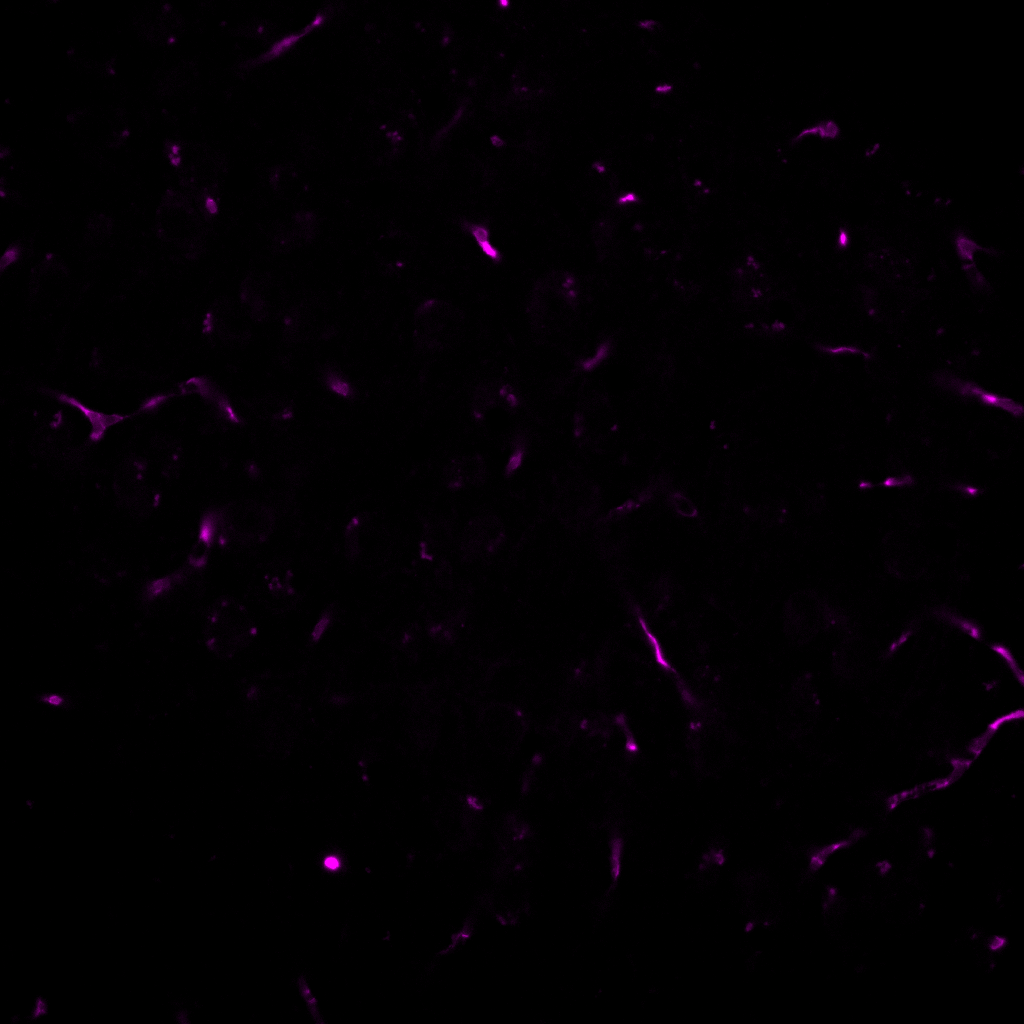** | **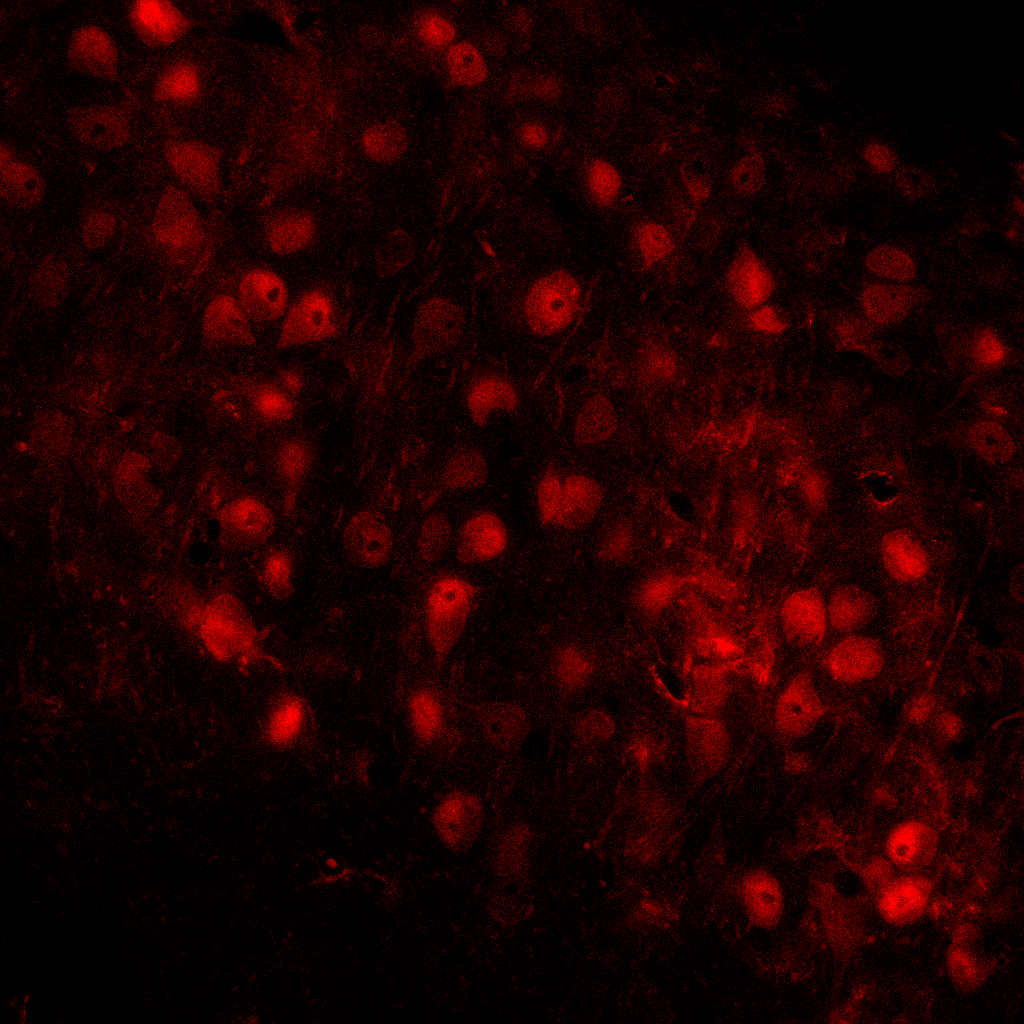** | | | **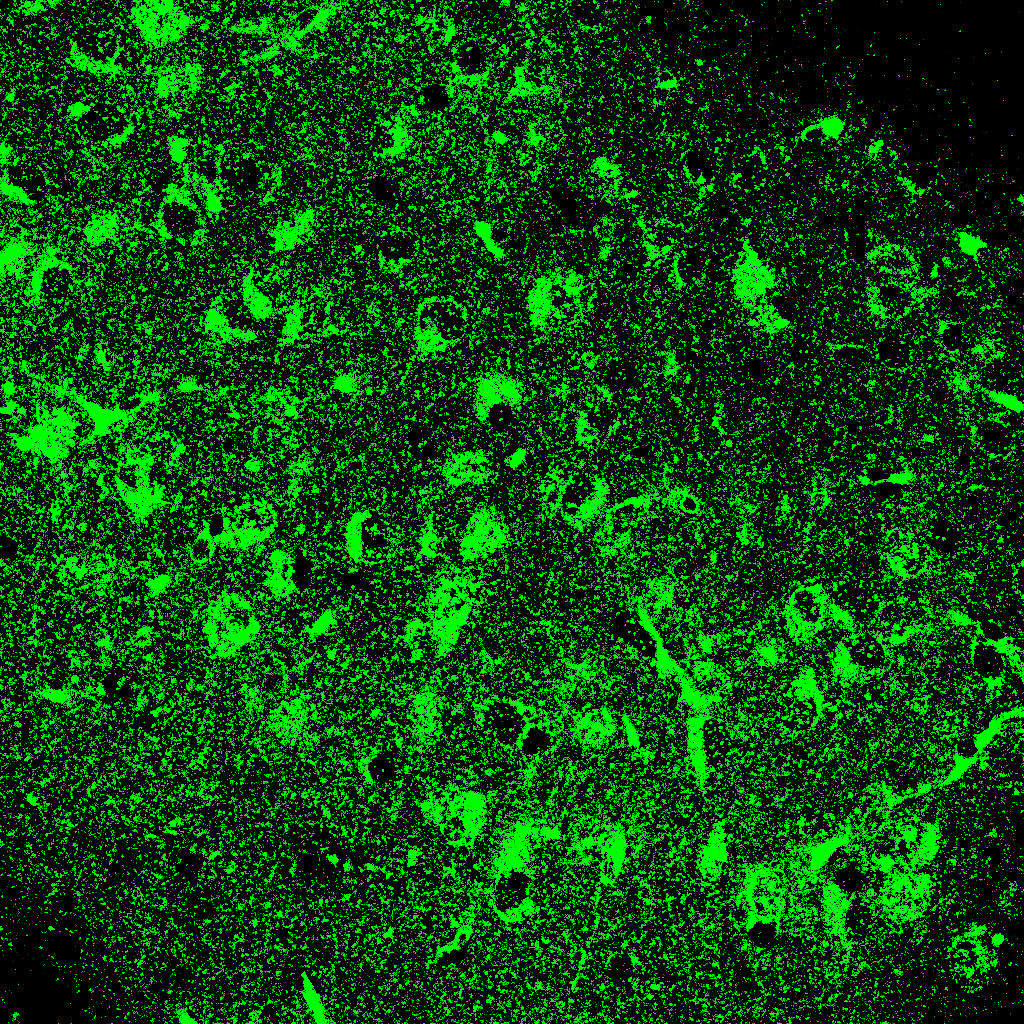** | | | **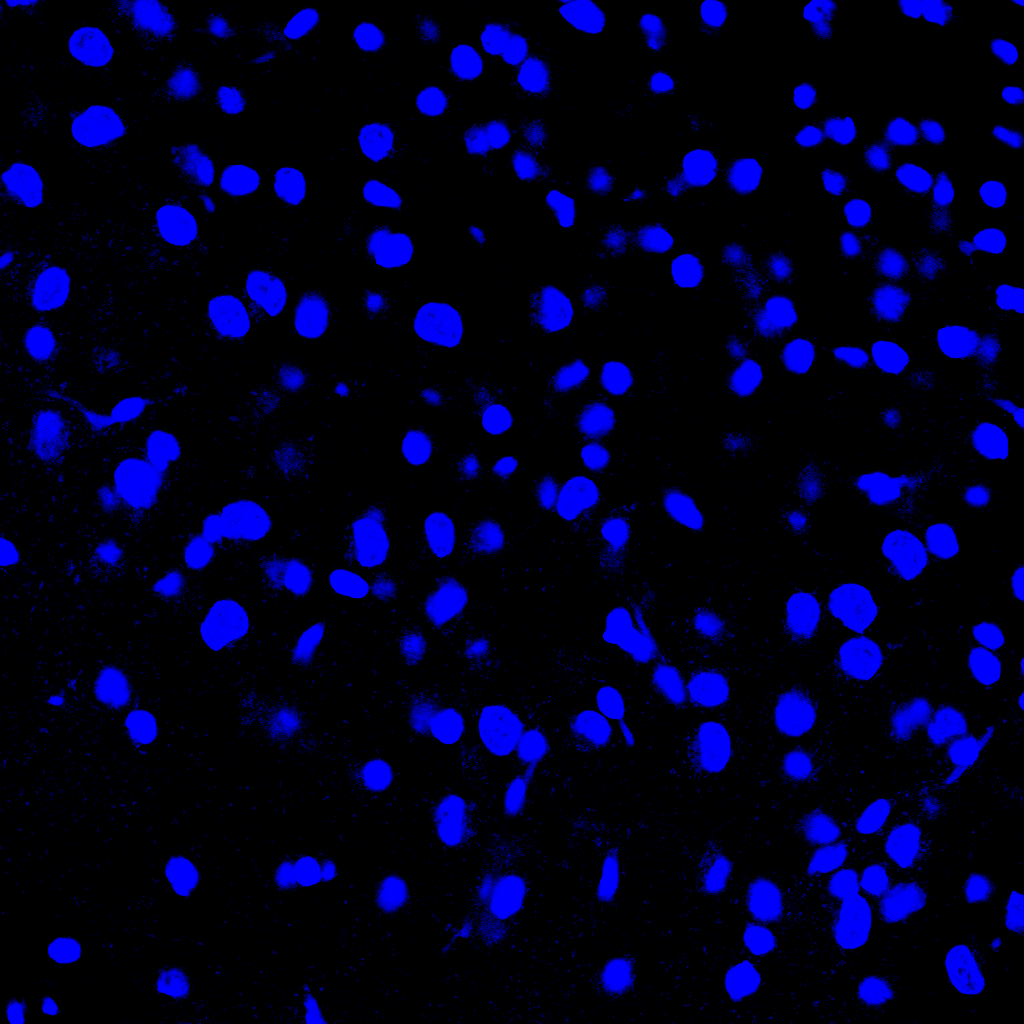** | | | **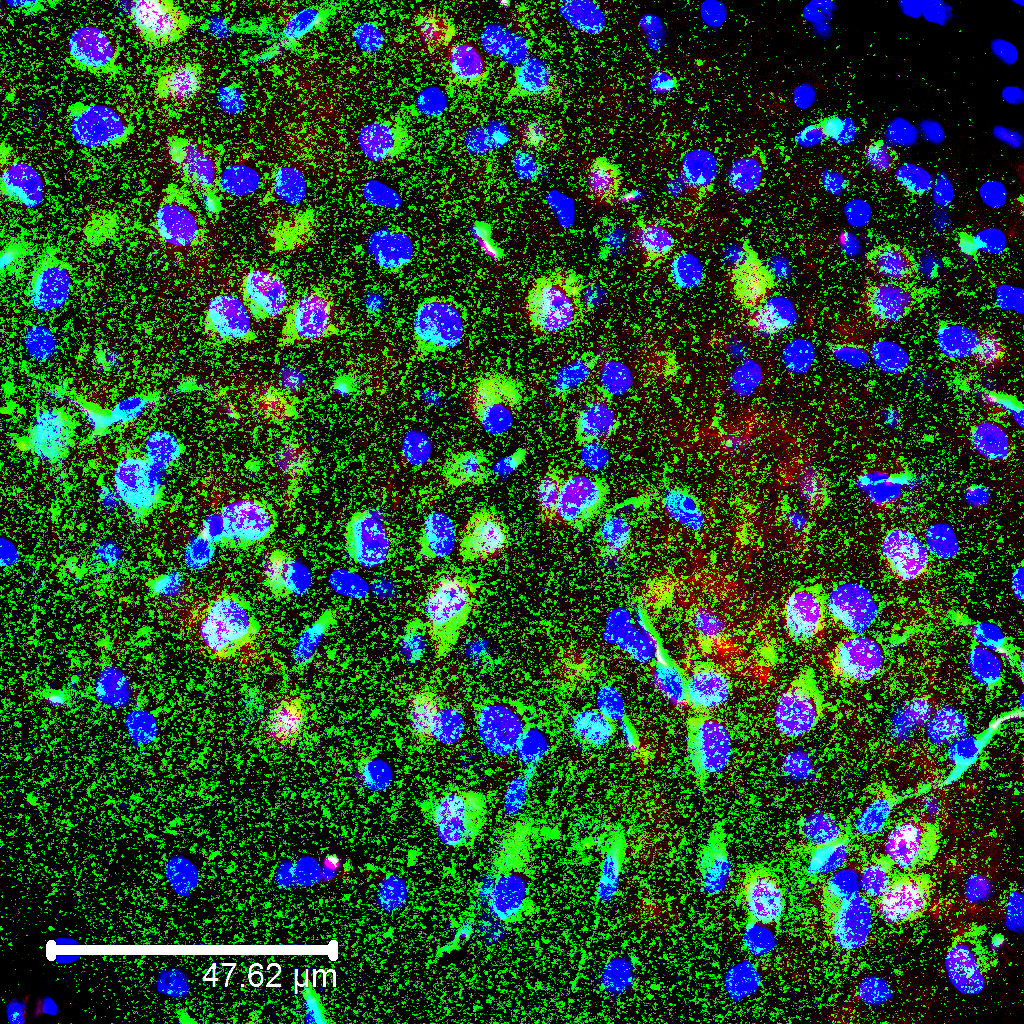** |
| **CCP** | **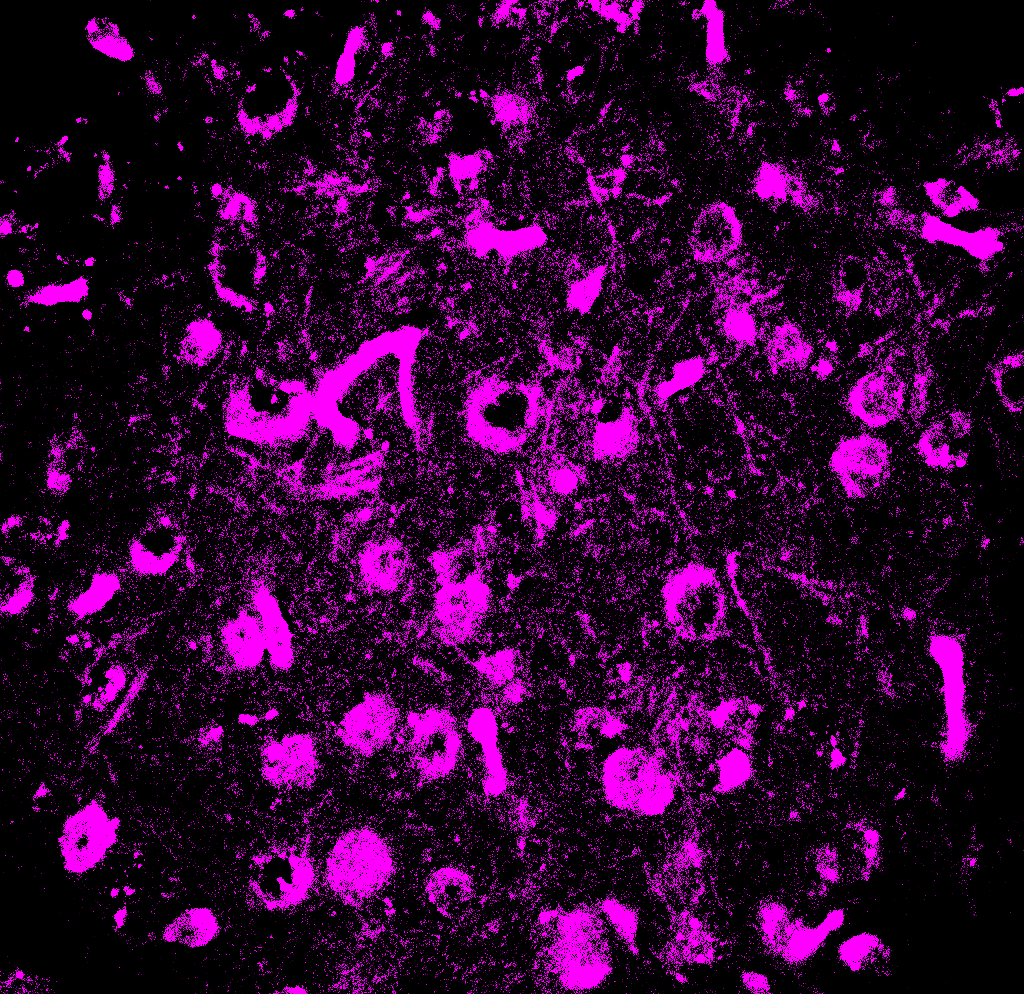** | **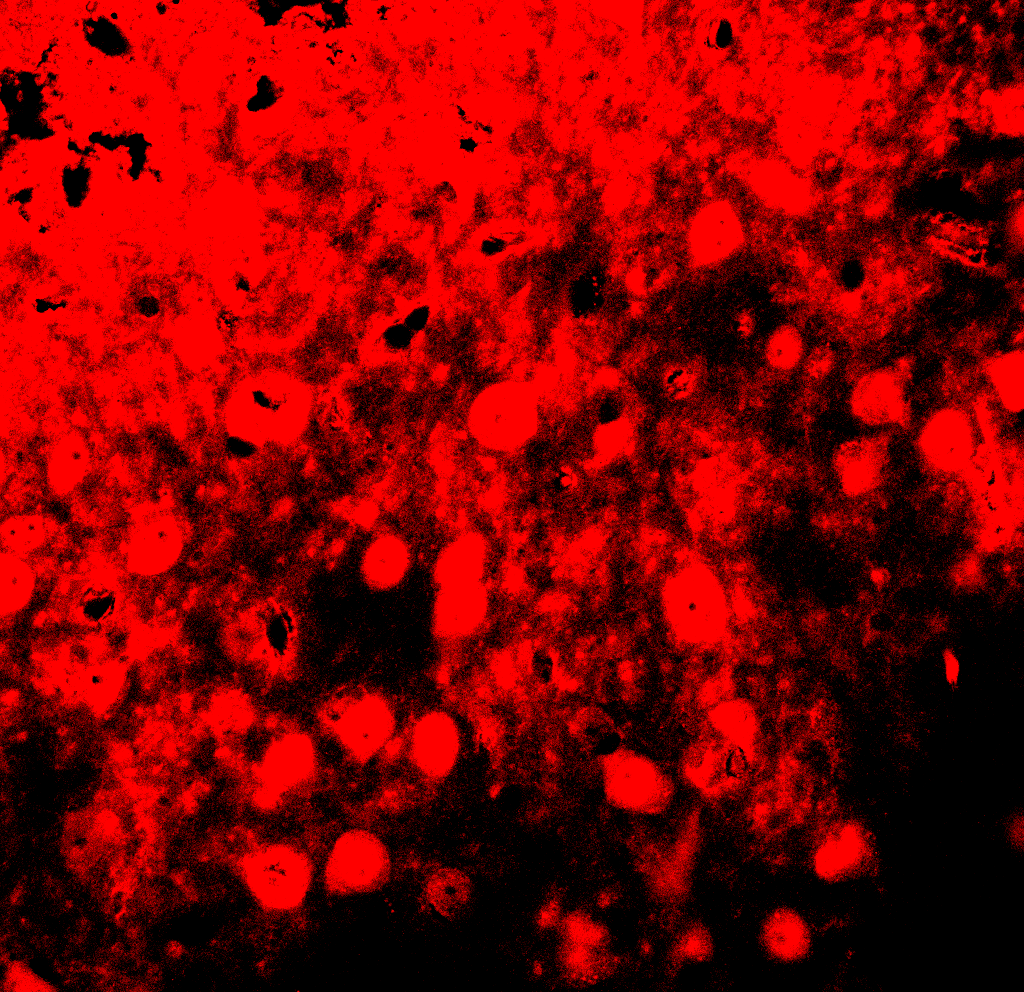** | | | **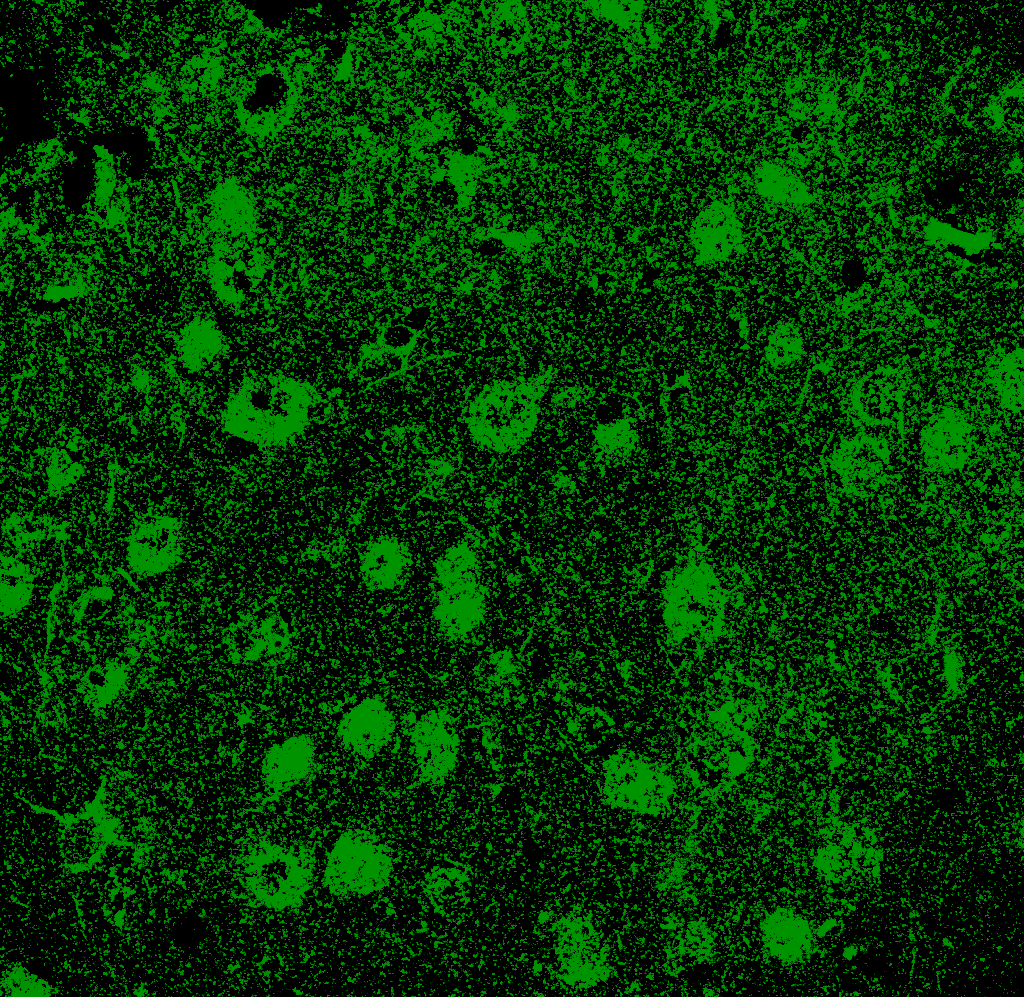** | | | **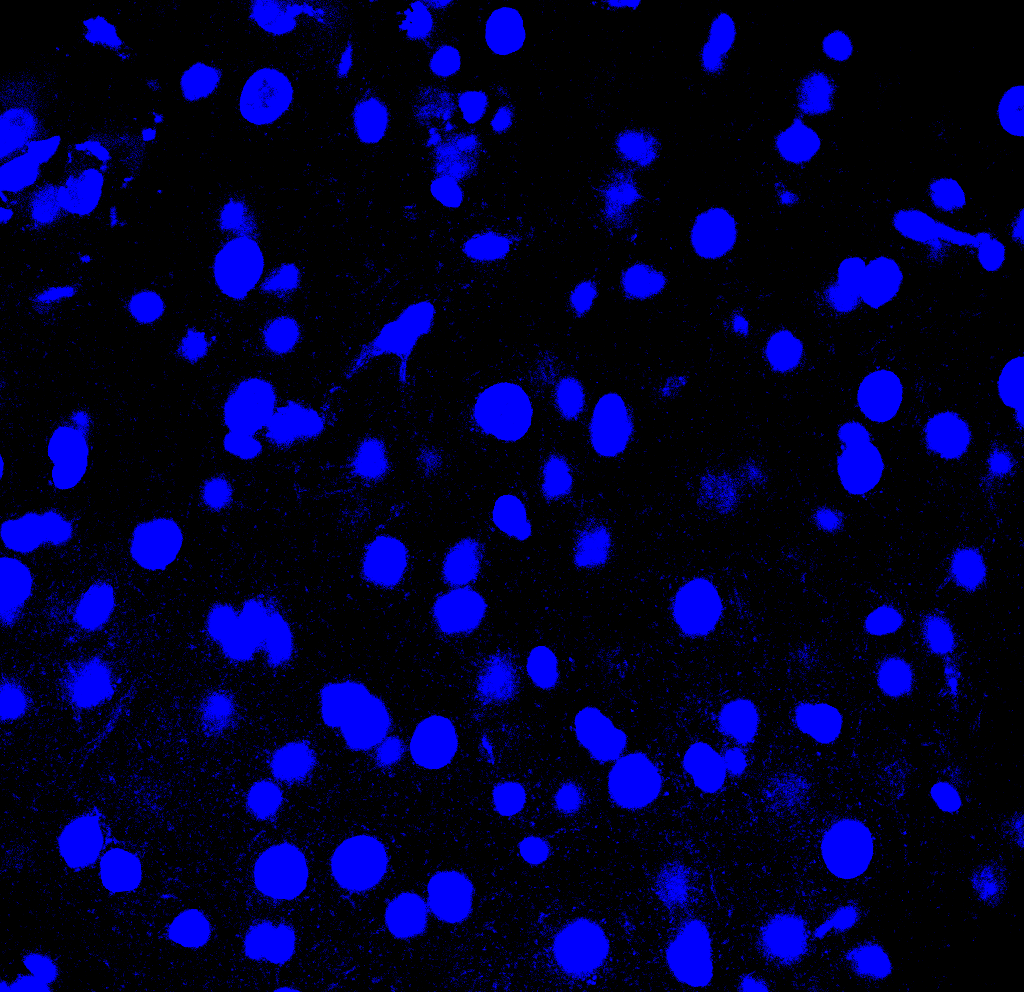** | | | **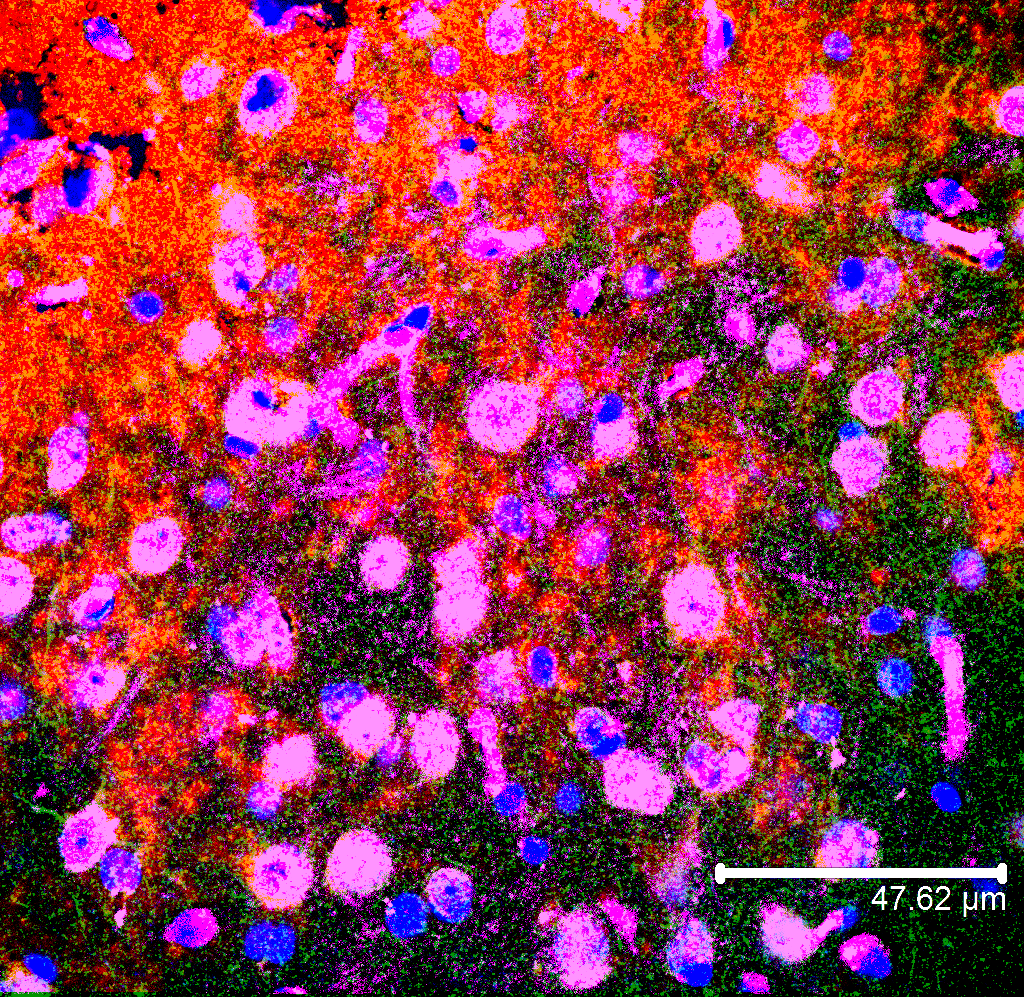** |
| **CCP+NK 1.1Ab** | **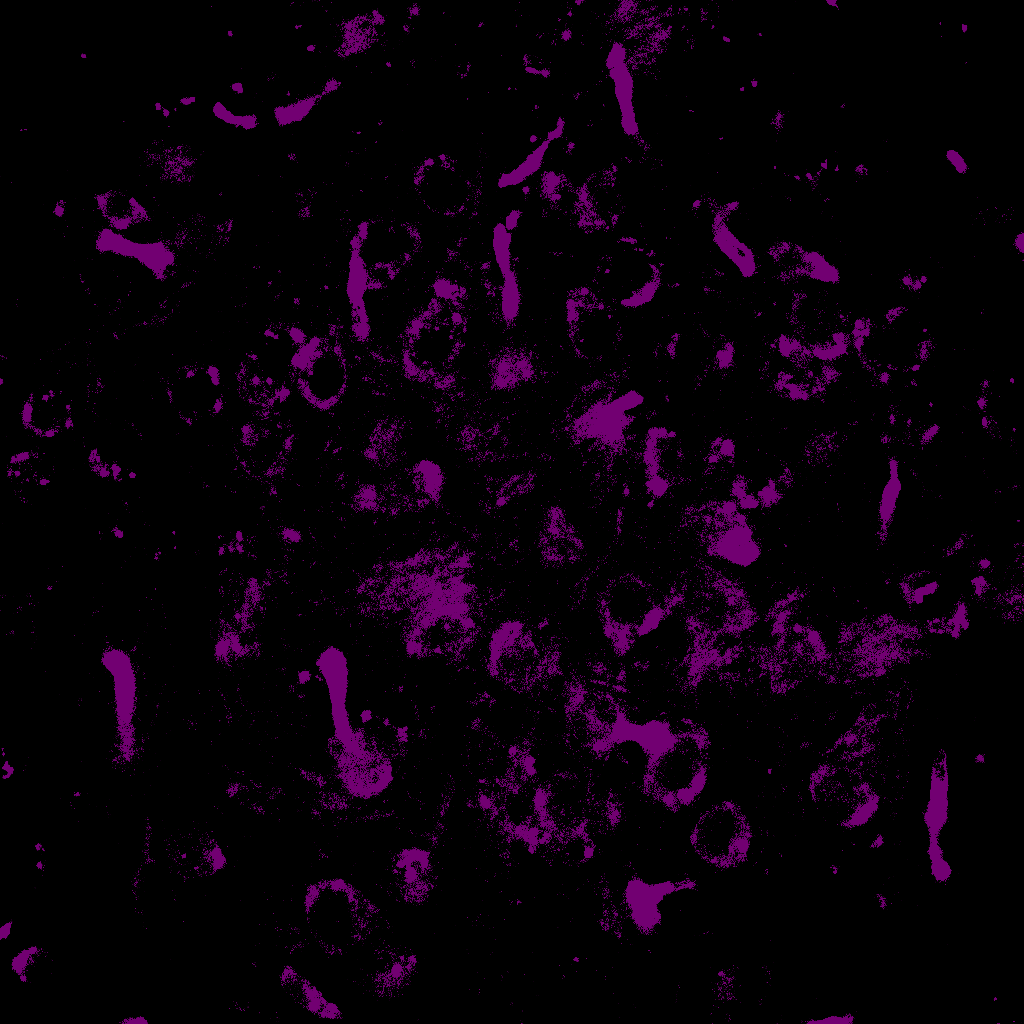** | **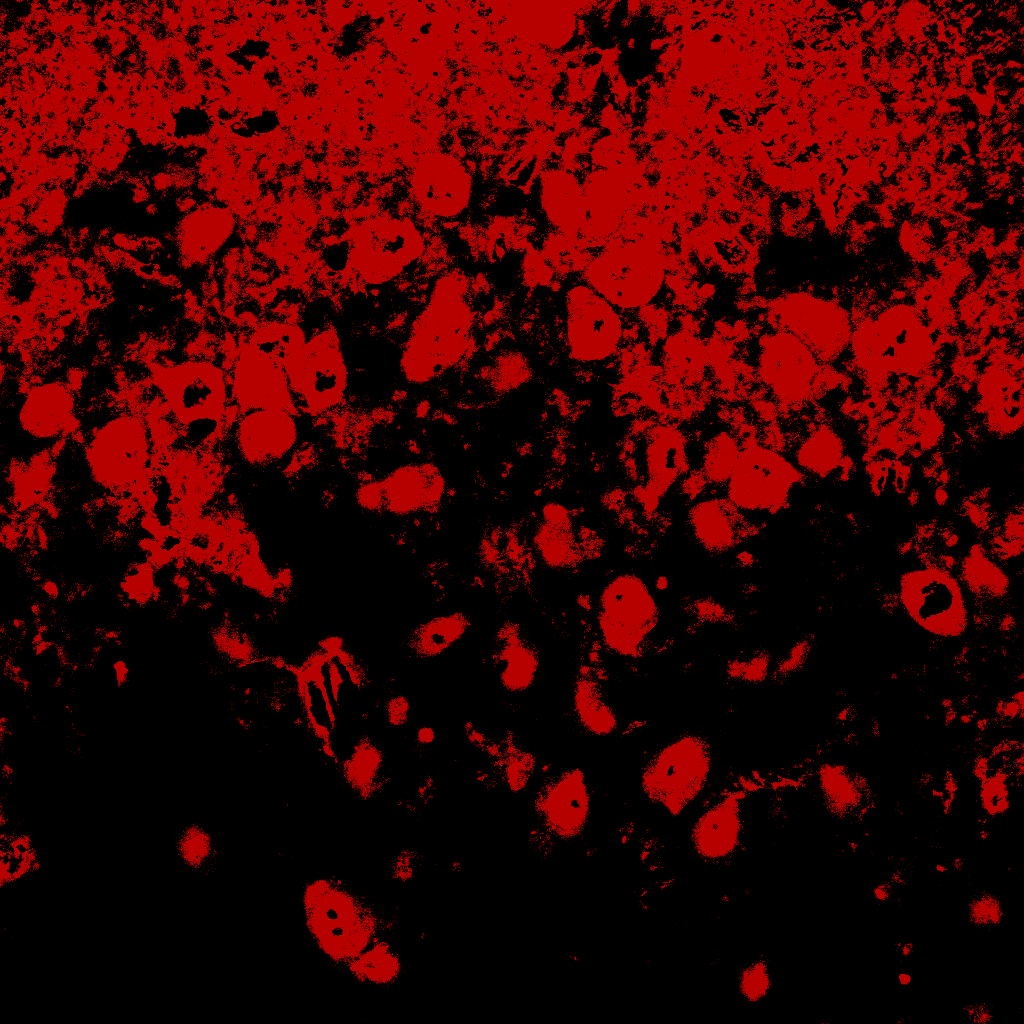** | | | | **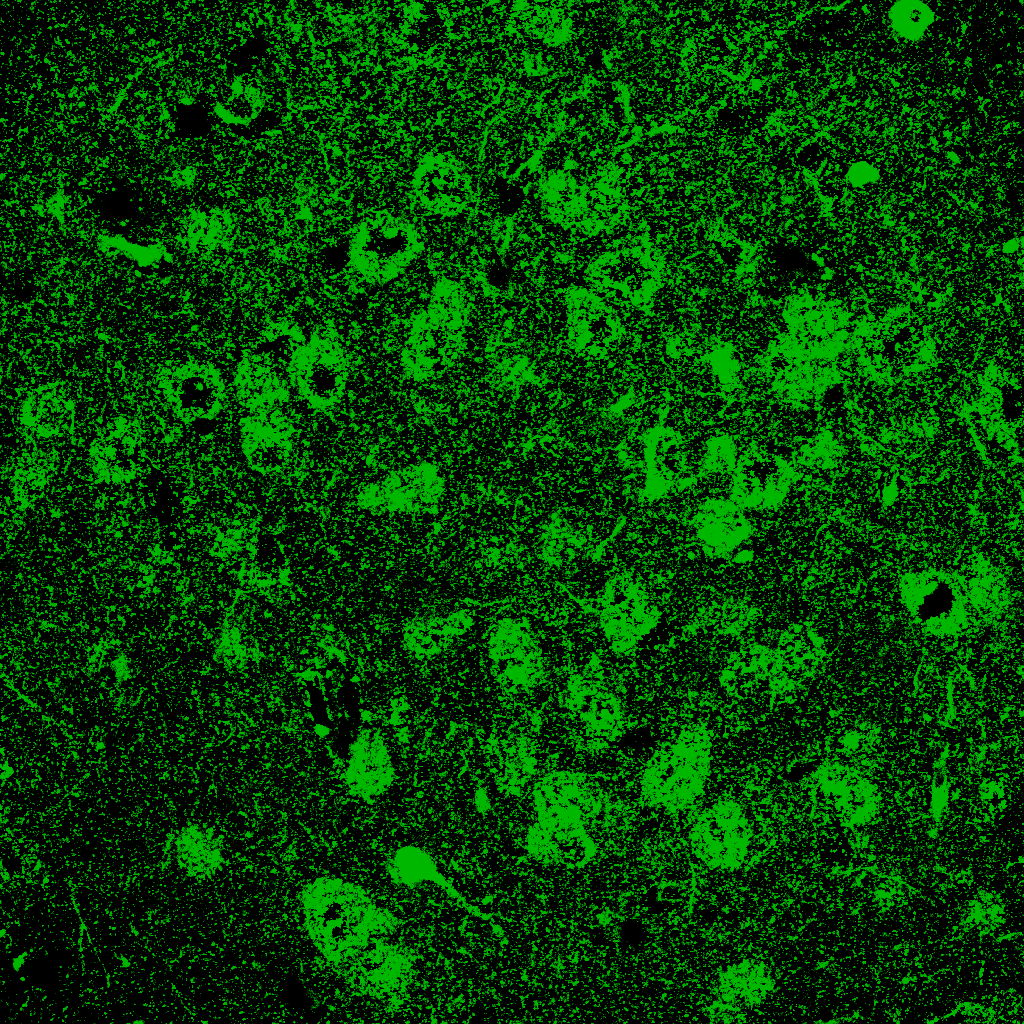** | | **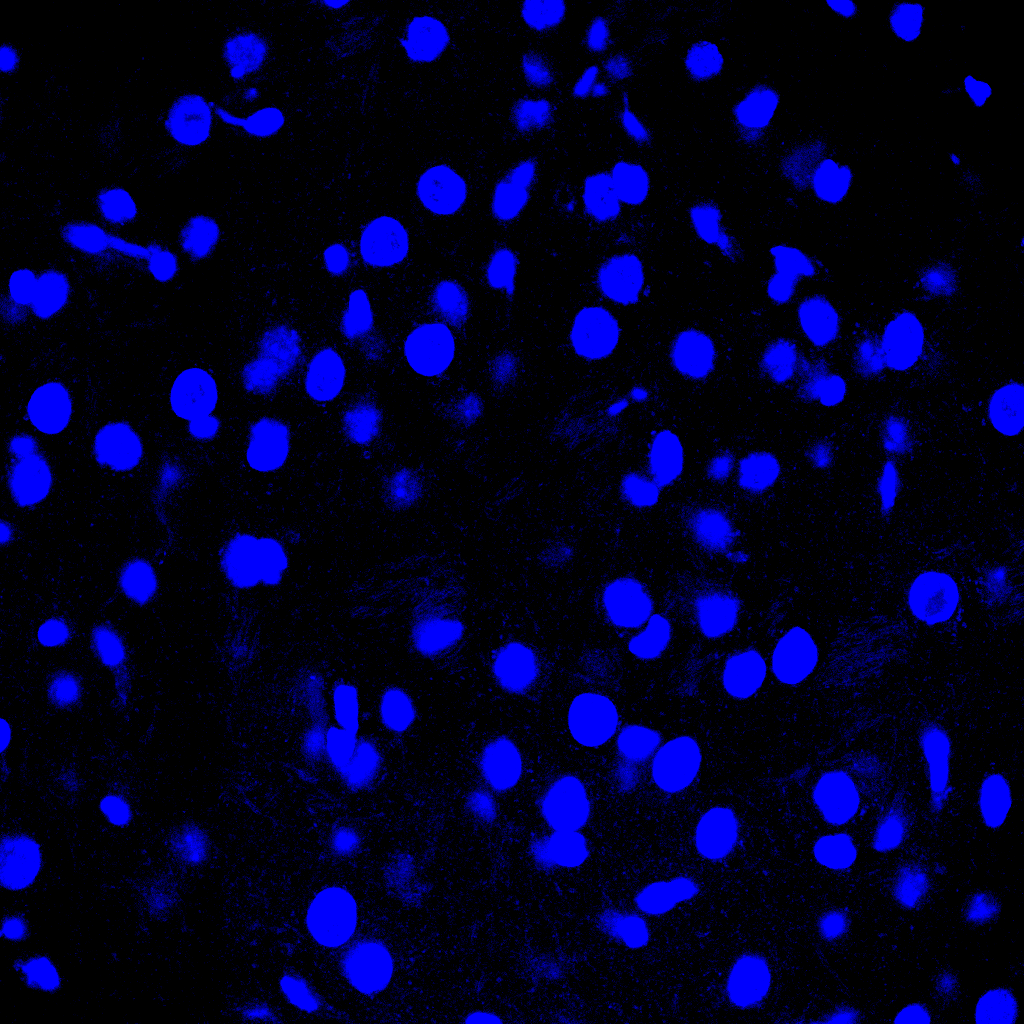** | | | **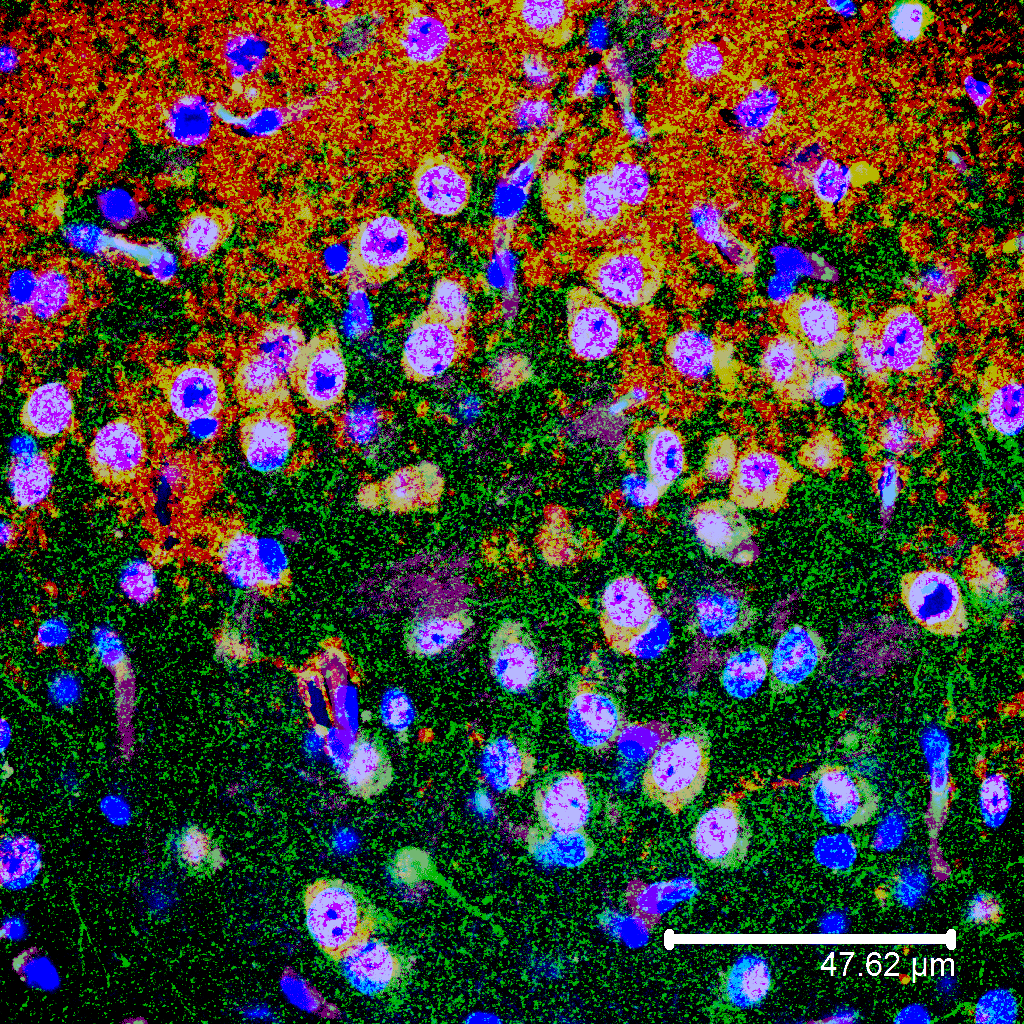** |
| **(B)**  **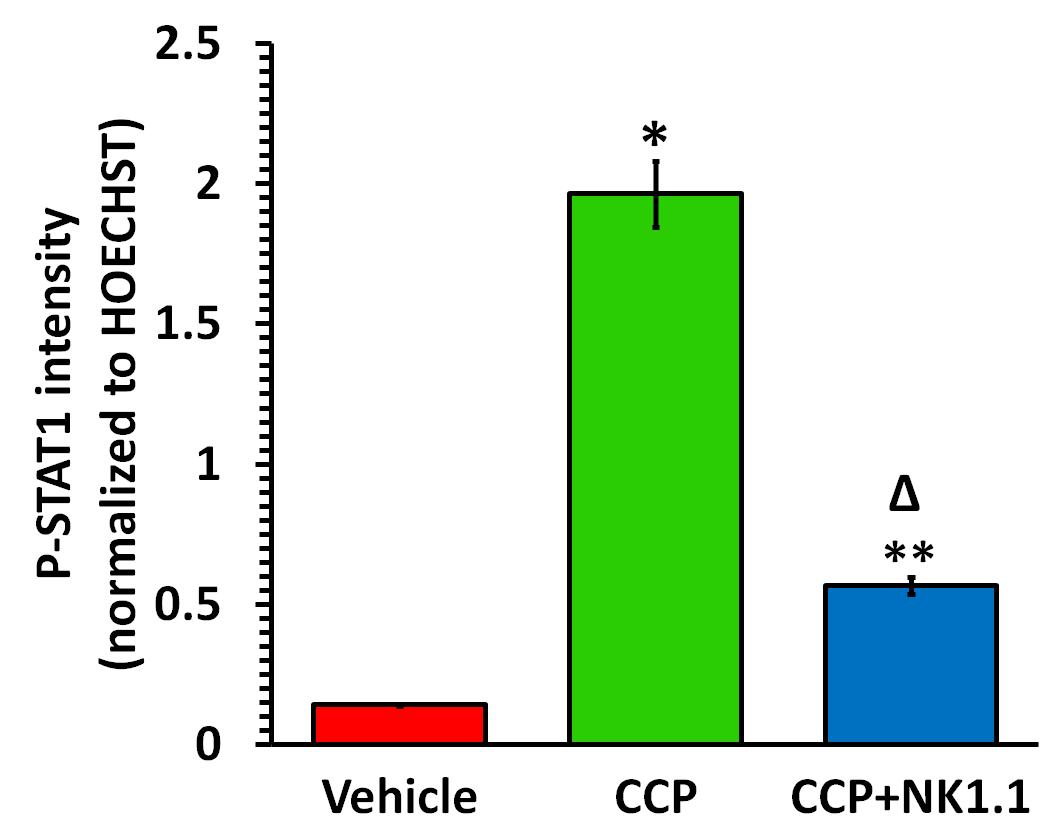** | | | **(C)**  **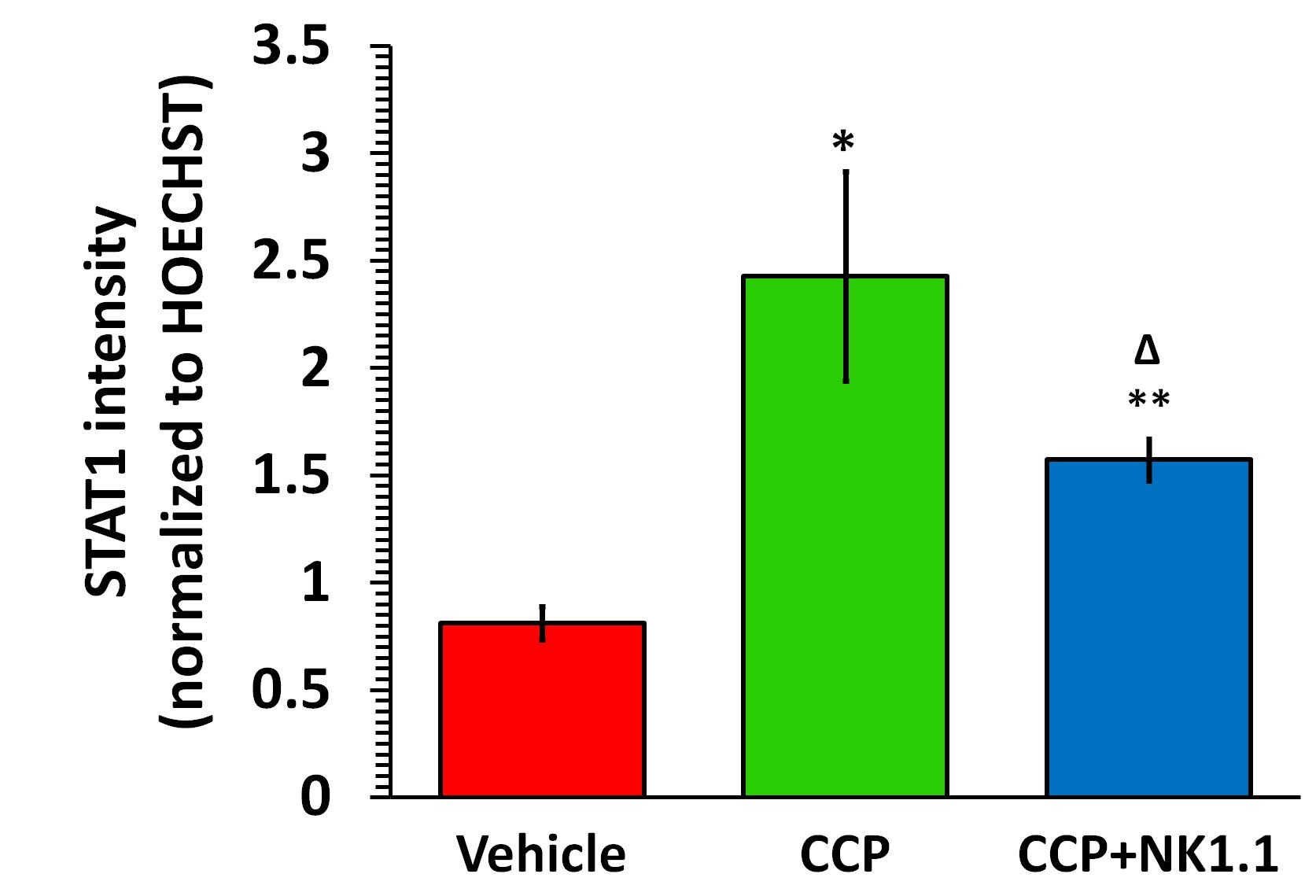** | | | | | | **(D)**  **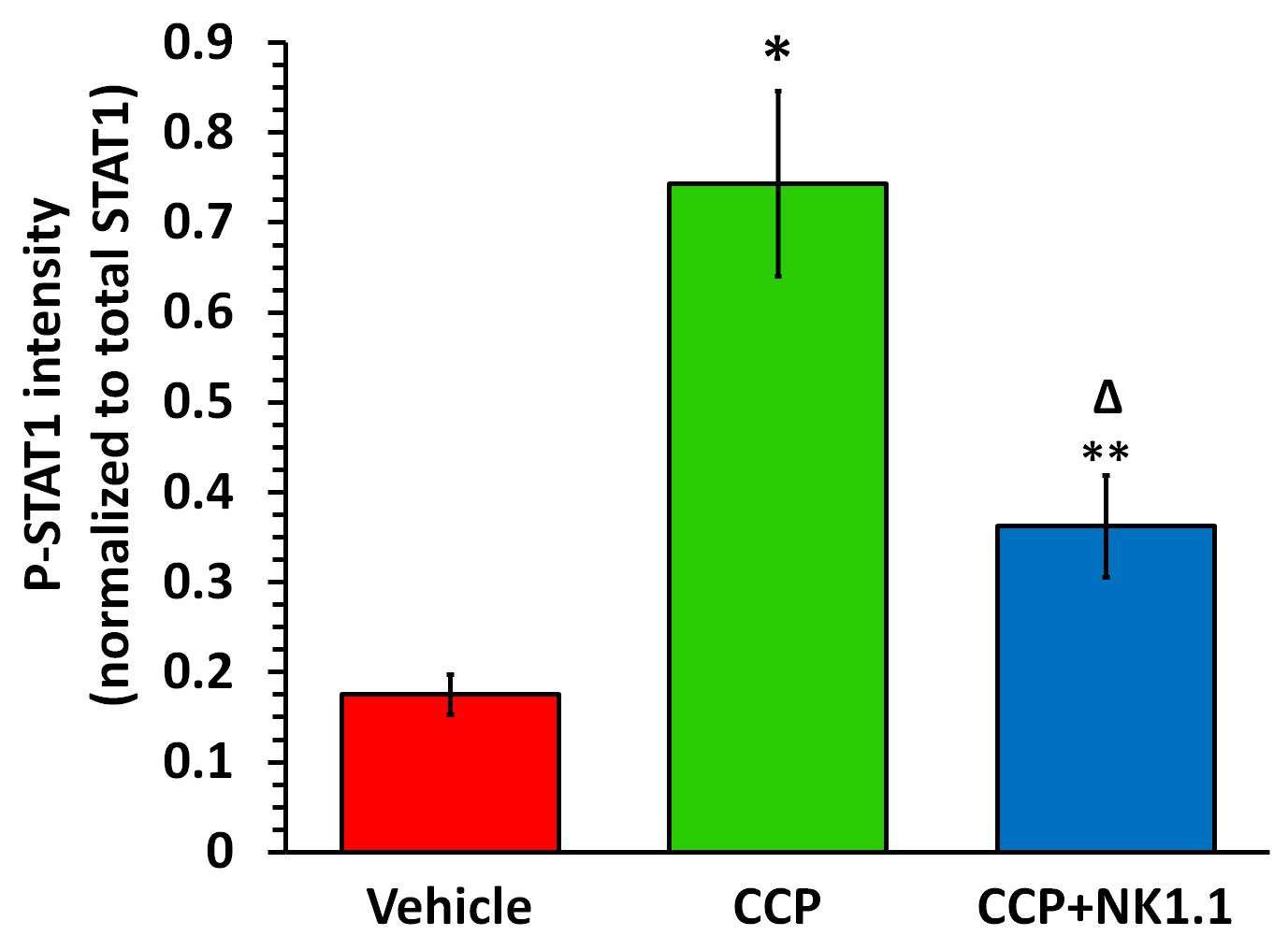** | | |

**Additional file 6: Figure S6. Peripheral neutralization of NK cells partially reverses the CCP-mediated induction and activation of STAT1 in the TAM.** GBM Brain sections parallel to those used in Figure S5 from the three groups (Vehicle, CCP and CCP+NK1.1Ab) were used to evaluate the levels of STAT1 and activated STAT1 (Tyr^701^-STAT1) (P-STAT1) in the Iba1(+) TAM. **(A)** The Vehicle-treated mice showed low levels of STAT1 (red) and P-STAT1 (purple) in the Iba1(+) (green) cells (First row and **B**), but a 1286% overall increase in P-STAT1 was observed in the CCP-treated GBM sections (Second row and **B**) (*p = 2.3 x 10^-4^, Vehicle versus CCP). This CCP-evoked increase in P-STAT1 was only 300% in the CCP+NK1.1 mouse samples (Third row and **B**) (Δ p = 0.04, CCP versus CCP+NK1.1). The CCP-evoked increase in P-STAT1 was the result of a 300% induction in STAT1 (only 194% increase in the CCP+NK1.1 sections) **(A, C),** and a 423% augmentation of P-STAT1 with respect to STAT1 (activation) (only 206% activation in the CCP+NK1.1 sections) (******p = 2.9x10^-4^ , Vehicle versus CCP+NK1.1) **(A, D).** Three sections per mouse were used for imaging and the graphs represents mean ± S.D. obtained from mice treated with Vehicle (n=4), CCP (n=4), and CCP+NK1.1 (n=3). (Scale bar: 47.62 µm).
