# Supplementary material for: Phytosomal curcumin causes natural killer cell-dependent repolarization of glioblastoma (GBM) tumor-associated microglia/macrophages and elimination of GBM and GBM stem cells
Source: J Exp Clin Cancer Res. 2018 Jul 25;37:168. doi: 10.1186/s13046-018-0792-5 (PMC6058381; doi:10.1186/s13046-018-0792-5)
Supplement: Supplementary file 7 — Figure S7. Peripheral neutralization of NK cells partially reverses the CCP-induced M2➔M1 repolarization of TAM within the GBM mass. To verify the data presented in Fig. 4, brain sections parallel to those used in Fig. S6, harboring the GBM tumor from the three groups of mice (Vehicle, CCP and CCP + NK1.1Ab) were triple-stained with Iba1 (green), iNOS (red) and ARG1 (purple) antibodies. (A) The Vehicle-treated GBM sections displayed weak iNOS staining but strong ARG1 staining in the Iba1(+) TAM (A, top row). In contrast, the CCP-treated mice presented a 58% decrease in ARG1 (*p = 2.5 × 10− 6 CCP versus Vehicle). This CCP-evoked suppression in ARG1 was only 35% in the CCP + NK1.1 sections (Δ p = 5.6 × 10− 7, CCP + NK1.1 versus CCP; **p = 3 × 10− 4, CCP + NK1.1 versus Vehicle) (A, middle row, and B). In contrast, the Iba1(+) TAM in the CCP-treated mice showed a 212% increase in iNOS (*p = 6.2 × 10− 7, CCP versus Vehicle) and this CCP-evoked increase in iNOS was only 147% in the CCP + NK1.1 group (Δ p = 1.1 × 10− 4, CCP + NK1.1 versus CCP; **p = 7.6 × 10− 5, CCP + NK1.1 versus Vehicle) (A, lowest row, and C). Four sections per mouse were used for imaging and the graphs represent mean ± S.D. obtained from Vehicle (n = 4), CCP (n = 4), and CCP + NK1.1 (n = 3). (Scale bar: 47.62 μm). (DOC 1300 kb) [file 13046_2018_792_MOESM7_ESM.doc]

| **(A)** | **ARG1** | **iNOS** | **Iba1** | | **HOECHST** | **Merged** |
| --- | --- | --- | --- | --- | --- | --- |
|  | **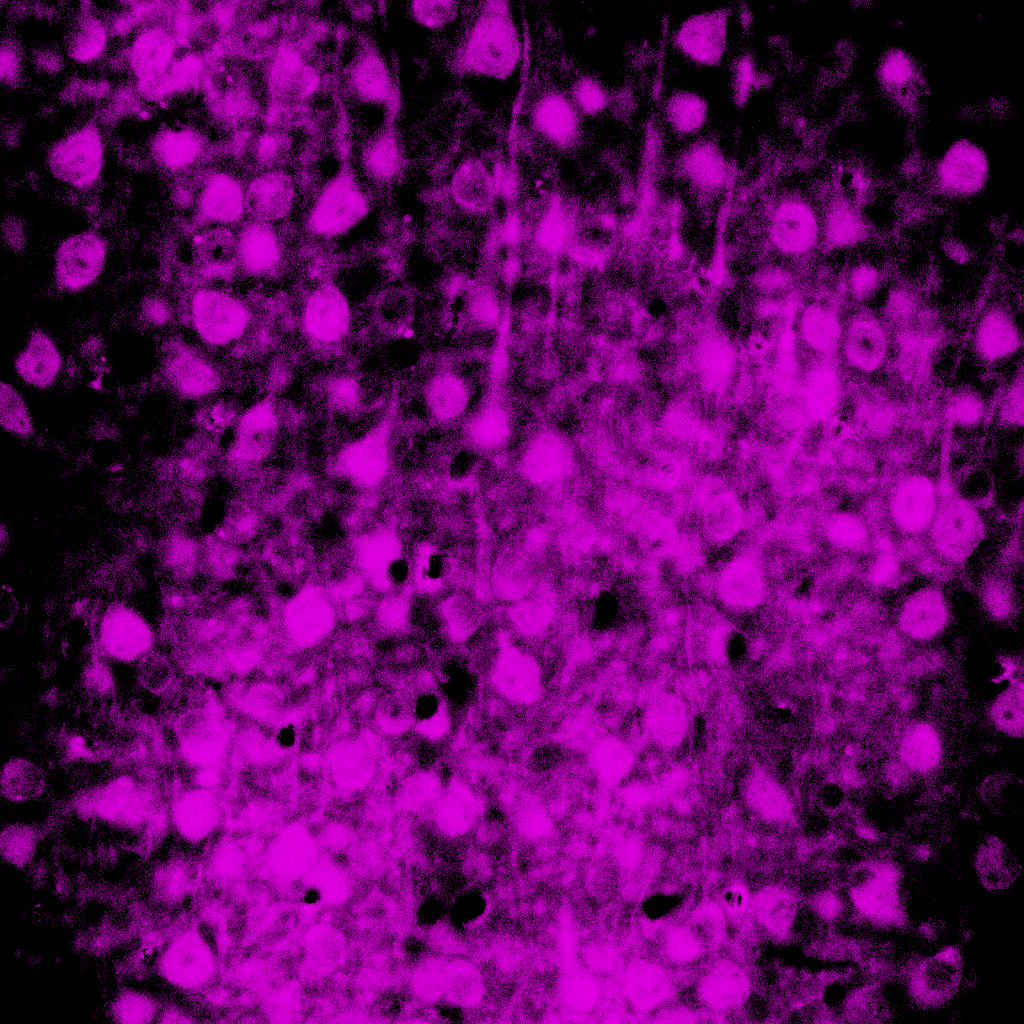** | **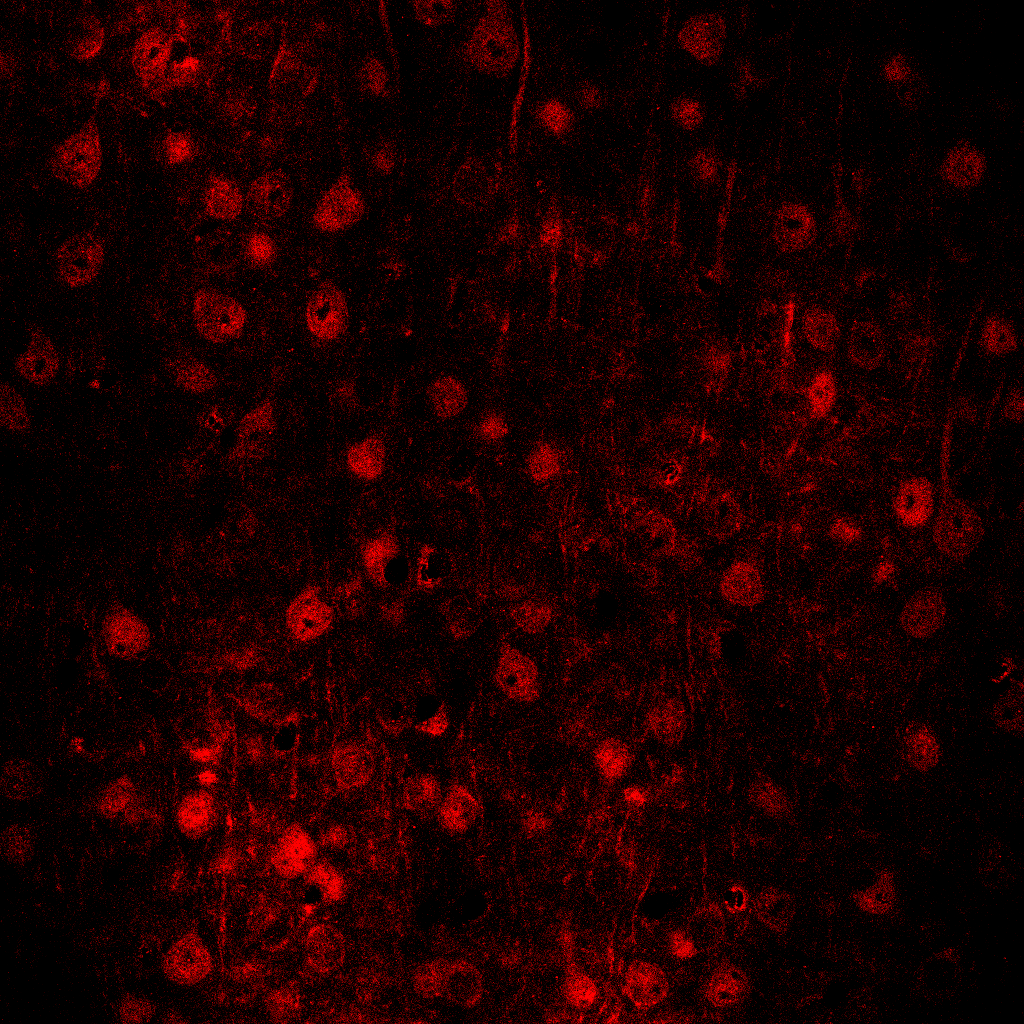** | **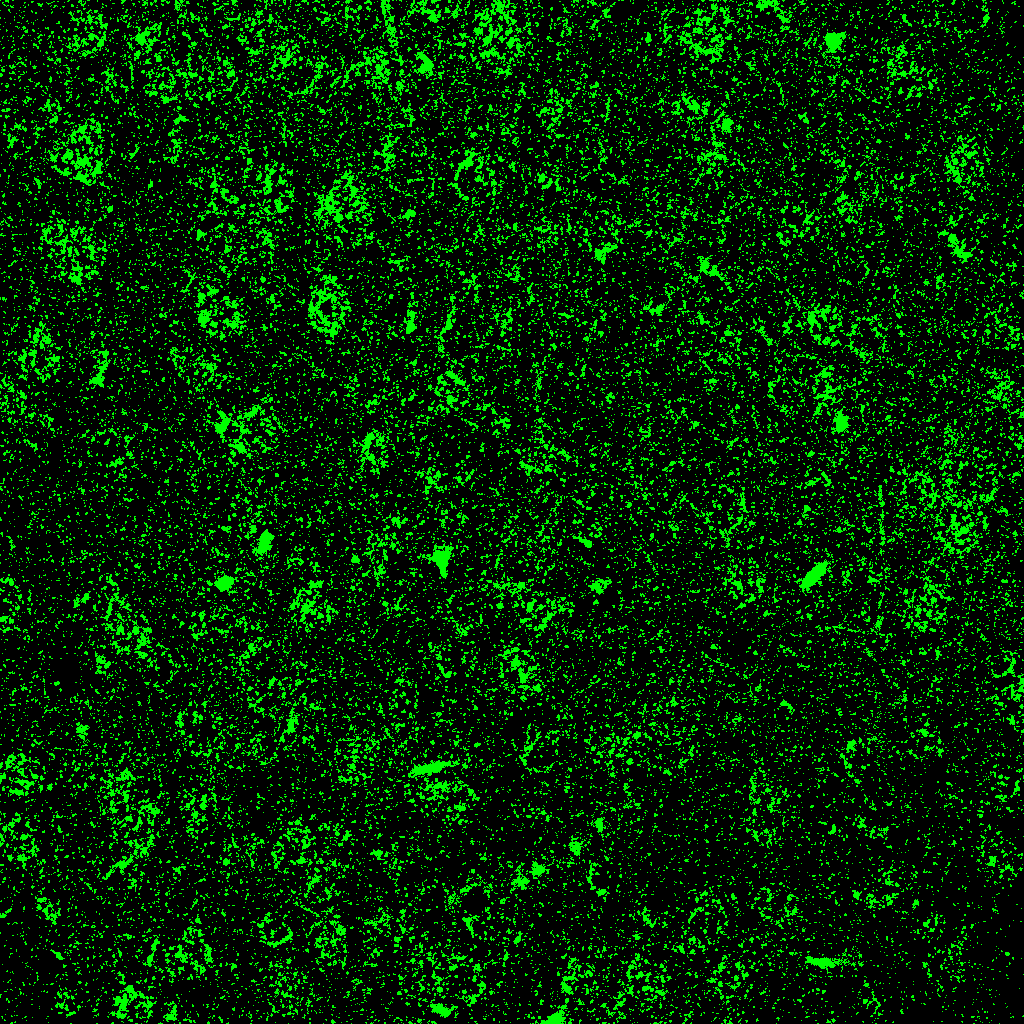** | | **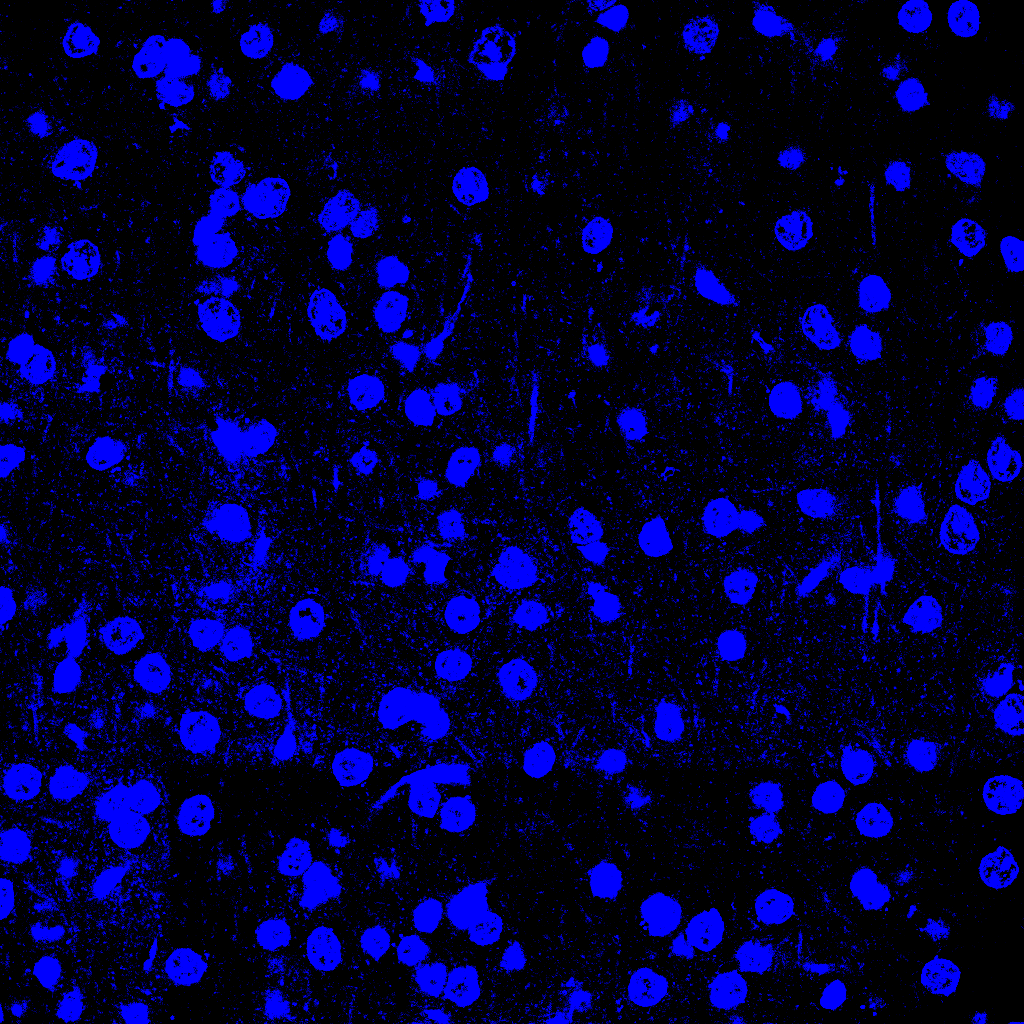** | **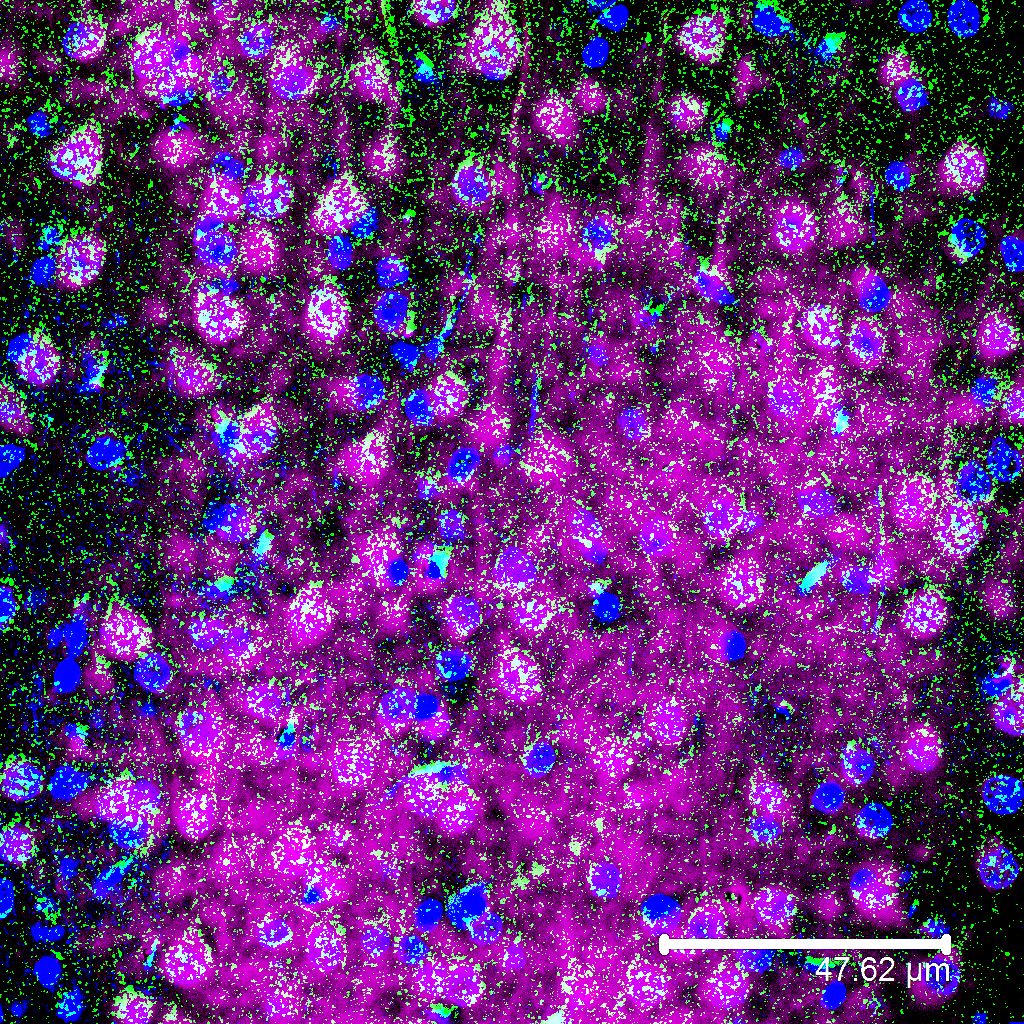** |
| **Vehicle** |
| **CCP** | **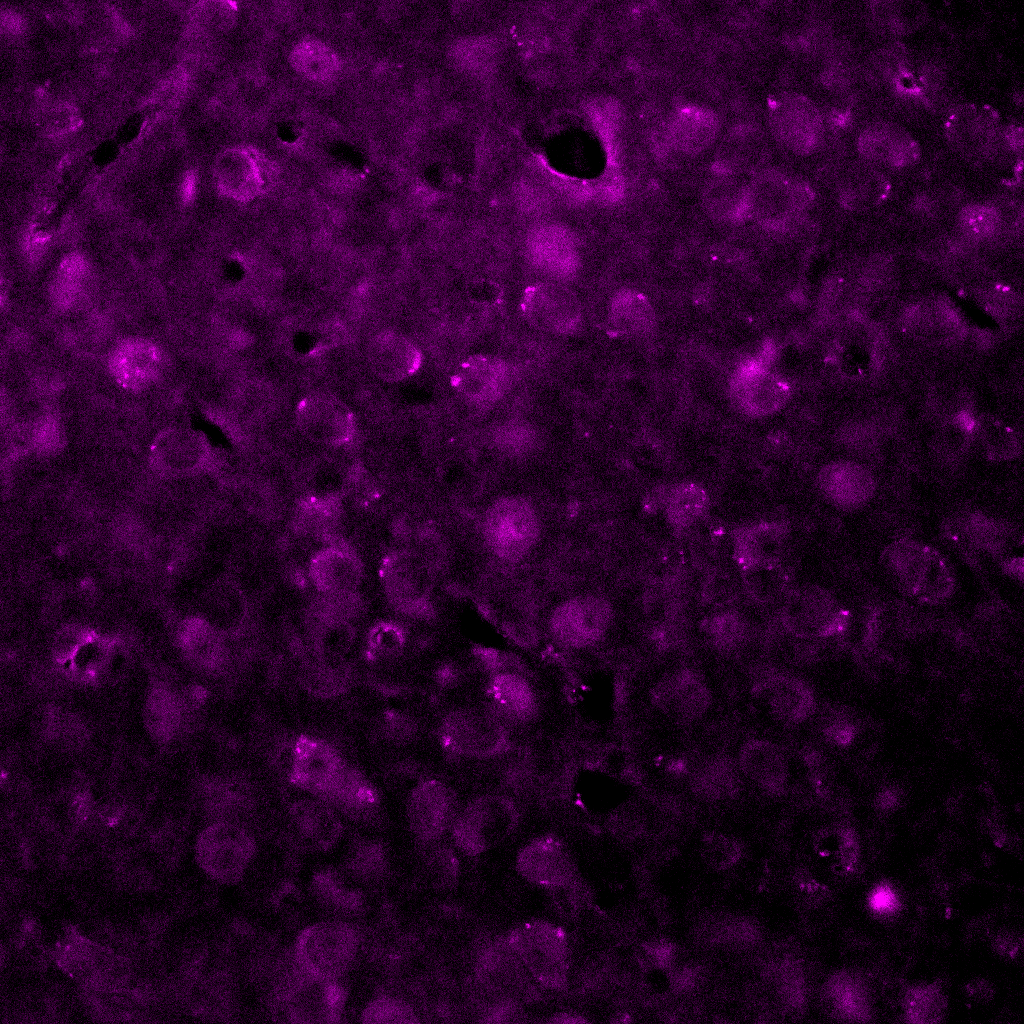** | **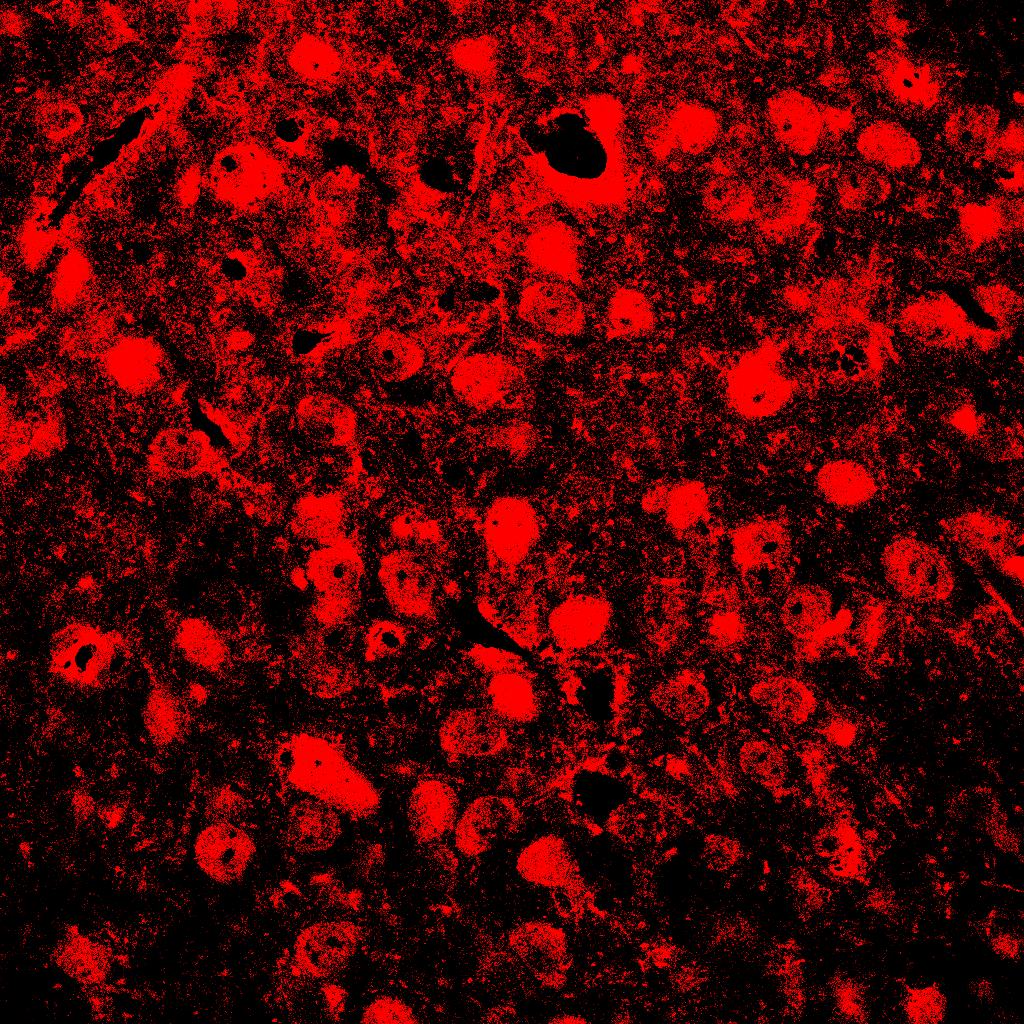** | **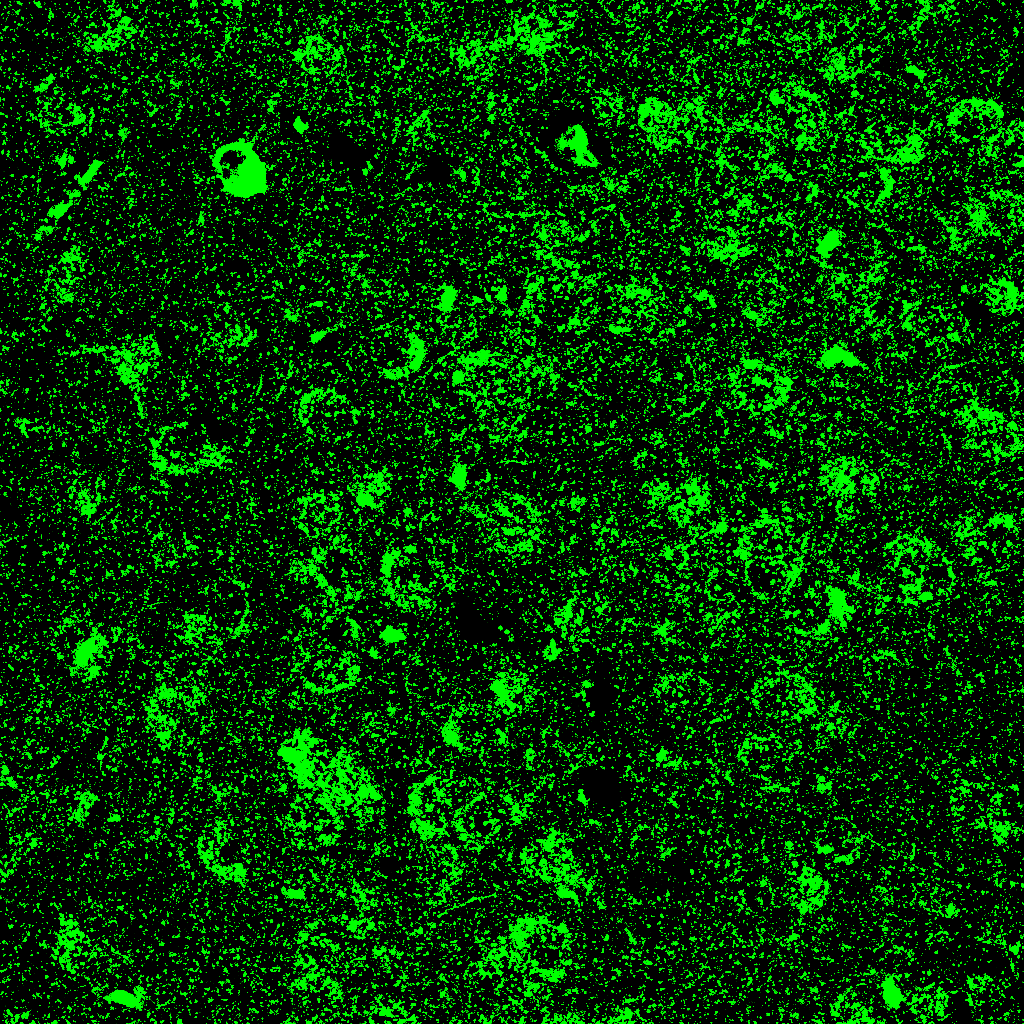** | | **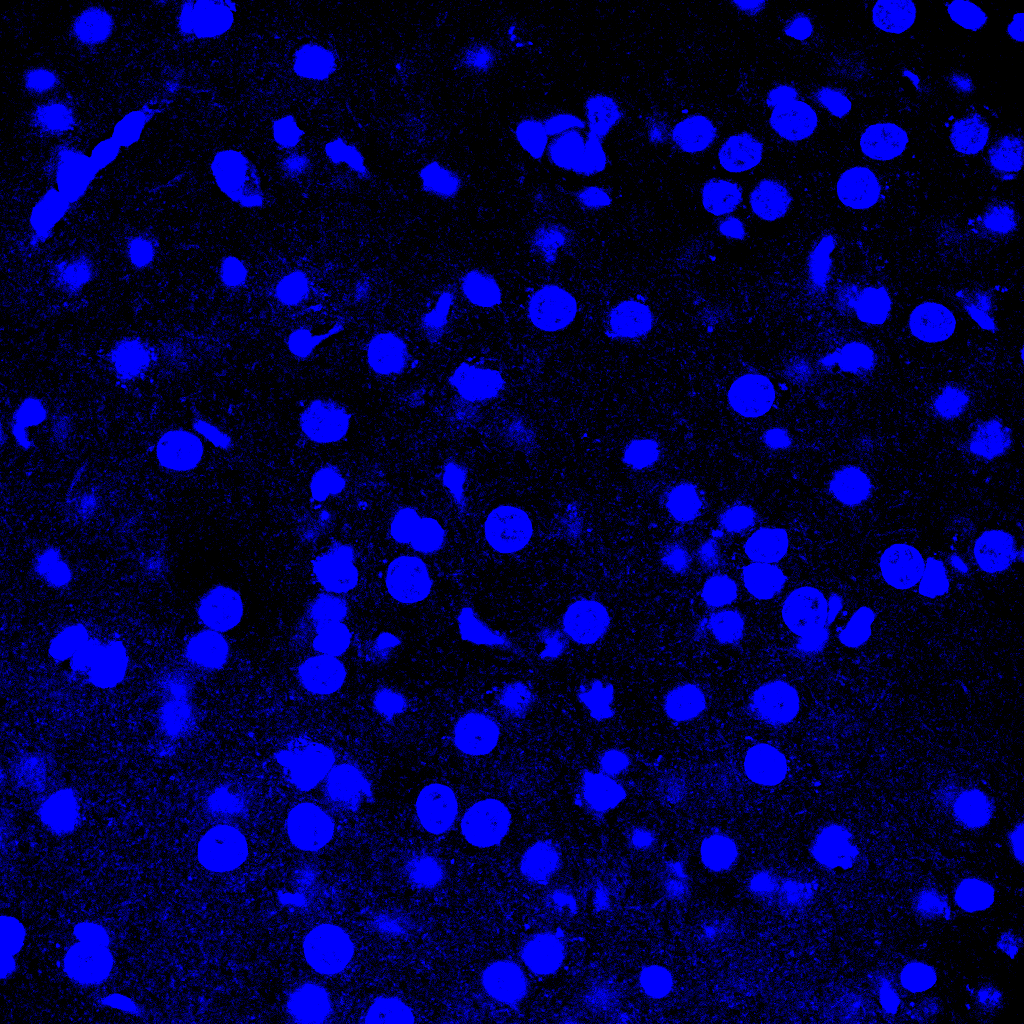** | **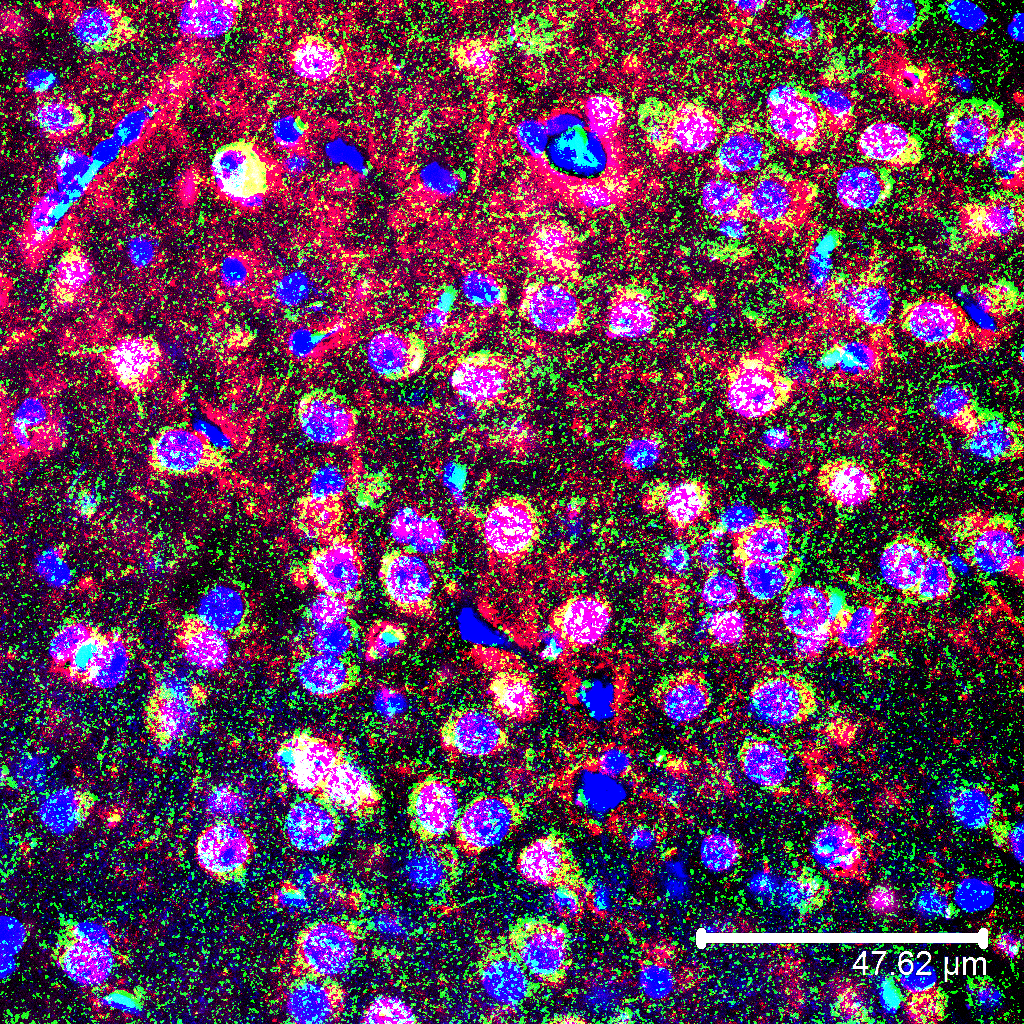** |
| **CCP + NK 1.1Ab** | **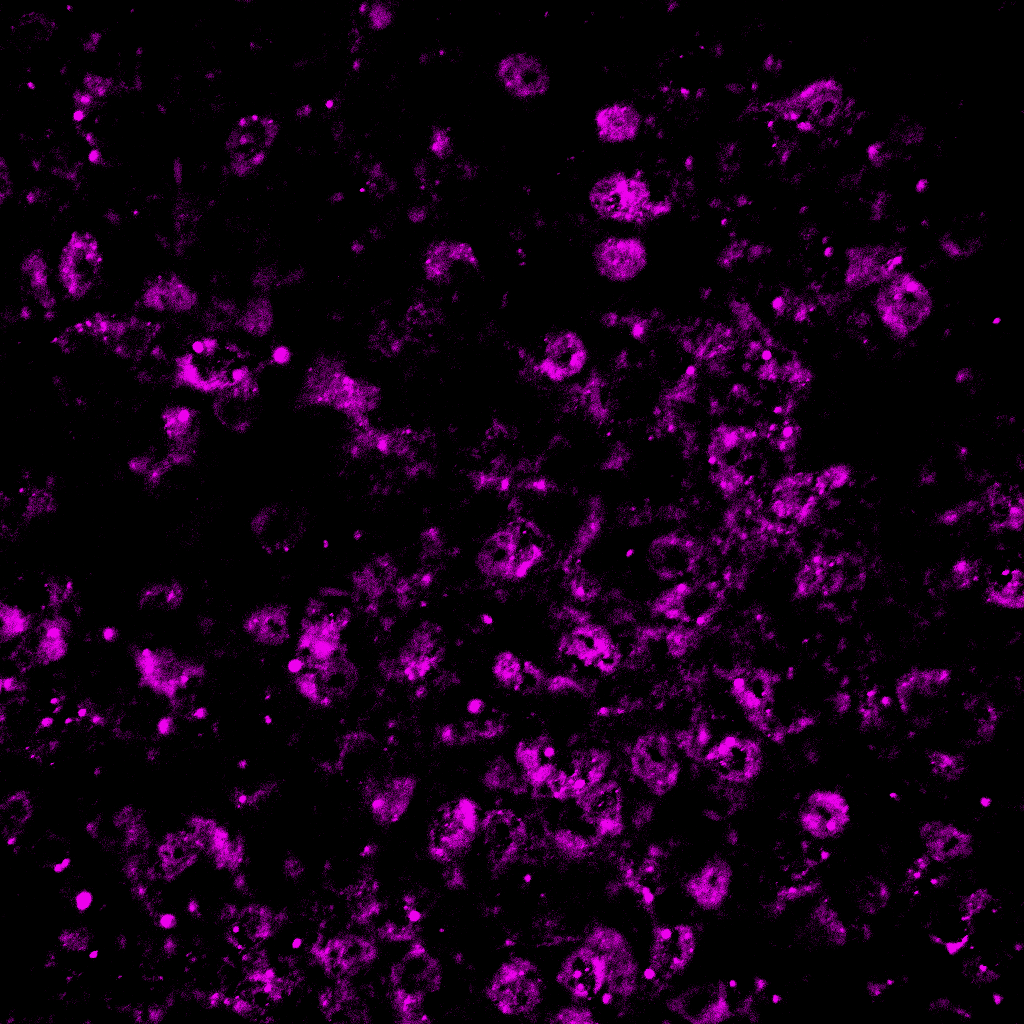** | **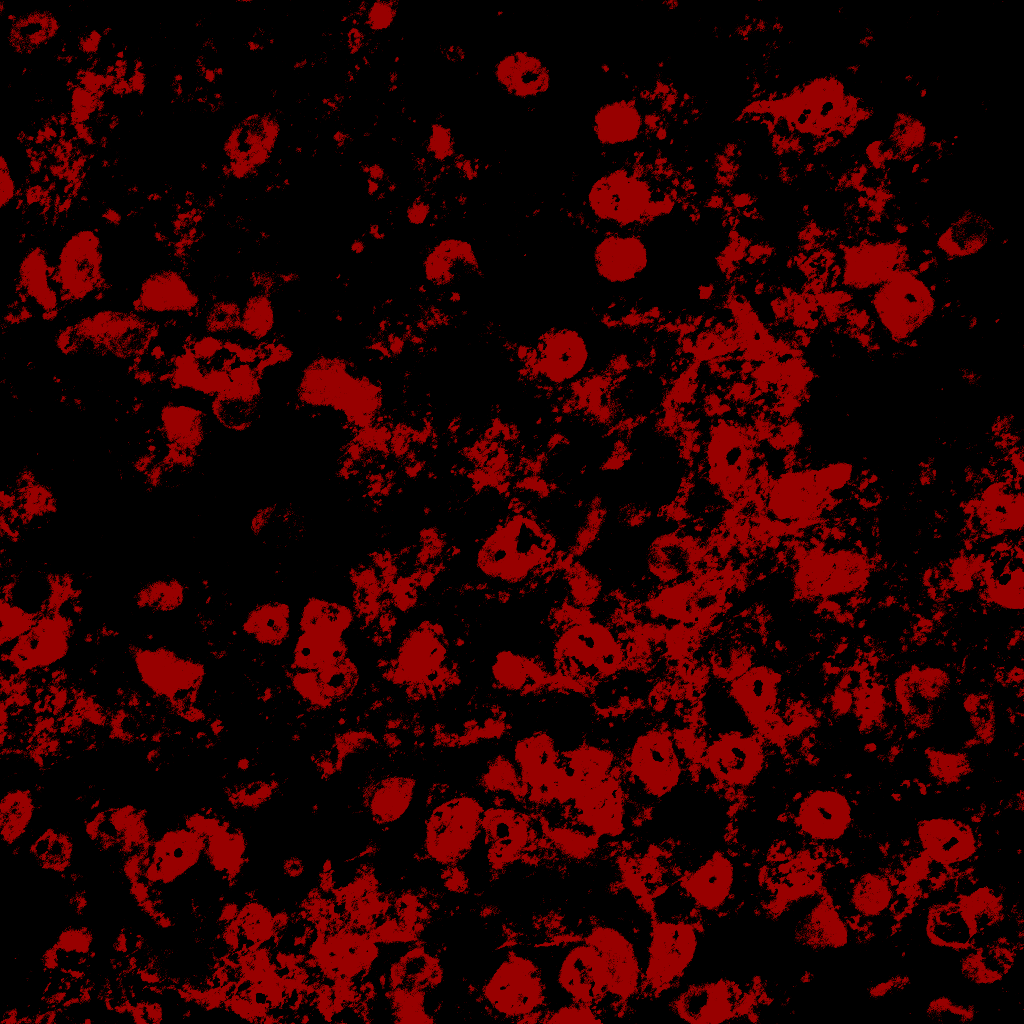** | **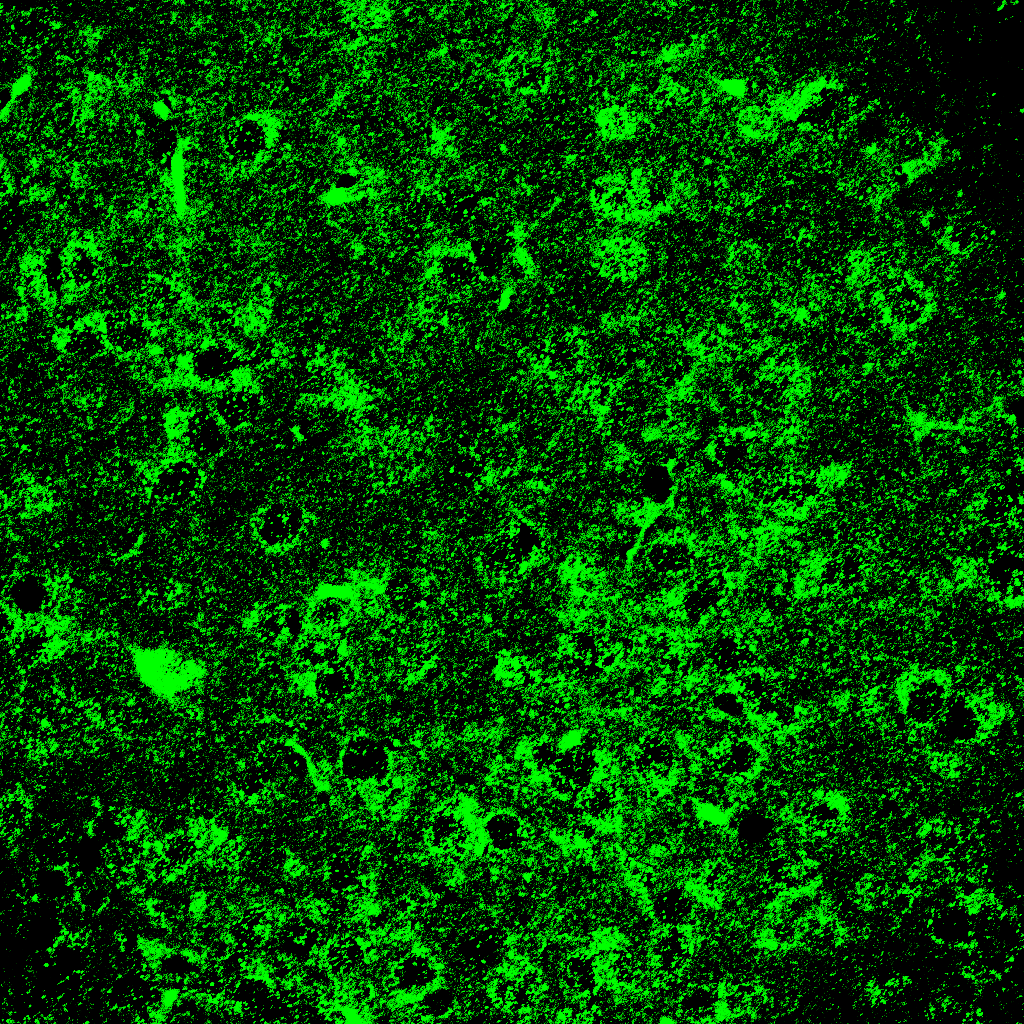** | | **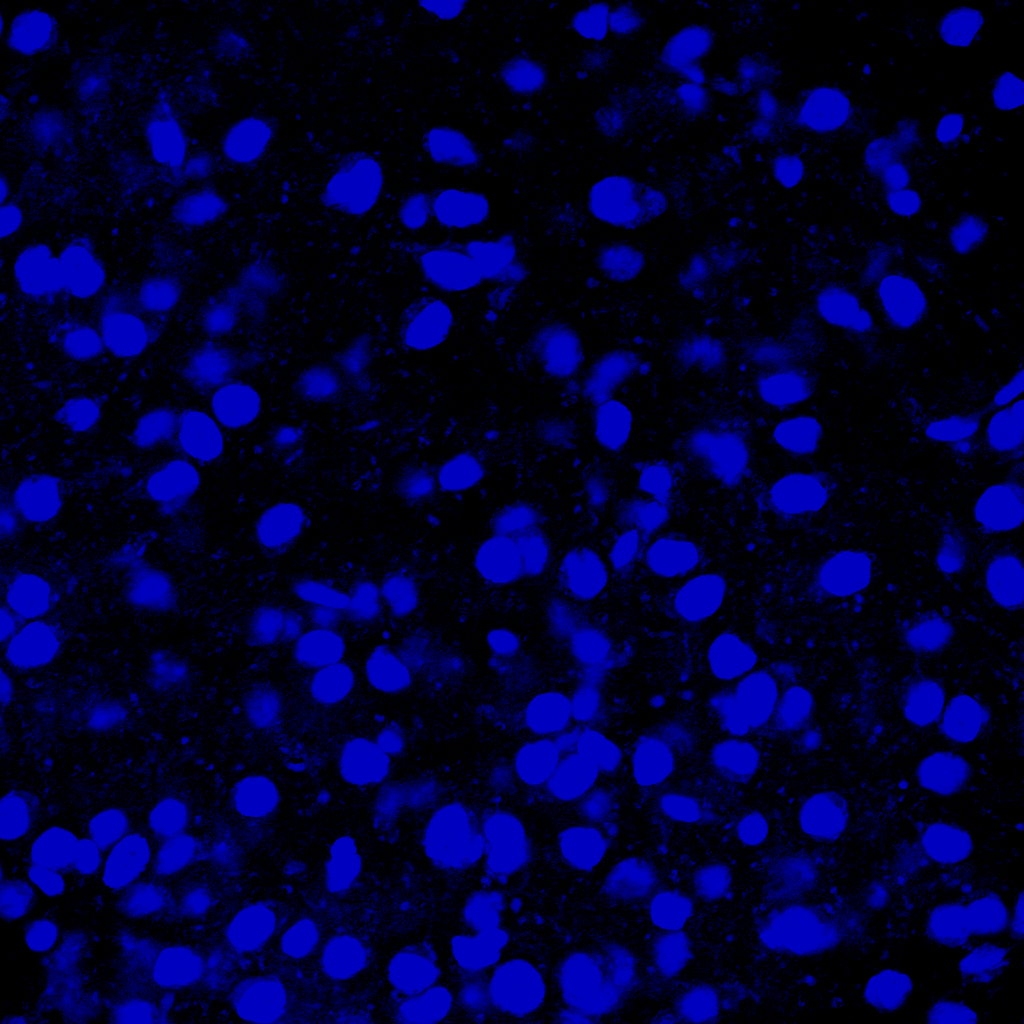** | **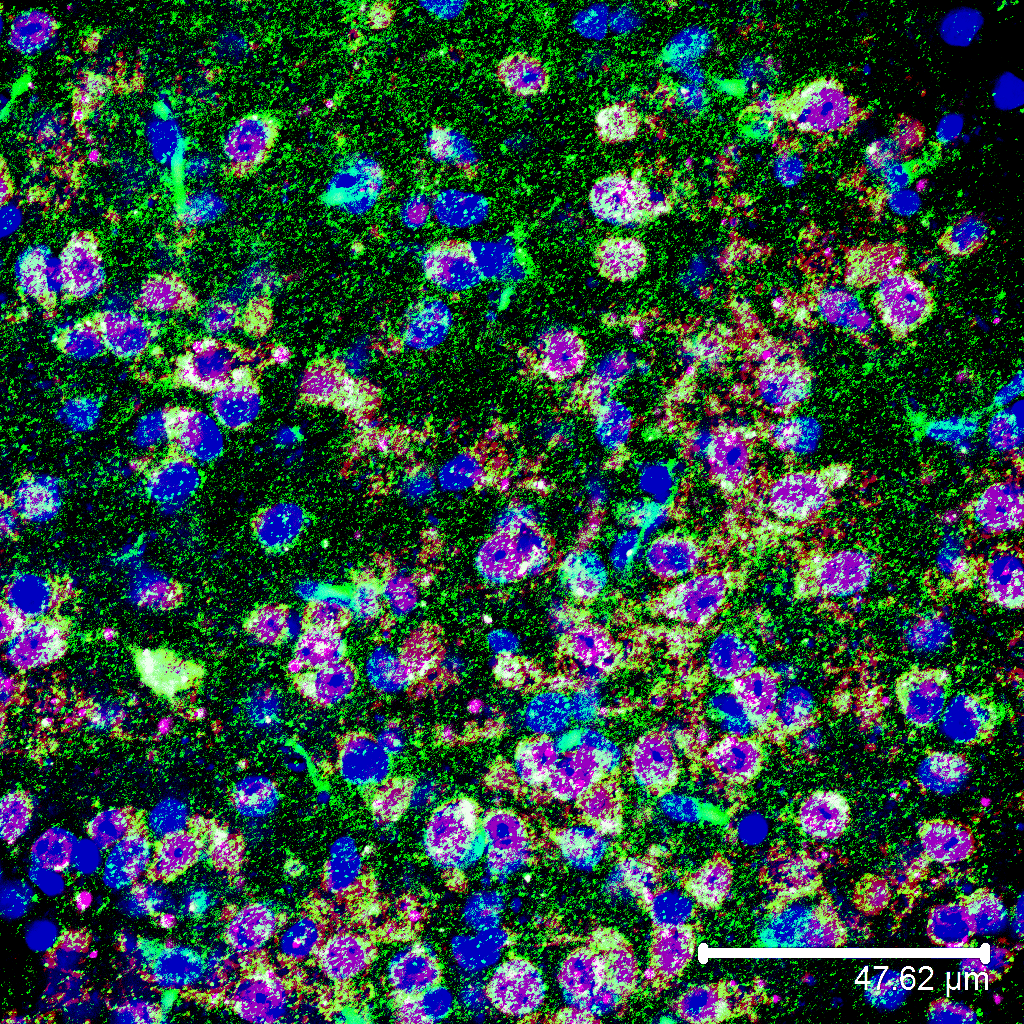** |
|  | **(B)**  **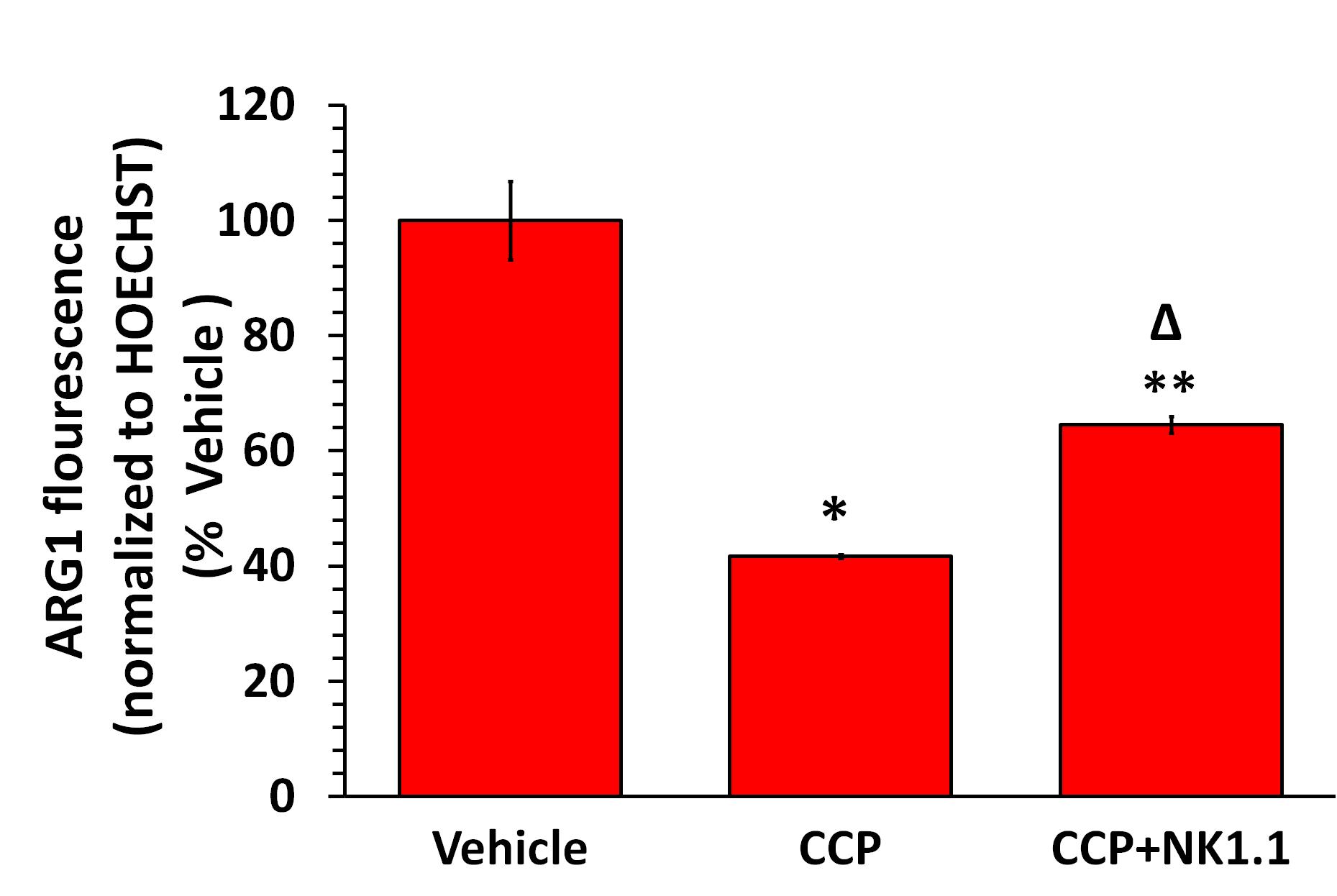** | | | **(C)**  **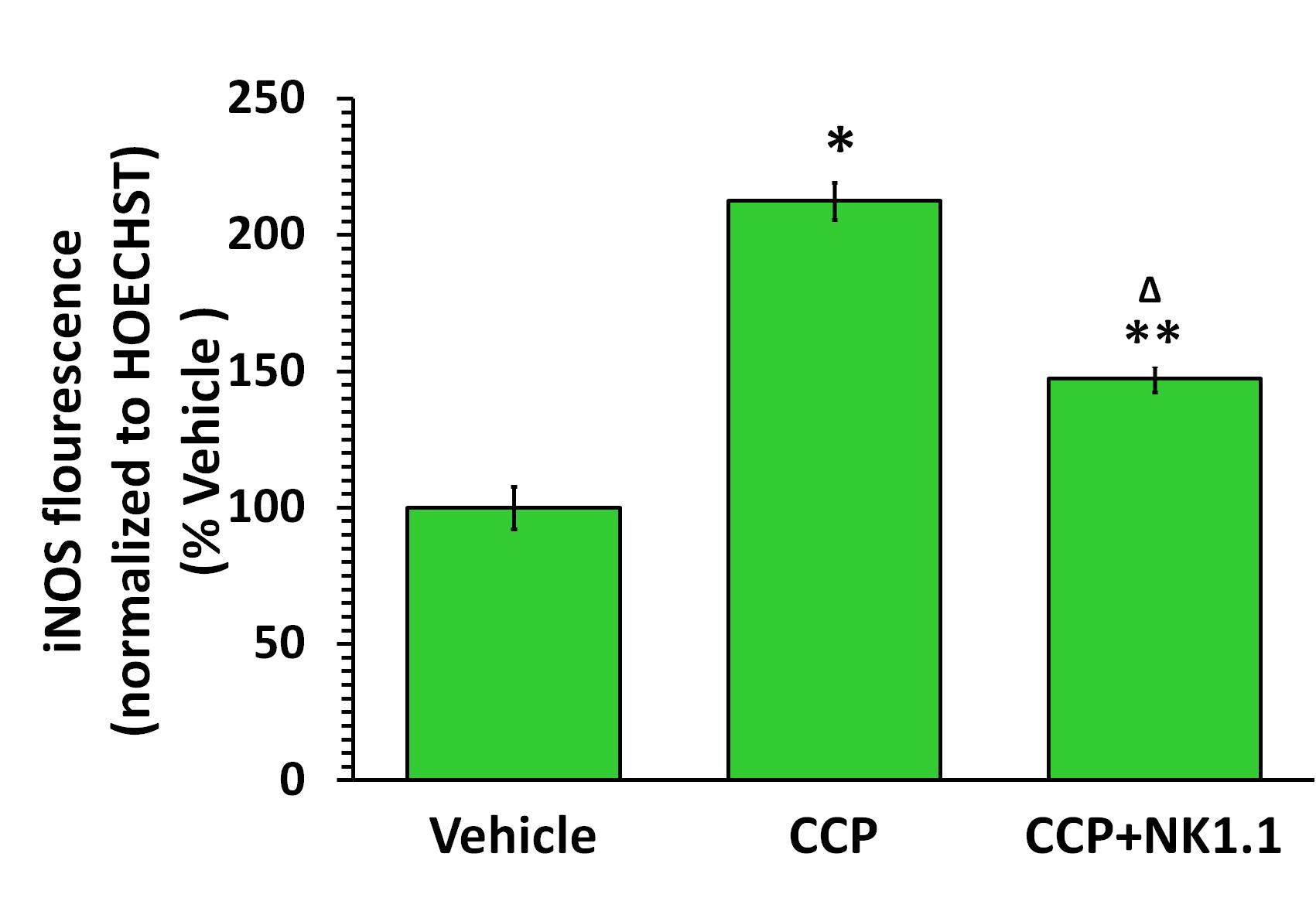** | | |

**Additional file 7: Figure S7. Peripheral neutralization of NK cells partially reverses the CCP-induced M2🡪M1 repolarization of TAM within the GBM mass.** To verify the data presented in Figure 4, brain sections parallel to those used in Figure S6, harboring the GBM tumor from the three groups of mice (Vehicle, CCP and CCP+NK1.1Ab) were triple-stained with Iba1 (green), iNOS (red) and ARG1 (purple) antibodies. **(A)** The Vehicle-treated GBM sections displayed weak iNOS staining but strong ARG1 staining in the Iba1(+) TAM (**A**, top row). In contrast, the CCP-treated mice presented a 58% decrease in ARG1 (*p= 2.5 x 10^-6^ CCP versus Vehicle). This CCP-evoked suppression in ARG1 was only 35% in the CCP+NK1.1 sections (Δ p=5.6x10^-7^, CCP+NK1.1 versus CCP; **p=3x10^-4^, CCP+NK1.1 versus Vehicle ) (**A**, middle row, and **B**). In contrast, the Iba1(+) TAM in the CCP-treated mice showed a 212% increase in iNOS (*p= 6.2x10^-7^, CCP versus Vehicle) and this CCP-evoked increase in iNOS was only 147% in the CCP+NK1.1 group (Δ p=1.1x10^-4^, CCP+NK1.1 versus CCP; **p=7.6x10^-5^, CCP+NK1.1 versus Vehicle) (**A**, lowest row, and **C**). Four sections per mouse were used for imaging and the graphs represent mean ± S.D. obtained from Vehicle (n=4), CCP (n=4), and CCP+NK1.1 (n=3). (Scale bar: 47.62 µm).
