# Supplementary material for: Phytosomal curcumin causes natural killer cell-dependent repolarization of glioblastoma (GBM) tumor-associated microglia/macrophages and elimination of GBM and GBM stem cells
Source: J Exp Clin Cancer Res. 2018 Jul 25;37:168. doi: 10.1186/s13046-018-0792-5 (PMC6058381; doi:10.1186/s13046-018-0792-5)
Supplement: Supplementary file 8 — Figure S8. Abrogation of NK cells by peripheral infusion of NK1.1 antibody partially reverts the CCP-mediated suppression of IL10 and induction of IL12 in the TAM within the GBM mass. As a corroboration of the flow cytometry data presented in Fig. 4, GBM brain sections parallel to those used in Fig. S7, harboring the tumor from the three groups (Vehicle, CCP and CCP + NK1.1Ab) were triple-stained with antibodies against Iba1 (green), IL12 (red), and IL10 (purple). Sections from Vehicle-treated mice showed strong IL10 expression in the Iba1(+) TAM (A first row), which was suppressed by 83% in the CCP-treated mice (*p = 9.1 × 10− 8, CCP versus Vehicle) (A, second row and B), but this CCP-evoked suppression of IL10 was only 45% in the CCP + NK1.1 sections (Δ p = 8.3 × 10− 8, CCP + NK1.1 versus CCP; **p = 2.7 × 10− 5, CCP + NK1.1 versus Vehicle) (A third row and B). In contrast, IL12 expression in the Iba1(+) cells was very low in the sections from the Vehicle-treated mice (A first row), but it increased by 439% in the CCP-treated mice (*p = 1.3 × 10− 10, CCP versus Vehicle) (A second row and C), and this increase was only 277% in the CCP + NK1.1 mice (Δ p = 9.3 × 10− 6, CCP + NK1.1 versus CCP; **p = 4.6 × 10− 7, CCP + NK1.1 versus Vehicle) (A third row and C). Four sections per mouse from Vehicle (n = 4), CCP (n = 4), and CCP + NK1.1 (n = 3) mice were used for imaging and each graph represents mean ± S.D. (Scale bar: 47.62 μm). (DOC 4096 kb) [file 13046_2018_792_MOESM8_ESM.doc]

| **(A)** | **IL10** | **IL12** | **Iba1** | | **HOECHST** | **Merged** |
| --- | --- | --- | --- | --- | --- | --- |
| **Vehicle** | **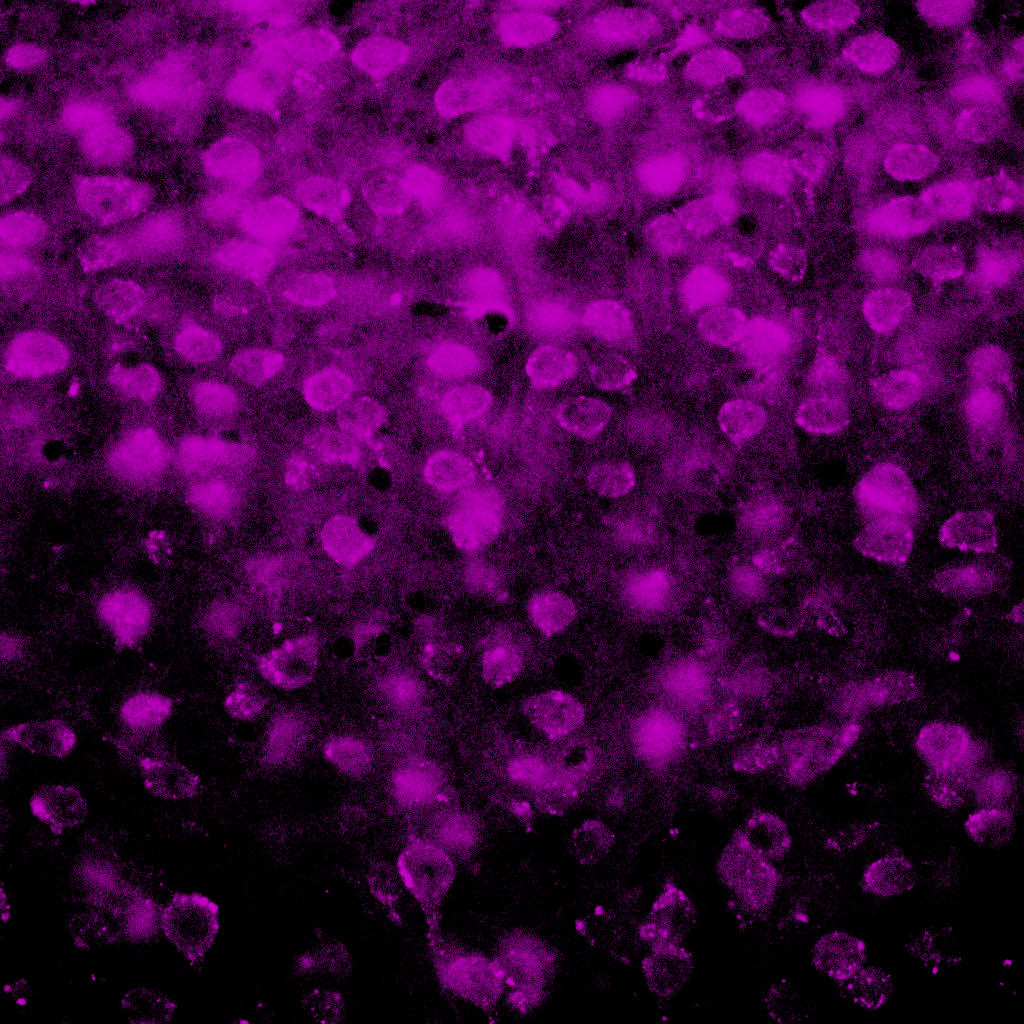** | **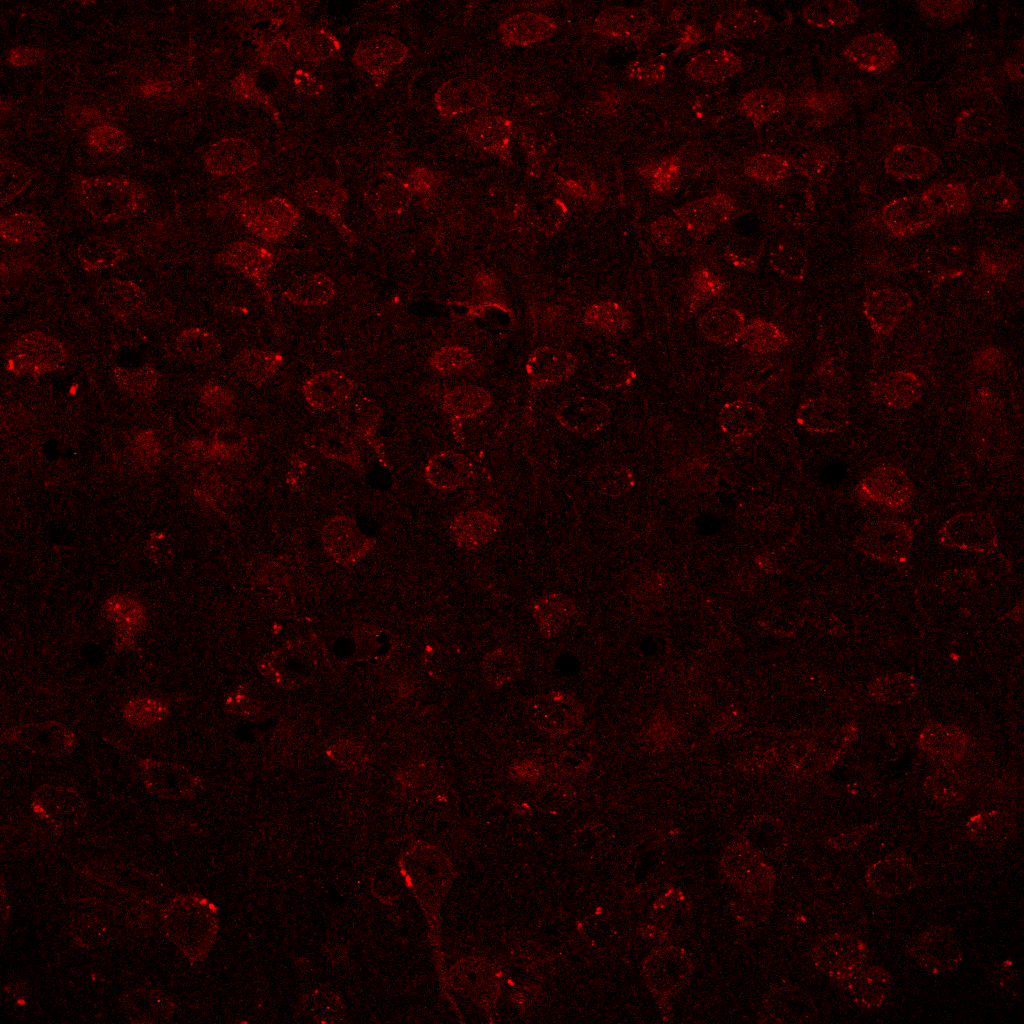** | **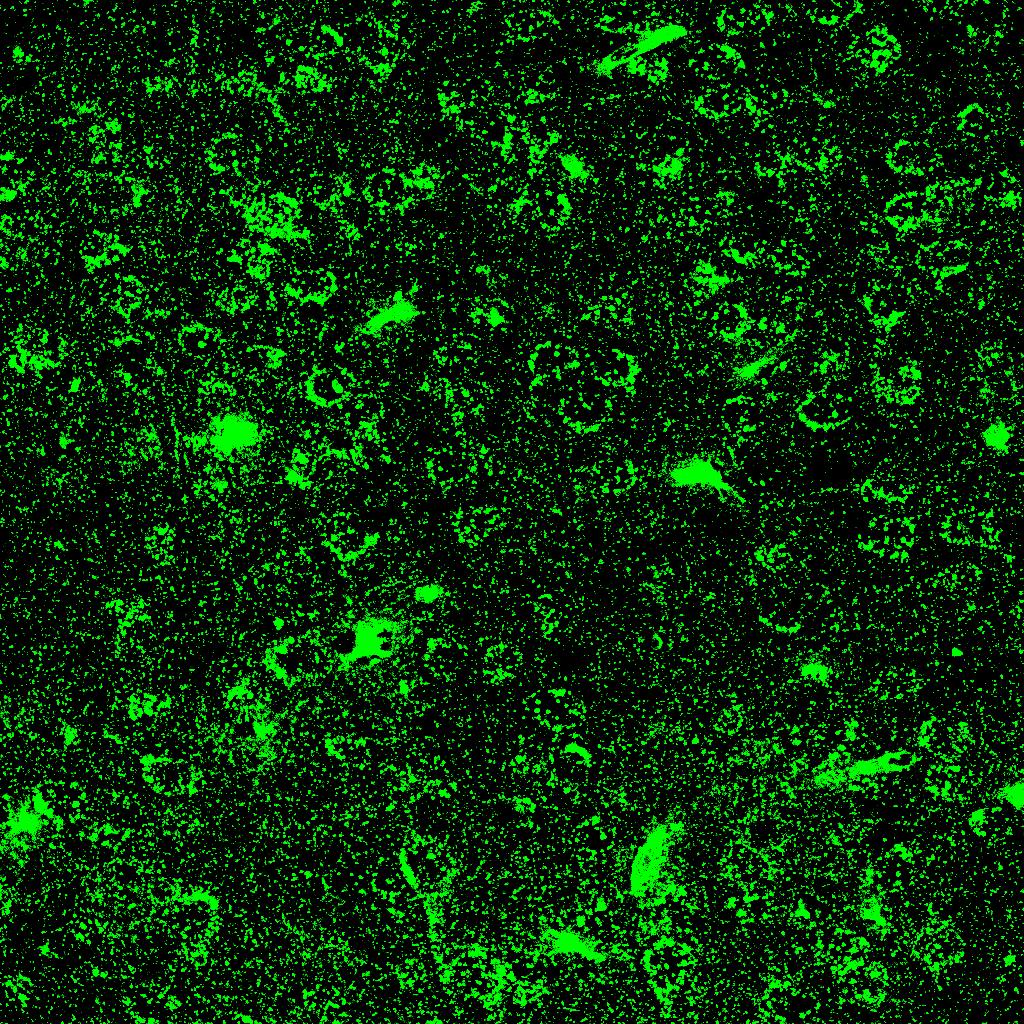** | | **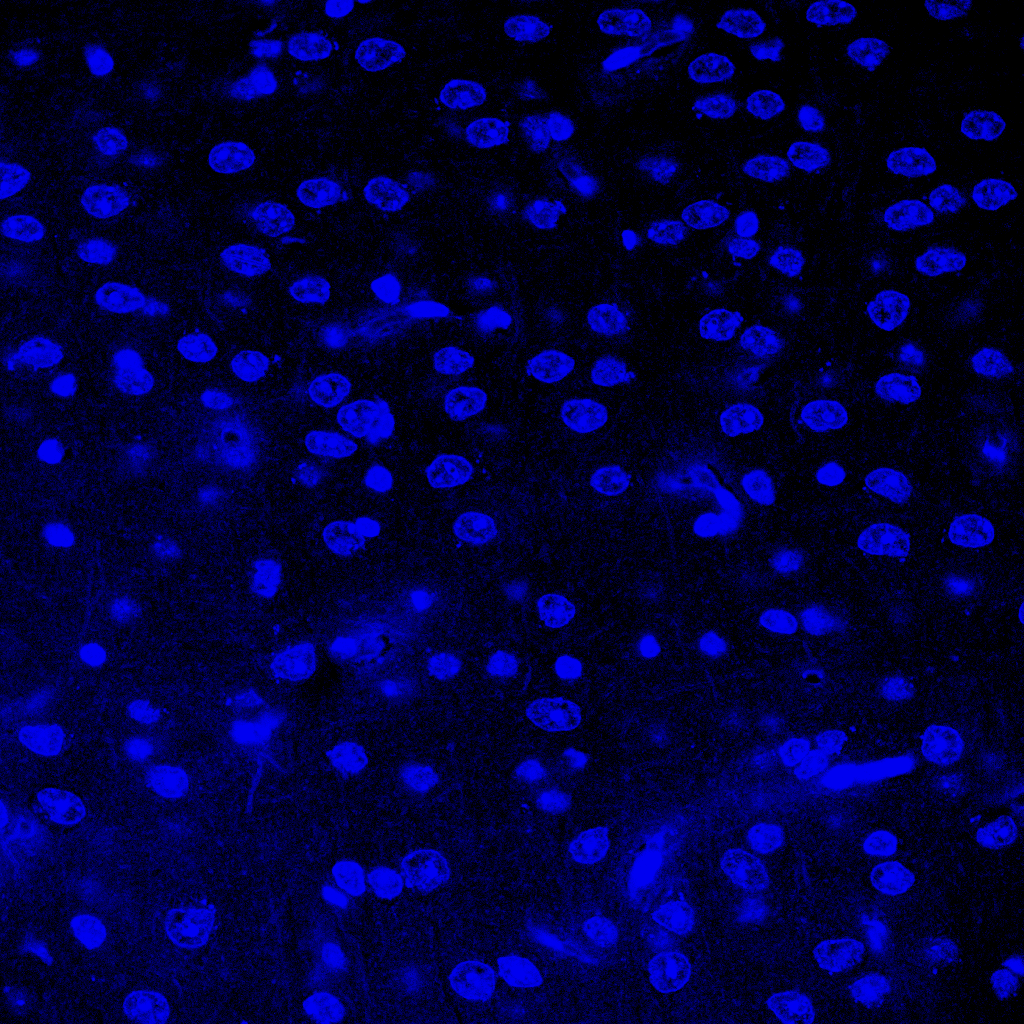** | **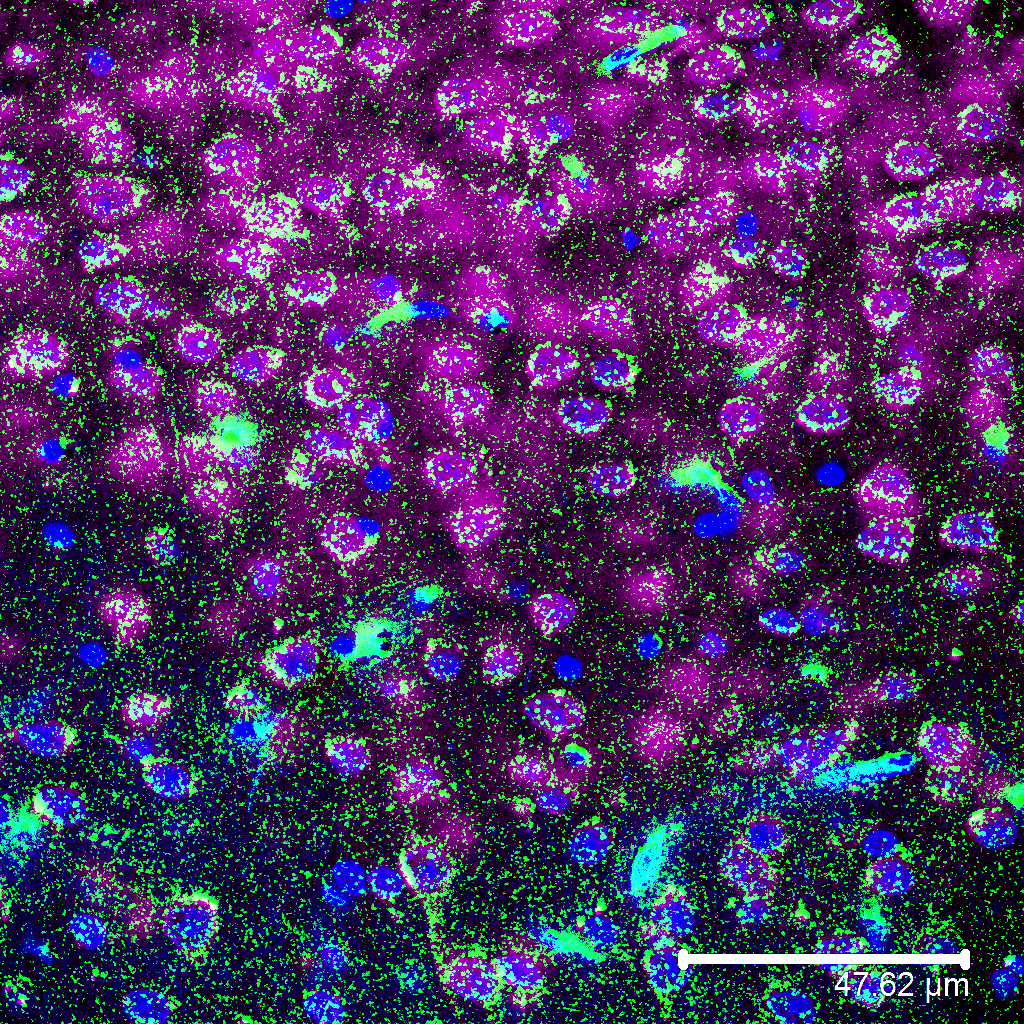** |
| **CCP** | **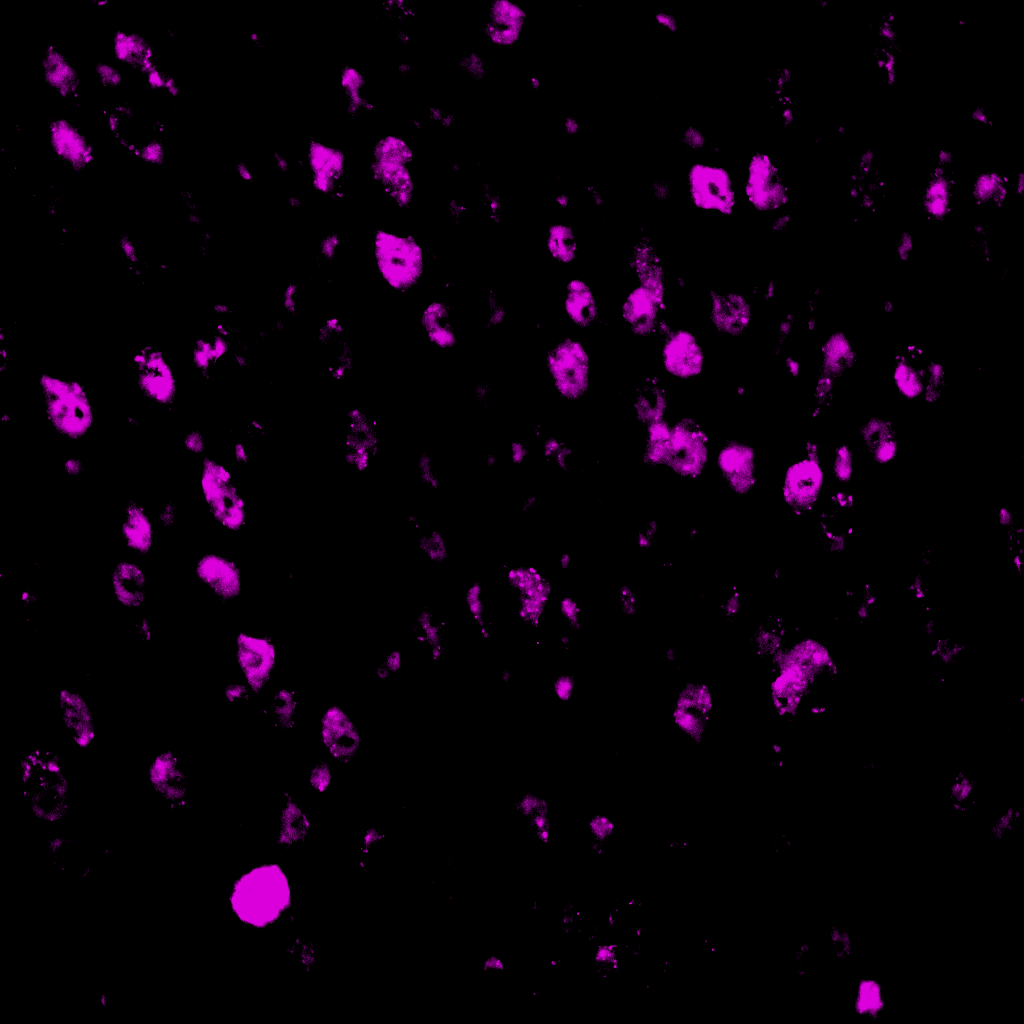** | **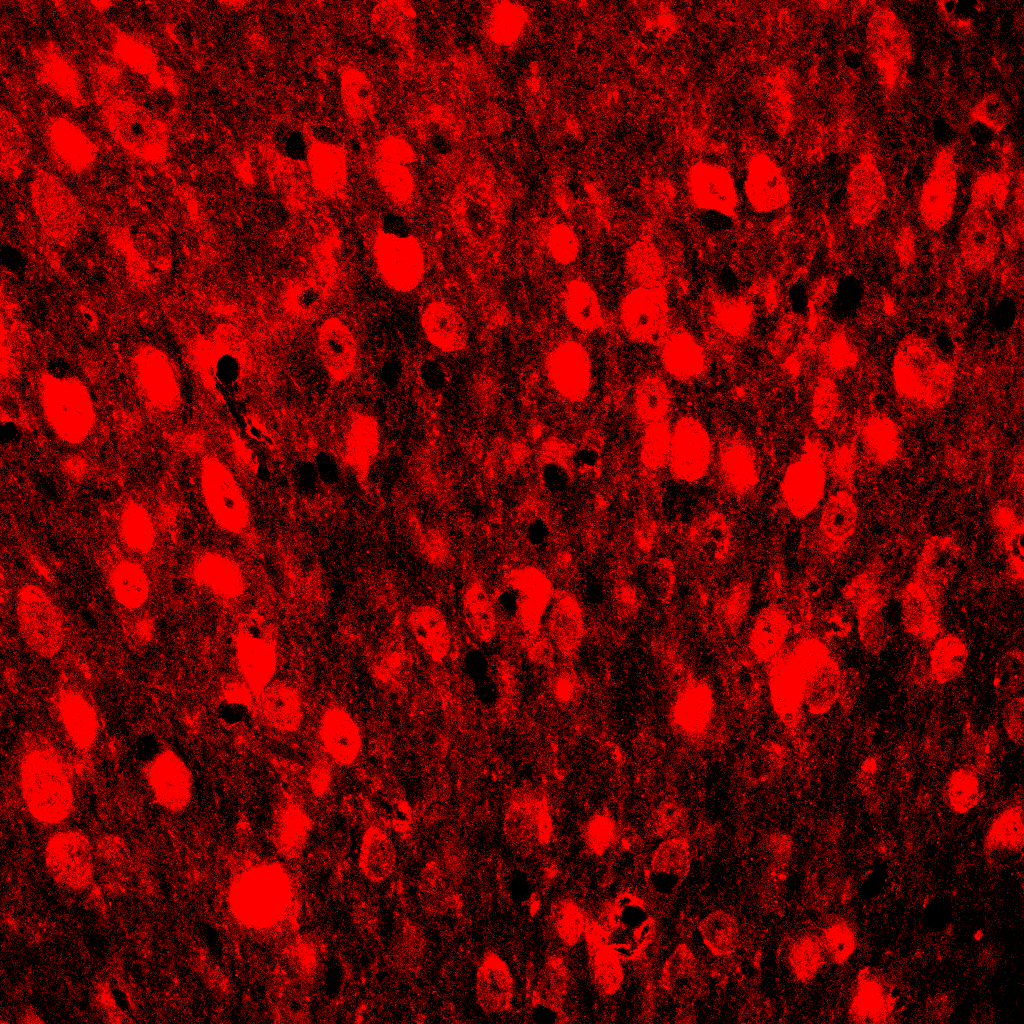** | **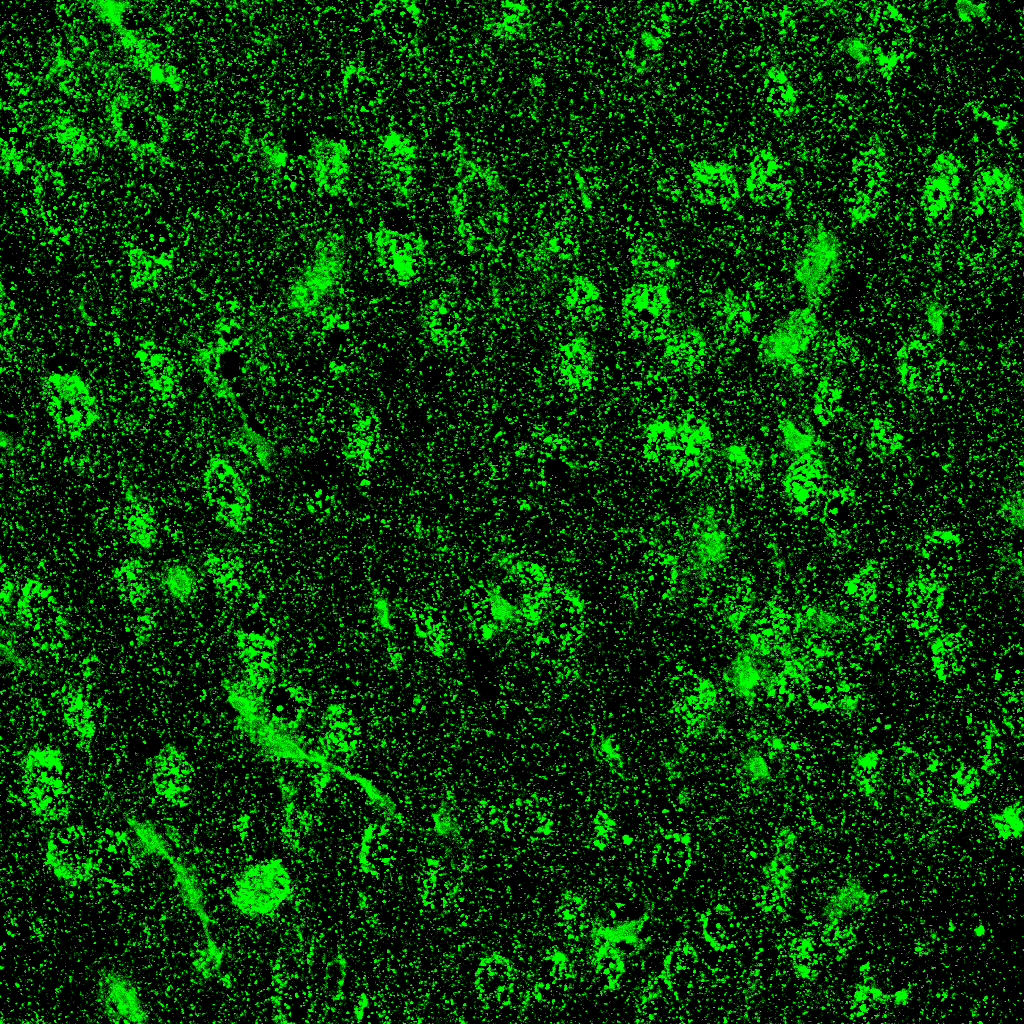** | | **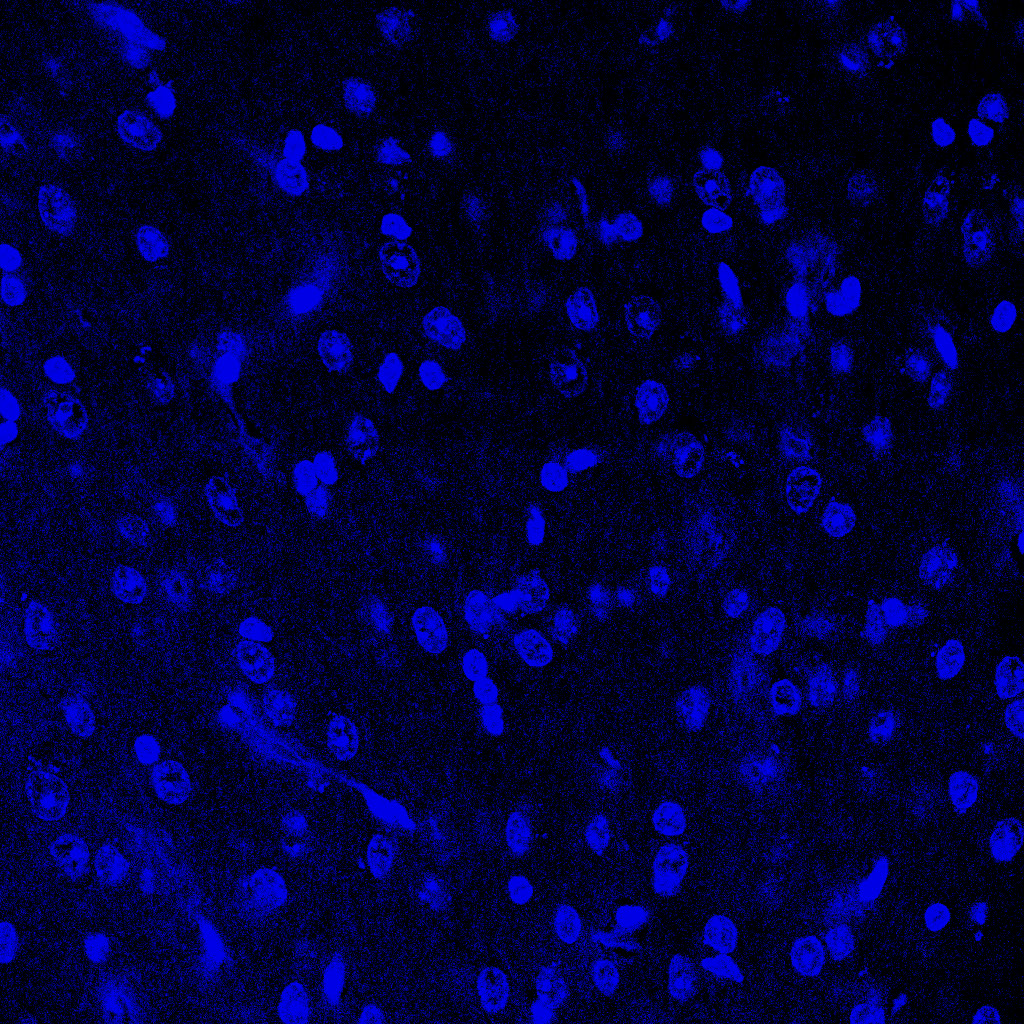** | **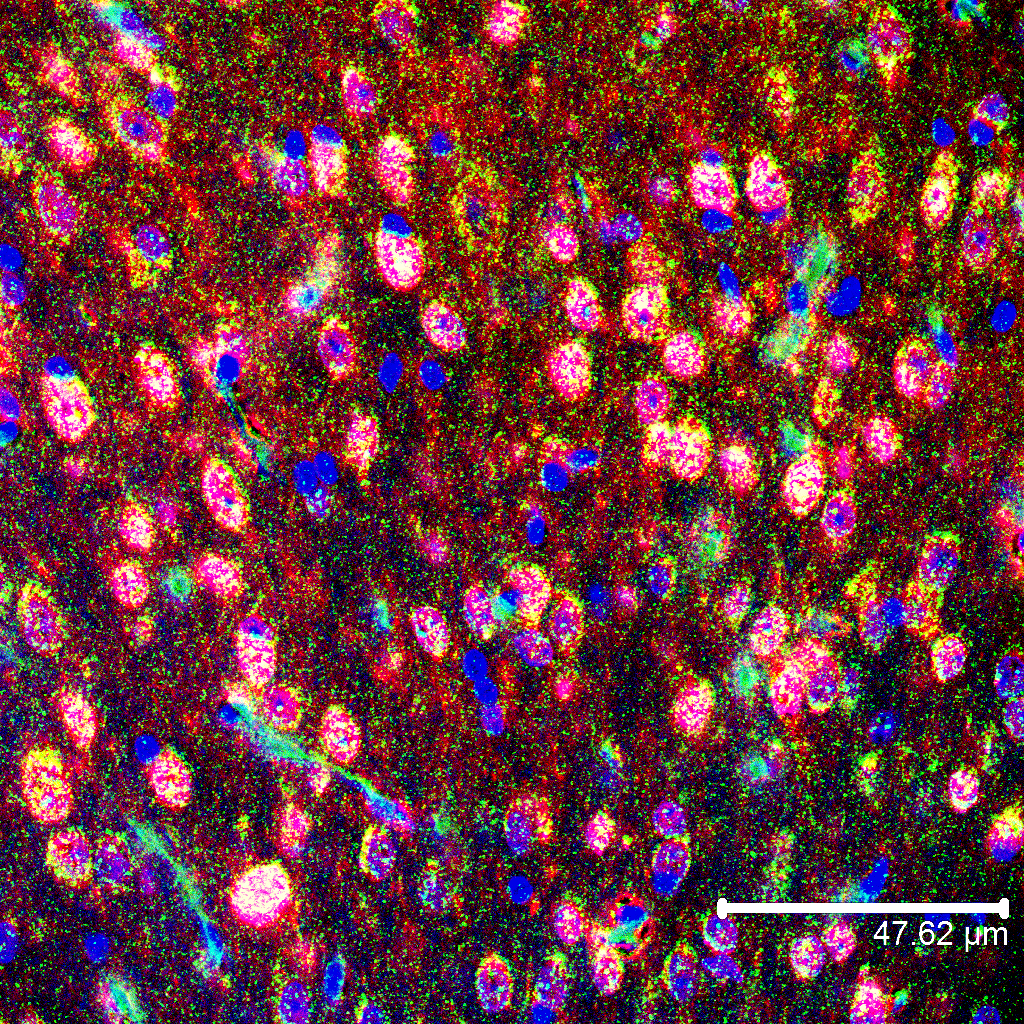** |
| **CCP + NK1.1** | **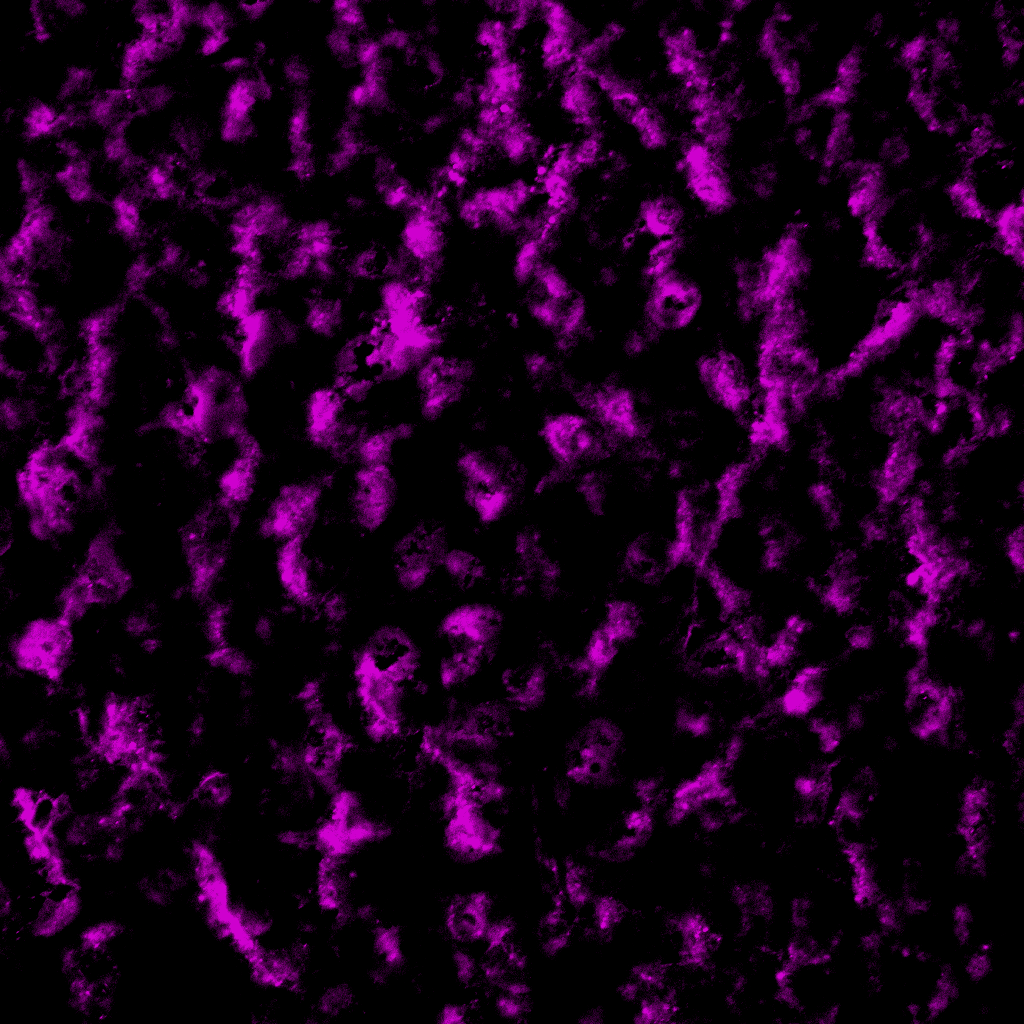** | **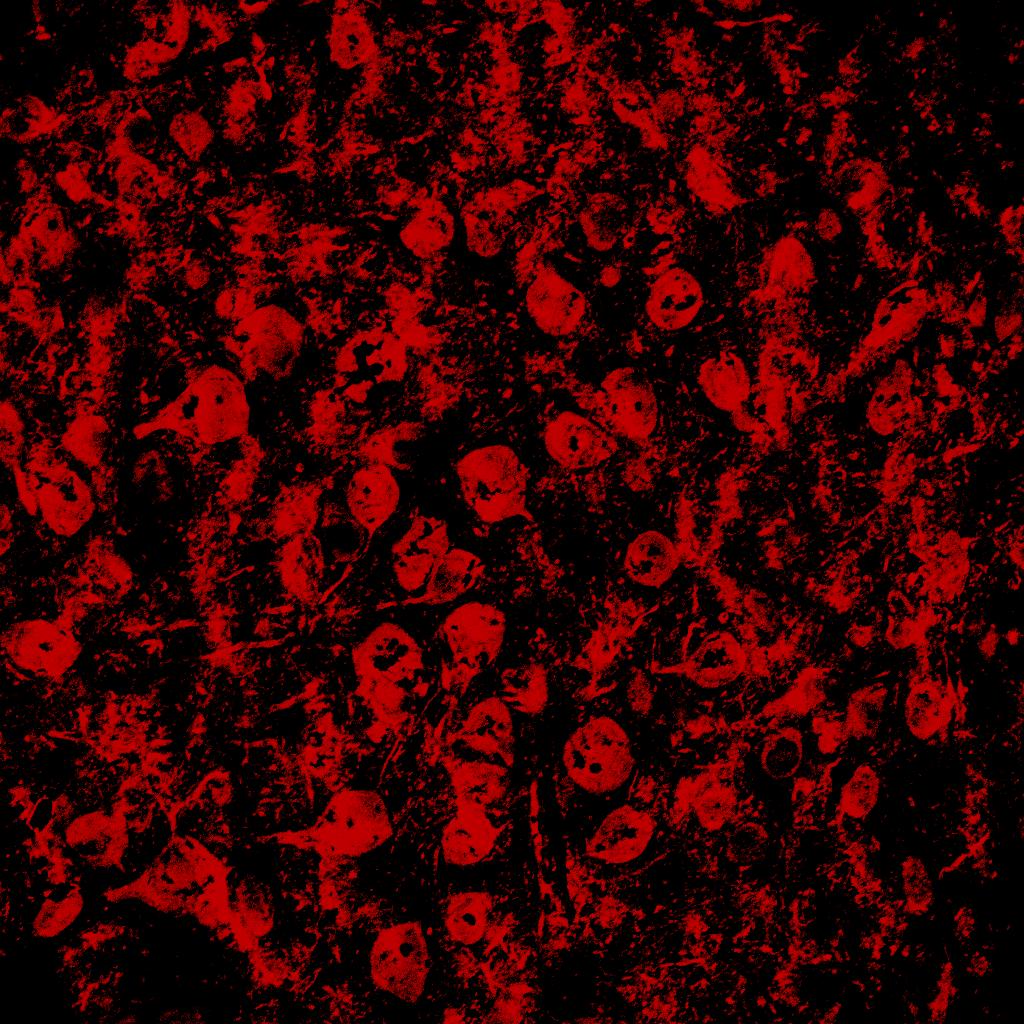** | **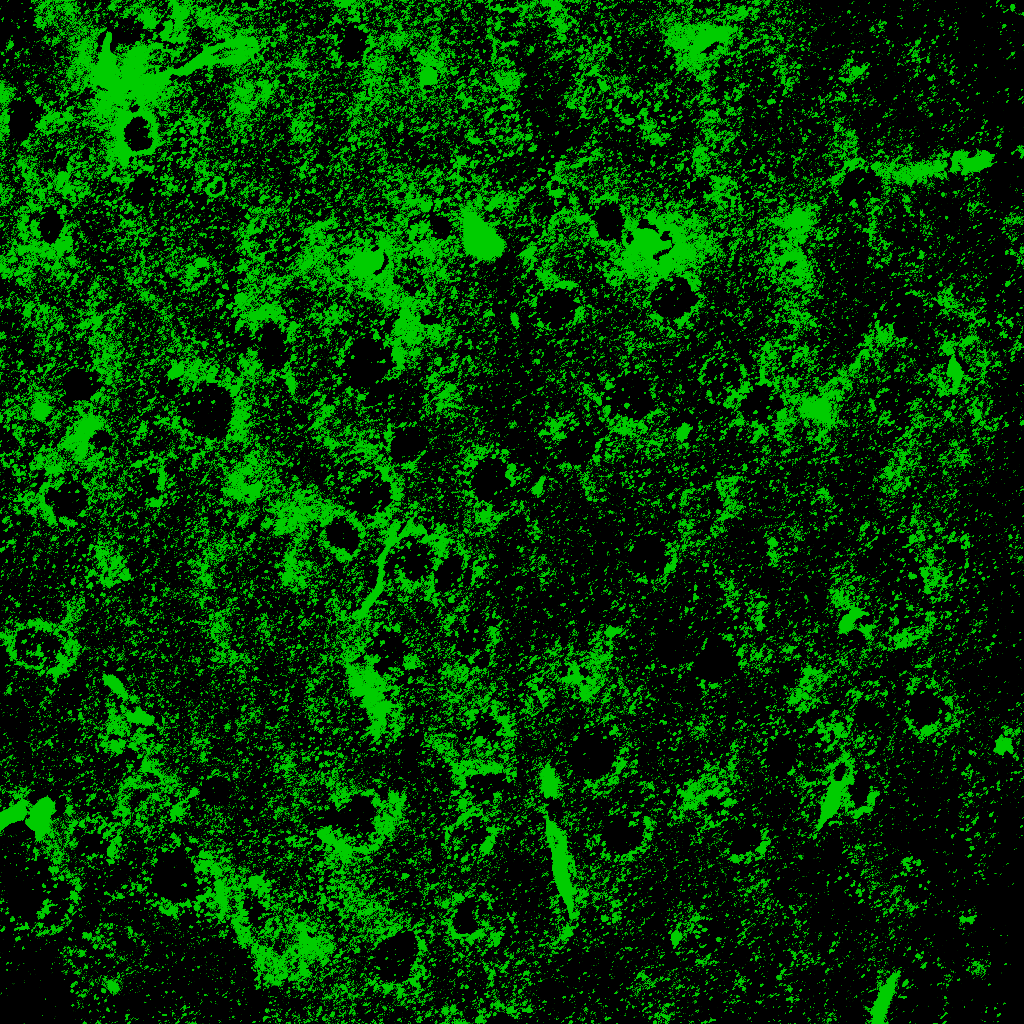** | | **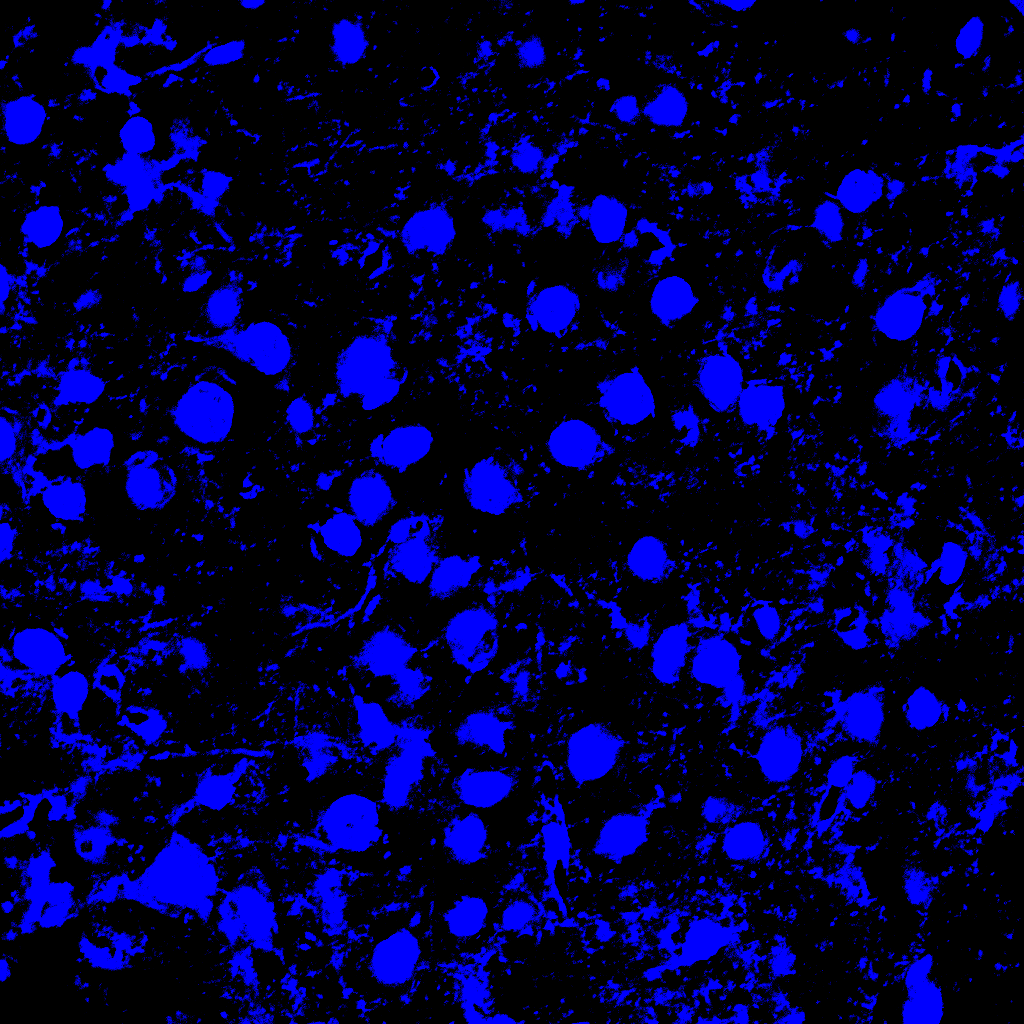** | **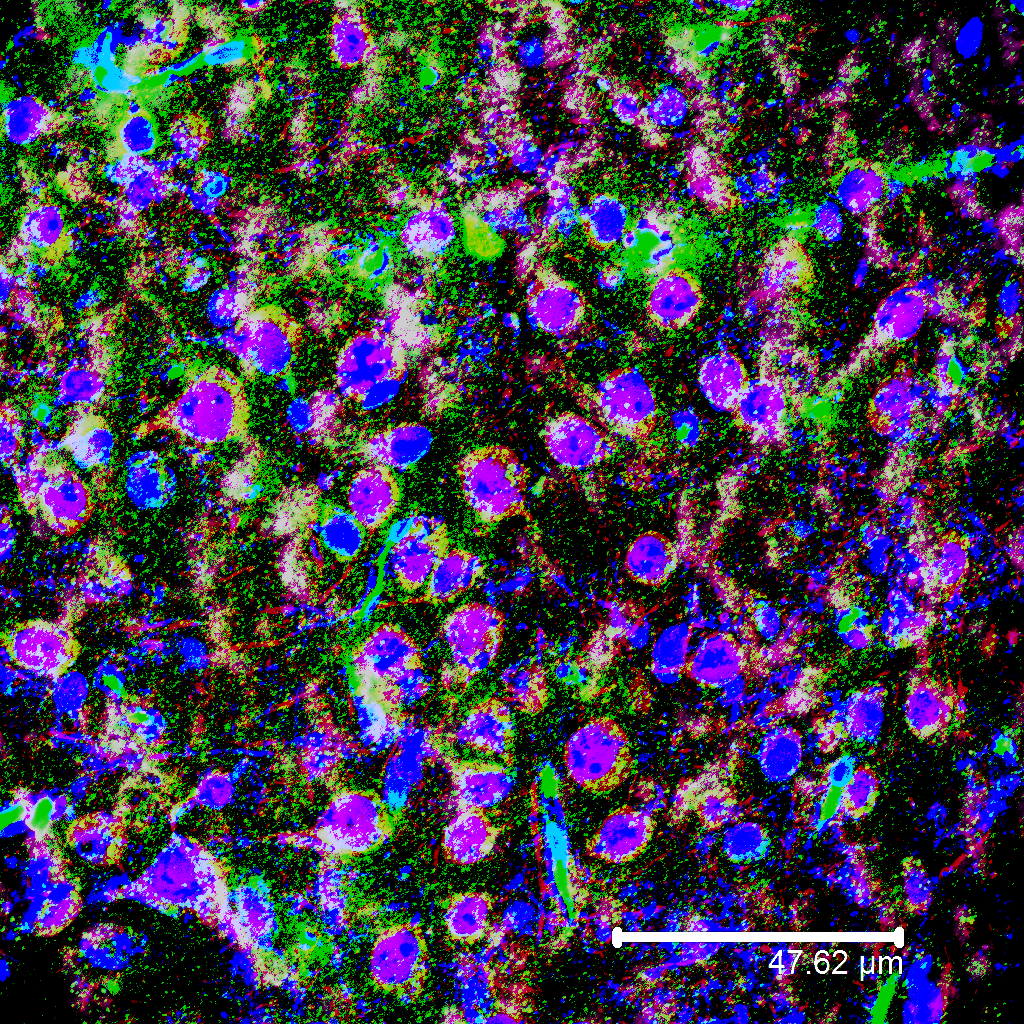** |
|  | **(B)**  **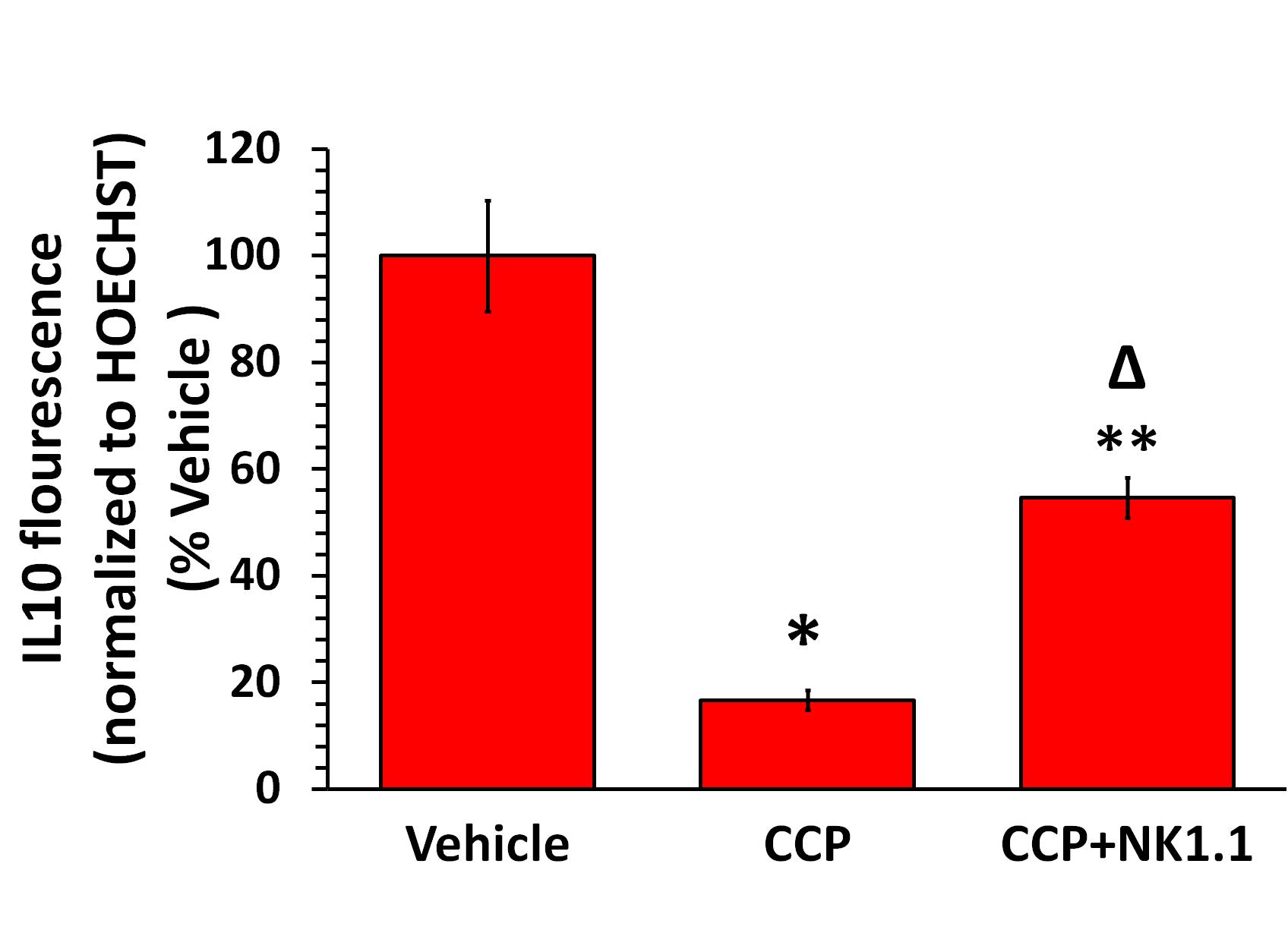** | | | **(C)**  **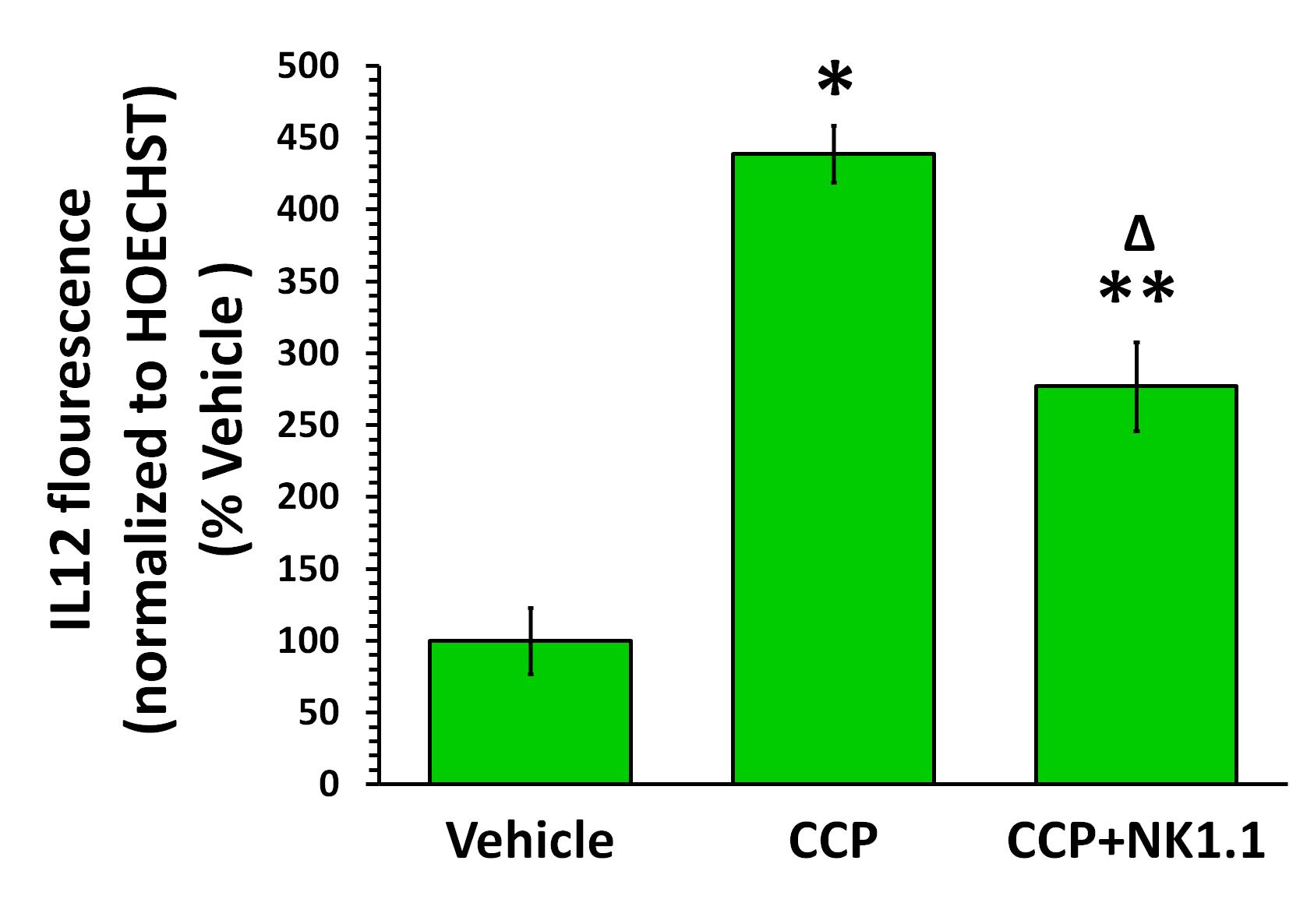** | | |

**Additional file 8: Figure S8. Abrogation of NK cells by peripheral infusion of NK1.1 antibody partially reverts the CCP-mediated suppression of IL10 and induction of IL12 in the TAM within the GBM mass.** As a corroboration of the flow cytometry data presented in Figure. 4, GBM brain sections parallel to those used in Figure S7, harboring the tumor from the three groups (Vehicle, CCP and CCP+NK1.1Ab) were triple-stained with antibodies against Iba1 (green), IL12 (red), and IL10 (purple). Sections from Vehicle-treated mice showed strong IL10 expression in the Iba1(+) TAM (**A** first row), which was suppressed by 83% in the CCP-treated mice (*p = 9.1x10^-8^, CCP versus Vehicle) (**A**, second row and **B**), but this CCP-evoked suppression of IL10 was only 45% in the CCP+NK1.1 sections (Δ p = 8.3x10^-8^, CCP+NK1.1 versus CCP; **p=2.7x10^-5^, CCP+NK1.1 versus Vehicle) (**A** third row and **B**). In contrast, IL12 expression in the Iba1(+) cells was very low in the sections from the Vehicle-treated mice (**A** first row), but it increased by 439% in the CCP-treated mice (*p = 1.3x10^-10^, CCP versus Vehicle) (**A** second row and **C**), and this increase was only 277% in the CCP+NK1.1 mice (Δ p= 9.3x10^-6^, CCP+NK1.1 versus CCP; **p=4.6x10^-7^, CCP+NK1.1 versus Vehicle) (**A** third row and **C**). Four sections per mouse from Vehicle (n=4), CCP (n=4), and CCP+NK1.1 (n=3) mice were used for imaging and each graph represents mean ± S.D. (Scale bar: 47.62 µm).
