# Supplementary material for: Phytosomal curcumin causes natural killer cell-dependent repolarization of glioblastoma (GBM) tumor-associated microglia/macrophages and elimination of GBM and GBM stem cells
Source: J Exp Clin Cancer Res. 2018 Jul 25;37:168. doi: 10.1186/s13046-018-0792-5 (PMC6058381; doi:10.1186/s13046-018-0792-5)
Supplement: Supplementary file 9 — Figure S9. Peripheral neutralization of NK cells partially reverses the CCP-mediated elimination of SOX2(+) GBM stem cells. (A) Multiple, randomly chosen GBM brain sections parallel to those used in Fig. S8, from the three groups (Vehicle, CCP and CCP + NK1.1Ab) were single-stained with an antibody against SOX2. The sections from the Vehicle-treated mice displayed significant number of SOX2(+) GBM stem cells (A first row). In contrast, the sections from CCP-treated mice showed a 79% suppression of SOX2(+) cells (*p = 8.6 × 10− 3, CCP versus Vehicle) (A, second row, and B). In contrast, the CCP-evoked suppression of SOX2(+) cells was only 54% in the sections from CCP + NK1.1 treated mice (Δ p = 0.015, CCP + NK1.1 versus CCP;**p = 0.025, CCP + NK1.1 versus Vehicle) (A, third row, and B). Three sections per mouse were used for imaging and the graphs represent mean ± S.D. obtained from Vehicle (n = 4), CCP (n = 4), and CCP + NK1.1 (n = 3). (Scale bar: 47.62 μm). (DOC 6174 kb) [file 13046_2018_792_MOESM9_ESM.doc]

| **(A)** | **SOX2** | **HOECHST** | **Merged** |
| --- | --- | --- | --- |
| **Vehicle** | **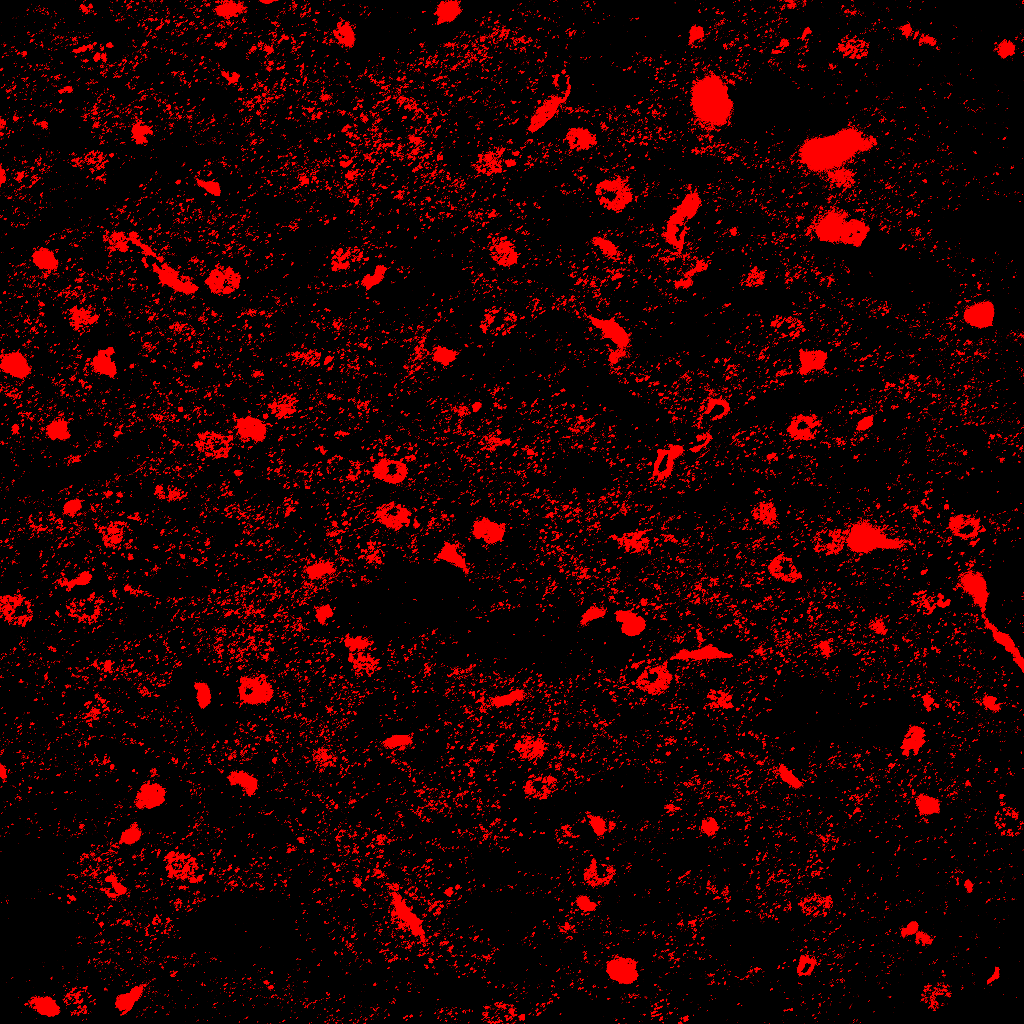** | **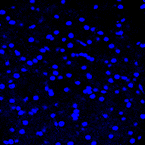** | **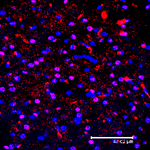** |
| **CCP** | **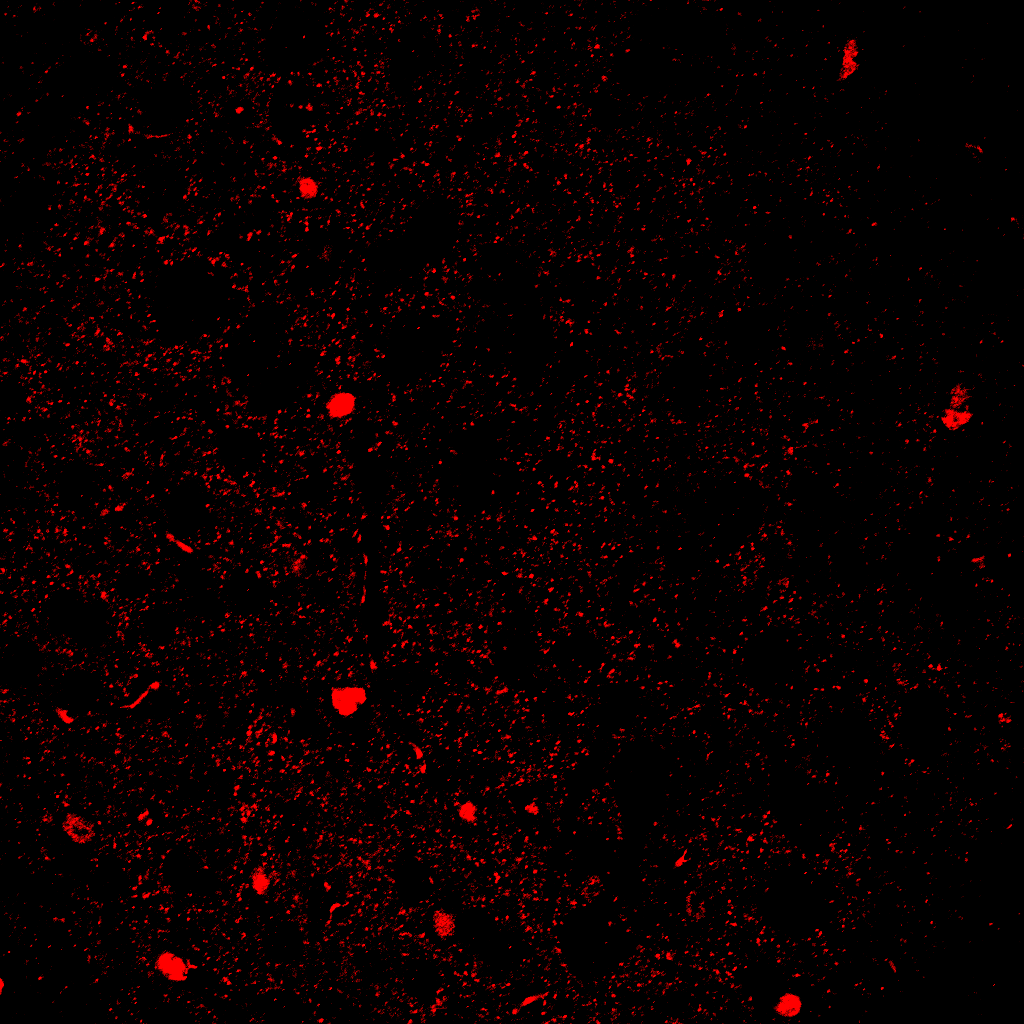** | **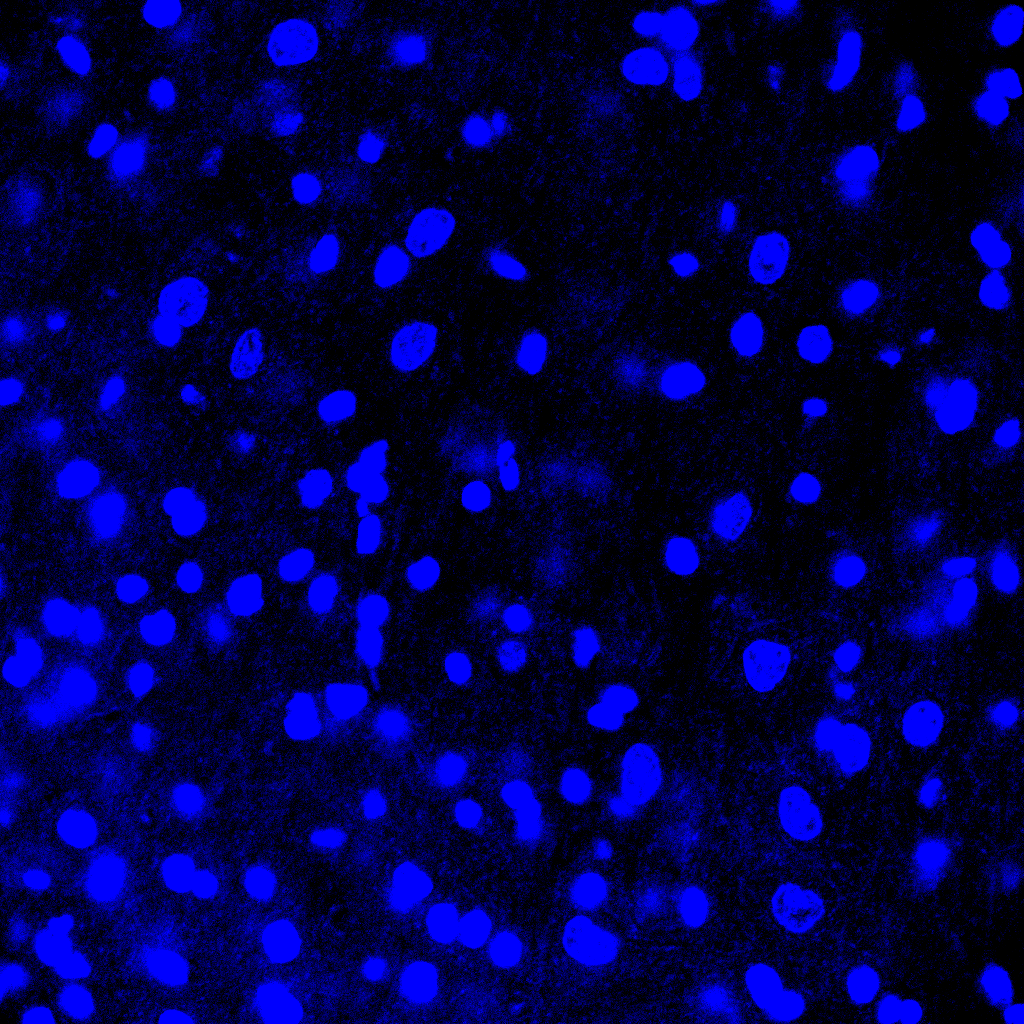** | **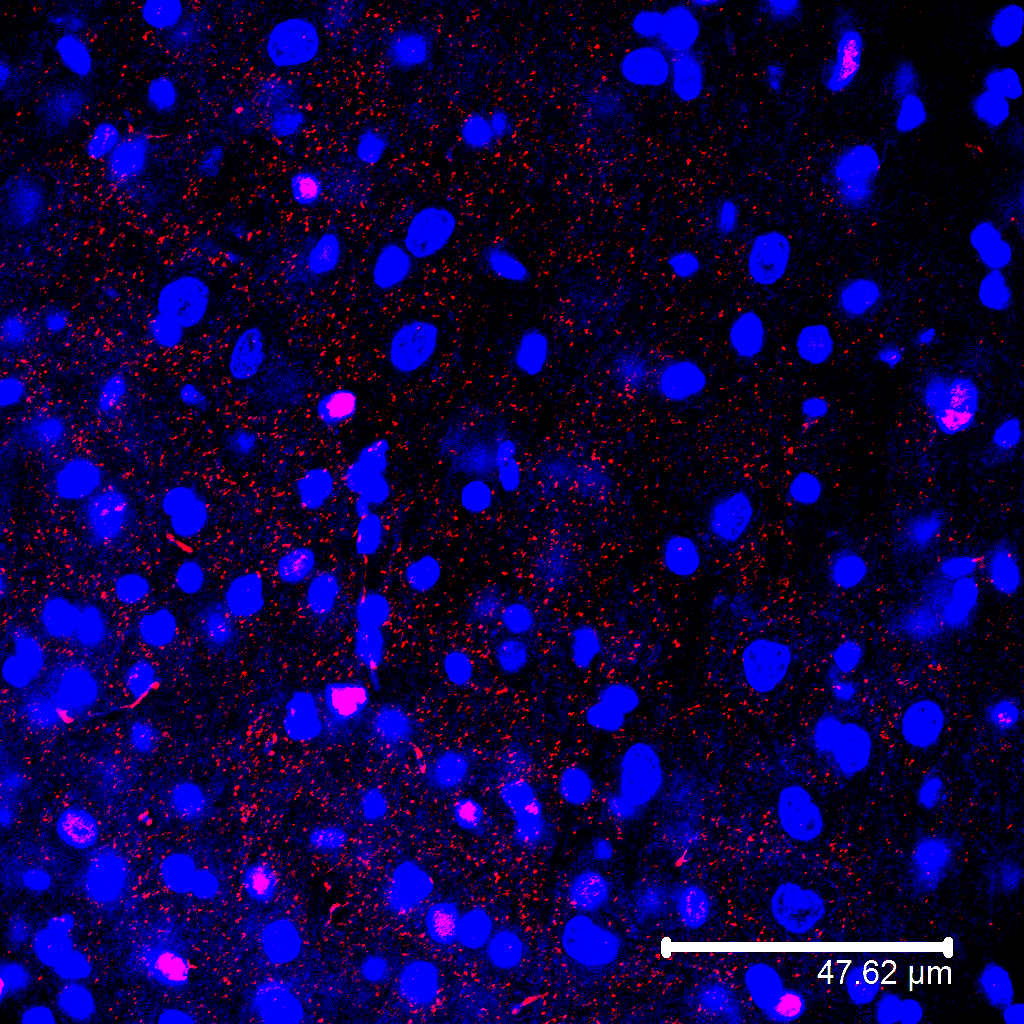** |
| **CCP + NK1.1Ab** | **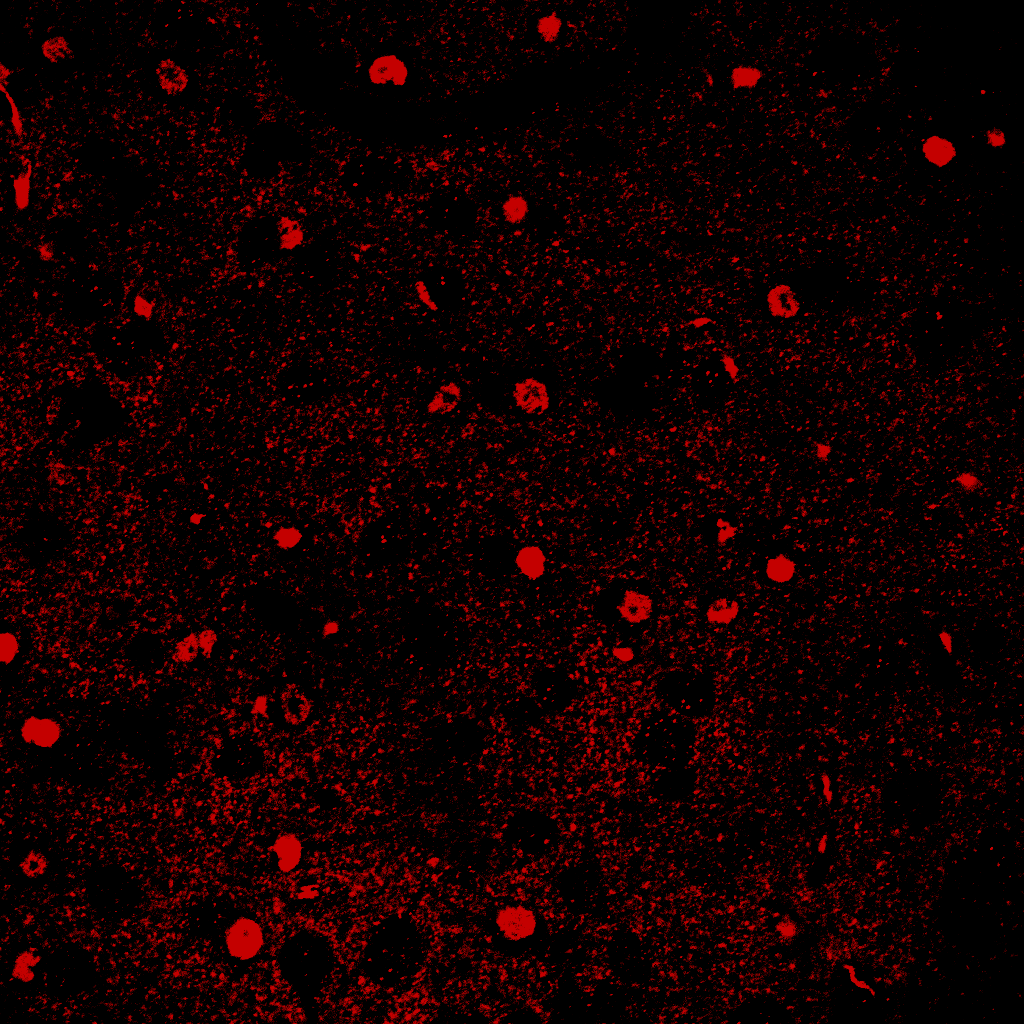** | **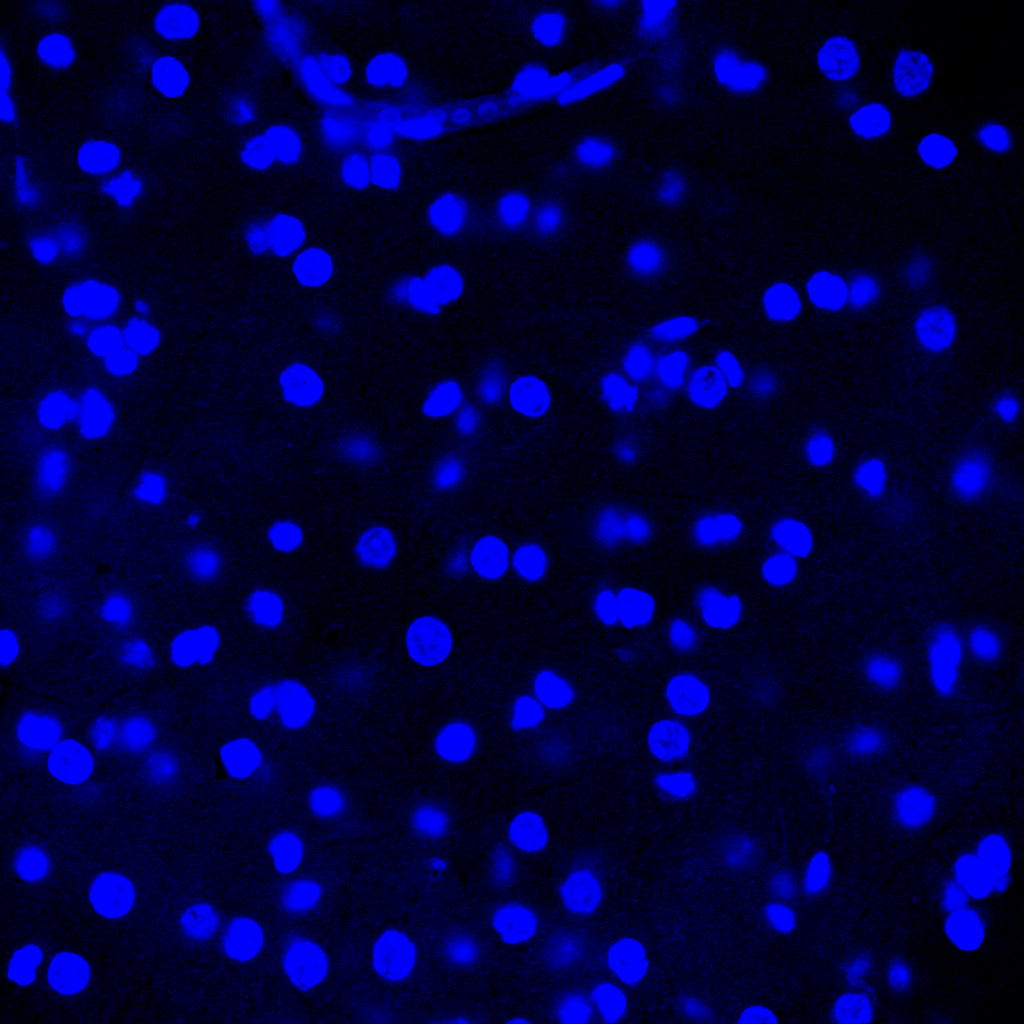** | **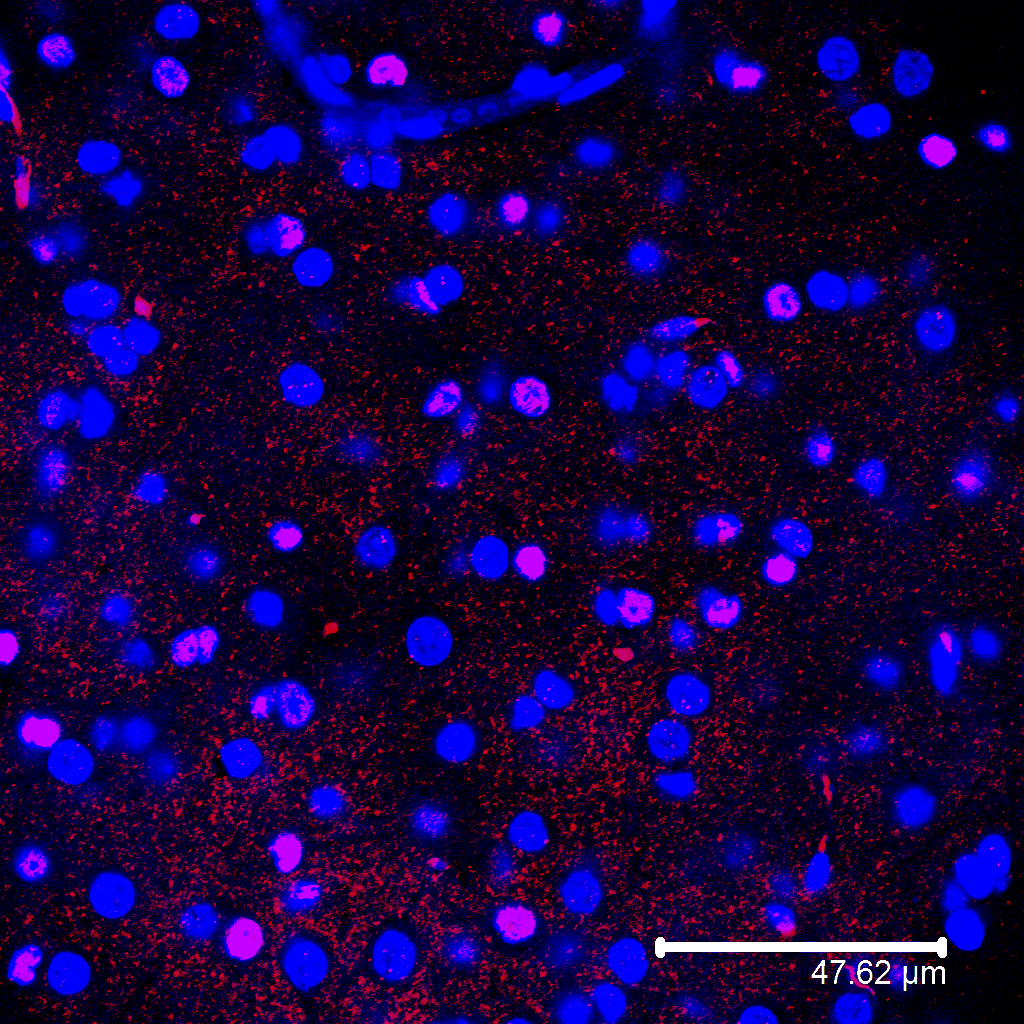** |
| **(B)**  **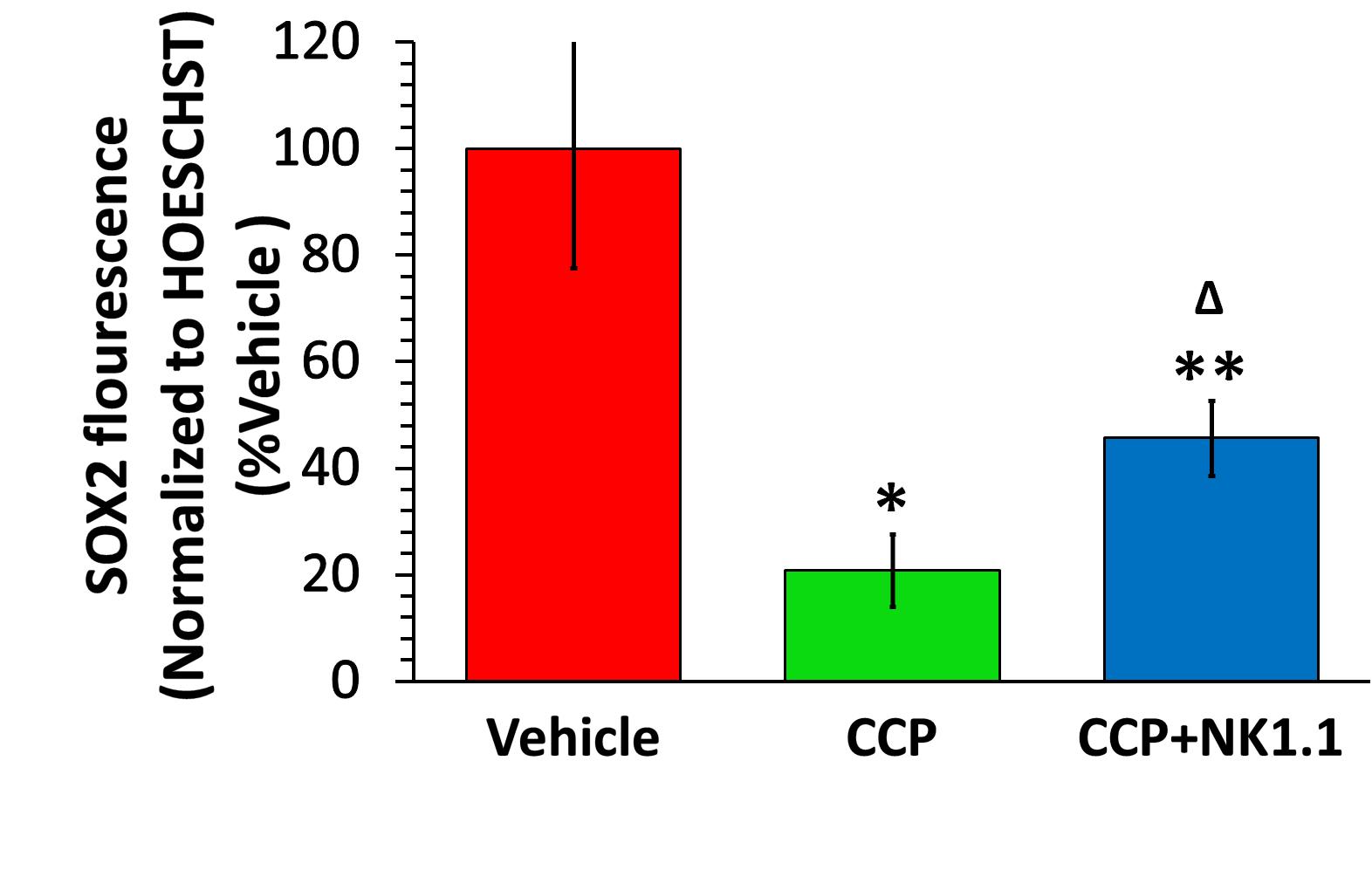** | | | |

**Additional file 9: Figure S9. Peripheral neutralization of NK cells partially reverses the CCP-mediated elimination of SOX2(+) GBM stem cells. (A)** Multiple, randomly chosen GBM brain sections parallel to those used in Figure S8, from the three groups (Vehicle, CCP and CCP+NK1.1Ab) were single-stained with an antibody against SOX2. The sections from the Vehicle-treated mice displayed significant number of SOX2(+) GBM stem cells (**A** first row). In contrast, the sections from CCP-treated mice showed a 79% suppression of SOX2(+) cells (*p = 8.6x10^-3^, CCP versus Vehicle) (**A**, second row, and **B**). In contrast, the CCP-evoked suppression of SOX2(+) cells was only 54% in the sections from CCP+NK1.1 treated mice (Δ p = 0.015, CCP+NK1.1 versus CCP ;**p = 0.025, CCP+NK1.1 versus Vehicle) (**A**, third row, and **B**). Three sections per mouse were used for imaging and the graphs represent mean ± S.D. obtained from Vehicle (n=4), CCP (n=4), and CCP+NK1.1 (n=3). (Scale bar: 47.62 µm).
