# Supplementary material for: Phytosomal curcumin causes natural killer cell-dependent repolarization of glioblastoma (GBM) tumor-associated microglia/macrophages and elimination of GBM and GBM stem cells
Source: J Exp Clin Cancer Res. 2018 Jul 25;37:168. doi: 10.1186/s13046-018-0792-5 (PMC6058381; doi:10.1186/s13046-018-0792-5)
Supplement: Supplementary file 10 — Figure S10. Peripheral pre-treatment with IL12 antibody partially eliminates the CCP-mediated M1-like phenotype of the TAM in GBM tumor. GBM Brain sections parallel to the dispersed cells used in Fig. S4 from the Vehicle, CCP and CCP + IL12Ab groups were used to assess and quantify the expression of iNOS on tumor associated microglia (Iba1(+)/RM0029-11H3(−)) and macrophages (Iba1(+)/RM0029-11H3(+)) upon CCP and CCP + IL12Ab treatment. (A) The GBM sections from the Vehicle-treated mice harbored mostly tumor-associated microglia and few macrophages (first row) which showed sparse iNOS staining. The CCP (second row) treatment showed copious presence of both iNOS+ intra-GBM recruited tumor-associated macrophages and resident tumor-associated microglia. The CCP + IL12Ab-treated (third row) mice showed intermediate levels of iNOS in the macrophages and microglia. (B) (Left) CCP-treatment caused a 474% increase in the intensity of microglia-associated iNOS (fluorescence intensity normalized to the number of cells) (*p = 8.8 × 10− 6 Vehicle versus CCP), while CCP + IL12Ab treatment reduced this augmentation to 242% with respect to the Vehicle (**p = 1.2 × 10− 3 Vehicle versus CCP + IL12Ab; ∆ p = 4.1 × 10− 3 CCP versus CCP + IL12Ab). (Right) CCP-treatment induced a 498% increase in macrophage-associated iNOS intensity (*p = 5.4 × 10− 5 Vehicle versus CCP), whereas the CCP + IL12Ab group showed a partial reversal of this increase to 250% with respect to the Vehicle (**p = 5.4 × 10− 5 Vehicle versus CCP + IL12Ab; ∆ p = 3.5 × 10− 4 CCP versus CCP + IL12Ab). Four sections per mouse were used for imaging and counting and the graphs represent mean ± S.D. obtained from Vehicle (n = 3), CCP (n = 3), and CCP + IL12Ab (n = 3). (Scale bar: 47.62 μm). (DOC 4392 kb) [file 13046_2018_792_MOESM10_ESM.doc]

| **(A)** | **RM0029-11H3** | **Iba1** | **iNOS** | **HOECHST** | **Merged** |
| --- | --- | --- | --- | --- | --- |
| **Vehicle** | **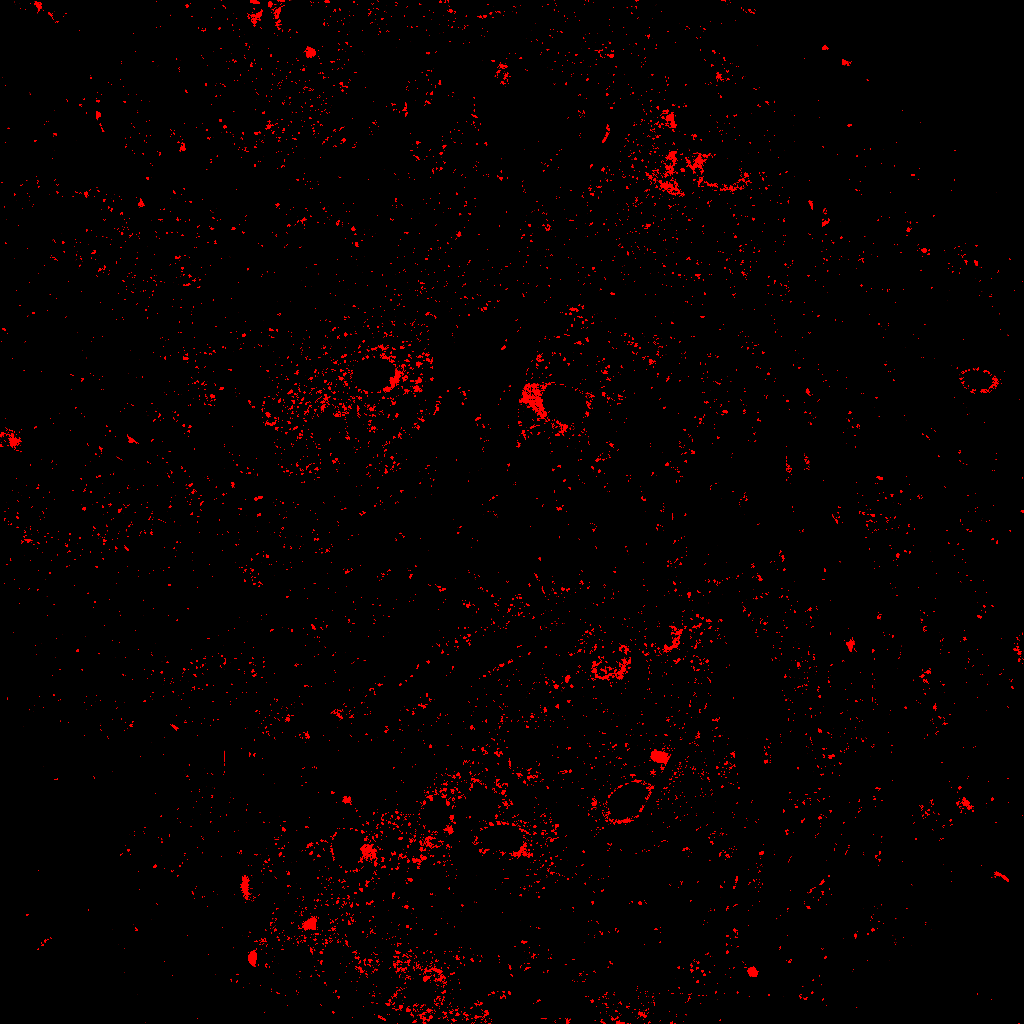** | **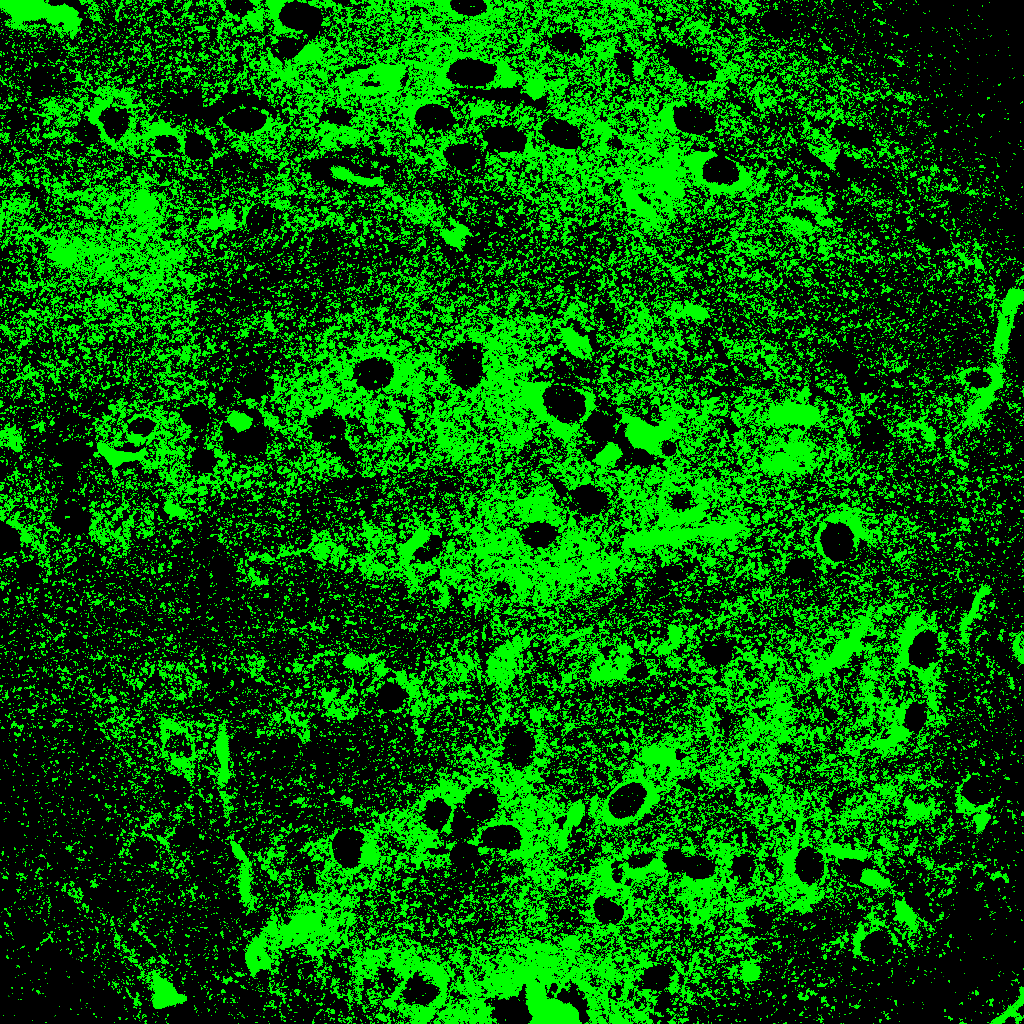** | **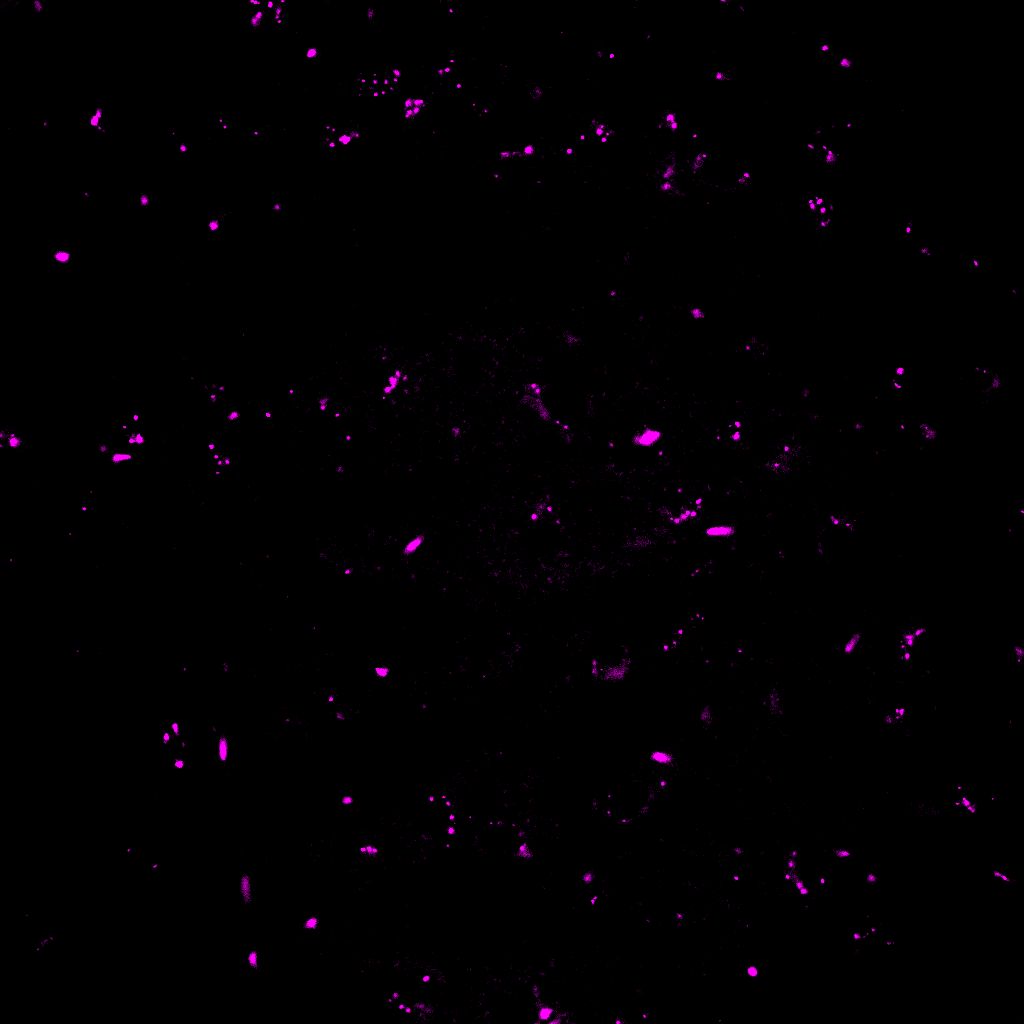** | **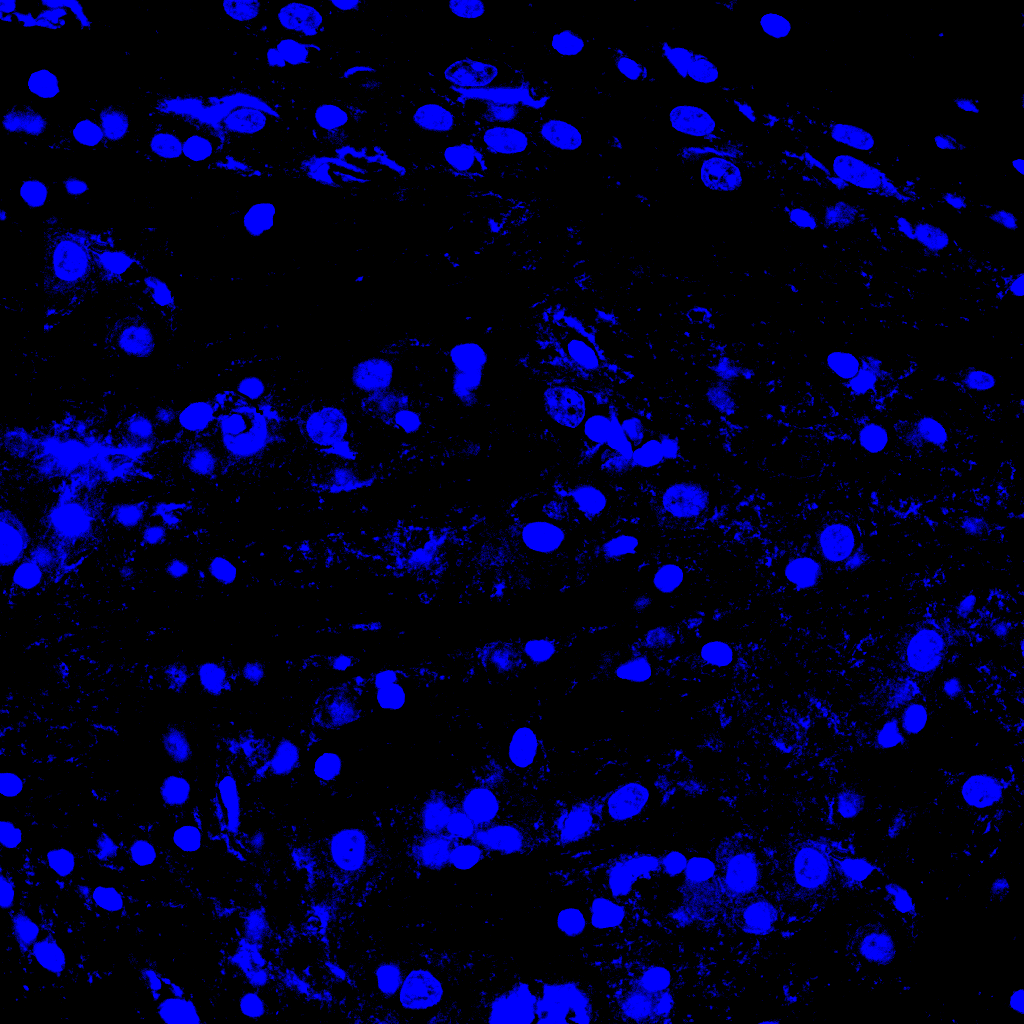** | **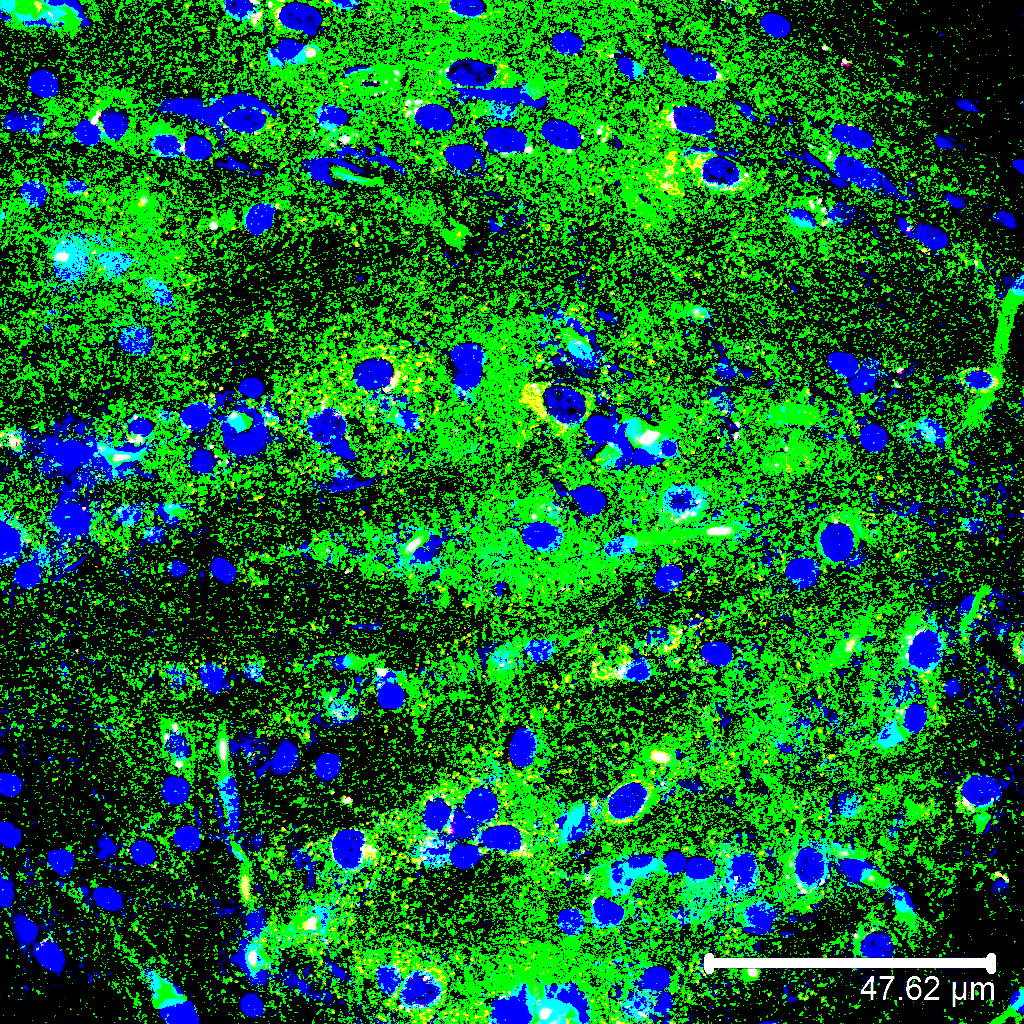** |
| **CCP** | **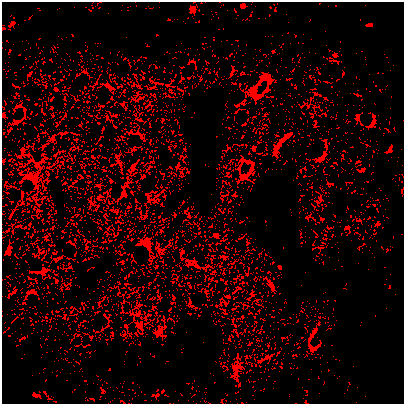** | **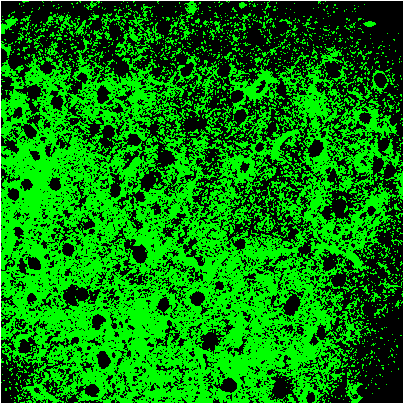** | **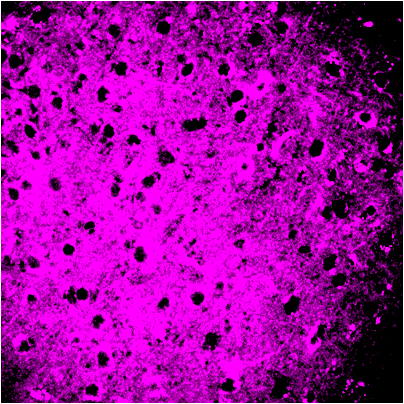** | **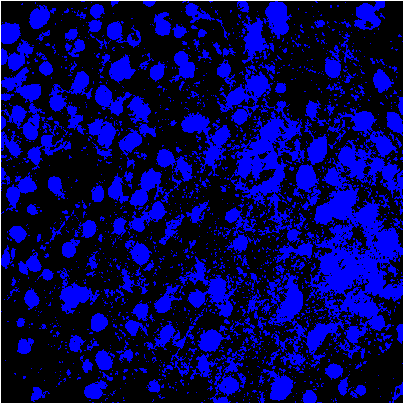** | **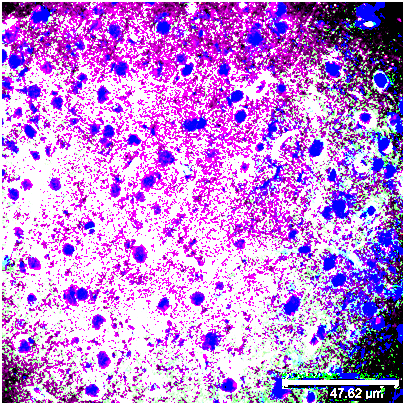** |
| **CCP + IL12Ab** | **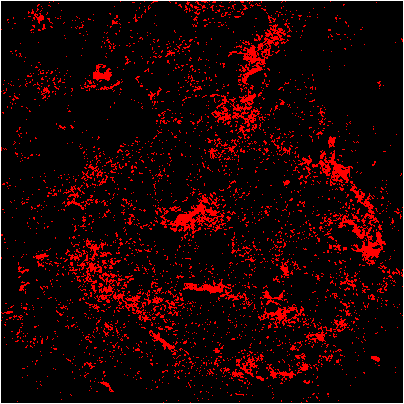** | **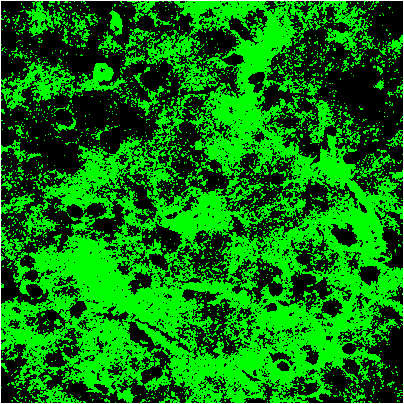** | **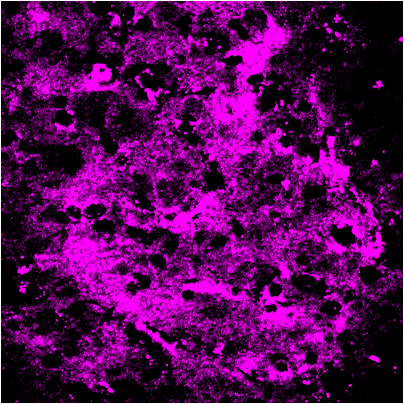** | **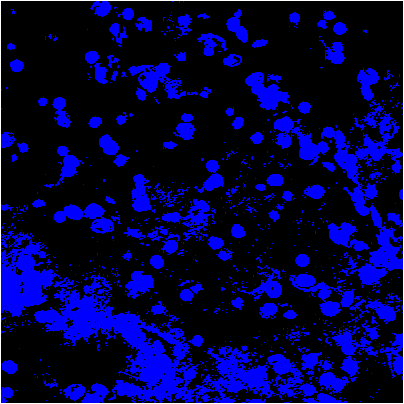** | **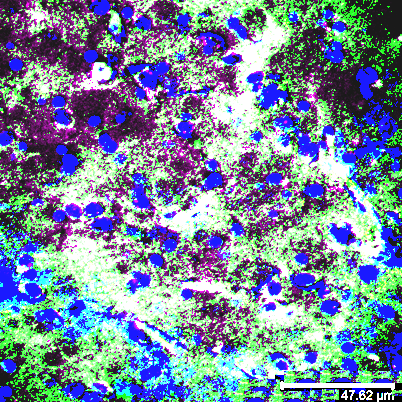** |
| **(B)**  **Microglia Macrophages**  **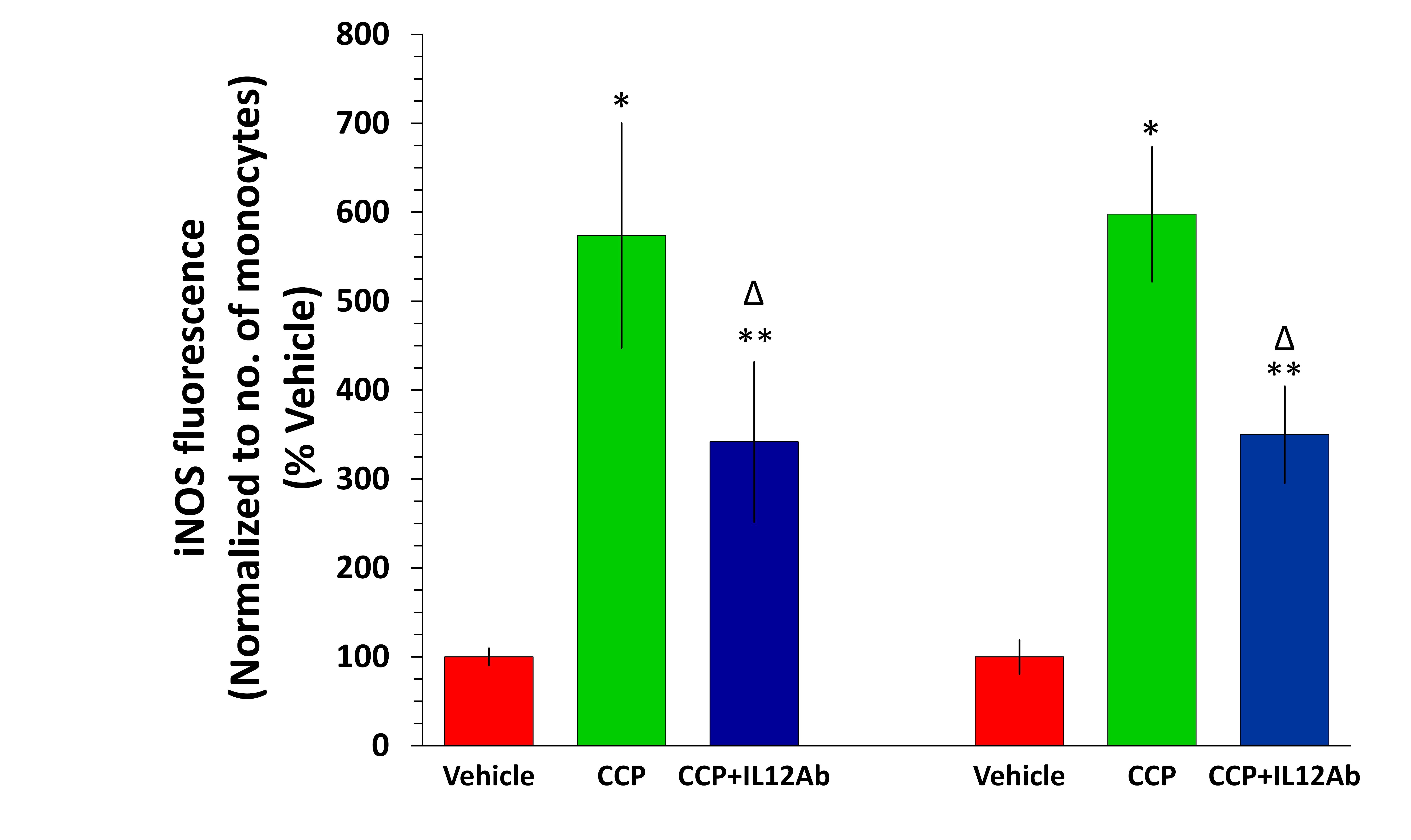** | | | | | |

**Additional file 10: Figure S10. Peripheral pre-treatment with IL12 antibody partially eliminates the CCP-mediated M1-like phenotype of the TAM in GBM tumor.** GBM Brain sections parallel to the dispersed cells used in Figure S4 from the Vehicle, CCP and CCP+IL12Ab groups were used to assess and quantify the expression of iNOS on tumor associated microglia (Iba1(+)/RM0029-11H3(-)) and macrophages (Iba1(+)/RM0029-11H3(+)) upon CCP and CCP+IL12Ab treatment. **(A)** The GBM sections from the Vehicle-treated mice harbored mostly tumor-associated microglia and few macrophages (first row) which showed sparse iNOS staining. The CCP (second row) treatment showed copious presence of both iNOS+ intra-GBM recruited tumor-associated macrophages and resident tumor-associated microglia. The CCP+IL12Ab-treated (third row) mice showed intermediate levels of iNOS in the macrophages and microglia. **(B) (Left)** CCP-treatment caused a 474% increase in the intensity of microglia-associated iNOS (fluorescence intensity normalized to the number of cells) (*p = 8.8x10^-6^ Vehicle versus CCP), while CCP+IL12Ab treatment reduced this augmentation to 242% with respect to the Vehicle (**p = 1.2x10^-3^ Vehicle versus CCP+IL12Ab; ∆ p = 4.1x10^-3^ CCP versus CCP+IL12Ab). **(Right)** CCP-treatment induced a 498% increase in macrophage-associated iNOS intensity (*p = 5.4x10^-5^ Vehicle versus CCP), whereas the CCP+IL12Ab group showed a partial reversal of this increase to 250% with respect to the Vehicle (**p = 5.4x10^-5^ Vehicle versus CCP+IL12Ab; ∆ p = 3.5x10^-4^ CCP versus CCP+IL12Ab). Four sections per mouse were used for imaging and counting and the graphs represent mean ± S.D. obtained from Vehicle (n=3), CCP (n=3), and CCP+IL12Ab (n=3). (Scale bar: 47.62 µm.).
