# Supplementary material for: Phytosomal curcumin causes natural killer cell-dependent repolarization of glioblastoma (GBM) tumor-associated microglia/macrophages and elimination of GBM and GBM stem cells
Source: J Exp Clin Cancer Res. 2018 Jul 25;37:168. doi: 10.1186/s13046-018-0792-5 (PMC6058381; doi:10.1186/s13046-018-0792-5)
Supplement: Supplementary file 11 — Figure S11. CCP treatment causes a dramatic induction of MCP-1 in the Iba1(+) TAM. Sections made from GBM brain tissues parallel to the dispersed cells used in Fig. S10, from the three groups (Vehicle, CCP and CCP + IL12Ab) were immunostained to assess the expression of MCP-1 on the Iba1(+) TAM. (A) The GBM sections from the Vehicle-treated mice harbored mostly tumor-associated microglia and few macrophages (first row) which expressed very little MCP-1, whereas the tumors from both the CCP (second row) and CCP + IL12Ab-treated (third row) mice showed both recruited tumor-associated macrophages and resident tumor-associated microglia, both strongly expressing MCP-1. (B) CCP-treatment triggered a 374% increase in the MCP-1 fluorescence in the microglia (*p = 2.6 × 10− 5 Vehicle versus CCP), whereas CCP + IL12Ab-treatment showed 323% increase in MCP-1 intensity (**p = 6.5 × 10− 6 Vehicle versus CCP + IL12Ab). No significant difference was observed between the CCP and CCP + IL12Ab groups. Four sections per mouse were used for imaging and counting and the graph represent mean ± S.D. (fluorescence intensity normalized to the number of cells) obtained from Vehicle (n = 3), CCP (n = 3), and CCP + IL12Ab (n = 3). (Scale bar: 47.62 μm). We have shown earlier that CCP-treatment of mice causes an induction of activated, p65 NF-kB in GBM TAM [8, 27]. Additionally, p65 NF-kB has been shown to induce MCP-1 expression [81], which is most likely the mechanism of CCP-mediated induction of MCP-1 in the GBM TAM in these mice. (DOC 8284 kb) [file 13046_2018_792_MOESM11_ESM.doc]

**Additional file 11: Figure S11.** **CCP treatment causes a dramatic induction of MCP-1 in the Iba1(+) TAM.** Sections made from GBM brain tissues parallel to the dispersed cells used in Figure S10, from the three groups (Vehicle, CCP and CCP+IL12Ab) were immunostained to assess the expression of MCP-1 on the Iba1(+) TAM. **(A)** The GBM sections from the Vehicle-treated mice harbored mostly tumor-associated microglia and few macrophages (first row) which expressed very little MCP-1, whereas the tumors from both the CCP (second row) and CCP+IL12Ab-treated (third row) mice showed both recruited tumor-associated macrophages and resident tumor-associated microglia, both strongly expressing MCP-1. **(B)** CCP-treatment triggered a 374% increase in the MCP-1 fluorescence in the microglia (*p = 2.6 x 10^-5^ Vehicle versus CCP), whereas CCP+IL12Ab-treatment showed 323% increase in MCP-1 intensity (**p = 6.5x10^-6^ Vehicle versus CCP+IL12Ab). No significant difference was observed between the CCP and CCP+IL12Ab groups. Four sections per mouse were used for imaging and counting and the graph represent mean ± S.D. (fluorescence intensity normalized to the number of cells) obtained from Vehicle (n=3), CCP (n=3), and CCP+IL12Ab (n=3). (Scale bar: 47.62 µm).

| **(A)** | **Iba1** | **MCP-1** | **HOECHST** | **Merged** |
| --- | --- | --- | --- | --- |
| **Vehicle** | **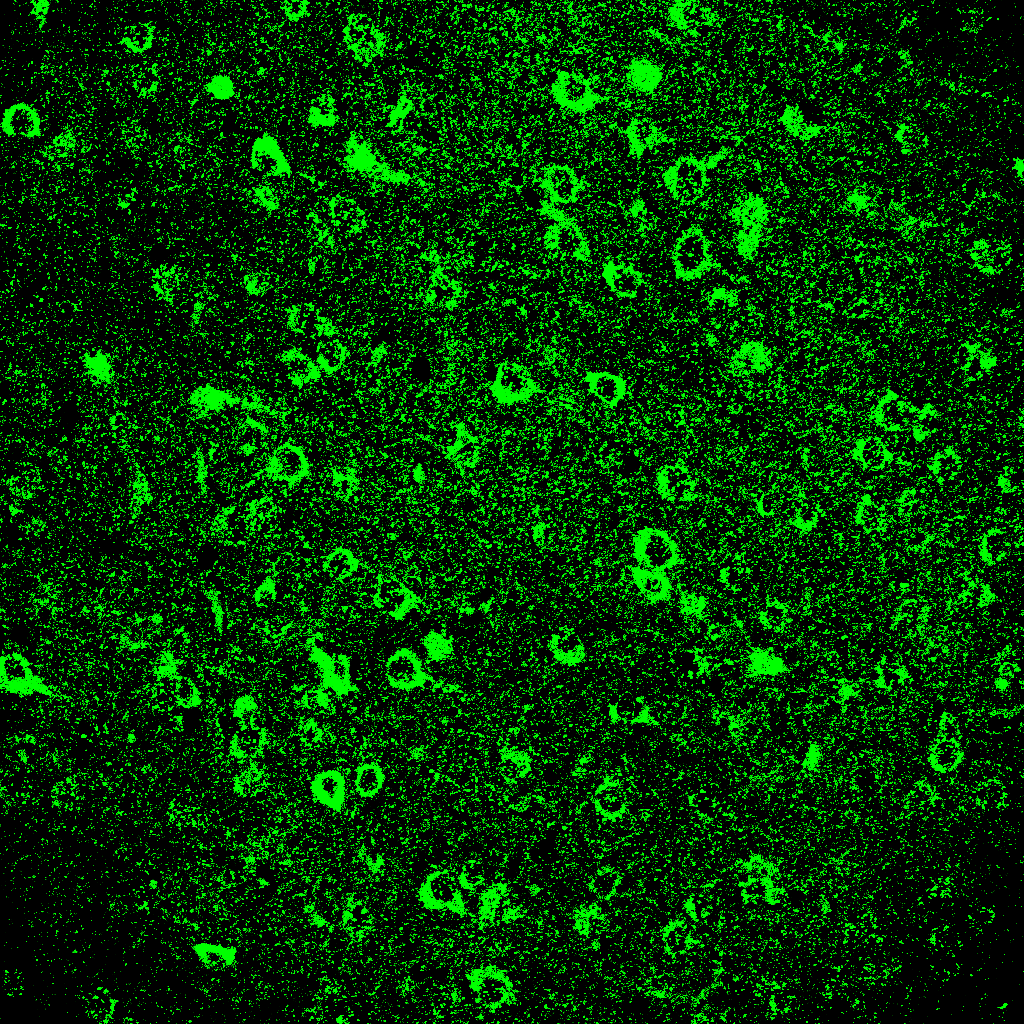** | **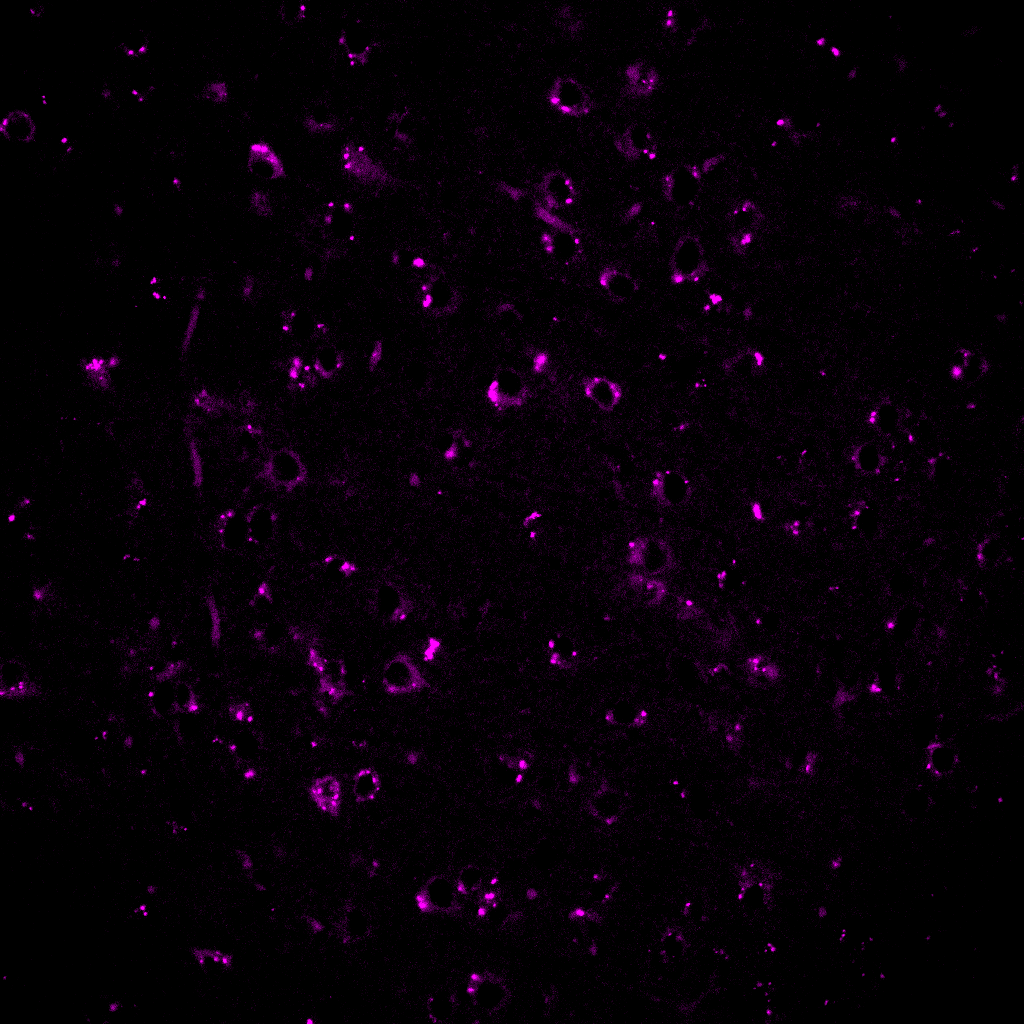** | **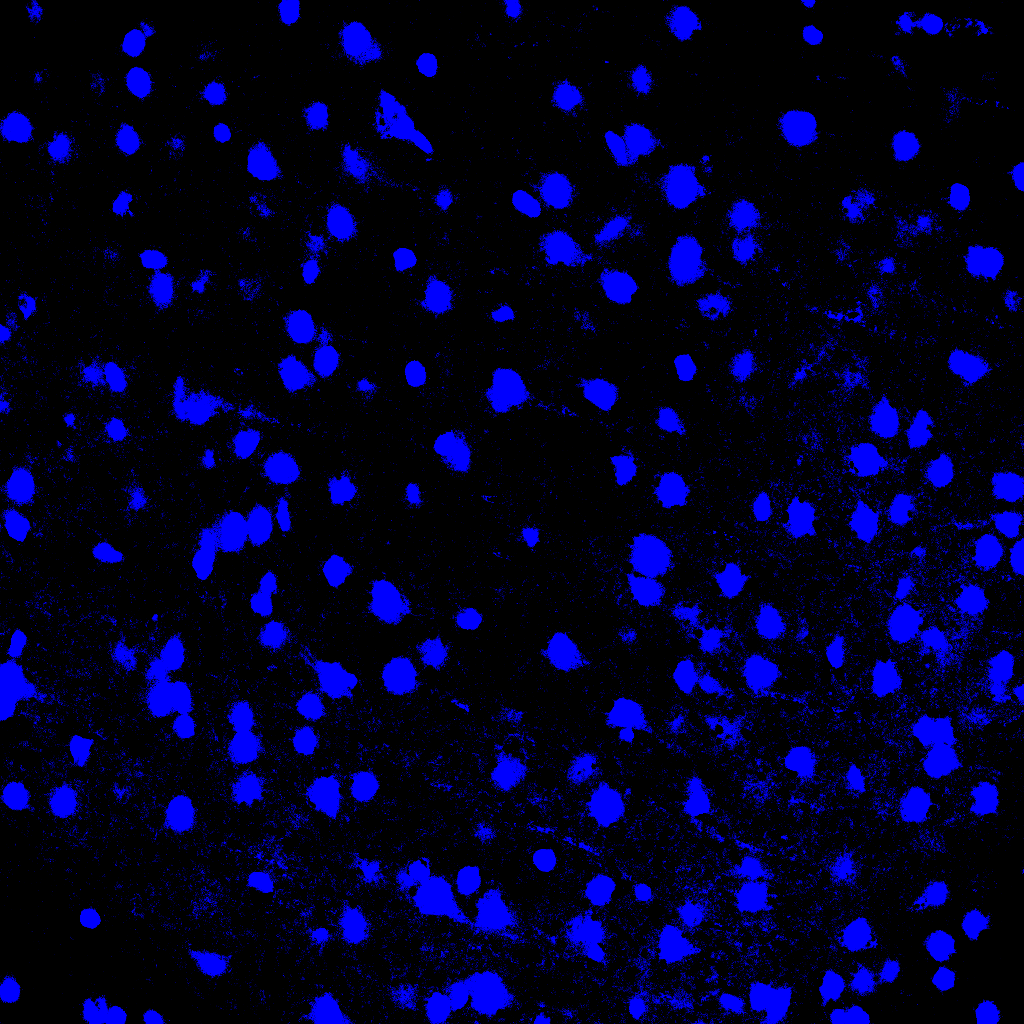** | **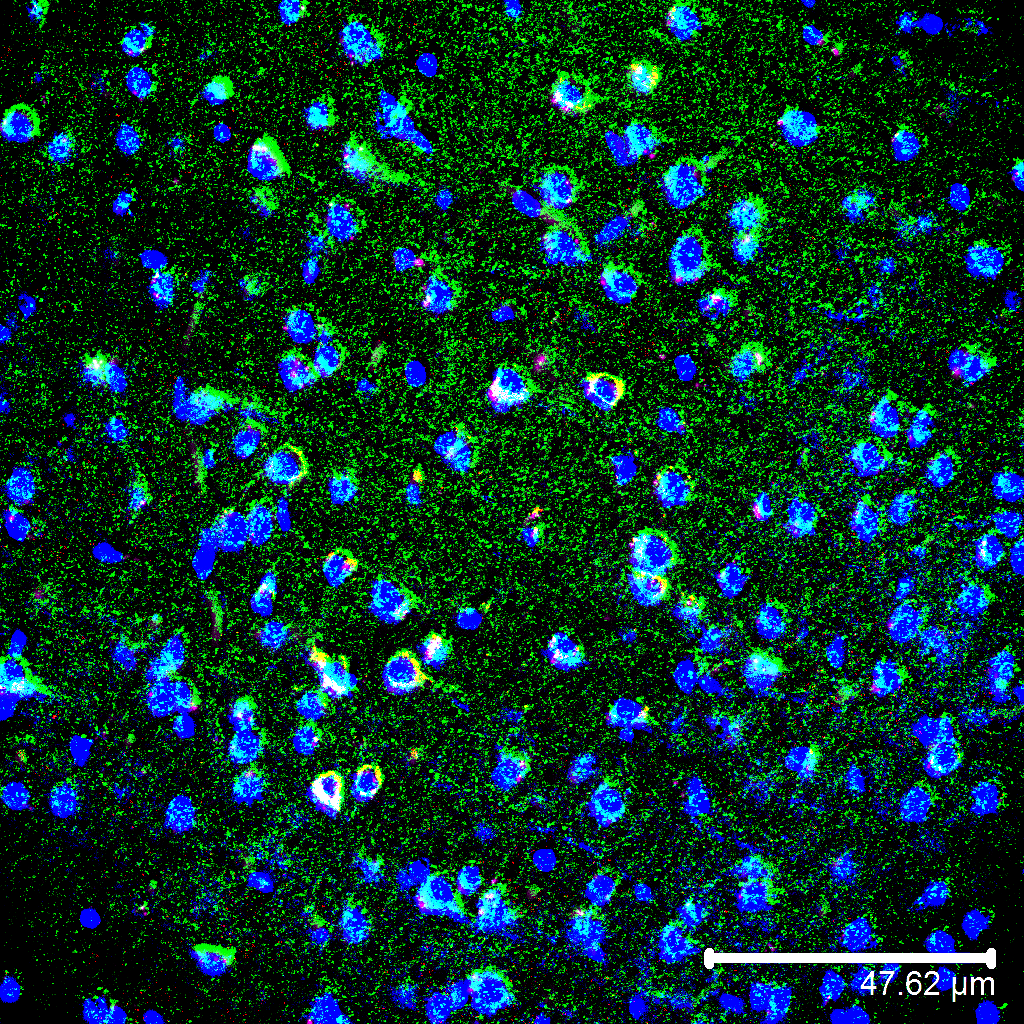** |
| **CCP** | **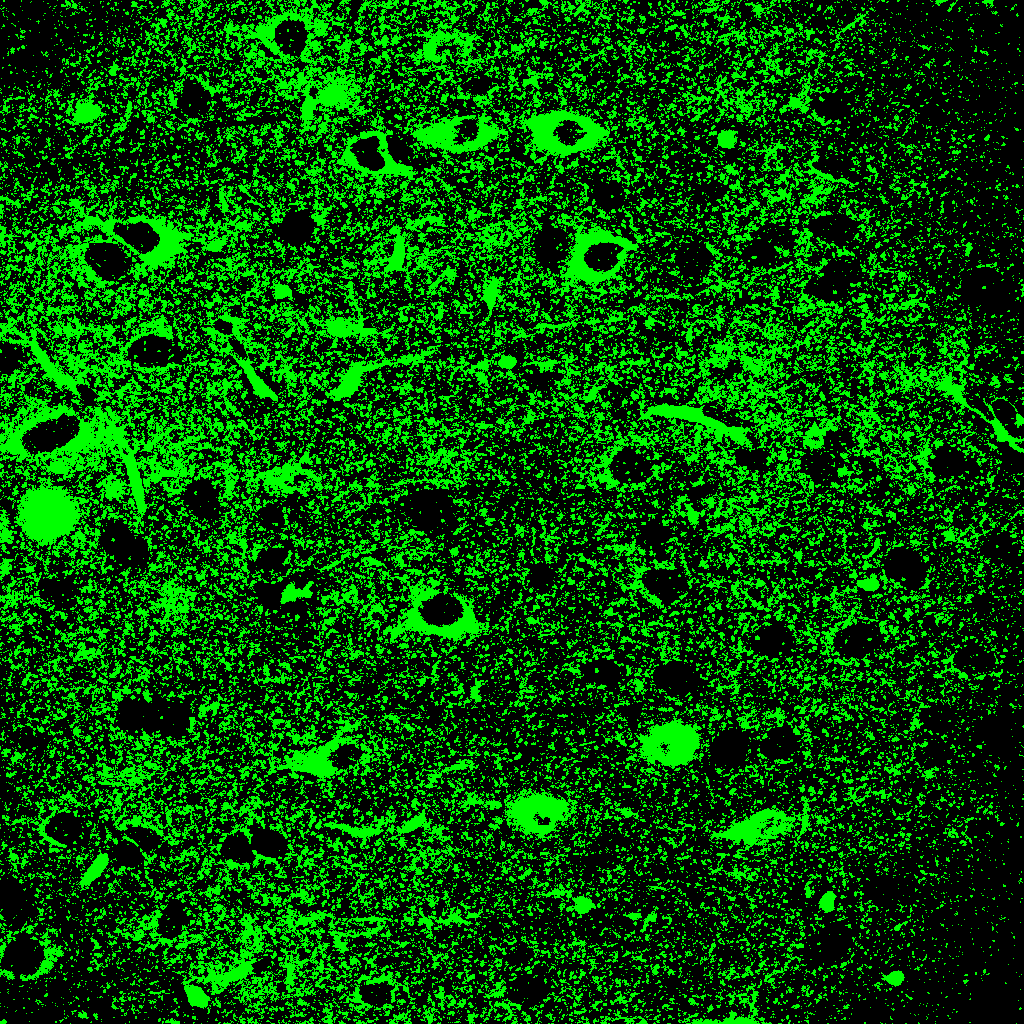** | **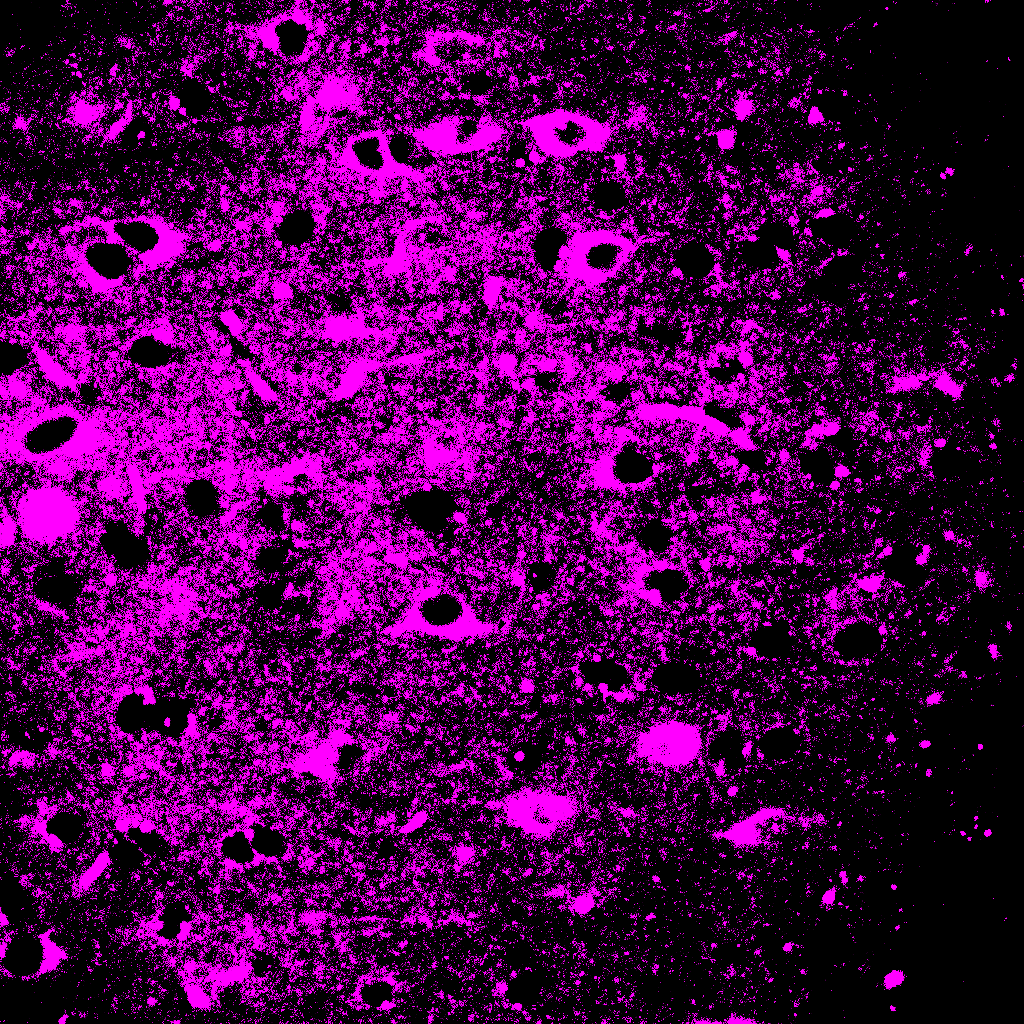** | **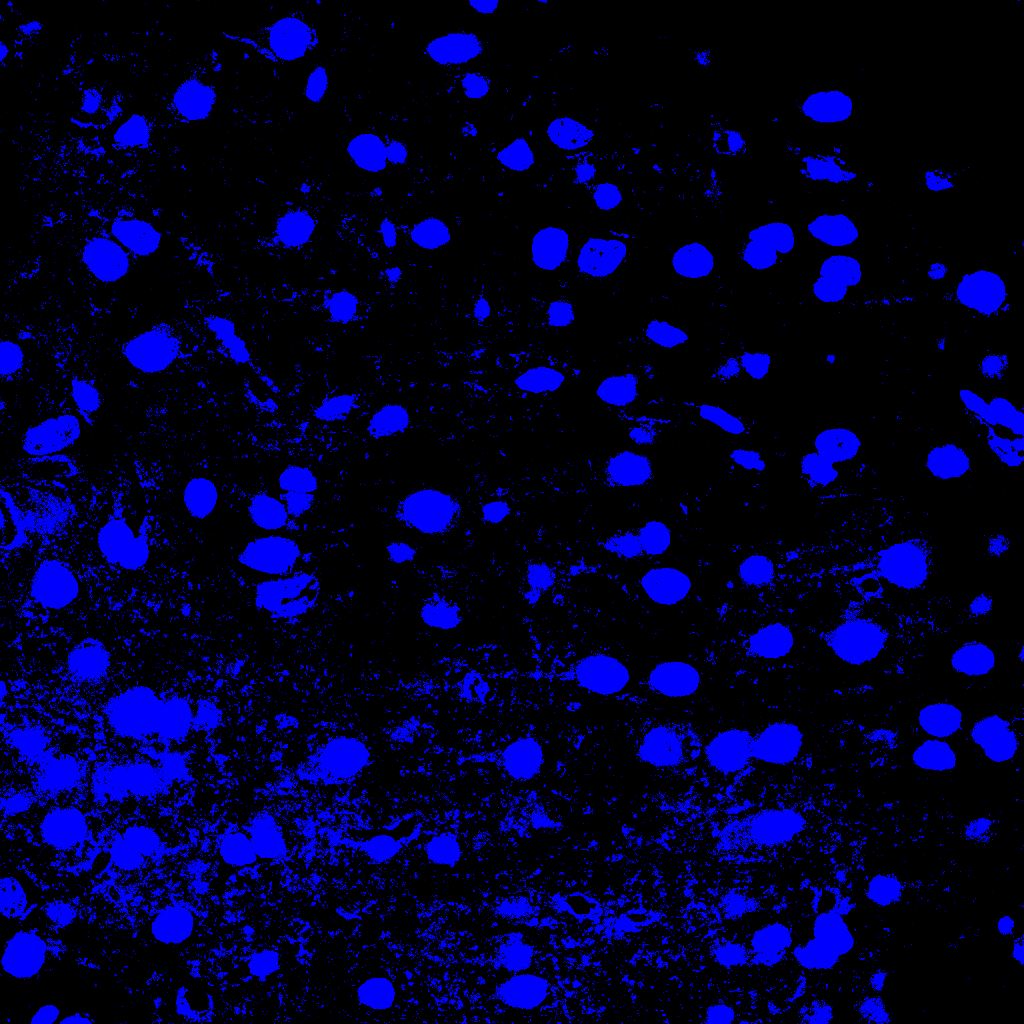** | **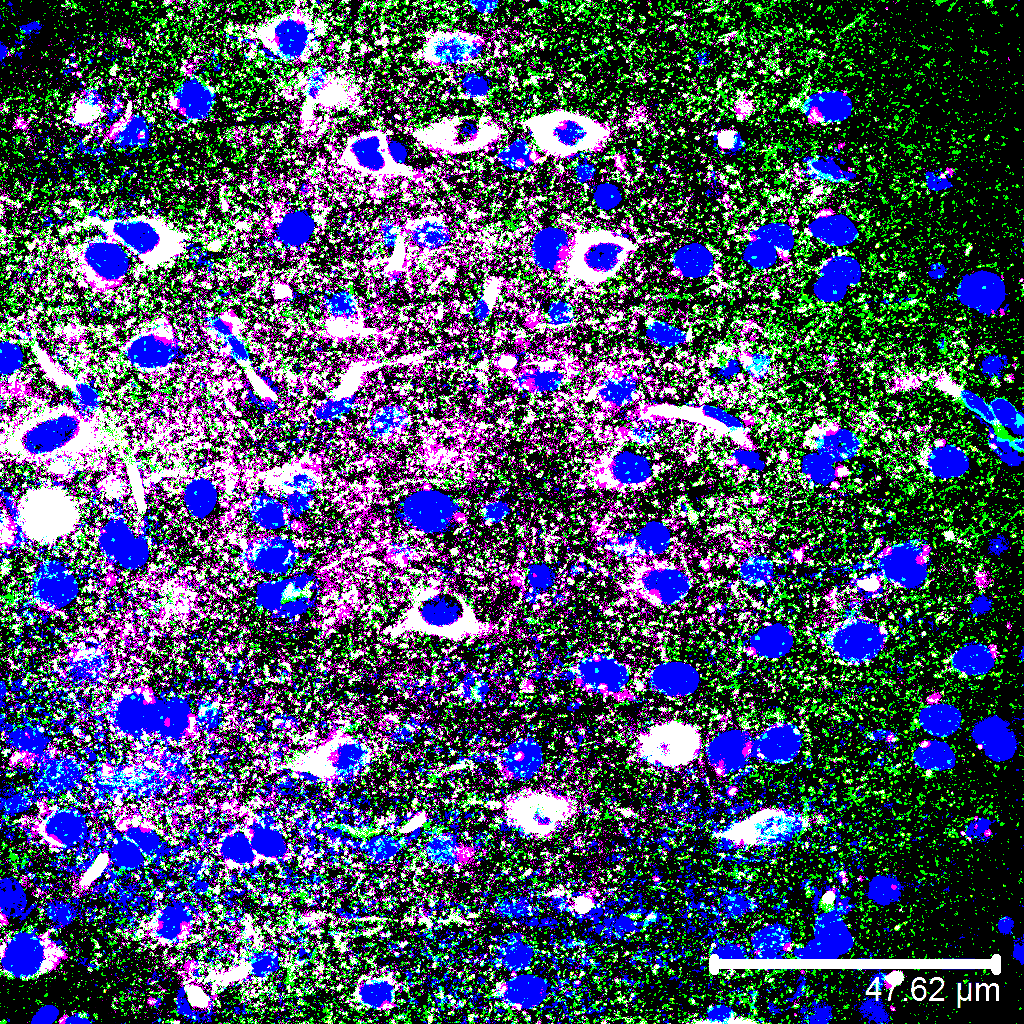** |
| **CCP + IL12Ab** | **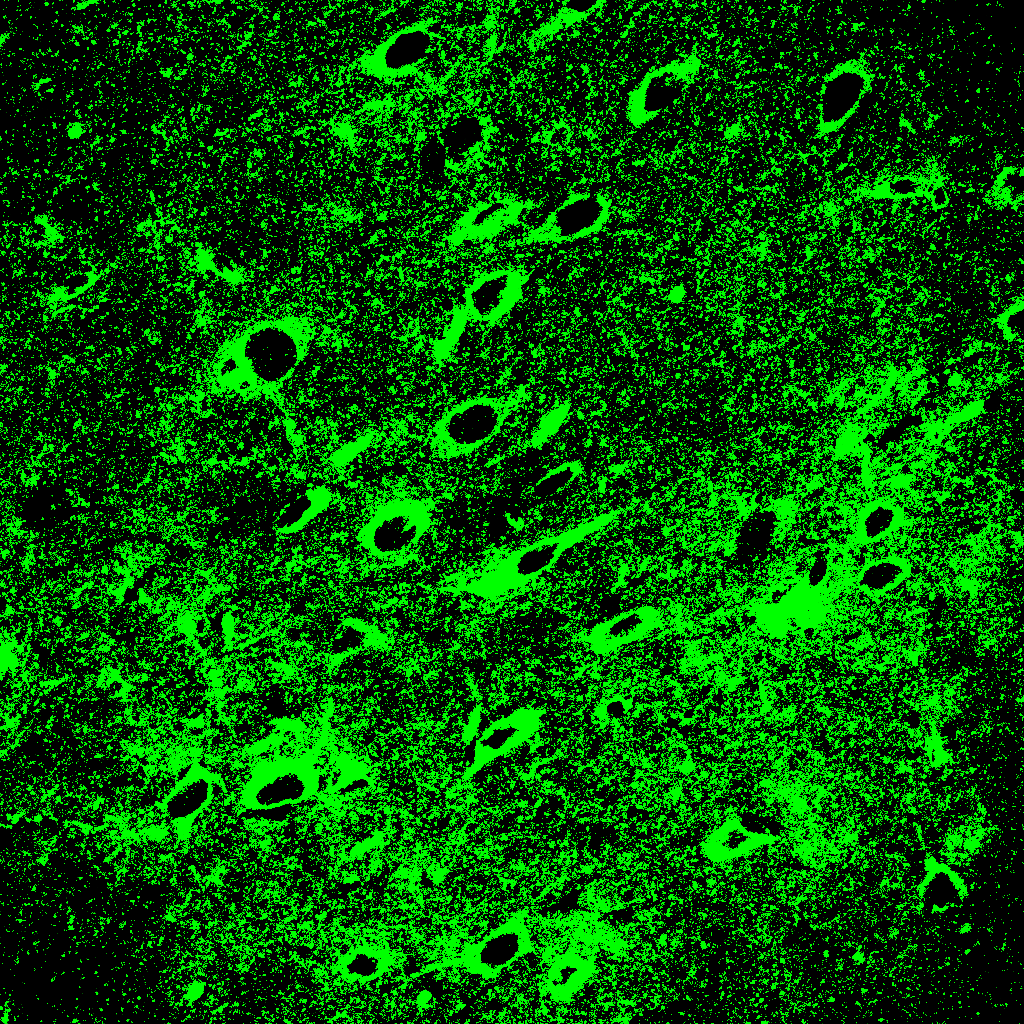** | **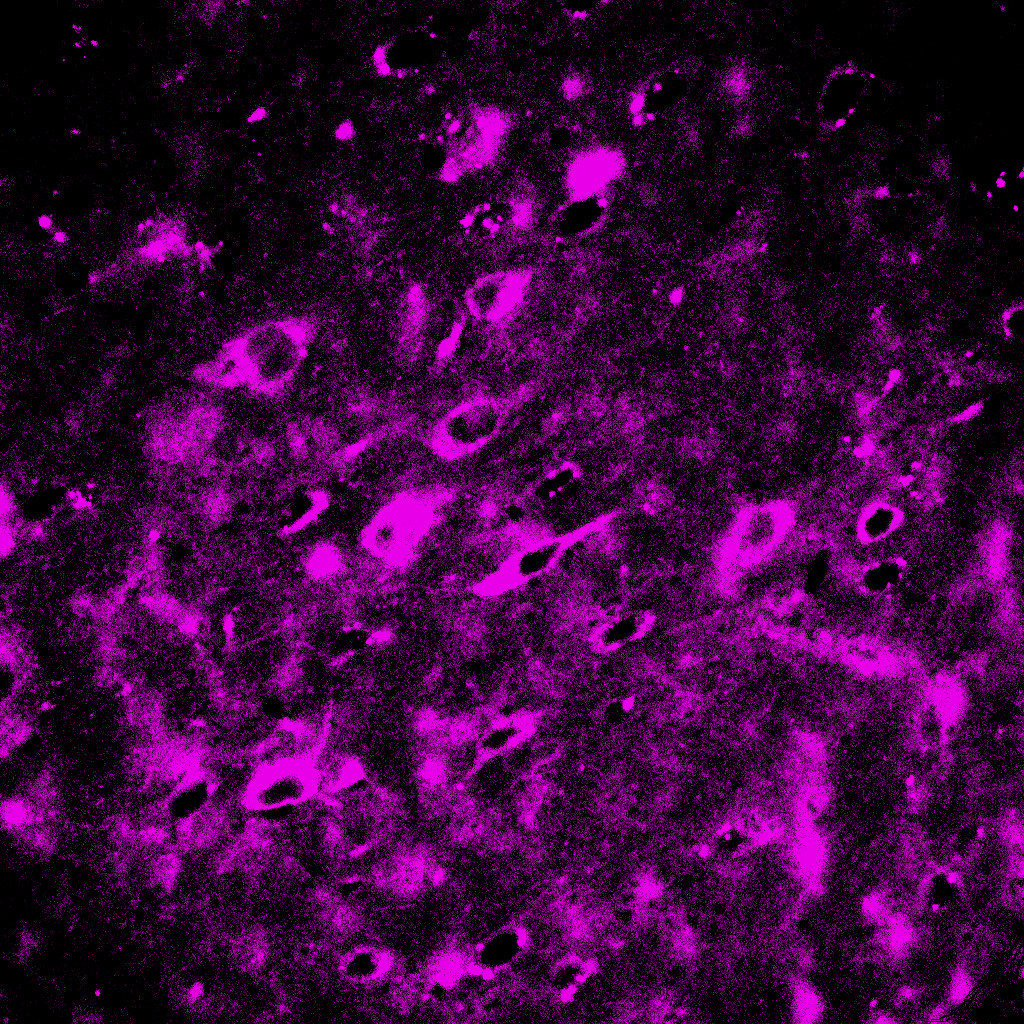** | **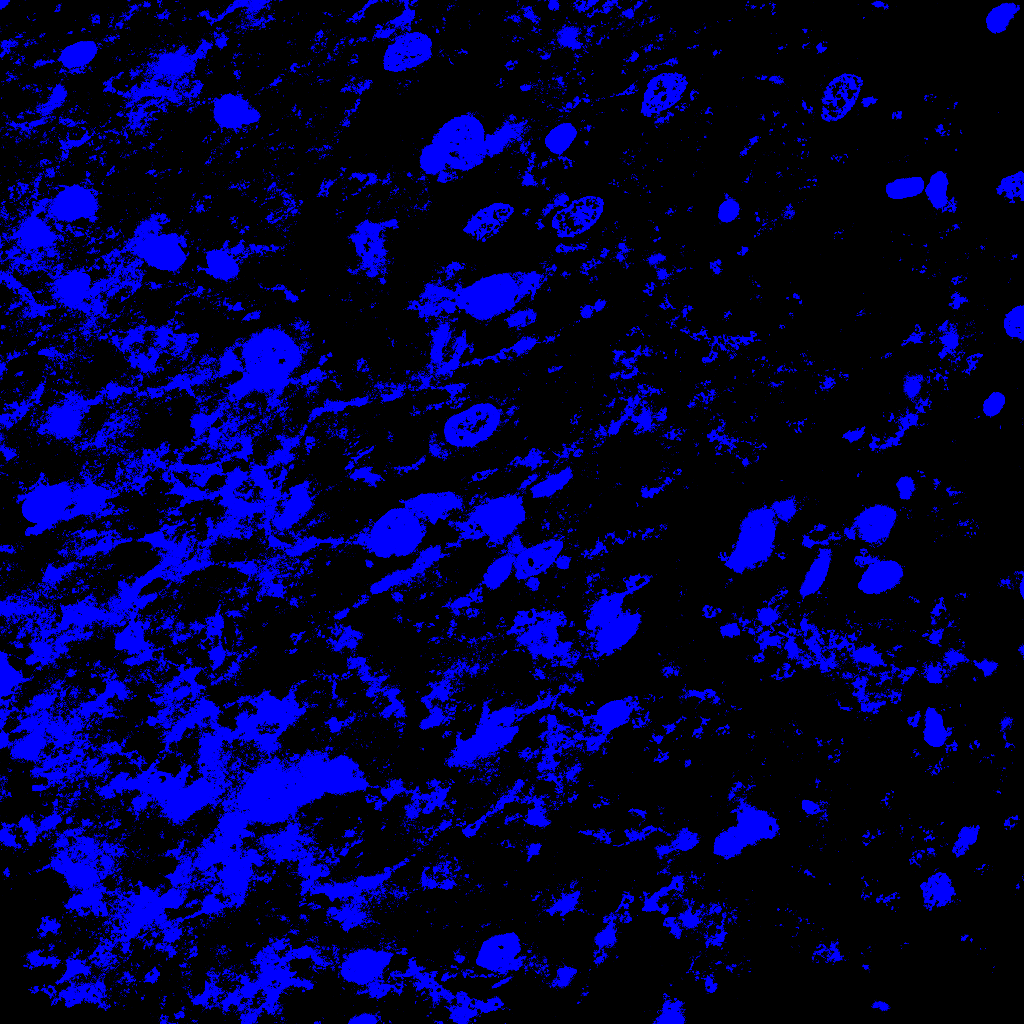** | **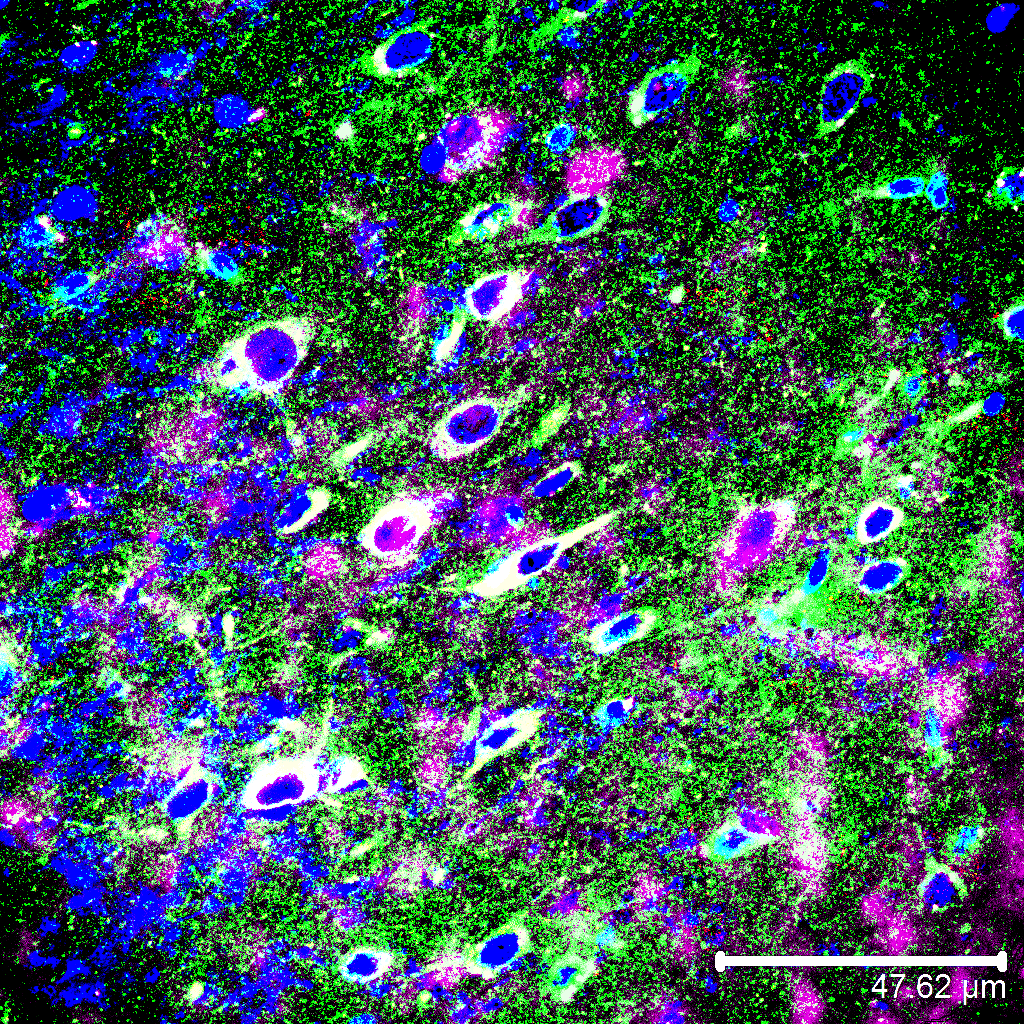** |
|  | **(B)**  **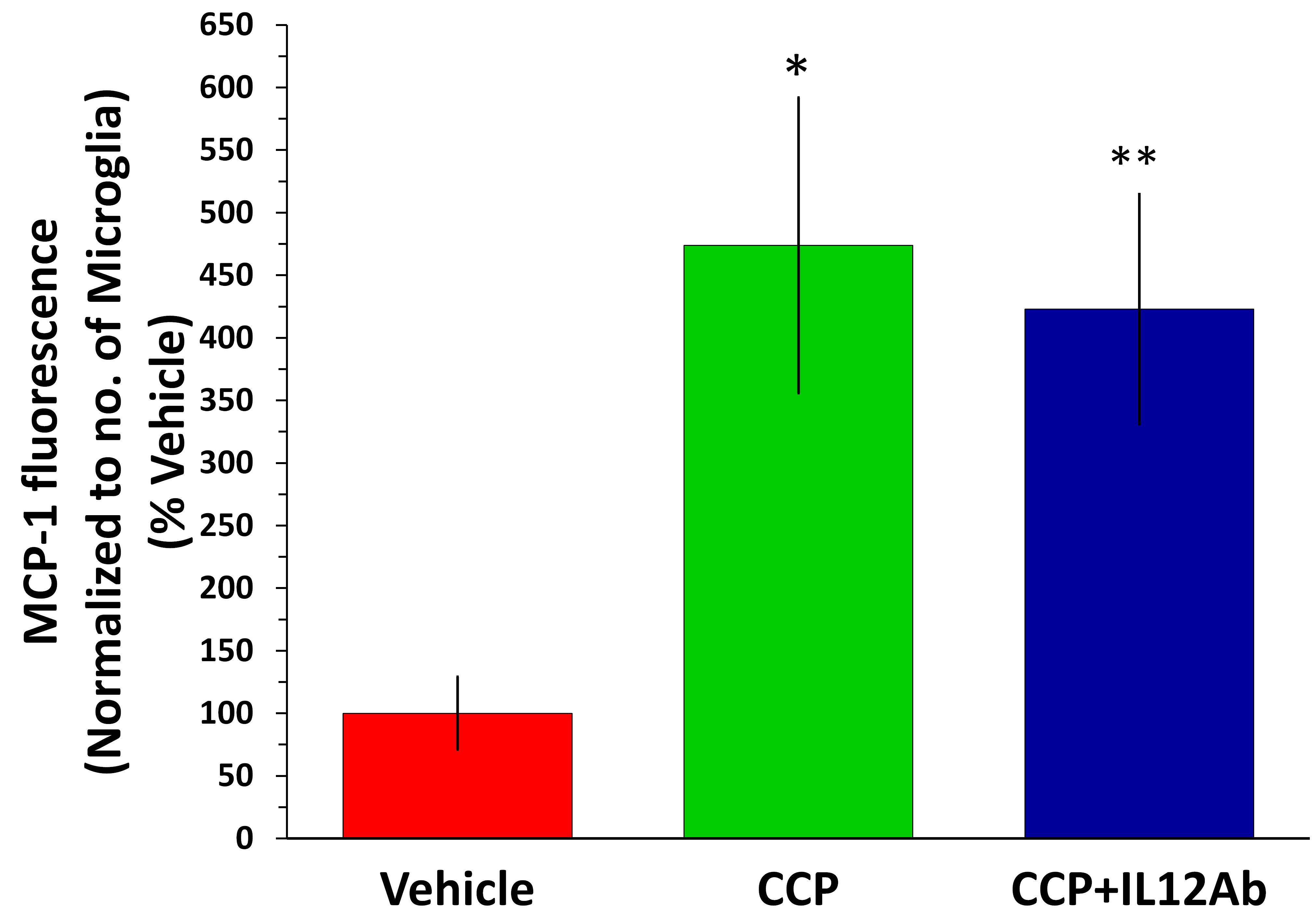** | | | |

We have shown earlier that CCP-treatment of mice causes an induction of activated, p65 NF-kB in GBM TAM [8, 27]. Additionally, p65 NF-kB has been shown to induce MCP-1 expression [81], which is most likely the mechanism of CCP-mediated induction of MCP-1 in the GBM TAM in these mice.
