# Supplementary material for: Phytosomal curcumin causes natural killer cell-dependent repolarization of glioblastoma (GBM) tumor-associated microglia/macrophages and elimination of GBM and GBM stem cells
Source: J Exp Clin Cancer Res. 2018 Jul 25;37:168. doi: 10.1186/s13046-018-0792-5 (PMC6058381; doi:10.1186/s13046-018-0792-5)
Supplement: Supplementary file 12 — Figure S12. Possible signaling pathways involved in intra-GBM recruitment of M1-type macrophages and activated NK cells upon CCP treatment. CCP initiates a complete cycle by causing inhibition of STAT3 in the tumor-associated microglia [82]. This releases STAT1 from the inhibitory effects of STAT3 [83]. Induced P-STAT1 triggers the synthesis of iNOS, IL12, thereby increasing M1-type microglia [62, 63]. MCP-1 released by M1-type microglia compromises the blood-brain barrier, exits into the blood, binds to its receptor (CCR2) on macrophages, polarize them to the M1-type state, and recruits them into GBM in the brain [45, 64, 65, 73, 84, 85]. Meanwhile, M1-type macrophages in blood elicit STAT1-mediated IL12 synthesis and release [43]. The released IL12 binds to IL12 receptor (IL12R) on the NK cells, thereby activating these cells and causing interferon-gamma (IFNγ) release [67]. The released IFNγ causes receptor-mediated inhibition of STAT3 in the macrophages [86–88], which in turn amplifies activated STAT1 and IL12 release [83]. Additionally, IFNγ also causes receptor-mediated activation of STAT1 [89, 90]. This stabilizes the M1 phenotype and the activation of NK cells. Concomitantly, GBM-associated M1-type microglia-released MCP-1 binds to CCR2 on the IL12-activated NK cells [70] and causes recruitment of these cells into the GBM [45]. Once in the GBM, the activated NK cells engage in receptor-mediated interactions with the GBM and GBM stem cells [58, 59], thereby killing GBM and GBM stem cells. Additionally, the activated NK cells also kill resting microglia, thus enriching the M1-type microglia in the TAM [66]. Simultaneously, the M1-type macrophages and microglia within the GBM elicit iNOS-mediated release of nitric oxide (NO) [29, 84], which eliminates GBM and GBM stem cells. (DOC 1280 kb) [file 13046_2018_792_MOESM12_ESM.doc]

**
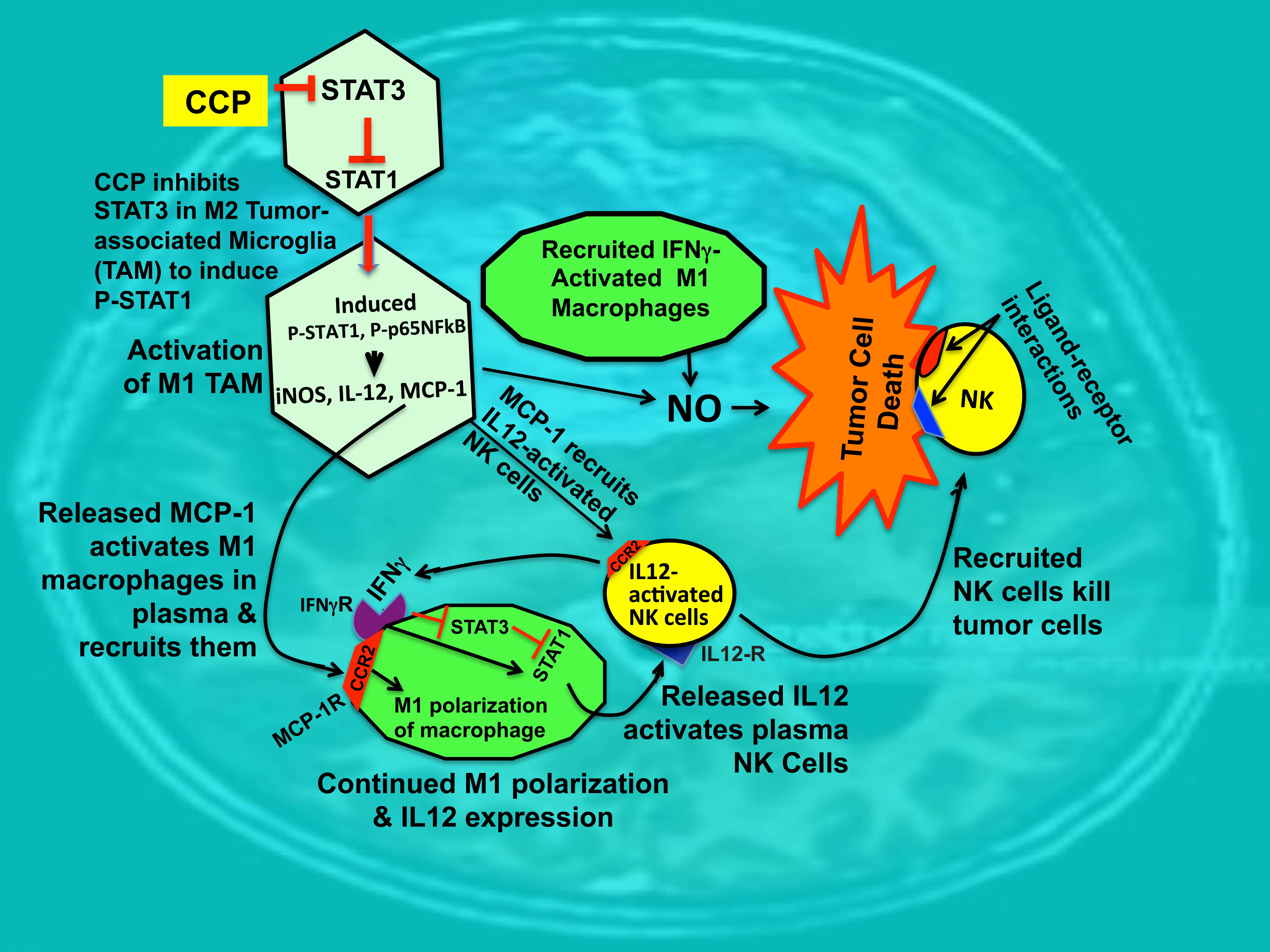
**

**Additional file 12: Figure S12. Possible signaling pathways involved in intra-GBM recruitment of M1-type macrophages and activated NK cells upon CCP treatment.** CCP initiates a complete cycle by causing inhibition of STAT3 in the tumor-associated microglia [82]. This releases STAT1 from the inhibitory effects of STAT3 [83]. Induced P-STAT1 triggers the synthesis of iNOS, IL12, thereby increasing M1-type microglia [62, 63]. MCP-1 released by M1-type microglia compromises the blood-brain barrier, exits into the blood, binds to its receptor (CCR2) on macrophages, polarize them to the M1-type state, and recruits them into GBM in the brain [45, 64, 65, 73, 84, 85]. Meanwhile, M1-type macrophages in blood elicit STAT1-mediated IL12 synthesis and release [43]. The released IL12 binds to IL12 receptor (IL12R) on the NK cells, thereby activating these cells and causing interferon-gamma (IFNγ) release [67]. The released IFNγ causes receptor-mediated inhibition of STAT3 in the macrophages [86-88], which in turn amplifies activated STAT1 and IL12 release [83]. Additionally, IFNγ also causes receptor-mediated activation of STAT1 [89, 90]. This stabilizes the M1 phenotype and the activation of NK cells. Concomitantly, GBM-associated M1-type microglia-released MCP-1 binds to CCR2 on the IL12-activated NK cells [70] and causes recruitment of these cells into the GBM [45]. Once in the GBM, the activated NK cells engage in receptor-mediated interactions with the GBM and GBM stem cells [58, 59], thereby killing GBM and GBM stem cells. Additionally, the activated NK cells also kill resting microglia, thus enriching the M1-type microglia in the TAM [66]. Simultaneously, the M1-type macrophages and microglia within the GBM elicit iNOS-mediated release of nitric oxide (NO) [29, 84], which eliminates GBM and GBM stem cells.
